# Chemoselective Cu-catalyzed acylsilylation of vinyl arenes using silylboronates and acyl fluorides

**DOI:** 10.1039/d5sc05220c

**Published:** 2025-09-10

**Authors:** Zhengyu Zhao, Jun Zhou, Seishu Ochiai, Sota Ikawa, Norio Shibata

**Affiliations:** a Department of Engineering, Nagoya Institute of Technology, Gokiso Showa-ku Nagoya 466-8555 Japan nozshiba@nitech.ac.jp; b Department of Nanopharmaceutical Sciences, Nagoya Institute of Technology, Gokiso Showa-ku Nagoya 466-8555 Japan

## Abstract

We report the chemoselective copper-catalyzed acylsilylation of vinyl arenes using bench-stable silylboronates and acyl fluorides, which enables efficient access to β-silyl ketones under mild conditions. The reaction proceeds without the need for photochemical activation and exhibits a broad substrate scope, tolerating a wide range of electron-deficient and heteroaromatic vinyl arenes, as well as electronically diverse acyl fluorides, including drug-derived motifs. A key to this success is the LUMO-lowering effect of the electron-withdrawing substituents on the aryl ring, which enhances nucleophilic attack by a silylcopper(i) species. Mechanistically, the transformation proceeds *via* a PCy_3_-ligated copper catalyst, mediating Si–B σ-bond transmetalation, alkene insertion, and nucleophilic substitution with acyl fluoride. Notably, acyl fluorides outperform commonly used acid chlorides and acyl imidazoles, offering both improved reactivity and catalyst turnover through the formation of a reactive Cu–F intermediate, which regenerates the active silylcopper species with the concomitant formation of F–Bpin. It is worth noting that this system enables clear discrimination between electronically similar vinyl arenes. The method should be a promising platform for site-selective and chemoselective alkene functionalization in complex settings.

## Abstract 2

We report the chemoselective copper-catalyzed acylsilylation of vinyl arenes using bench-stable silylboronates and acyl fluorides, which enables efficient access to β-silyl ketones under mild conditions.

## Graphical abstract

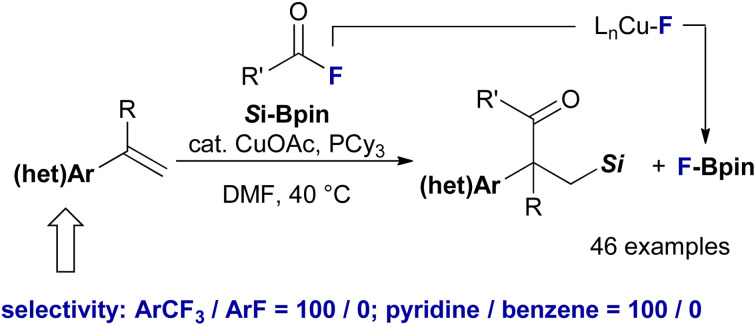

## Introduction

Organosilicon compounds are indispensable in modern chemical science, with applications ranging from advanced materials such as elastomers to pharmaceuticals and agrochemicals.^1^ Their value stems in part from the unique physicochemical properties of the silicon–carbon (C–Si) bond, which can modulate molecular lipophilicity, metabolic stability, and stereochemistry. As a result, the development of efficient synthetic methodologies to form C–Si bonds remains an area of intense interest.^2^ Among these, three-component alkene difunctionalization has emerged as a particularly powerful strategy.^3^ This approach enables the simultaneous installation of a silyl group and a carbon-based fragment across an olefin, rapidly increasing molecular complexity from simple precursors. In this context, silylboronic esters (R_3_SiBpin)^4^ have proven particularly valuable because of their stability, ease of handling, and reactivity in both two- and three-component C–Si bond-forming transformations.

**Fig. 1:**
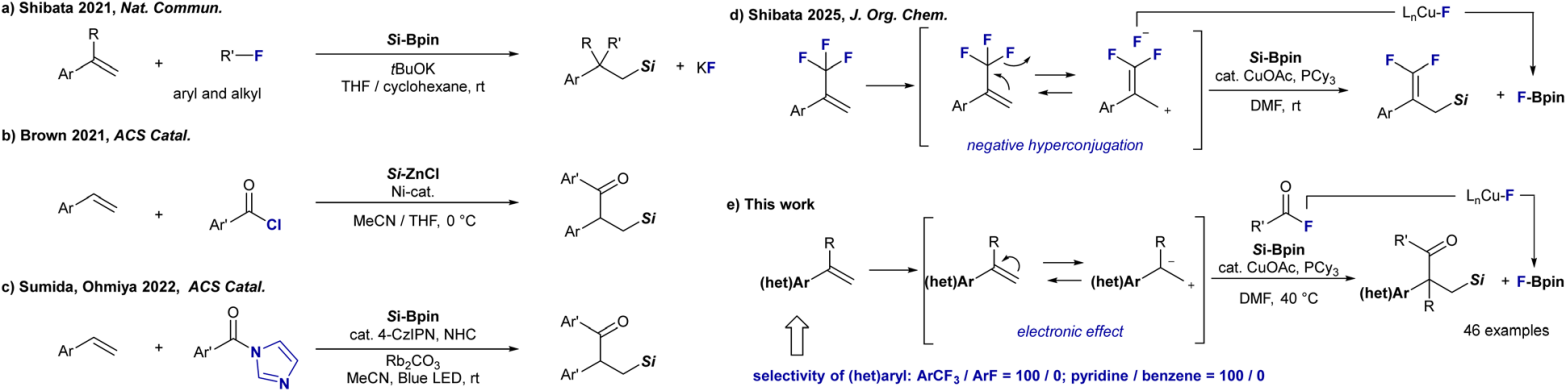
Background and motivation of this work. (a–d) Previous works. (e) Chemoselective acylsilylation of vinyl arenes (this work).

In 2021, our group demonstrated that silylboronates could engage aryl and alkyl fluorides in a catalyst-free defluorinative carbosilylation of vinyl arenes, revealing that even the typically inert C–F bond can cleave to function as a traceless leaving group in alkene functionalization.^5^ In this process, fluoride is efficiently sequestered as potassium fluoride (KF), enabling clean and selective transformations of readily available organic fluorides^6^*via* C–F bond activation^7^ (Fig. 1a). In the same year, Brown and co-workers developed a Ni-catalyzed silylacylation of alkenes using preformed silylzinc reagents and acid chlorides, furnishing β-silyl ketones *via* a nucleophilic [Ni]–SiR_3_ intermediate that undergoes alkene insertion and acyl trapping (Fig. 1b).^8^ More recently, Ohmiya *et al.* reported a visible-light-mediated acylsilylation using R_3_SiBpin as a radical precursor in combination with *N*-heterocyclic carbene (NHC) catalysis, achieving acylsilylation under mild conditions (Fig. 1c).^9^ Despite these notable advances, existing methods still face limitations, including: (i) the need for air- and moisture-sensitive silylzinc reagents; (ii) the use of corrosive and unstable acid chlorides; and (iii) photochemical activation requiring specialized catalyst systems and light sources. Although acyl imidazoles are known to be effective acylating agents, their synthetic accessibility is limited.^10^ To overcome these challenges, we turned our attention to acyl fluorides,^11^ bench-stable electrophiles that are readily accessible *via* deoxyfluorination or oxidative fluorination.^12^ Although the C–F bond in acyl fluorides is typically inert under the standard conditions, it can be selectively activated by π-acidic late transition metals, particularly copper (Cu), which is capable of engaging in oxidative addition to polarized C–F bonds. This makes acyl fluorides attractive partners for catalytic processes that aim for both efficiency and environmental sustainability. Recently, our group reported the Cu-catalyzed defluorosilylation of trifluoromethylalkenes, in which trifluoromethyl (CF_3_) moiety plays a dual mechanistic role: its electron-withdrawing nature and negative hyperconjugation activate the alkene toward nucleophilic attack by the silylcopper species, whereas selective C–F bond cleavage enables C–Si bond formation, accompanied by the generation of a stable F–Bpin byproduct *via* a Cu–F intermediate (Fig. 1d).^13^

Building on this mechanistic foundation, we now report an chemoselective Cu-catalyzed acylsilylation of vinyl arenes using readily available silylboronates and acyl fluorides. This transformation proceeds smoothly at 40 °C under photochemical-free conditions and exhibits broad compatibility with electron-deficient and heteroaromatic vinyl arenes, as well as a diverse range of acyl fluorides, including those derived from drug-like molecules. The reaction delivers β-silyl ketones in up to 99% isolated yield, with excellent chemoselectivity and functional group tolerance. A critical factor for the success of this transformation is the polarization of the alkene moiety in the vinyl arenes with electron-deficient substituents, which lowers the LUMO energy of the alkene and thereby facilitates nucleophilic attack by the silylcopper species. Mechanistically, the reaction is promoted by a PCy_3_-ligated copper catalyst that mediates a sequence of key steps: Si–B σ-bond transmetalation, migratory insertion into the alkene, and nucleophilic substitution with acyl fluoride. The resulting fluoride byproduct is efficiently captured as F–Bpin, formed through a Cu–F intermediate. It is worth noting that this system enables clear discrimination between electronically similar vinyl arenes, such as CF_3_-substituted vinylbenzene *vs. para*-fluorostyrene, and 4-vinylpyridine *vs.* styrene. This level of selectivity highlights the potential of the methodology as a promising platform for site-selective and chemoselective alkene functionalization in complex molecular settings (Fig. 1e).

## Results and discussion

Based on our previous findings on Cu-catalyzed defluorosilylation of trifluoromethylalkenes,^13^ we hypothesized that the alkene component in this three-component coupling should be electronically polarized to enable an efficient reaction. Thus, methyl 4-vinylbenzoate (1a) was selected as a model substrate for optimization, in combination with benzoyl fluoride (2a) and silylboronate, PhMe_2_SiBpin, under copper catalysis (Table 1). Initial experiments were performed under our previously established conditions:^13^ CuOAc (5 mol%) and PCy_3_ (6 mol%) in DMF at room temperature (entry 1). While the reaction proceeded cleanly by TLC monitoring, the conversion was incomplete, and the desired β-silyl ketone 3aa was isolated in 91% yield, with a little amount of unreacted 1a and 2a remaining. Increasing the temperature to 40 °C led to quantitative formation of 3aa (99%), establishing this as the optimal temperature (entry 2). To examine the influence of the copper source, a variety of copper(i) and copper(ii) salts were tested. Both CuCl and CuBr were catalytically inactive, affording no detectable product (entries 3 and 4). Other copper salts such as CuF_2_, Cu(OAc)_2_, [(MeCN)_4_Cu]PF_6_, and Cu(CF_3_COO)_2_·H_2_O gave diminished yields ranging from 32% to 74% (entries 5–8), indicating that CuOAc is uniquely suited for this transformation. To probe metal specificity, Fe(OAc)_2_ and Pd(OAc)_2_ were also examined, but failed to catalyze the reaction (entries 9 and 10), emphasizing the reactivity of copper in this system. Next, we evaluated the effect of various phosphine ligands (entries 11–14). PPh_3_ and P^*t*^Bu_3_ gave moderate yields (35% and 33%, respectively), whereas XPhos (dicyclohexyl[2′,4′,6′-tris(propan-2-yl)[1,1′-biphenyl]-2-yl]phosphane) was entirely ineffective (0%) and DCPE (1,2-bis(dicyclohexylphosphino)ethane) provided only 29% of the product. Finally, control experiments confirmed that both CuOAc and PCy_3_ are essential for the reaction; omitting either component resulted in no product formation, confirming their cooperative role in catalysis (entries 15 and 16).

**Table 1:** Optimization of the reaction conditions^a^

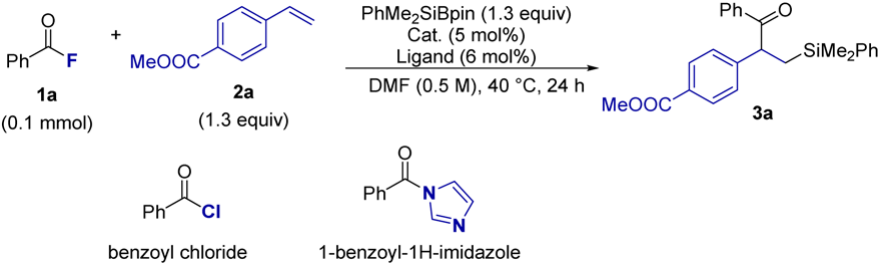			
Entry	Cat.	Ligand	Yield^b^ of 3aa
1^c^	CuOAc	PCy_3_	91%
2	CuOAc	PCy_3_	99% (96%)^d^
3	CuCl	PCy_3_	NR
4	CuBr	PCy_3_	NR
5	CuF_2_	PCy_3_	32%
6	Cu(OAc)_2_	PCy_3_	74%
7	[(MeCN)_4_Cu]PF_6_	PCy_3_	36%
8	Cu(CF_3_COO)_2_·H_2_O	PCy_3_	49%
9	Fe(OAc)_2_	PCy_3_	NR
10	Pd(OAc)_2_	PCy_3_	NR
11	CuOAc	PPh_3_	35%
12	CuOAc	P^*t*^Bu_3_	33%
13	CuOAc	XPhos	NR
14	CuOAc	DCPE	29%
15	—	PCy_3_	NR
16	CuOAc	—	NR
17^e^	CuOAc	PCy_3_	0
18^f^	CuOAc	PCy_3_	68%

a PhMe_2_SiBpin (1.3 equiv), 2a (1.3 equiv), and 1a (0.1 mmol) in DMF, 40 °C for 24 h.

b Determined by trimethyl orthoformate (11.0 μL) as internal standard.

c The reaction was performed at room temperature.

d Isolated yield.

e Reaction was performed using benzoyl chloride (PhCOCl) instead of 1a.

f Reaction was performed using 1-benzoyl-1*H*-imidazole instead of 1a.

To elucidate the unique role of acyl fluoride (1a) in this transformation, we compared its reactivity with other common acyl electrophiles under optimized Cu-catalyzed conditions. Benzoyl chloride, which was previously used in the Ni/Zn system (by Brown, Fig. 1b),^8^ was completely unreactive, affording 0% of the acylsilylation product (entry 17). In contrast, benzoyl imidazole, the electrophile employed by Sumida and Ohmiya under photoredox/NHC conditions (Fig. 1c),^9^ gave a moderate 68% yield (entry 18). These results suggest that, despite its strong bond, the C–F moiety in acyl fluorides serves as the most compatible leaving group in the current copper system.

**Scheme 1:**
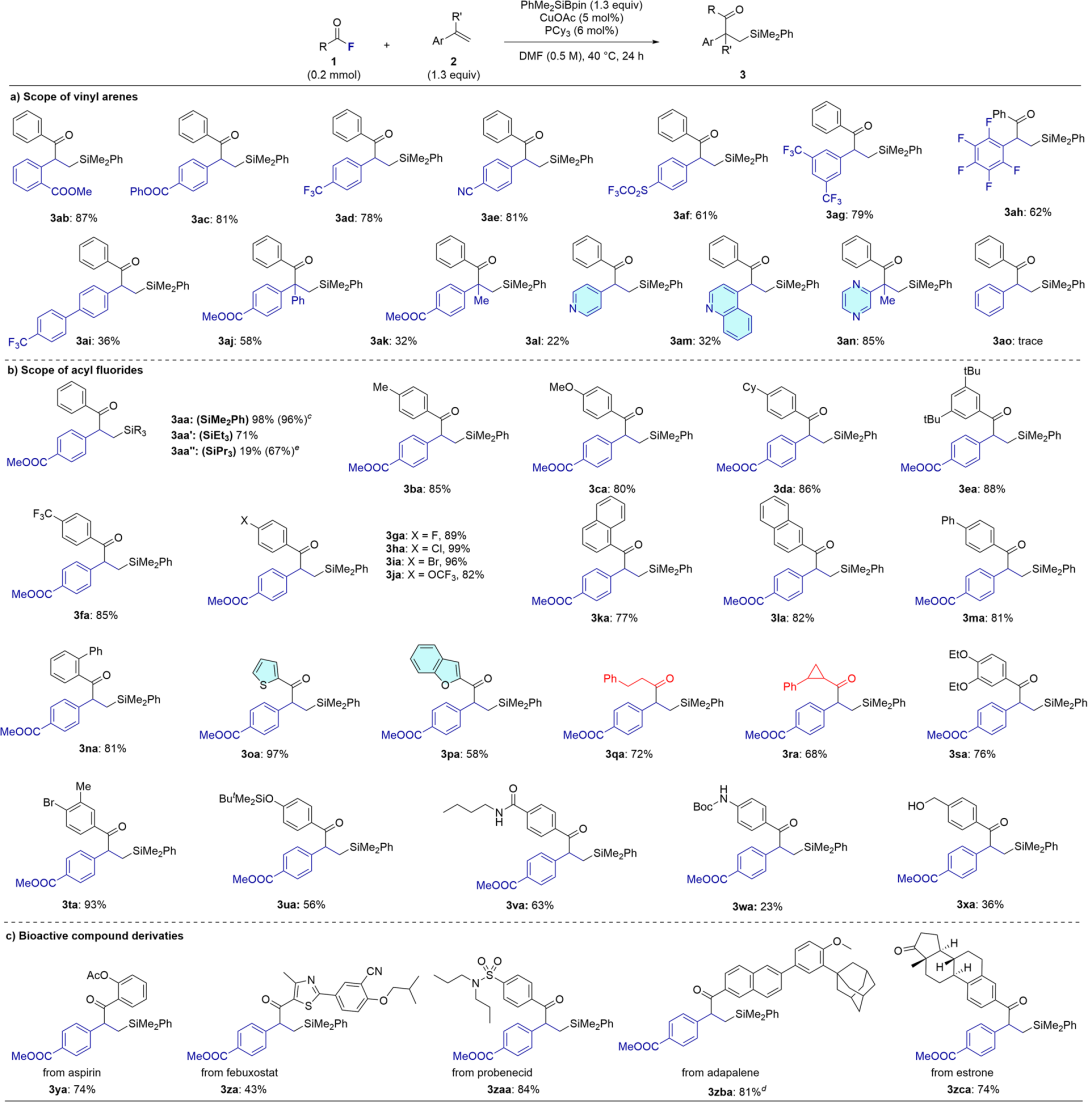
Scope of substrates.^*a*^ Yields of isolated products;^*b*^ unless otherwise noted, the reaction was conducted with CuOAc (5 mol%), PCy_3_ (6 mol%), PhMe_2_SiBpin (1.3 equiv), 2 (1.3 equiv), and 1 (0.2 mmol) in DMF (0.5 M), 40 °C for 24 h;^*c*^ the reaction was performed with 2.0 mmol scale;^*d*^ the reaction was performed at 100 °C;^*e*^ the reaction was performed at 80 °C.

With the optimized reaction conditions established, we explored the substrate scope of the acylsilylation reaction. First, to confirm the hypothesis of the polarization of the alkene moiety in vinyl arenes with substituents, we evaluated various electron-deficient vinyl arenes (2) under optimized conditions (Scheme 1a). Methyl 2-vinylbenzoate (2b) underwent smooth acylsilylation with 1a and PhMe_2_SiBpin, affording 3ab in 87% yield. Phenyl 4-vinylbenzoate (2c) also provided acylsilylation product 3ac in 81% yield. Vinyl arenes bearing CF_3_ (2d, 2g), cyano (2e), and trifluoromethanesulfonyl (2f) groups afforded desired products 3ad–3ag in 61–81% yields. Also, 1,2,3,4,5-pentafluoro-6-vinylbenzene 2h furnished the desired product 3ah in good yield of 62%. Interestingly, even vinyl biphenyl (2i) bearing a remotely positioned CF_3_ group afforded product 3ai in 36% yield, suggesting that electron-withdrawing effects at a distance can still promote the reaction, albeit with reduced efficiency in extended aryl systems. Disubstituted vinyl arenes (2j and 2k) were also examined, delivering 3aj and 3ak in 58% and 32% yields, respectively. Notably, 4-vinylpyridine (2l) underwent the transformation to give 3al in 22% yield, demonstrating the applicability of the protocol to heteroaromatic alkenes. We then tried 2 more *N*-heteroaromatic alkenes, including quinoline and pyrazine, corresponding products obtained with 32% (3am) and 85% (3an), respectively. On the other hand, styrene (2o) showed little to no reactivity under the standard conditions resulting in a trace amount of 3ao detected, importance the critical role of electronic situation on the aromatic ring in facilitating the reaction.

Next, we investigated a range of acyl fluorides (1) in combination with the vinyl arenes derivative 2a to evaluate the generality of the protocol (Scheme 1b). Acyl fluorides bearing electron-donating groups such as methyl (1b), methoxy (1c), and cyclohexyl (1d) at the *para* position of the aryl ring afforded the desired products 3ba–3da in good yields (80–86%). A bulky 3,5-di-*tert*-butyl-substituted acyl fluoride (1e) also performed well, delivering 3ea in 88% yield. Next, we examined substrates bearing electron-withdrawing and halogen substituents including CF_3_ (1f), fluoro (1g), chloro (1h), bromo (1i), and trifluoromethoxy (1j). These gave excellent results with PhMe_2_SiBpin and 2a, affording 3fa–3ja in 82–99% yields. Extended π-systems such as naphthyl and biphenyl derivatives (1k–1m) also participated efficiently, delivering 3ka–3ma in 77–82% yields. Notably, a sterically hindered *ortho*-phenyl-substituted acyl fluoride (1n) gave 3na in 81% yield, suggesting that steric effects are minimal under the optimized conditions. Heteroaryl acyl fluorides, including thiophene (1o) and benzofuran (1p) derivatives, were also compatible, affording 3oa and 3pa in 97% and 58% yields, respectively. Furthermore, aliphatic acyl fluorides (1q and 1r) delivered the corresponding products 3qa and 3ra in 72% and 68% yields, highlighting the versatility of this method. Moreover, disubstituted aryl fluorides (1s and 1t) furnished the desired β-silyl ketones 3sa and 3ta in 76% and 93% yields, respectively. In addition, we examined the reactivity of several challenging substrates. *tert*-Butyldimethylsilyl-protected derivative (1u) afforded the desired product 3ua in a moderate yield of 56%. Substrates bearing functional groups with relatively acidic protons, such as a secondary amide (1v), a secondary amine (1w), and a benzyl alcohol (1x), were also investigated. Among these, substrate 1v furnished the corresponding product in good yield (3va: 63%), whereas 1w and 1x delivered only modest yields (3wa: 23%, 3xa: 36%). We next evaluated alternative silylboranes by replacing PhMe_2_SiBpin with Et_3_SiBpin and *^n^*Pr_3_SiBpin. Both reagents proved suitable for the transformation. The use of Et_3_SiBpin furnished the desired product in good yield (3aa′: 71%), whereas *^n^*Pr_3_SiBpin afforded the corresponding product (3aa′′) in 67% yield, albeit requiring an elevated reaction temperature. The reaction between 1a and 2a was successfully scaled up from 0.2 mmol to 2 mmol, affording 3aa in 96% yield without any loss in efficiency, thereby demonstrating the practicality and scalability of the method.

### Synthetic application I

To demonstrate the synthetic utility of this Cu-catalyzed acylsilylation protocol, we investigated its application in the late-stage functionalization of various pharmaceutical derivatives bearing carbonyl fluoride moiety in the presence of vinyl arene 2a and PhMe_2_SiBpin (Scheme 1c). Structurally simple aspirin-derived acyl fluoride (1y) underwent smooth conversion to the corresponding β-silyl ketone 3ya in 74% yield. In contrast, the more structurally complex febuxostat derivative (1z) afforded a moderate 43% yield of product 3za, likely due to steric or electronic interference. Encouragingly, several other drug-derived acyl fluorides were well tolerated under the reaction conditions. Derivatives of probenecid (1za), adapalene (1zb), and estrone (1zc) furnished the corresponding products 3zaa, 3zba, and 3zca in 84%, 81%, and 74% yields, respectively, indicating broad functional group tolerance and suitability for late-stage diversification of bioactive molecules.

### Synthetic application II

**Scheme 2:**
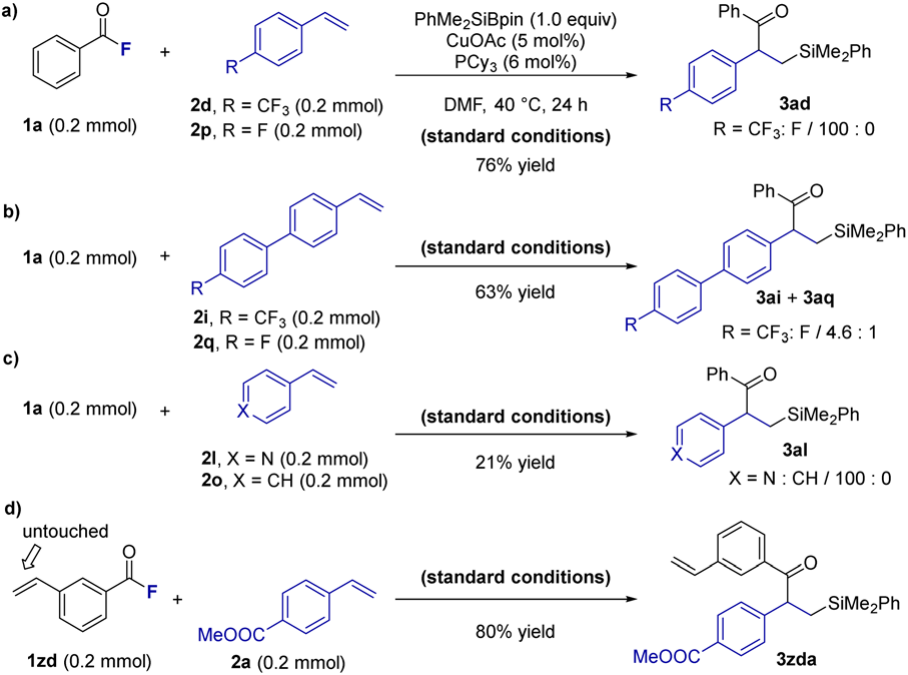
Competitive experiments.^*a*^ Yields of isolated products.

Encouraged by the unique reactivity profile of this chemoselective Cu-catalyzed acylsilylation, particularly its high sensitivity to the electronic nature of the arene moiety as exemplified by the low reactivity of styrene 2o (see Scheme 1), we explored the potential for chemoselective transformation in mixtures of electronically distinct two vinyl arenes 2. We first investigated the competitive chemoselective acylsilylation of *p*-CF_3_ vinyl benzene (2d) and *p*-fluoro vinyl benzene (2p) using benzoyl fluoride (1a) under the standard conditions. Remarkably, only 2d reacted to afford the desired product 3ad in 76% yield, with exclusive selectivity (CF_3_ : F = 100 : 0) (Scheme 2a). This high discrimination suggests that the stronger electron-withdrawing CF_3_ group more effectively activates the alkene for nucleophilic attack by the silylcopper species. This selectivity also extended to biphenyl derivatives bearing remote CF_3_ and F groups. In a competitive experiment, the acylsilylation product 3ai derived from the CF_3_-substituted substrate 2i was obtained in 63% yield with notable chemoselectivity (CF_3_ : F = 4.6 : 1), highlighting the ability to distinguish subtle electronic differences at distant positions (Scheme 2b). Next, we examined heteroarene discrimination. A mixture of 4-vinylpyridine (2l) and styrene (2o) was subjected to the standard reaction with 1a. In this case, only 2l reacted to deliver 3al in 21% yield, with complete selectivity (pyridine : benzene = 100 : 0) (Scheme 2c). This result highlights the high reactivity of heteroarenes in this system. Finally, to test site-selectivity within a single molecule, we subjected acyl fluoride bearing a vinyl arene group (compound 1zd) to acylsilylation conditions in the presence of 4-methoxycarbonyl vinyl benzene (2a). The reaction exclusively furnished product 3zda in 80% yield, with the vinyl group in 1zd remaining untouched (Scheme 2d). These results collectively demonstrate the distinctive electronic discrimination achievable in this system, making it a promising platform for site-selective and chemoselective alkene functionalization in complex settings.

### Synthetic application III

**Scheme 3:**
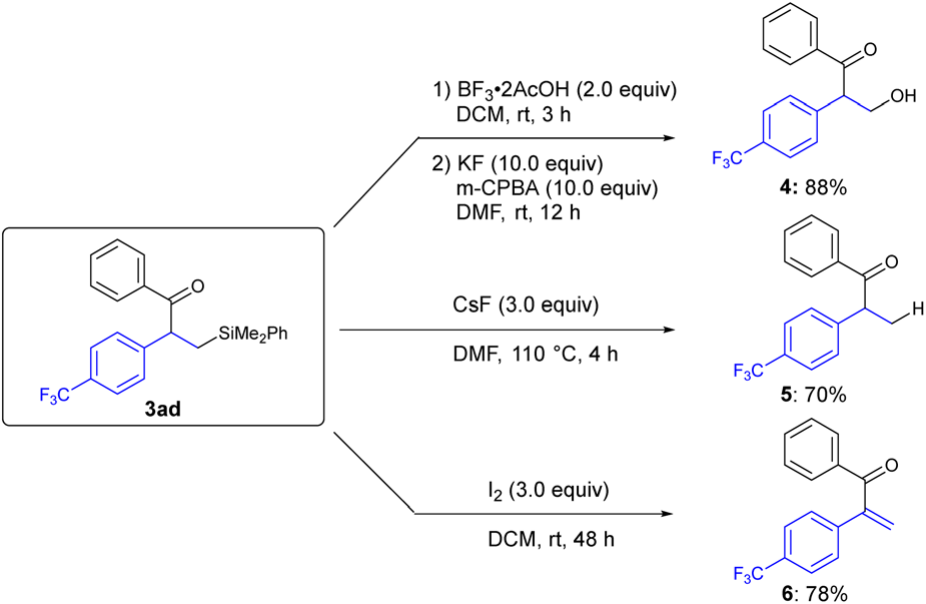
Representative transformations of 3ad^*a*^.^*a*^ Yields of isolated products.

Representative transformations of β-silyl ketone were performed at the C–Si bond while retaining the ketone functionality (Scheme 3). Fleming–Tamao oxidation of 3ad with BF_3_·AcOH and *m*-CPBA afforded β-hydroxy ketone 4 in 88% yield. Desilylation of 3ad with CsF gave product 5 in 70% yield, and iodine-mediated olefination of 3ad furnished α,β-unsaturated ketone 6 in 78% yield.

### Reaction mechanism

**Fig. 2:**
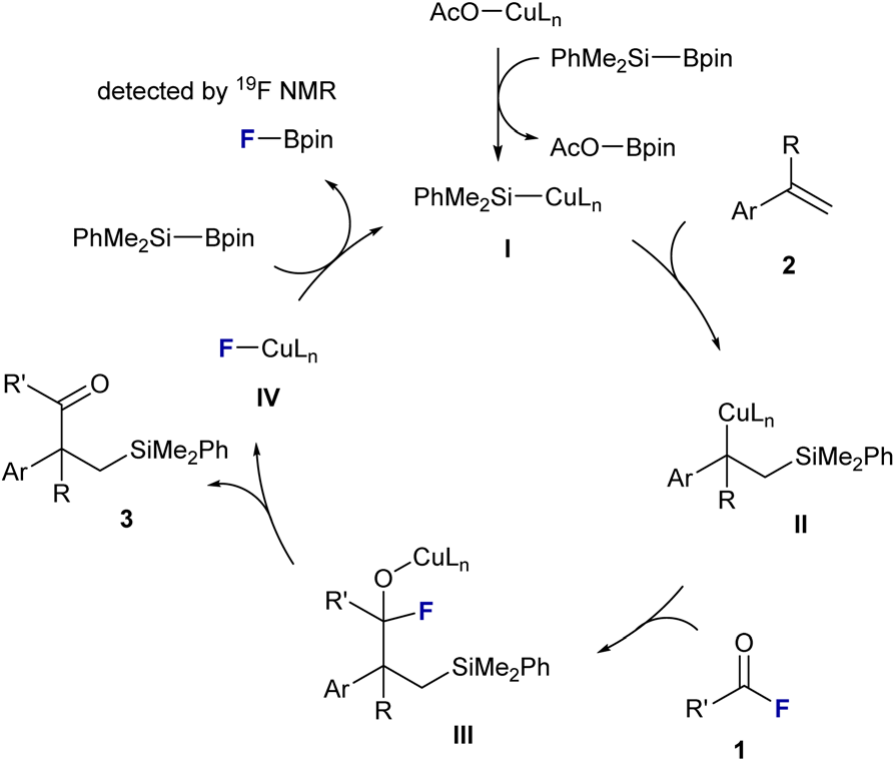
Proposed reaction mechanism.

A plausible catalytic cycle, consistent with these observations, is shown in Fig. 2. The reaction begins with σ-bond metathesis between CuOAc/PCy_3_ and PhMe_2_SiBpin, generating silylcopper(i) species I and releasing AcO–Bpin. Next, migratory insertion of an electron-deficient vinyl arene into the Cu–Si bond affords the corresponding alkyl–copper(i) intermediate II. This intermediate then undergoes nucleophilic acyl substitution with the acyl fluoride. The intrinsic affinity of Cu for fluorine facilitates C–F bond cleavage, forming a putative acyl–copper(i) intermediate III. The strong σ-donating PCy_3_ ligand stabilizes this high-valent species and promotes the substitution event. Subsequently, β-fluoride elimination from III delivers the β-silyl ketone 3 and a Cu(i)–F complex IV. Finally, regeneration of active silylcopper(i) species I occurs *via* a second σ-bond metathesis between IV and PhMe_2_SiBpin, liberating F–Bpin as the byproduct. The formation of F–Bpin was confirmed by ^19^F NMR spectroscopy, supporting the proposed fluoride-transfer pathway.

The superior performance of acyl fluorides in this transformation can be rationalized by two synergistic effects. First, the polarized C–F bond in acyl fluorides undergoes more facile oxidative addition or nucleophilic substitution by copper than the more covalent C–Cl bond in acyl chlorides (see entry 17, Table 1). Second, with respect to catalyst turnover, the resulting Cu–F species IV is readily reactivated by Si–Bpin to regenerate the silylcopper complex, whereas the analogous Cu–Cl species is less reactive in σ-bond metathesis, thereby impeding the catalytic cycle. Together, these mechanistic insights explain the high efficiency of acyl fluorides over acyl chlorides and acyl imidazoles (entries 17 and 18, Table 1) in the present Cu/PCy_3_ system, and emphasize the strategic utility of carbonyl fluorides as ideal electrophiles in copper-catalyzed alkene difunctionalization chemistry.

### Limitations of the study

We explored the applicability of this acylsilylation protocol to alkyl-substituted alkenes beyond the scope of vinyl arenes 2. However, no reaction was observed, highlighting a key limitation of the method. The starting alkenes remained unconsumed, suggesting that the aryl moiety in vinyl arenes 2 is critical for reactivity, particularly in the initial step involving insertion of the Cu–Si species (intermediate I). Without the conjugated aryl group, the alkene likely lacks sufficient electronic activation to undergo migratory insertion, preventing formation of the key alkyl–Cu intermediate (II) (Fig. 2).

## Conclusions

In summary, we have developed a chemoselective copper-catalyzed acylsilylation of electron-deficient vinyl arenes including heteroaromatics using readily available acyl fluorides and silylboronates under mild and operationally simple conditions. This method provides efficient access to a broad range of β-silyl ketones with high chemoselectivity and excellent functional group tolerance, including halogens, nitriles, esters, heterocycles, and pharmaceutically relevant motifs. Even reactive functional groups such amine, amide, and alcohol are compatible with this reaction system. The reaction proceeds *via* a well-defined catalytic cycle involving silylcopper(i) species, migratory alkene insertion, and nucleophilic substitution with the acyl fluoride, followed by β-fluoride elimination. Mechanistic studies highlight the strategic advantage of using acyl fluorides over acyl chlorides and acyl imidazoles, with both improved reactivity and more efficient catalyst turnover. The formation of F–Bpin as the terminal byproduct further supports the proposed fluoride-transfer pathway. The utility of this protocol was demonstrated through the late-stage functionalization of complex pharmaceutical derivatives. The ability to distinguish between electronically similar alkenes—such as CF_3_- *vs.* fluoro-substituted styrenes, or 4-vinylpyridine *vs.* styrene—highlights the potential of this method for precise chemoselective alkene functionalization.

## Author contributions

ZZ, JZ, SO and SI performed the experiments and analyzed the data. ZZ, SO and NS wrote the manuscript. NS supervised the project. All authors contributed to the manuscript and have approved the final version of the manuscript.

## Conflicts of interest

There are no conflicts to declare.

## Supplementary Material

SC-016-D5SC05220C-s001

## Data Availability

The data that support the findings of this study are available within the article and the SI. Supplementary information: Materials and methods, experimental procedures, characterization data, and NMR spectral. See DOI: https://doi.org/10.1039/d5sc05220c.
