# Supplementary material for: Chemoselective Cu-catalyzed acylsilylation of vinyl arenes using silylboronates and acyl fluorides
Source: Chem Sci. 2025 Sep 10;16(40):18679–85. doi: 10.1039/d5sc05220c (PMC12434727; doi:10.1039/d5sc05220c)
Supplement: SC-016-D5SC05220C-s001 [file SC-016-D5SC05220C-s001.pdf]

Supplementary Information (SI) for Chemical Science.
This journal is © The Royal Society of Chemistry 2025

Electronic Supplementary Information

Chemoselective Cu-catalyzed acylsilylation of vinyl arenes using silylboronates and acyl fluorides

Zhengyu Zhao,[a] Jun Zhou,[a] Seishu Ochiai,[a] Sota Ikawa,[a] Norio Shibata [a,b]*

[a]*Department of Engineering, Nagoya Institute of Technology, Gokiso, Showa-ku, Nagoya 466-8555, Japan.*

[b]*Department of Nanopharmaceutical Sciences, Nagoya Institute of Technology, Gokiso, Showa-ku, Nagoya 466-8555, Japan.*

*Correspondence and requests for materials should be addressed to N.S. (email: *nozshiba@nitech.ac.jp*)

Contents

Contents

1. General information and materials 3

2. Reaction condition optimization 4

3. General procedures for synthesis of starting materials 5

3. 1 Synthesis of acyl fluorides 1 from benzoic acids 5

3.2 General procedures for synthesis of vinyl arenes 10

4. General synthetic procedure of silyl boronates 16

5. General procedure 16

5.1 General procedure **A** for reaction conditions optimalization 16

5.2 General procedure **B** for substrate scope 17

5.3 General procedure **C** for larger scale reaction 17

6. Characterization data for products 17

7. Synthetic application 36

7. 1 Tamao-Fleming oxidation using compound **3ad** 36

7. 2 Desilylation of compound **3ad** 37

7. 3 Olefination of compound **3ad** 37

8. NMR experiment for detecting the generation of FBpin 38

9. Chemoselectivity Studies 38

10. References 41

11. NMR Data (^{1}H NMR, ^{13}C NMR and ^{19}F NMR) 42

1. General information and materials

All reactions were performed in oven-dried glassware under a positive pressure of nitrogen or argon. Solvents were transferred via syringe and were introduced into the reaction vessels through a rubber septum. All solvents were dried by standard method. All the reactions were monitored by thin-layer chromatography (TLC) carried out on 0.25 mm Merck silica gel (60-F254). The TLC plates were visualized with UV light. All the reaction products were purified by column chromatography and was carried out on a column packed with silica gel 60N spherical neutral size 50-63 mm. The ^{1}H NMR (300 MHz and 500 MHz) was recorded on Varian Mercury 300, BRUKER 500 Ultra Shield TR, and JEOL ECZ700R; ^{19}F NMR (41 MHz, 282 MHz and 658 MHz) spectra as for solution in $CDCl_3$ were recorded on Spinsolve 90, Varian Mercury 300 and JEOL ECZ700R; ^{13}C NMR (126 MHz and 176 MHz) spectra for solution in $CDCl_3$ was recorded on a BRUKER Avance 500 and JEOL ECZ700R. The chemical shifts (δ) are expressed in ppm downfield from internal TMS (δ = 0.00) and coupling constants (*J*) are reported in hertz (Hz). The hexafluorobenzene (C_6F_6) [δ = −162.2 ($CDCl_3$)] was used as internal standard for ^{19}F NMR. TMS ($SiMe_4$) [δ = 0 ($CDCl_3$)] was used as internal standard for ^{29}Si NMR. The following abbreviations were used to explain the multiplicities: s = singlet, d = doublet, t = triplet, q = quartet, m = multiplet, br = broad. Mass spectra were recorded on a JEOL JMS-Q1050GC (EI-MS). High resolution mass spectrometry (HRMS) was carried out on an electron impact ionization mass spectrometer with a micro-TOF analyzer and recorded on a Waters, GCT Premier (EI-MS) with a TOF analyzer. Melting points were recorded on a BUCHI M-565.

Commercially available chemicals were obtained from Aldrich Chemical Co., Alfa Aesar, TCI and used as received unless otherwise noted. Solvent acetonitrile, DME, Dioxane, DMF and THF were dried and distilled before use.

2. Reaction condition optimization

Table S1. Optimization of reaction conditions[a]

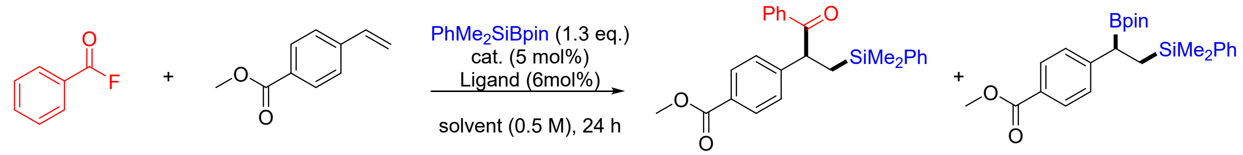

1a **2a** **3aa** **4**

Entry	cat.	Ligand	Solvent	T (°C)	Yield of 3aa[b]	Yield of 4[b]
1	CuOAc	PCy_3	DMF	60	99%(99%)[d]	-
2	CuOAc	PCy_3	THF	60	77%	14%
3	CuOAc	PCy_3	MeCN	60	73%	-
4	CuOAc	PCy_3	c-hex	60	88%	42%
5	CuOAc	PCy_3	Dioxane	60	80%	37%
6	CuOAc	PCy_3	DCM	60	85%	-
7	CuOAc	PCy_3	Toluene	60	83%	23%
8	CuOAc	PCy_3	DMF	50	94%	-
9	CuOAc	PCy_3	DMF	40	99%(96%)[d]	-
10	CuOAc	PCy_3	DMF	rt	91%	-
11	-	PCy_3	DMF	40	NR	-
12	CuOAc	-	DMF	40	NR	-
13	$Cu(OAc)_2$	PCy_3	DMF	40	74%	-
14	CuCl	PCy_3	DMF	40	NR	-
15	CuBr	PCy_3	DMF	40	NR	-
16	$Fe(OAc)_2$	PCy_3	DMF	40	NR	-
17	$Pd(OAc)_2$	PCy_3	DMF	40	NR	-
18	$Cu(OAc)_2/PCy_3$(5/12)[c]	PCy_3	DMF	40	86%	-
19	$CuSCF_3$	PCy_3	DMF	40	NR	-
20	$Cu(CF_3COO)_2\cdot H_2O$	PCy_3	DMF	40	49%	-
21	$[(MeCN)_4Cu]PF_6$	PCy_3	DMF	40	36%	-
22	CuOAc	PPh_3 (**L1**)	DMF	40	35%	14%
23	CuOAc	P^tBu_3 (**L2**)	DMF	40	33%	-
24	CuOAc	**L3**	DMF	40	29%	-
25	CuOAc	Xphos (**L4**)	DMF	40	NR	-
26	CuOAc	**L5**	DMF	40	trace	5%

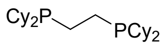

L3

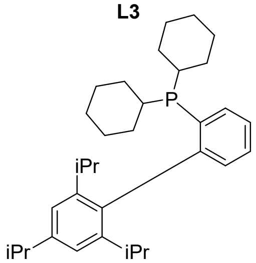

L4

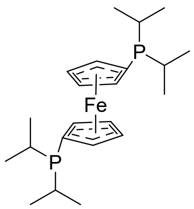

L5

→

[a]Reaction conditions: **1a** (0.1 mmol), **2a** (1.3 equiv.), $PhMe_2SiBpin$ (1.3 equiv.) and cat. (5 mol%), Ligand (6 mol%) in Solvent (0.5 M) react for 24 h; [b]Determined by ^1H NMR spectroscopy using trimethyl orthoformate as internal standard; [c]Ligand (12 mol%) was used. [d]Isolated yields.

3. General procedures for synthesis of starting materials

3. 1 Synthesis of acyl fluorides 1 from benzoic acids

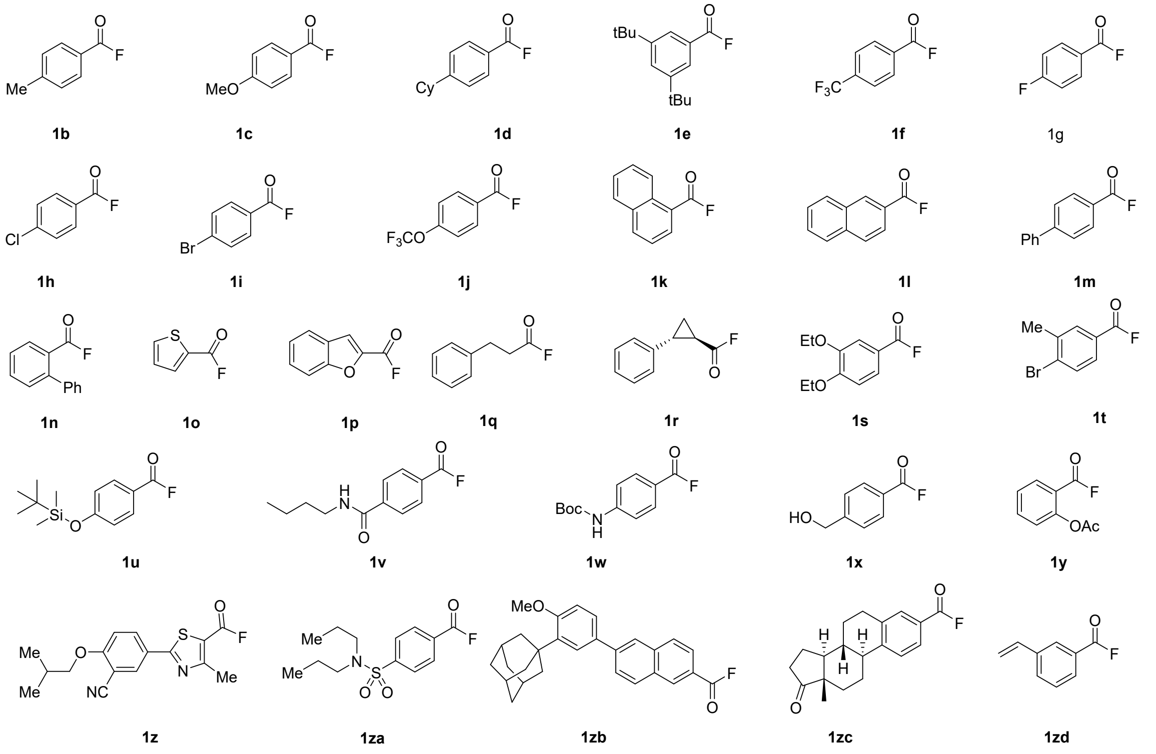

Acyl fluoride **1a** was purchased from TCI, **1b**-**1p**, **1s**, **1t**, **1v**, **1w**, **1y** compounds were synthesized were prepared according to reported methods[1-3], and preparation experimental procedures of **1q**, **1r**, **1u**, **1x**, **1z** were described below.

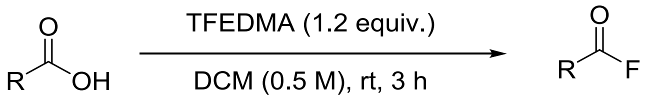

To stirred solution of corresponding carboxylic acid in dichloromethane (DCM) under nitrogen atmosphere was added TFEDMA (1.2 equiv) at room temperature. The reaction mixture was stirred for 3 hours at the same temperature, after the reaction completed, quench it with sat. aq. $NaHCO_3$, extract with DCM for 3 times. The combined organic phases were washed with brine and dried over Na_2SO_4. After filtration, the filtrate was concentrated under reduced pressure and the residue was purified by column chromatography on silica gel to afford the corresponding acyl fluoride products.

3-phenylpropanoyl fluoride (**1q**)

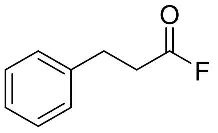

Following the above procedure, the reaction was conducted on a 10 mmol scale, **1q** was purified by column chromatography on silica gel (*n*-hexane/EtOAc 10/1) as a colorless oil. (1.02 g, 67% yield).

^{1}H NMR (500 MHz, $CDCl_3$) δ 7.32 (t, *J* = 7.6 Hz, 2H), 7.26 – 7.21 (m, 3H), 2.99 (t, *J* = 7.6 Hz, 2H), 2.83 (t, *J* = 7.6 Hz, 2H).
^{19}F NMR (282 MHz, $CDCl_3$) δ -44.7 (s, 1F).
MS(EI): m/z 152 $[M]^+$.
The chemical shifts were consistent with reported literature.[4]

trans-2-phenylcyclopropane-1-carbonyl fluoride (**1r**)

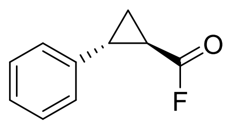

Following the above procedure, the reaction was conducted on a 4.3 mmol scale, 1r was purified by column chromatography on silica gel (*n*-hexane/EtOAc 10/1) as a colorless oil. (434 mg, 61% yield).

^{1}H NMR (500 MHz, $CDCl_3$) δ 7.31 (t, *J* = 6.2 Hz, 2H), 7.28 – 7.20 (m, 1H), 7.11 (d, *J* = 7.4 Hz, 2H), 2.73 (dt, *J* = 8.9, 4.1 Hz, 1H), 1.96 – 1.84 (m, 1H), 1.76 (dt, *J* = 9.8, 5.1 Hz, 1H), 1.64 – 1.51 (m, 1H).
^{13}C NMR (126 MHz, $CDCl_3$) δ 163.78 (d, *J* = 345.9 Hz), 138.09, 128.82, 127.39, 126.46, 28.18, 21.55 (d, *J* = 72.9 Hz), 17.93.
^{19}F NMR (282 MHz, $CDCl_3$) δ 35.2 (s, 1F).
HRMS (ESI) $[C_{11}H_{13}O_2]$ $[M+OMe+H]^+$ for corresponding methyl ester, calculated for: 177.0910, found: 177.0914.

tert-butyl (4-(fluorocarbonyl)phenyl)carbamate **(1w)**

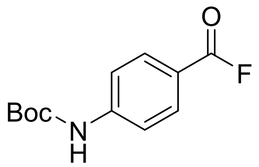

Following the above procedure, the reaction was conducted on a 2 mmol scale, **1w** was purified by column chromatography on silica gel (*n*-hexane/EtOAc 10/1) as a white solid. (337 mg, 70% yield).

m.p. = 91.4 – 92.2℃
^{1}H NMR (500 MHz, $CDCl_3$) δ 7.97 (d, *J* = 8.8 Hz, 2H), 7.52 (d, *J* = 8.4 Hz, 2H), 6.82 (s, 1H), 1.54 (s, 9H).
^{13}C NMR (176 MHz, $CDCl_3$) δ 157.3 152.1, 145.2, 133.1, 118.6, 117.8, 81.9, 28.3.
^{19}F NMR (658 MHz, $CDCl_3$) δ 16.3 (s, 1F).

HRMS (ESI) [$C_{13}H_{17}NO_4Na$] [M+OMe+Na]$^+$ for corresponding methyl ester, calculated for: 274.1049, found: 274.1057.

4-(hydroxymethyl)benzoyl fluoride (**1x**)

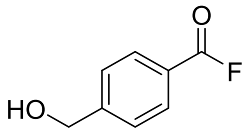

Following the above procedure, the reaction was conducted on a 4 mmol scale, **1x** was purified by column chromatography on silica gel (*n*-hexane/EtOAc 3/1) as a white solid. (52.1 mg, 8% yield).

^{1}H NMR (500 MHz, $CDCl_3$) δ 8.04 (d, *J* = 8.26 Hz, 2H), 7.53 (d, *J* = 7.33 Hz, 2H), 4.83 (s, 2H).
^{19}F NMR (658 MHz, $CDCl_3$) δ 18.28.
MS(EI): m/z 154 [M]$^+$.
The chemical shifts were consistent with reported literature.[6]

2-(fluorocarbonyl)phenyl acetate (**1y**)

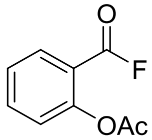

Following the above procedure, the reaction was conducted on a 5 mmol scale, **1y** was purified by column chromatography on silica gel (*n*-hexane/EtOAc 10/1) as a colorless oil. (248 mg, 27% yield).

^{1}H NMR (300 MHz, $CDCl_3$) δ 8.06 – 8.03 (m, 1H), 7.75 – 7.69 (m, 1H), 7.41 (t, *J* = 8.1 Hz, 1H), 7.21 (d, *J* = 8.2 Hz, 1H), 2.38 (s, 3H).
^{19}F NMR (282 MHz, $CDCl_3$) δ 28.01 (s, 1F).
MS(EI): m/z 182 [M]$^+$.
The chemical shifts were consistent with reported literature.[5]

6-(3-(adamantan-1-yl)-4-methoxyphenyl)-2-naphthoyl fluoride (**1zb**)

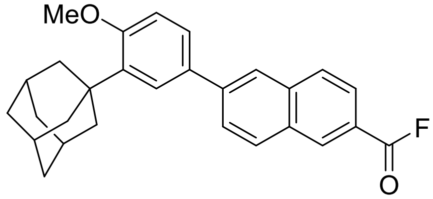

Following the above procedure, the reaction was conducted on a 2.4 mmol scale, **1zb** was purified by column chromatography on silica gel (*n*-hexane/EtOAc 10/1) as a white solid. (732 mg, 74% yield).

m.p. = 228.4 – 228.9 °C.
^{1}H NMR (500 MHz, $CDCl_3$) δ 8.63 (d, *J* = 1.7 Hz, 1H), 8.08 – 7.94 (m, 4H), 7.86 (dd, *J* = 8.5, 1.8 Hz, 1H), 7.61 (t, *J* = 1.8 Hz, 1H), 7.56 (dd, *J* = 8.4, 2.4 Hz, 1H), 7.01 (d, *J* = 8.4 Hz, 1H), 3.91 (s, 3H), 2.18 (s, 6H), 2.11 (s, 3H), 1.80 (s, 6H).
^{13}C NMR (126 MHz, $CDCl_3$) δ 159.33, 156.54, 143.00, 139.27, 137.07, 133.96, 132.16, 131.13, 130.14, 129.20, 127.30, 126.15, 125.99, 124.91, 121.72, 121.24, 112.26, 55.31, 40.71, 37.36, 37.23, 29.21.
^{19}F NMR (282 MHz, $CDCl_3$) δ 17.63 (s, 1F).
HRMS (ESI) [$C_{28}H_{27}FO_2Na$] [M+Na]$^+$ calculated for: 437.1887, found: 437.1880.

3-vinylbenzoyl fluoride (**1zd**)

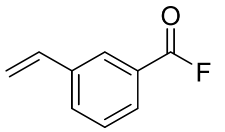

Following the above procedure, the reaction was conducted on a 2 mmol scale, 1q was purified by column chromatography on silica gel (*n*-hexane/EtOAc 10/1) as a colorless oil. (233 mg, 76% yield).

^{1}H NMR (500 MHz, $CDCl_3$) δ 8.1 (t, *J* = 1.9 Hz, 1H), 7.9 (dt, *J* = 7.7, 1.5 Hz, 1H), 7.7 (dt, *J* = 7.8, 1.5 Hz, 1H), 7.5 (t, *J* = 7.8 Hz, 1H), 6.8 (dd, *J* = 17.6, 10.9 Hz, 1H), 5.9 (d, *J* = 17.5 Hz, 1H), 5.4 (d, *J* = 10.9 Hz, 1H).
^{13}C NMR (126 MHz, $CDCl_3$) δ 157.5 (d), 138.8, 135.3, 132.9, 130.6 (d), 129.4, 129.2 (d), 125.4 (d), 116.5.
^{19}F NMR (282 MHz, $CDCl_3$) δ 18.0 (s, 1F).
HRMS (ESI) [$C_9H_7O_2$] [M-H]$^-$ for corresponding carboxylic acid, calculated for: 147.0452, found: 147.0446.

4-(butylcarbamoyl)benzoyl fluoride (**1v**)

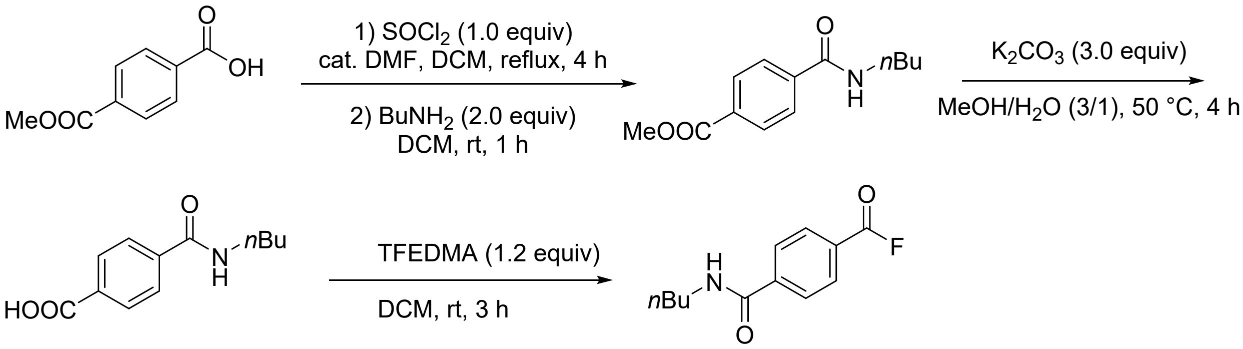

An oven-dried 100 mL round-bottom flask equipped with a magnetic stir bar was charged with 4-(methoxycarbonyl)benzoic acid (3.60 g, 20.0 mmol), DCM(50 mL), and DMF (3 drops) at ambient temperature. Thionyl chloride (2.40 g, 1.00 equiv) was added, and the reaction mixture was heated to reflux for 4 h. After cooling to room temperature, the reaction mixture was concentrated under reduced pressure to afford the crude 4-(methoxycarbonyl)benzoyl chloride, which was used without further purification. The residue was dissolved in DCM (40 mL), and n-butylamine (4.00

mL, 2.00 equiv) was added dropwise at ambient temperature. The mixture was stirred for 1 h, diluted with EtOAc (50 mL), and washed sequentially with water twice and 1 M HCl. The combined organic layers were dried over anhydrous Na_2SO_4, filtered, and concentrated under reduced pressure. The crude product was recrystallized from diethyl ether/hexane to afford *N*-butyl-4-(methoxycarbonyl)benzamide as a white solid (2.90 g, 62%).

N-Butyl-4-(methoxycarbonyl)benzamide (2.00 g, 8.70 mmol) was dissolved in MeOH/H_2O (3:1 v/v, 40 mL) in a 100 mL round-bottom flask equipped with a magnetic stir bar. K_2CO_3 (3.60 g, 3.00 equiv) was added, and the reaction mixture was stirred at 50 °C for 4 h. The solvent was removed under reduced pressure, and the residue was diluted with water and acidified to pH ~2 with 1 M HCl. The precipitate was collected by filtration, dissolved in 0.5 M aqueous NaOH (30 mL), and extracted with EtOAc twice. The aqueous phase was acidified to pH ~2 with 1 M HCl, and the resulting precipitate was collected by filtration, washed with water, and dried under vacuum to afford *N*-butyl-4-carboxybenzamide as a colorless solid (1.80 g, 96%).

To stirred solution of the above product (442.0 mg, 2 mmol) in DCM (6 mL) under nitrogen atmosphere was added TFEDMA (1.2 equiv) at room temperature. The reaction mixture was stirred for 3 hours at the same temperature, after the reaction completed, quench it with sat. aq. $NaHCO_3$, extract with DCM for 3 times. The combined organic phases were washed with brine and dried over Na_2SO_4. After filtration, the filtrate was concentrated under reduced pressure and the residue was purified by column chromatography on silica gel (EtOAc/hexane 1/3) to afford the title product as a white solid. (357.6 mg, 80%).

m.p. = 103.4 – 104.3℃

^{1}H NMR (500 MHz, $CDCl_3$) δ 8.10 (dd, *J* = 8.5, 1.9 Hz, 2H), 7.93 – 7.87 (m, 2H), 6.25 (s, 1H), 3.48 (q, *J* = 6.4, 5.7 Hz, 2H), 1.63 (p, *J* = 7.2 Hz, 2H), 1.43 (h, *J* = 7.3 Hz, 2H), 0.97 (t, *J* = 7.4 Hz, 3H).

^{13}C NMR (176 MHz, $CDCl_3$) δ 166.1, 156.7, 141.1, 131.8, 127.7, 127.4, 40.2, 31.7, 20.3, 13.9.

^{19}F NMR (658 MHz, $CDCl_3$) δ 19.8 (s, 1F).

HRMS (ESI) [$C_{13}H_{17}NO_3Na$] $[M+OMe+Na]^+$ for corresponding methyl ester, calculated for: 258.1100, found: 258.1109.

4-((*tert*-butyldimethylsilyl)oxy)benzoyl fluoride (**1za**)

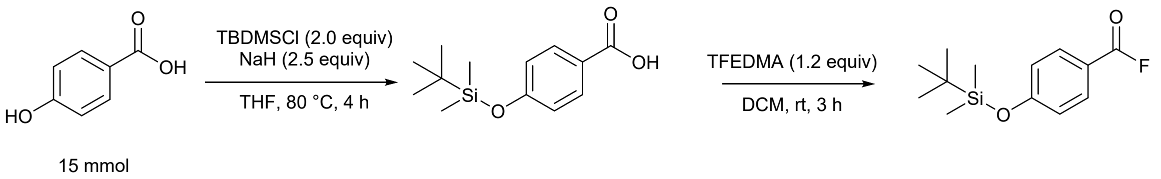

To a stirred suspension of NaH (1.5 g, 37.5 mmol) in THF under nitrogen atmosphere at 0 °C. 4-hydroxybenzoic acid (2.0 g, 15 mmol) was added portion wise. The resulting mixture allowed to warm up to room temperature and stirred for 30 min. TBDMSCl (4.5 g, 30 mmol) was added. The mixture was further stirred at 80 °C for 4 hours. After the reaction was completed, the solvent was removed by evaporation, the resulting mixture was diluted with EtOAc, subsequently, acidified by 1M HCl solution, The organic phases were washed with brine and dried over Na_2SO_4. After

filtration, the filtrate was concentrated under reduced pressure, and the residue was purified by recrystallization upon ether/hexane to afford the silylated product (1.69 g, 45%).
To stirred solution of above silylated compound (504 mg, 2 mmol) in dichloromethane (DCM) under nitrogen atmosphere was added TFEDMA (1.2 equiv) at room temperature. The reaction mixture was stirred for 3 hours at the same temperature, after the reaction completed, quench it with sat. aq. $NaHCO_3$, extract with DCM for 3 times. The combined organic phases were washed with brine and dried over Na_2SO_4. After filtration, the filtrate was concentrated under reduced pressure, and the residue was purified by column chromatography(EtOAc/*n*-hexane 1/50) on silica gel to afford the corresponding acyl fluoride product as a colorless oil (478.1 mg, 94%).

^{1}H NMR (700 MHz, $CDCl_3$) δ 7.94 (d, *J* = 8.7 Hz, 2H), 6.92 (dd, *J* = 8.8, 1.1 Hz, 2H), 0.99 (s, 9H), 0.26 (s, 6H).
^{13}C NMR (176 MHz, $CDCl_3$) δ 162.4, 157.4, 133.9, 120.7, 117.6, 25.7, 18.4, -4.2.
^{19}F NMR (658 MHz, $CDCl_3$) δ 16.3 (s, 1F).
HRMS (ESI) [$C_{14}H_{23}O_3Si$] $[M+OMe+H]^+$ for corresponding methyl ester, calculated for: 267.1411, found: 267.1416.

3.2 General procedures for synthesis of vinyl arenes

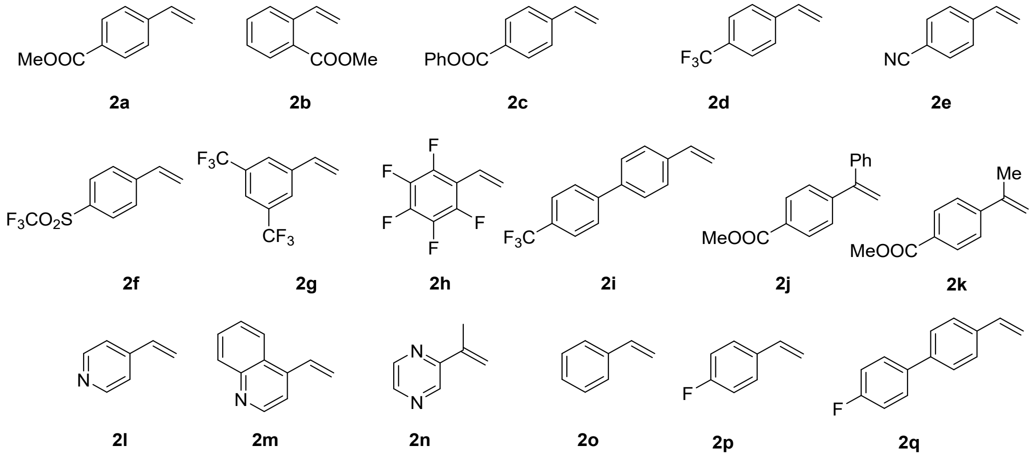

Vinyl arenes **2h**, **2l**, **2o, 2p** were purchased from TCI, **2a**, **2b**, **2d**, **2g**, **2k**, **2m**, **2n** compounds were prepared using experimental procedures described below.

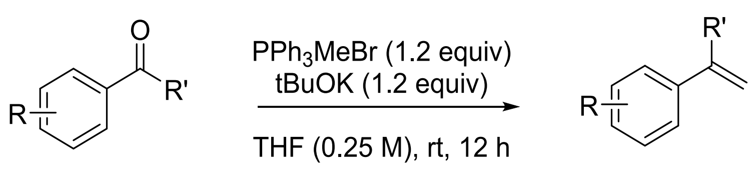

To an oven-dried flask was charged with PPh_3MeBr (1.2 equiv.) and THF under nitrogen atmosphere. Cool the suspension to 0 °C. tBuOK (1.2 equiv.) was added portion wise. The resulting mixture allowed to warm up to room temperature and stirred for 30 min. The yellow suspension was cooled to 0 °C again followed by drop-wise addition of the corresponding

aldehydes/ketones in THF (1.0 M). Subsequently, the mixture was further stirred at room temperature for 12 hours. After the reaction was completed, the solvent was removed by evaporation, the resulting mixture was diluted with water (30 mL) and extracted with Et_2O for 3 times, The combined organic phases were washed with brine and dried over Na_2SO_4. After filtration, the filtrate was concentrated under reduced pressure and the residue was purified by column chromatography on silica gel to afford the corresponding vinyl arene products.

methyl 4-vinylbenzoate (**2a**)

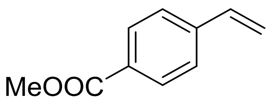

Purified by column chromatography on silica gel (*n*-hexane/EtOAc 15/1) to afford **2a** as a white solid (1 g, 69% yield) on a 9 mmol scale.
^{1}H NMR (300 MHz, $CDCl_3$) δ 8.00 (d, *J* = 7.8 Hz, 2H), 7.47 (d, *J* = 6.6 Hz, 2H), 6.76 (dd, *J* = 17.5, 10.9 Hz, 1H), 5.87 (d, *J* = 17.6 Hz, 1H), 5.39 (d, *J* = 10.9 Hz, 1H), 3.92 (s, 3H).
MS(EI): m/z 162 $[M]^+$.
The chemical shifts were consistent with reported literature.[7]

methyl 2-vinylbenzoate (**2b**)

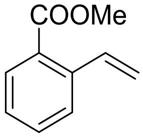

Purified by column chromatography on silica gel (*n*-hexane/EtOAc 15/1) to afford **2b** as a pale-yellow oil (102 mg, 25% yield) on a 2.5 mmol scale.
^{1}H NMR (300 MHz, $CDCl_3$) δ 7.88 (d, *J* = 7.5 Hz, 1H), 7.59 (d, *J* = 7.8 Hz, 1H), 7.53 – 7.38 (m, 2H), 7.32 (t, *J* = 7.5 Hz, 1H), 5.66 (d, *J* = 17.3 Hz, 1H), 5.36 (d, *J* = 10.7 Hz, 1H), 3.91 (s, 3H).
MS(EI): m/z 162 $[M]^+$.
The chemical shifts were consistent with reported literature.[8]

1-(trifluoromethyl)-4-vinylbenzene (**2d**)

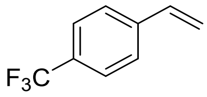

Purified by column chromatography on silica gel (*n*-pentane) to afford **2d** as a colorless oil (456 mg, 53% yield) on a 5 mmol scale.
^{1}H NMR (300 MHz, $CDCl_3$) δ 7.60 – 7.49 (m, 4H), 6.75 (dd, *J* = 17.6, 10.9 Hz, 1H), 5.85 (d, *J* = 17.6 Hz, 1H), 5.39 (d, *J* = 10.9 Hz, 1H). ^{19}F NMR (282 MHz, $CDCl_3$) δ -63.04 (1s, 3F).
MS(EI): m/z 172 $[M]^+$.
The chemical shifts were consistent with reported literature.[9]

1,3-bis(trifluoromethyl)-5-vinylbenzene (**2g**)

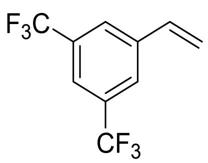

Purified by column chromatography on silica gel (*n*-hexane) to afford **2g** as a colorless oil (83 mg, 17% yield) on a 2 mmol scale.
^{1}H NMR (300 MHz, $CDCl_3$) δ 7.82 (s, 2H), 7.76 (s, 1H), 6.78 (dd, *J* = 18.2, 11.0 Hz, 1H), 6.01 – 5.84 (m, 1H), 5.50 (d, *J* = 11.0 Hz, 1H). ^{19}F NMR (282 MHz, $CDCl_3$) δ -63.52 (s, 6F).
MS(**EI**): m/z 240 $[M]^+$.
The chemical shifts were consistent with reported literature.[10]

methyl 4-(prop-1-en-2-yl)benzoate (**2k**)

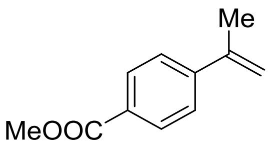

Purified by column chromatography on silica gel (*n*-hexane/EtOAc 15/1) to afford **2k** as a white solid (159 mg, 90% yield) on a 1 mmol scale.
^{1}H NMR (300 MHz, $CDCl_3$) δ 8.00 (d, *J* = 8.5 Hz, 2H), 7.52 (d, *J* = 8.4 Hz, 2H), 5.48 (s, 1H), 5.20 (s, 1H), 3.92 (s, 3H), 2.18 (s, 3H).
MS(**EI**): m/z 176 $[M]^+$.
The chemical shifts were consistent with reported literature.[11]

4-vinylquinoline (**2m**)

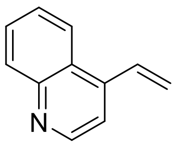

Purified by column chromatography on silica gel (*n*-hexane/EtOAc 10/1) to afford **2m** as a pale yellow oil (257.2 mg, 33% yield) on a 5.0 mmol scale.
^{1}H NMR (500 MHz, $CDCl_3$) δ 8.88 (d, *J* = 4.5 Hz, 1H), 8.13 – 8.10 (m, 2H), 7.72 (ddd, *J* = 8.4, 6.8, 1.5 Hz, 1H), 7.57 (ddd, *J* = 8.1, 6.8, 1.4 Hz, 1H), 7.48 (dd, *J* = 4.5, 0.7 Hz, 1H), 7.44 (dd, *J* = 17.4, 11.0 Hz, 1H), 5.99 (dd, *J* = 17.3, 1.1 Hz, 1H), 5.68 (dd, *J* = 11.0, 1.2 Hz, 1H).
MS(**EI**): m/z 155 $[M]^+$.
The chemical shifts were consistent with reported literature.[12]

2-(prop-1-en-2-yl)pyrazine (**2n**)

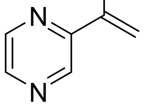

Purified by column chromatography on silica gel (*n*-hexane/EtOAc 10/1) to afford **2n** as a pale yellow oil (70.4 mg, 12% yield) on a 5.0 mmol scale.
^{1}H NMR (500 MHz, $CDCl_3$) δ 8.80 (d, J = 1.5 Hz, 1H), 8.53 (dd, J = 2.5, 1.5 Hz, 1H), 8.44 (d, J = 2.5 Hz, 1H), 5.94 (s, 1H), 5.43 (s, 1H), 2.24 (s, 3H).
MS(EI): m/z 120 $[M]^+$.
The chemical shifts were consistent with reported literature.[13]

phenyl 4-vinylbenzoate (**2c**)

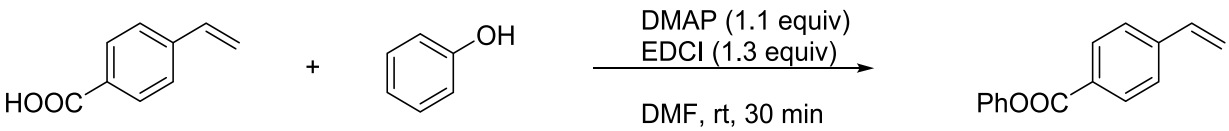

2c

To a stirred solution of 4-vinylbenzoic acid (250 mg, 2 mmol), phenol (207 mg, 2.2 mmol), and 4-(dimethylamino)pyridine (DMAP, 269 mg, 2.2 mmol) in DMF (2 mL) at 0 °C was added 1-ethyl-3-(3-dimethylaminopropyl)carbodiimide hydrochloride (EDCI, 404 mg, 2.6 mmol), and the mixture was stirred at room temperature for 30 min. After water was added, the mixture was extracted with diethyl ether, and the organic layer was washed with 1 M HCl, water, sat. Na_2CO_3 aq., and brine, and dried over anhydrous Na_2SO_4. The crude product was purified by column chromatography on silica gel (*n*-hexane/EtOAc 10/1) to afford **2c** as a white solid (347 mg, 77%).
^{1}H NMR (300 MHz, $CDCl_3$) δ 8.16 (d, *J* = 8.2 Hz, 2H), 7.53 – 7.40 (m, 4H), 7.29 – 7.20 (m, 3H), 6.89 – 6.61 (m, 1H), 5.90 (d, *J* = 17.6 Hz, 1H), 5.42 (d, *J* = 10.7 Hz, 1H).
MS(EI): m/z 224 $[M]^+$.
The chemical shifts were consistent with reported literature.[14]

4-vinylbenzonitrile (**2e**)

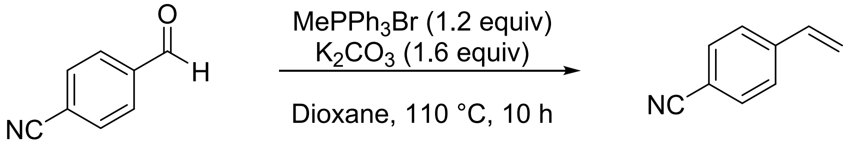

2e

4-Cyanobenzaldehyde (131 mg, 1 mmol) was added to a solution of methyl-triphenylphosphonium bromide (430 mg, 1.2 mmol) and K_2CO_3 (220 mg, 1.6 mmol) in dioxane (1 mL). The mixture was refluxed at 110 °C for 10 h. After being cooled to room temperature, the excess solvent was removed by rotary evaporation. The residue was diluted with hexane and filtered through celite and concentrated under vacuum. The crude product was purified by flash column chromatography on silica gel (*n*-hexane/EtOAc 15/1) to afford **2e** as a colorless oil (93 mg, 72% yield).
^{1}H NMR (300 MHz, $CDCl_3$) δ 7.62 (d, *J* = 8.2 Hz, 2H), 7.49 (d, *J* = 8.1 Hz, 2H), 6.73 (dd, *J* = 17.6, 10.9 Hz, 1H), 5.91 (s, 1H), 5.45 (d, *J* = 10.9 Hz, 1H).
MS(EI): m/z 129 $[M]^+$.
The chemical shifts were consistent with reported literature.[15]

1-((trifluoromethyl)sulfonyl)-4-vinylbenzene (**2f**)

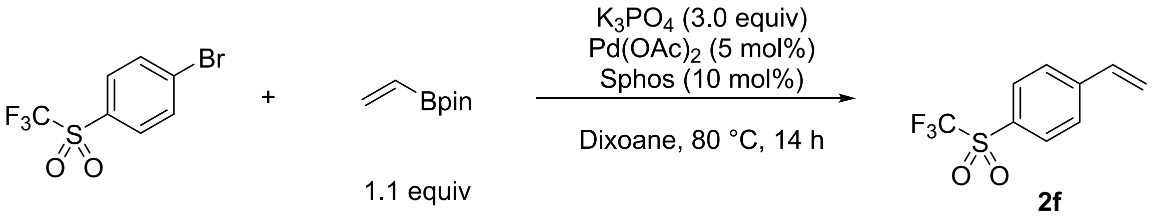

To a round bottom flask was charged with 1-bromo-4- (trifluoromethylsulfonyl)benzene (578 mg, 2.0 mmol), SPhos (82 mg, 0.2 mmol), K_3PO_4 (1.3 g, 6.0 mmol), 4,4,5,5-tetramethyl-2- vinyl-1,3,2-dioxaborolane (0.38 mL, 2.2 mmol), 1,4-dioxane (12 ml) and water (1.5 ml). The reaction vessel was sealed with a septum and purged with N_2 three times. $Pd(OAc)_2$ (23 mg, 0.1 mmol) was added and the reaction mixture was heated to 80 °C and stirred for 14 h. The reaction mixture was allowed to cool to ambient temperature and was subsequently diluted with ethyl acetate (10 ml). The mixture was filtered through a pad of silica. The organic phase was washed with brine (20 ml), dried over Na_2SO_4 and the solvent was removed under reduced pressure. The residue was purified by column chromatography (n-hexane/EtOAc 10/1) to afford 2f as a pale yellow solid (331 mg, 70% yield).

^{1}H NMR (500 MHz, $CDCl_3$) δ 7.99 (d, *J* = 8.5 Hz, 2H), 7.67 (d, *J* = 8.5 Hz, 2H), 6.81 (dd, *J* = 17.6, 10.9 Hz, 1H), 6.01 (d, *J* = 17.6 Hz, 1H), 5.58 (d, *J* = 10.9 Hz, 1H).

^{19}F NMR (41 MHz, $CDCl_3$) δ -78.89 (s, 3F).

MS(EI): m/z 236 $[M]^+$.

The chemical shifts were consistent with reported literature.[16]

4-(trifluoromethyl)-4'-vinyl-1,1'-biphenyl (**2i**)

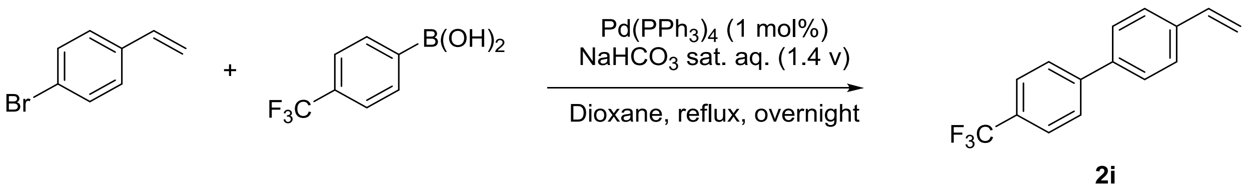

A dried flask was charged with 1-bromo-4-vinylbenzene (275 µL, 2.0 mmol), (4-(trifluoromethyl)phenyl)boronic acid (418 mg, 2.2 mmol), $Pd(PPh_3)_4$ (23 mg, 0.02 mmol), and sat. aq. $NaHCO_3$ (2.8 mL) in 1,4-Dioxane (8 mL). The mixture was stirred under reflux overnight, and the reaction progress was monitored by TLC. To this mixture was added water and extracted with EtOAc three times. The combined organic phases were washed with brine and dried over Na_2SO_4. After filtration, the filtrate was concentrated under reduced pressure. The residue was purified by column chromatography on silica gel (*n*-hexane) to afford **2i** as a white solid (393 mg, 79%).

^{1}H NMR (300 MHz, $CDCl_3$) δ 7.69 (s, 4H), 7.59 – 7.49 (m, 5H), 6.76 (dd, *J* = 17.4, 10.8 Hz, 1H), 5.82 (d, *J* = 17.5 Hz, 1H), 5.31 (d, *J* = 10.8 Hz, 1H).

^{19}F NMR (282 MHz, $CDCl_3$) δ -62.39 (s, 3F).

MS(EI): m/z 248 $[M]^+$.

The chemical shifts were consistent with reported literature.[17]

methyl 4-(1-phenylvinyl)benzoate (**2j**)

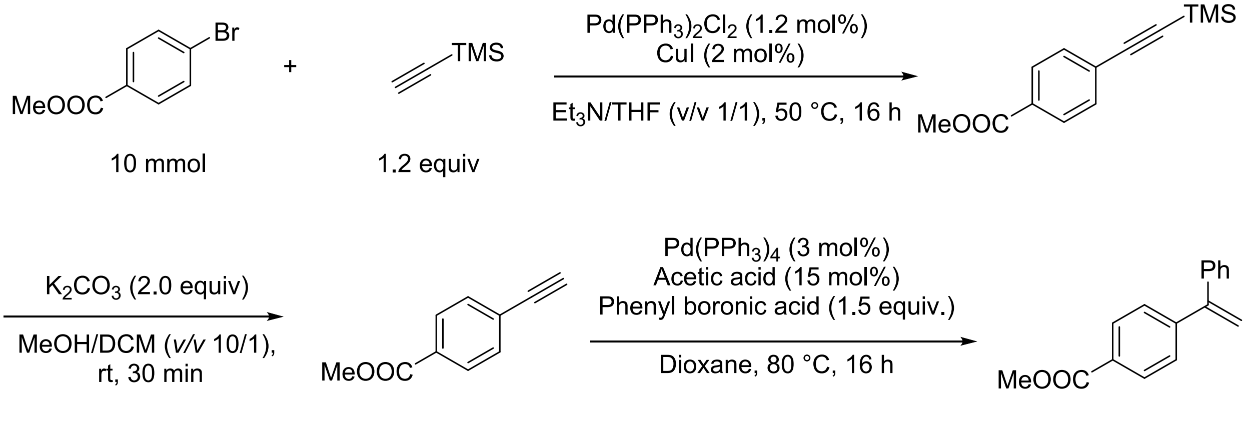

2j

To a round bottom flask was charged with methyl 4-bromobenzoate (2.15 g, 10 mmol), $PdCl_2(PPh_3)_2$ (84 mg, 0.12 mmol), and CuI (38 mg, 0.2 mmol) and purged with N_2 three times. Then the mixture of Et_3N (15 mL), THF (15 mL) and ethynyltrimethylsilane (1.7 mL, 12 mmol) was added dropwise. The reaction mixture was stirred at 50 °C for 16 h. The mixture was filtered, washed with brine, dried using anhydrous Na_2SO_4, and concentrated. The residue was purified by column chromatography on silica gel (*n*-hexane/EtOAc = 10/1) to give the product as a yellow solid (2.03 g, 87% yield).

The above product was dissolved in a mixed solution of MeOH/DCM (50 mL, 9/1, v/v). K_2CO_3 (2.4 g, 2.0 equiv.) was added. The mixture was stirred for 30 minutes before filtered through a pad of silica gel with MeOH to give the crude product methyl 4-ethynylbenzoate (1.4 g, >99% yield). The product was used for the next step without further purification.

To a round bottom flask was charged with methyl 4-ethynylbenzoate (160 mg, 1 mmol), $Pd(PPh_3)_4$ (34 mg, 0.03 mmol), and purged with N_2 three times. Dioxane (2.5 mL) was added followed by the addition of acetic acid (8.6 µL, 0.15 mmol). The mixture was stirred at 80 °C for 16 h. after the completion, cool the mixture to room temperature, and dilute the mixture with diethyl ether. Subsequently, the diluted reaction mixture was filtered through a pad of celite, the collected filtrate was concentrated under reduced pressure. The resulting residue was purified by column chromatography on silica gel (*n*-hexane/EtOAc 20/1) to afford the final product **2j** as a white solid (145 mg, 61% yield).

^{1}H NMR (300 MHz, $CDCl_3$) δ 8.01 (d, J = 8.0 Hz, 2H), 7.41 (d, J = 7.8 Hz, 2H), 7.33 (s, 5H), 5.55 (s, 2H), 3.93 (s, 3H).
MS(EI): m/z 238 $[M]^+$.
The chemical shifts were consistent with reported literature.[18]

4-(trifluoromethyl)-4'-vinyl-1,1'-biphenyl (**2q**)

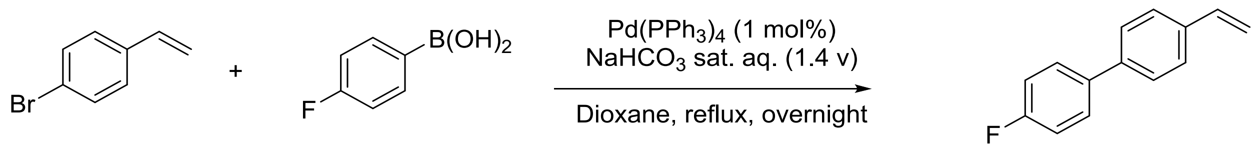

2 mmol

2q

A dried flask was charged with 1-bromo-4-vinylbenzene (275 μL, 2.0 mmol), 4-fluorophenyl boronic acid (418 mg, 2.2 mmol), $Pd(PPh_3)_4$ (23 mg, 0.02 mmol), and sat. aq. $NaHCO_3$ (2.8 mL) in 1,4-Dioxane (8 mL). The mixture was stirred under reflux overnight, and the reaction progress was monitored by TLC. To this mixture was added water and extracted with EtOAc three times. The combined organic phases were washed with brine and dried over Na_2SO_4. After filtration, the filtrate was concentrated under reduced pressure. The residue was purified by column chromatography on silica gel (*n*-hexane) to afford **2q** as a white solid (334 mg, 84%).
^1H NMR (700 MHz, $CDCl_3$) δ 7.57 – 7.53 (m, 2H), 7.52 – 7.46 (m, 4H), 7.12 (t, *J* = 8.7 Hz, 2H), 6.75 (dd, *J* = 17.6, 10.9 Hz, 1H), 5.79 (dd, *J* = 17.6, 0.8 Hz, 1H), 5.28 (dd, *J* = 10.8, 0.8 Hz, 1H).
^{19}F NMR (658 MHz, $CDCl_3$) δ -116.2 (s, 1F).
MS(EI): m/z 198 $[M]^+$.
The chemical shifts were consistent with reported literature.[17]

4. General synthetic procedure of silyl boronates

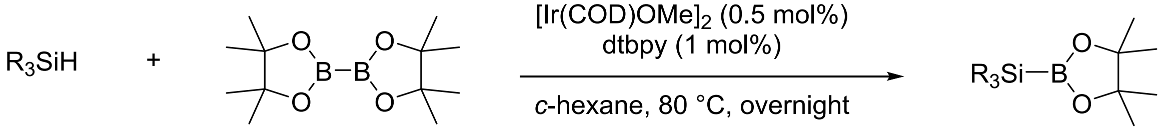

4.0 equiv

To an oven dried vial was charged with $[Ir(COD)OMe]_2$ (0.05 mol%), dtbpy (0.1 mol%), B_2pin_2, cyclohexane (1 M), and triethylsilane (4 equivlent) inside a nitrogen-filled glovebox. The resulting dark brown solution was heated at 80 °C overnight outside the glovebox. After being cooled to room temperature, the crude reaction mixture was concentrated in vacuo, and the residue was purified by flash column chromatography to afford the $R_3SiBpin$.

Following the above procedure, $Et_3SiBpin$ was synthesized on a 10 mmol scale, purified by flash column chromatography (DCM/*n*-hexane: 1/9) as a colorless oil (1.6 g, 66% yield).
^1H NMR (500 MHz, $CDCl_3$) δ 1.23 (s, 12H), 0.97 (t, *J* = 7.92 Hz, 9H), 0.59 (q, *J* = 7.92 Hz, 6H).
MS(EI): m/z 226 $[M-CH_3]^+$.
The chemical shifts were consistent with reported literature.[19]

Following the above method, *n*$Pr_3SiBpin$ was synthesized on a 2 mmol scale, purified by flash column chromatography (DCM/*n*-hexane: 1/9) as a colorless oil (312.8 mg, 55% yield).
^1H NMR (500 MHz, $CDCl_3$) δ 1.40 – 1.33 (m, 6H), 1.22 (s, 12H), 0.95 (t, *J* = 7.25 Hz, 9H), 0.62 – 0.58 (m, 6H).
MS(EI): m/z 268 $[M-CH_3]^+$.

The chemical shifts were consistent with reported literature.[19]

5. General procedure

5.1 General procedure **A** for reaction conditions optimalization

In a nitrogen filled glovebox, to a flame-dried screw-capped test tube was added catalyst (5 mol%), Ligand (6 mol%), solvent (0.5 M), benzoyl fluoride (0.1 mmol), methyl 4-vinylbenzoate, $PhMe_2SiBpin$ sequentially. The tube then was sealed and moved out from the glovebox. The solution was stirred at given temperature for given hours. The reaction tube was filtered through a pad of silica, concentrated under vacuum, followed by adding trimethyl orthoformate (11.0 μL, 0.1 mmol) as an internal standard for NMR analysis, the mixture was then concentrated again to give the residue, which was purified by column chromatography on silica gel (*n*-hexane/EtOAc) to give the corresponding product **3aa**.

5.2 General procedure **B** for substrate scope

In a nitrogen filled glovebox, to a flame-dried screw-capped test tube was added CuOAc (5 mol%), PCy_3 (6 mol%), DMF (0.5 M), acyl fluoride (0.2 mmol), vinyl arene (1.3 equiv.), $PhMe_2SiBpin$ (1.3 equiv.) sequentially. The tube then was sealed and moved out from the glovebox. The solution was stirred at 40 °C for 24 hours. The reaction tube was diluted with Et_2O and water, then extracted with Et_2O for 3 times, The combined organic phases were washed with brine and dried over Na_2SO_4. After filtration, the filtrate was concentrated under reduced pressure and the residue was purified by column chromatography on silica gel to afford the corresponding product.

5.3 General procedure **C** for larger scale reaction

In a nitrogen filled glovebox, to a flame-dried round bottom flask was added CuOAc (5 mol%), PCy_3 (6 mol%), DMF (0.5 M), benzoyl fluoride (2 mmol), methyl 4-vinylbenzoate (1.3 equiv.), $PhMe_2SiBpin$ (1.3 equiv.) sequentially. The flask then was sealed and moved out from the glovebox. The solution was stirred at 40 °C for 24 hours. The reaction tube was diluted with Et_2O and water, then extracted with Et_2O for 3 times, The combined organic phases were washed with brine and dried over Na_2SO_4. After filtration, the filtrate was concentrated under reduced pressure and the residue was purified by column chromatography on silica gel to afford the corresponding product **3aa**.

6. Characterization data for products

methyl 2-(3-(dimethyl(phenyl)silyl)-1-oxo-1-phenylpropan-2-yl)benzoate (3ab)

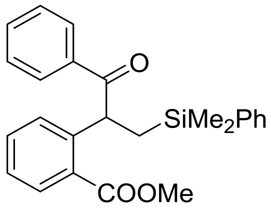

Following the general procedure **B**, the title compound was purified by flash column chromatography (EtOAc/*n*-hexane: 1/12) to afford **3ab** as colorless oil. (70.2 mg, 87% yield).
^{1}H NMR (500 MHz, CDCl$_3$) δ 7.88 (d, *J* = 7.1 Hz, 2H), 7.82 (d, *J* = 7.9 Hz, 1H), 7.47 – 7.41 (m, 3H), 7.37 – 7.27 (m, 7H), 7.23 – 7.18 (m, 1H), 5.86 (dd, *J* = 8.2, 6.3 Hz, 1H), 3.79 (s, 3H), 1.76 (dd, *J* = 14.8, 8.2 Hz, 1H), 1.26 (dd, *J* = 14.8, 6.4 Hz, 1H), 0.21 (s, 3H), 0.18 (s, 3H).
^{13}C NMR (126 MHz, CDCl$_3$) δ 200.9, 168.3, 143.0, 138.9, 136.7, 133.8, 132.8, 132.4, 131.0, 129.2, 129.0, 128.8, 128.6, 127.8, 126.6, 52.3, 44.3, 22.0, -2.3, -2.5.
HRMS (ESI) [$C_{25}H_{26}O_3NaSi$] [M+Na]$^+$ calculated for: 425.1543, found: 425.1547.

phenyl 4-(3-(dimethyl(phenyl)silyl)-1-oxo-1-phenylpropan-2-yl)benzoate (3ac)

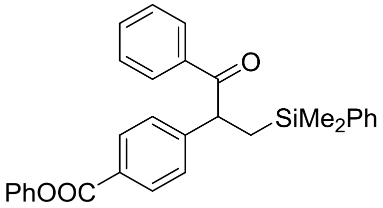

Following the general procedure **B**, the title compound was purified by flash column chromatography (EtOAc/*n*-hexane: 1/12) to afford **3ac** as white solid. (75.7 mg, 81% yield).
m.p. = 99.6 – 100.4 °C.
^{1}H NMR (500 MHz, CDCl$_3$) δ 8.06 (d, *J* = 8.3 Hz, 2H), 7.81 (d, *J* = 7.1 Hz, 2H), 7.49 – 7.43 (m, 3H), 7.43 – 7.38 (m, 2H), 7.38 – 7.31 (m, 7H), 7.27 – 7.22 (m, 1H), 7.19 – 7.15 (m, 2H), 4.67 (t, *J* = 7.4 Hz, 1H), 1.82 (dd, *J* = 14.9, 7.4 Hz, 1H), 1.43 (dd, *J* = 14.9, 7.5 Hz, 1H), 0.19 (s, 3H), 0.15 (s, 3H).
^{13}C NMR (126 MHz, CDCl$_3$) δ 199.6, 165.0, 151.0, 147.4, 138.2, 136.3, 133.8, 133.1, 130.8, 129.6, 129.3, 128.7, 128.6, 128.3, 128.0, 126.0, 121.8, 49.4, 21.4, -2.3, -2.4.
HRMS (ESI) [$C_{30}H_{28}O_3NaSi$] [M+Na]$^+$ calculated for: 487.1699, found: 487.1710.

3-(dimethyl(phenyl)silyl)-1-phenyl-2-(4-(trifluoromethyl)phenyl)propan-1-one (3ad)

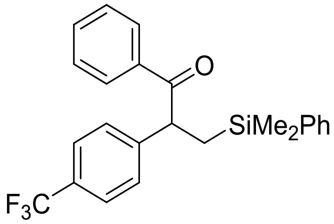

Following the general procedure **B**, the title compound was purified by flash column chromatography (EtOAc/*n*-hexane: 1/10) to afford **3ad** as colorless oil. (64.5 mg, 78% yield).
^{1}H NMR (300 MHz, CDCl$_3$) δ 7.79 (d, *J* = 8.5 Hz, 2H), 7.46 (d, *J* = 7.5 Hz, 3H), 7.43 – 7.28 (m, 9H), 4.64 (t, *J* = 7.4 Hz, 1H), 1.78 (dd, *J* = 14.8, 7.3 Hz, 1H), 1.39 (dd, *J* = 14.9, 7.6 Hz, 1H), 0.18 (s, 3H), 0.13 (s, 3H).
^{13}C NMR (176 MHz, CDCl$_3$) δ 199.8, 145.3, 138.2, 136.3, 133.7, 133.2, 129.3, 129.3 (q), 128.8, 128.7, 128.7, 128.0, 125.8 (q), 124.2 (q),49.0, 21.6, -2.4, -2.5.
^{19}F NMR (282 MHz, CDCl$_3$) δ -63.05 (s, 3F).
MS(EI): m/z 413 [M]$^+$.
The chemical shifts were consistent with reported literature.[20]

4-(3-(dimethyl(phenyl)silyl)-1-oxo-1-phenylpropan-2-yl)benzonitrile (3ae)

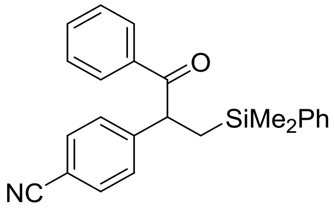

Following the general procedure **B**, the title compound was purified by flash column chromatography (EtOAc/*n*-hexane: 1/10) to afford **3ae** as colorless oil. (60.2 mg, 81% yield).
^{1}H NMR (300 MHz, CDCl$_3$) δ 7.77 (d, *J* = 7.4 Hz, 2H), 7.55 – 7.45 (m, 3H), 7.44 – 7.27 (m, 9H), 4.62 (t, *J* = 7.5 Hz, 1H), 1.77 (dd, *J* = 14.8, 7.2 Hz, 1H), 1.38 (dd, *J* = 14.8, 7.7 Hz, 1H), 0.19 (s, 3H), 0.12 (s, 3H).
^{13}C NMR (176 MHz, CDCl$_3$) δ 199.3, 146.6, 137.9, 136.1, 133.7, 133.4, 132.6, 129.4, 129.2, 128.8, 128.6, 128.0, 118.8, 111.0, 49.1, 21.6, -2.4, -2.5.
MS(EI): m/z 370 $[M]^+$
The chemical shifts were consistent with reported literature.[20]

3-(dimethyl(phenyl)silyl)-1-phenyl-2-(4-((trifluoromethyl)sulfonyl)phenyl)propan-1-one (3af)

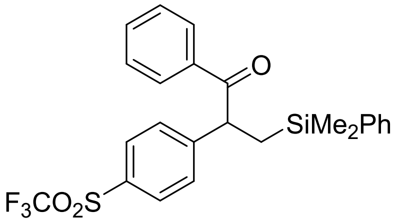

Following the general procedure B, the title compound was purified by flash column chromatography (EtOAc/*n*-hexane: 1/15) to afford **3af** as colorless oil. (57.8 mg, 61% yield).
^{1}H NMR (500 MHz, CDCl$_3$) δ 7.87 (d, *J* = 8.1 Hz, 2H), 7.82 – 7.75 (m, 2H), 7.56 – 7.48 (m, 3H), 7.40 (dd, *J* = 8.0, 6.6 Hz, 4H), 7.36 (d, *J* = 7.2 Hz, 1H), 7.32 (t, *J* = 6.9 Hz, 2H), 4.71 (t, *J* = 7.5 Hz, 1H), 1.81 (dd, *J* = 14.9, 7.3 Hz, 1H), 1.41 (dd, *J* = 15.0, 7.6 Hz, 1H), 0.21 (s, 3H), 0.13 (s, 3H).
^{13}C NMR (126 MHz, CDCl$_3$) δ 199.0, 150.9, 137.6, 135.9, 133.7, 133.6, 131.2, 130.0, 129.7, 129.5, 128.9, 128.7, 128.1, 119.9, 49.0, 22.1, -2.5, -2.6.
^{19}F NMR (282 MHz, CDCl$_3$) δ -78.9 (s, 3F).
HRMS (ESI) $[C_{24}H_{23}O_3NaSiSF_3]$ $[M+Na]^+$ calculated for: 499.0981, found: 499.0986.

2-(3,5-bis(trifluoromethyl)phenyl)-3-(dimethyl(phenyl)silyl)-1-phenylpropan-1-one (3ag)

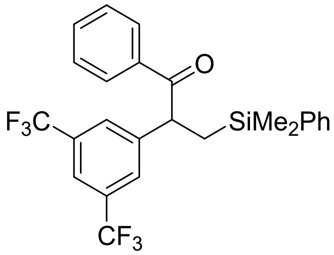

Following the general procedure **B**, the title compound was purified by flash column chromatography (EtOAc/Hexane: 1/15) to afford **3ag** as colorless oil. (76.4 mg, 79% yield).
^{1}H NMR (500 MHz, CDCl$_3$) δ 7.80 (d, *J* = 7.0 Hz, 2H), 7.65 (d, *J* = 3.7 Hz, 3H), 7.53 (t, *J* = 7.4 Hz, 1H), 7.44 – 7.38 (m, 2H), 7.38 – 7.33 (m, 3H), 7.33 – 7.28 (m, 2H), 4.68 (dd, *J* = 8.6, 6.6 Hz, 1H), 1.74 (dd, *J* = 15.0, 6.6 Hz, 1H), 1.44 (dd, *J* = 14.9, 8.7 Hz, 1H), 0.23 (s, 3H), 0.09 (s, 3H).
^{13}C NMR (126 MHz, CDCl$_3$) δ 199.3, 143.3, 137.4, 135.7, 133.7, 133.6, 132.2, 132.0, 131.7, 131.4, 129.5, 129.0, 128.9, 128.7, 128.1, 126.6, 124.4, 122.2, 121.2, 120.1, 48.4, 22.2, -2.6.
^{19}F NMR (282 MHz, CDCl$_3$) δ -63.32 (s, 6F).
HRMS (ESI) [$C_{25}H_{22}OF_6NaSi$] $[M+Na]^+$ calculated for: 503.1236, found: 503.1243.

3-(dimethyl(phenyl)silyl)-2-(perfluorophenyl)-1-phenylpropan-1-one (3ah)

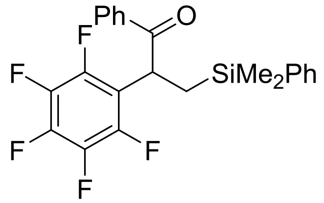

Following the general procedure B, the title compound was purified by flash column chromatography (EtOAc/*n*-hexane: 1/15) to afford **3ai** as white solid. (53.8 mg, 62% yield).
m.p. = 115.9 – 116.6 ℃.
^{1}H NMR (500 MHz, CDCl$_3$) δ 7.75 (dd, *J* = 8.4, 1.3 Hz, 2H), 7.52 – 7.49 (m, 1H), 7.40 – 7.37 (m, 2H), 7.34 (dd, *J* = 7.8, 1.7 Hz, 2H), 7.30 – 7.25 (m, 3H), 4.79 (dd, *J* = 10.0, 5.6 Hz, 1H), 1.65 – 1.60 (m, 2H), 0.37 (s, 3H), 0.24 (s, 3H).
^{13}C NMR (126 MHz, CDCl$_3$) δ 197.0, 144.9 (dt, *J* = 246.9, 10.4 Hz), 141.7 – 138.8 (m), 137.5 (dt, *J* = 252.9, 16.0 Hz), 137.4, 135.6, 133.3, 133.3, 129.2, 128.9, 128.3, 127.8, 114.6 – 113.8 (m), 39.4, 17.1, -2.1, -4.0.
^{19}F NMR (282 MHz, CDCl$_3$) δ -141.1 (d, *J* = 15.8 Hz), -156.3 (t, *J* = 21.1 Hz), -162.3 – -162.5 (m).
HRMS (ESI) [$C_{23}H_{19}ONaSi$] $[M+Na]^+$ calculated for: 457.1017, found: 457.1026.

3-(dimethyl(phenyl)silyl)-1-phenyl-2-(4'-(trifluoromethyl)-[1,1'-biphenyl]-4-yl)propan-1-one (3ai)

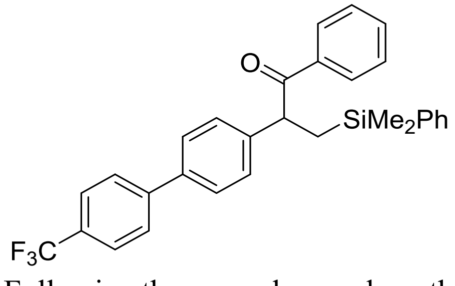

Following the general procedure, the title compound was purified by flash column chromatography (EtOAc/*n*-hexane: 1/10) to afford **3ah** as colorless oil. (35.5 mg, 36% yield).
^{1}H NMR (500 MHz, CDCl$_3$) δ 7.86 (d, *J* = 7.1 Hz, 2H), 7.68 – 7.58 (m, 4H), 7.50 – 7.42 (m, 5H), 7.40 – 7.29 (m, 7H), 4.66 (t, *J* = 7.4 Hz, 1H), 1.81 (dd, *J* = 14.9, 7.4 Hz, 1H), 1.45 (dd, *J* = 14.9, 7.4 Hz, 1H), 0.19 (s, 3H), 0.15 (s, 3H).
^{13}C NMR (126 MHz, CDCl$_3$) δ 200.1, 144.2, 141.4, 138.4, 138.3, 136.4, 133.7, 132.9, 129.3, 129.0, 128.9, 128.7, 128.6, 127.8, 127.6, 127.3, 125.7, 124.3, 48.8, 21.4, -2.5.

HRMS (ESI) [$C_{30}H_{27}ONaSiF_3$] $[M+Na]^+$ calculated for: 511.1675, found: 511.1684.

methyl 4-(3-(dimethyl(phenyl)silyl)-1-oxo-1,2-diphenylpropan-2-yl)benzoate (3aj)

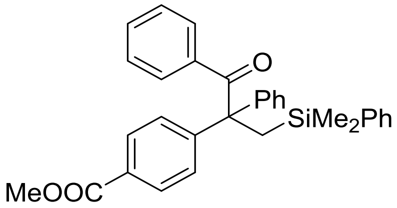

Following the general procedure **B**, the title compound was purified by flash column chromatography (EtOAc/Hexane: 1/10) to afford **3aj** as colorless oil. (55.4 mg, 58% yield).

^{1}H NMR (500 MHz, CDCl$_3$) δ 7.87 (d, J = 8.6 Hz, 2H), 7.52 (d, J = 7.1 Hz, 2H), 7.37 (d, J = 8.6 Hz, 2H), 7.33 – 7.20 (m, 12H), 7.18 – 7.13 (m, 2H), 3.89 (s, 3H), 2.07 (s, 2H), -0.01 (s, 3H), -0.07 (s, 3H).

^{13}C NMR (126 MHz, CDCl$_3$) δ 201.3, 166.9, 149.4, 143.0, 140.7, 137.1, 133.6, 131.9, 130.4, 129.4, 129.3, 129.2, 128.6, 128.5, 127.9, 127.6, 127.2, 64.1, 52.2, 33.3, -1.2, -1.3.

HRMS (ESI) [$C_{31}H_{30}O_3NaSi$] $[M+Na]^+$ calculated for: 501.1856, found: 501.1863.

methyl 4-(3-(dimethyl(phenyl)silyl)-2-methyl-1-oxo-1-phenylpropan-2-yl)benzoate (3ak)

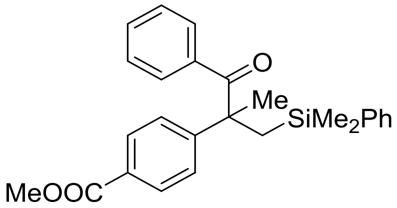

Following the general procedure **B**, the title compound was purified by flash column chromatography (EtOAc/Hexane: 1/12) to afford **3ak** as colorless oil. (27.0 mg, 32% yield).

^{1}H NMR (500 MHz, CDCl$_3$) δ 7.96 (d, J = 8.5 Hz, 2H), 7.40 – 7.25 (m, 11H), 7.20 – 7.15 (m, 2H), 3.91 (s, 3H), 1.82 – 1.68 (m, 2H), 1.57 (s, 3H), 0.18 (s, 3H), 0.08 (s, 3H).

^{13}C NMR (126 MHz, CDCl$_3$) δ 203.3, 167.0, 151.3, 139.6, 136.3, 133.7, 131.8, 130.3, 129.7, 128.9, 128.8, 128.1, 127.8, 126.2, 54.2, 52.3, 29.7, 26.7, -1.1, -1.4.

HRMS (ESI) [$C_{26}H_{28}O_3NaSi$] $[M+Na]^+$ calculated for: 439.1669, found: 439.1701.

3-(dimethyl(phenyl)silyl)-1-phenyl-2-(pyridin-2-yl)propan-1-one (3al)

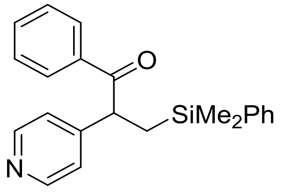

Following the general procedure **B**, the title compound was purified by flash column chromatography (EtOAc/Hexane: 1/3) to afford **3al** as pale yellow oil (15.5 mg, 22% yield).

^{1}H NMR (500 MHz, CDCl$_3$) δ 8.48 (d, J = 5.2 Hz, 2H), 7.77 (dd, J = 8.4, 1.3 Hz, 2H), 7.52 – 7.49 (m, 1H), 7.43 (dd, J = 7.8, 1.7 Hz, 2H), 7.39 – 7.31 (m, 5H), 7.19 – 7.18 (m, 2H), 4.56 (t, J = 7.4 Hz, 1H), 1.80 (dd, J = 14.9, 7.7 Hz, 1H), 1.35 (dd, J = 14.9, 7.1 Hz, 1H), 0.20 (s, 3H), 0.16 (s, 3H).

^{13}C NMR (126 MHz, CDCl$_3$) δ 199.1, 151.0, 149.6, 137.9, 136.0, 133.7, 133.4, 129.4, 128.8, 128.7, 128.1, 123.8, 48.6, 21.3, -2.4, -2.4.

HRMS (ESI) [$C_{22}H_{23}ONaSi$] $[M+Na]^+$ calculated for: 368.1441, found: 368.1448.

3-(dimethyl(phenyl)silyl)-1-phenyl-2-(quinolin-4-yl)propan-1-one (3am)

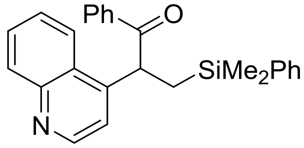

Following the general procedure **B**, the title compound was purified by flash column chromatography (EtOAc/*n*-hexane: 1/9) to afford **3am** as colorless oil. (25.1 mg, 32% yield).
^{1}H NMR (500 MHz, $CDCl_3$) δ 8.72 (d, *J* = 4.6 Hz, 1H), 8.11 (dd, *J* = 8.9, 1.3 Hz, 1H), 7.72 – 7.70 (m, 4H), 7.50 – 7.44 (m, 3H), 7.44 – 7.39 (m, 2H), 7.36 – 7.34 (m, 2H), 7.30 – 7.26 (m, 2H), 7.13 (d, *J* = 4.6 Hz, 1H), 5.25 (dd, *J* = 9.6, 4.4 Hz, 1H), 2.00 (dd, *J* = 15.0, 9.6 Hz, 1H), 1.27 (dd, *J* = 14.9, 4.3 Hz, 1H), 0.29 (s, 3H), 0.21 (s, 3H).
^{13}C NMR (126 MHz, $CDCl_3$) δ 199.2, 150.4, 148.9, 148.2, 138.1, 136.3, 133.9, 133.3, 130.8, 129.5, 129.4, 128.8, 128.5, 128.1, 127.2, 125.9, 122.5, 119.7, 44.1, 21.3, -2.3, -2.8.
HRMS (ESI) [$C_{26}H_{25}NONaSi$] $[M+Na]^+$ calculated for: 418.1597, found: 418.1607.

3-(dimethyl(phenyl)silyl)-2-methyl-1-phenyl-2-(pyrazin-2-yl)propan-1-one (3an)

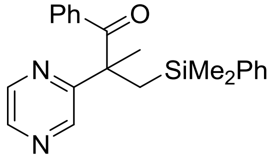

Following the general procedure, the title compound was purified by flash column chromatography (EtOAc/*n*-hexane: 1/9) to afford **3an** as colorless oil. (61.6 mg, 85% yield).
^{1}H NMR (500 MHz, $CDCl_3$) δ 8.5 (d, *J* = 1.6 Hz, 1H), 8.4 (dd, *J* = 2.6, 1.5 Hz, 1H), 8.3 (d, *J* = 2.5 Hz, 1H), 7.4 – 7.3 (m, 5H), 7.3 – 7.2 (m, 3H), 7.2 – 7.2 (m, 2H), 1.9 (s, 2H), 1.7 (s, 3H), 0.2 (s, 3H), 0.1 (s, 3H).
^{13}C NMR (126 MHz, $CDCl_3$) δ 201.6, 160.5, 143.7, 143.1, 142.6, 139.3, 135.6, 133.6, 132.0, 129.7, 128.9, 128.3, 127.8, 55.5, 28.6, 26.0, -1.2, -1.2.
HRMS (ESI) [$C_{22}H_{24}N_2ONaSi$] $[M+Na]^+$ calculated for: 383.1550, found: 383.1561.

methyl 4-(3-(dimethyl(phenyl)silyl)-1-oxo-1-phenylpropan-2-yl)benzoate (3aa)

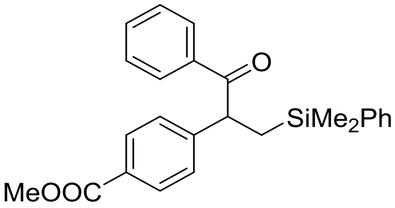

Following the general procedure **B**, the title compound was purified by flash column chromatography (EtOAc/Hexane: 1/15) to afford **3aa** as colorless oil. (79.1 mg, 98% yield).
Following the general procedure **C**, the title compound was purified by flash column chromatography (EtOAc/Hexane: 1/15) to afford **3aa** as colorless oil. (776.4 mg, 96% yield).

^{1}H NMR (300 MHz, CDCl$_3$) δ 7.90 (d, *J* = 8.4 Hz, 2H), 7.79 (d, *J* = 8.3 Hz, 2H), 7.50 – 7.40 (m, 3H), 7.38 – 7.31 (m, 5H), 7.30 – 7.24 (m, 3H), 4.62 (t, *J* = 7.5 Hz, 1H), 3.87 (s, 3H), 1.78 (dd, *J* = 14.8, 7.3 Hz, 1H), 1.40 (dd, *J* = 14.9, 7.6 Hz, 1H), 0.15 (s, 3H), 0.11 (s, 3H).
^{13}C NMR (176 MHz, CDCl$_3$) δ 199.8, 166.9, 146.6, 138.3, 136.4, 133.8, 133.1, 130.2, 129.2, 128.9, 128.7, 128.7, 128.4, 128.0, 52.2, 49.3, 21.4, -2.3, -2.5.
^{29}Si NMR (139 MHz, CDCl$_3$) δ -2.8.
MS(EI): m/z 402 $[M]^+$.
The chemical shifts were consistent with reported literature.[20]

methyl 4-(1-oxo-1-phenyl-3-(triethylsilyl)propan-2-yl)benzoate (3aa')

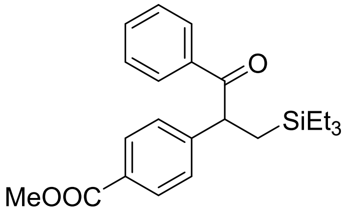

Following the general procedure **B**, the title compound was purified by flash column chromatography (EtOAc/Hexane: 1/12) to afford **3aa'** as colorless oil. (54.4 mg, 71% yield).

^{1}H NMR (500 MHz, CDCl$_3$) δ 7.96 – 7.93 (m, 4H), 7.50 – 7.47 (m, 1H), 7.42 – 7.38 (m, 4H), 4.74 (t, *J* = 7.38 Hz, 1H), 3.87 (s, 3H), 1.59 (dd, *J* = 14.90, 7.56 Hz, 1H), 1.16 (dd, *J* = 14.91, 7.19 Hz, 1H), 0.87 (t, *J* = 7.92 Hz, 9H), 0.48 – 0.35 (m, 6H).
^{13}C NMR (176 MHz, CDCl$_3$) δ 199.90, 166.98, 147.26, 136.58, 133.13, 130.29, 128.93, 128.78, 128.71, 128.32, 52.19, 49.24, 17.19, 7.45, 3.71.
^{29}Si NMR (139 MHz, CDCl$_3$) δ 7.1.
HRMS (ESI) [$C_{23}H_{30}O_3NaSi$] $[M+Na]^+$ calculated for: 405.1856, found: 405.1864.

methyl 4-(1-oxo-1-phenyl-3-(tripropylsilyl)propan-2-yl)benzoate (3aa'')

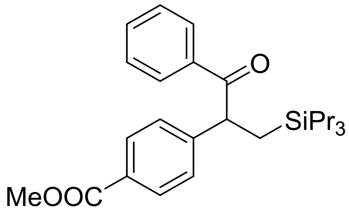

Following the general procedure **B**, the title compound was purified by flash column chromatography (EtOAc/Hexane: 1/19) to afford **3aa''** as colorless oil. (16.2 mg, 19% yield).
Following the general procedure **B,** the reaction was conducted at 80 ℃, the title compound was purified by flash column chromatography (EtOAc/Hexane: 1/19) to afford **3aa''** as colorless oil. (57.0 mg, 67% yield).
^{1}H NMR (500 MHz, CDCl$_3$) δ 7.95 – 7.92 (m, 4H), 7.51 – 7.48 (m, 1H), 7.42 – 7.39 (m, 4H), 4.73 (t, *J* = 7.32 Hz, 1H), 3.87 (s, 3H), 1.58 (dd, *J* = 14.90, 7.53 Hz, 1H), 1.30 – 1.20 (m, 6H), 1.14 (dd, *J* = 14.92, 7.14 Hz, 1H), 0.88 (t, *J* = 7.23 Hz, 9H), 0.46 – 0.33 (m, 6H).
^{13}C NMR (176 MHz, CDCl$_3$) δ 199.9, 167.0, 147.3, 136.6, 133.1, 130.3, 128.9, 128.8, 128.7, 128.3, 52.2, 49.3, 18.7, 18.2, 17.5, 15.6.

^{29}Si NMR (139 MHz, CDCl$_3$) δ 2.6.
HRMS (ESI) [$C_{26}H_{36}O_3NaSi$] [M+Na]$^+$ calculated for: 447.2326, found: 447.2337.

methyl 4-(3-(dimethyl(phenyl)silyl)-1-oxo-1-(p-tolyl)propan-2-yl)benzoate (3ba)

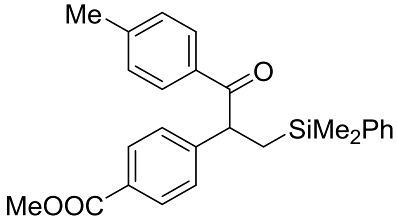

Following the general procedure **B**, the title compound was purified by flash column chromatography (EtOAc/Hexane: 1/15) to afford **3ba** as colorless oil. (71.0 mg, 85% yield).
^{1}H NMR (500 MHz, CDCl$_3$) δ 7.89 (d, *J* = 8.4 Hz, 2H), 7.71 (d, *J* = 8.3 Hz, 2H), 7.44 (d, *J* = 7.7 Hz, 2H), 7.34 – 7.28 (m, 3H), 7.28 (d, *J* = 8.4 Hz, 2H), 7.14 (d, *J* = 7.8 Hz, 2H), 4.62 (t, *J* = 7.4 Hz, 1H), 3.86 (s, 3H), 2.33 (s, 3H), 1.78 (dd, *J* = 14.9, 7.4 Hz, 1H), 1.40 (dd, *J* = 14.8, 7.5 Hz, 1H), 0.15 (s, 3H), 0.13 (s, 3H).
^{13}C NMR (126 MHz, CDCl$_3$) δ 199.3, 166.9, 146.8, 143.9, 138.4, 133.8, 130.2, 129.4, 129.2, 128.8, 128.4, 128.0, 52.2, 49.1, 21.7, 21.3, -2.3, -2.5.
HRMS (ESI) [$C_{26}H_{28}O_3NaSi$] [M+Na]$^+$ calculated for: 439.1706, found: 439.1705.

methyl 4-(3-(dimethyl(phenyl)silyl)-1-(4-methoxyphenyl)-1-oxopropan-2-yl)benzoate (3ca)

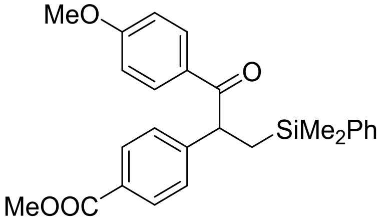

Following the general procedure **B**, the title compound was purified by flash column chromatography (EtOAc/Hexane: 1/7) to afford **3ca** as colorless oil. (69.2 mg, 80% yield).
^{1}H NMR (300 MHz, CDCl$_3$) δ 7.90 (d, *J* = 8.3 Hz, 2H), 7.80 (d, *J* = 8.9 Hz, 2H), 7.46 – 7.42 (m, 2H), 7.37 – 7.24 (m, 5H), 6.82 (d, *J* = 8.9 Hz, 2H), 4.59 (t, *J* = 7.4 Hz, 1H), 3.87 (s, 3H), 3.81 (s, 3H), 1.77 (dd, *J* = 14.8, 7.4 Hz, 1H), 1.39 (dd, *J* = 14.8, 7.5 Hz, 1H), 0.15 (s, 3H), 0.10 (s, 3H).
^{13}C NMR (176 MHz, CDCl$_3$) δ 198.2, 167.0, 163.5, 147.1, 138.4, 133.8, 131.0, 130.2, 129.3, 129.2, 128.8, 128.3, 128.0, 113.9, 55.5, 52.2, 48.9, 21.4, -2.3, -2.5.
MS(EI): m/z 433 [M]$^+$.
The chemical shifts were consistent with reported literature.[20]

methyl 4-(1-(4-cyclohexylphenyl)-3-(dimethyl(phenyl)silyl)-1-oxopropan-2-yl)benzoate (3da)

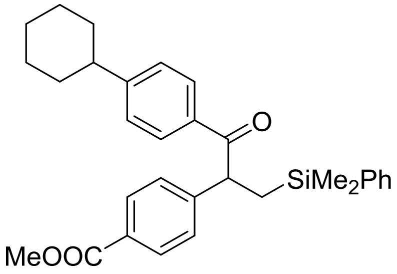

Following the general procedure **B**, the title compound was purified by flash column chromatography (EtOAc/Hexane: 1/15) to afford **3da** as colorless oil. (83.0 mg, 86% yield).
^{1}H NMR (500 MHz, CDCl$_3$) δ 7.90 (d, *J* = 8.3 Hz, 2H), 7.74 (d, *J* = 8.4 Hz, 2H), 7.43 (d, *J* = 7.7 Hz, 2H), 7.35 – 7.28 (m, 5H), 7.17 (d, *J* = 8.3 Hz, 2H), 4.62 (t, *J* = 7.4 Hz, 1H), 3.86 (s, 3H), 2.51 – 2.46 (m, 1H), 1.84 – 1.72 (m, 6H), 1.43 – 1.34 (m, 5H), 1.27 – 1.20 (m, 1H), 0.15 (s, 3H), 0.10 (s, 3H).
^{13}C NMR (126 MHz, CDCl$_3$) δ 199.3, 167.0, 153.8, 146.8, 138.4, 134.1, 133.8, 130.2, 129.2, 128.9, 128.8, 128.4, 128.0, 127.2, 52.2, 49.1, 44.7, 34.1, 26.8, 26.1, 21.4, -2.3, -2.5.
HRMS (ESI) [$C_{31}H_{36}O_3NaSi$] $[M+Na]^+$ calculated for: 507.2325, found: 507.2329.

methyl 4-(1-(3,5-di-tert-butylphenyl)-3-(dimethyl(phenyl)silyl)-1-oxopropan-2-yl)benzoate (3ea)

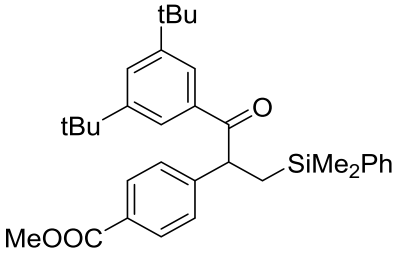

Following the general procedure **B**, the title compound was purified by flash column chromatography (EtOAc/Hexane: 1/15) to afford **3ea** as colorless oil. (90.3 mg, 88% yield).
^{1}H NMR (500 MHz, CDCl$_3$) δ 7.91 (d, *J* = 8.4 Hz, 2H), 7.69 (d, *J* = 1.8 Hz, 2H), 7.54 (t, *J* = 1.8 Hz, 1H), 7.45 – 7.43 (m, 2H), 7.35 – 7.29 (m, 5H), 4.69 – 4.63 (m, 1H), 3.87 (s, 3H), 1.76 (dd, *J* = 14.9, 6.9 Hz, 1H), 1.43 (dd, *J* = 14.9, 8.1 Hz, 1H), 1.27 (s, 18H), 0.16 (d, 3H), 0.13 (s, 3H).
^{13}C NMR (126 MHz, CDCl$_3$) δ 200.3, 167.0, 151.2, 146.9, 138.4, 135.8, 133.8, 130.1, 129.2, 128.8, 128.5, 128.0, 127.4, 123.1, 52.2, 49.4, 35.0, 31.4, 21.4, -2.3, -2.5.
HRMS (ESI) [$C_{33}H_{42}O_3NaSi$] $[M+Na]^+$ calculated for: 537.2795, found: 537.2803.

methyl 4-(3-(dimethyl(phenyl)silyl)-1-oxo-1-(4-(trifluoromethyl)phenyl)propan-2-yl)benzoate (3fa)

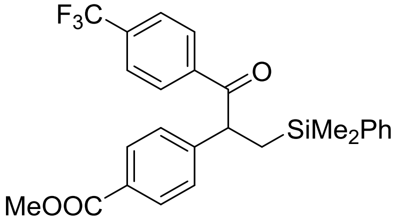

Following the general procedure **B**, the title compound was purified by flash column chromatography (EtOAc/Hexane: 1/15) to afford **3fa** as colorless oil. (80.2 mg, 85% yield).
^{1}H NMR (500 MHz, CDCl$_3$) δ 7.92 (d, *J* = 8.4 Hz, 2H), 7.83 (d, *J* = 8.2 Hz, 2H), 7.58 (d, *J* = 8.1 Hz, 2H), 7.43 (d, *J* = 7.8 Hz, 2H), 7.39 – 7.30 (m, 3H), 7.26 (d, *J* = 8.4 Hz, 2H), 4.58 (t, *J* = 7.4 Hz, 1H), 3.87 (s, 3H), 1.79 (dd, *J* = 14.9, 7.4 Hz, 1H), 1.40 (dd, *J* = 14.9, 7.4 Hz, 1H), 0.18 (s, 3H), 0.12 (s, 3H).
^{13}C NMR (126 MHz, CDCl$_3$) δ 198.9, 166.8, 145.8, 139.1, 138.0, 134.2, 133.8, 130.4, 129.4, 129.2, 128.9, 128.4, 128.1, 125.7, 123.6, 52.2, 49.9, 21.3, -2.3, -2.6.

^{19}F NMR (282 MHz, CDCl$_3$) δ -63.7 (s, 3F).
HRMS (ESI) [$C_{26}H_{25}O_3NaSiF_3$] [M+Na]$^+$ calculated for: 493.1417, found: 493.1418.

methyl 4-(3-(dimethyl(phenyl)silyl)-1-(4-fluorophenyl)-1-oxopropan-2-yl)benzoate (3ga)

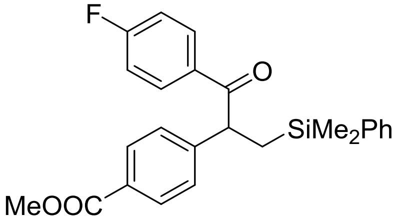

Following the general procedure **B**, the title compound was purified by flash column chromatography (EtOAc/Hexane: 1/15) to afford **3ga** as colorless oil. (75.0 mg, 89% yield).
^{1}H NMR (500 MHz, CDCl$_3$) δ 7.91 (d, *J* = 8.4 Hz, 2H), 7.80 (dd, *J* = 8.9, 5.4 Hz, 2H), 7.44 – 7.42 (m, 2H), 7.36 – 7.31 (m, 3H), 7.27 (d, *J* = 8.4 Hz, 2H), 7.03 – 6.97 (m, 2H), 4.56 (t, *J* = 7.4 Hz, 1H), 3.87 (s, 3H), 1.77 (dd, *J* = 14.9, 7.4 Hz, 1H), 1.39 (dd, *J* = 14.9, 7.4 Hz, 1H), 0.17 (s, 3H), 0.11 (s, 3H).
^{13}C NMR (126 MHz, CDCl$_3$) δ 198.2, 166.9, 165.6, 146.4, 138.1, 133.8, 132.7, 131.3, 130.3, 129.3, 129.0, 128.3, 128.0, 115.8, 52.2, 49.3, 21.4, -2.3, -2.6.
^{19}F NMR (282 MHz, CDCl$_3$) δ -105.7 – -105.6 (m, 1F).
HRMS (ESI) [$C_{25}H_{25}O_3NaSiF$] [M+Na]$^+$ calculated for: 443.1449, found: 443.1452.

methyl 4-(1-(4-chlorophenyl)-3-(dimethyl(phenyl)silyl)-1-oxopropan-2-yl)benzoate (3ha)

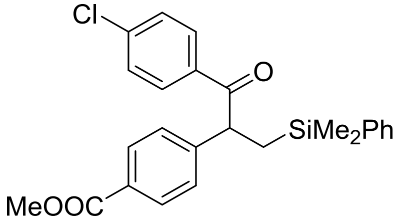

Following the general procedure **B**, the title compound was purified by flash column chromatography (EtOAc/Hexane: 1/15) to afford **3ha** as colorless oil. (87.2 mg, 99% yield).
^{1}H NMR (500 MHz, CDCl$_3$) δ 7.91 (d, *J* = 8.4 Hz, 2H), 7.71 (s, 2H), 7.43 (d, *J* = 7.8 Hz, 2H), 7.36 – 7.22 (m, 7H), 4.54 (t, *J* = 7.4 Hz, 1H), 3.87 (s, 3H), 1.77 (dd, *J* = 14.9, 7.4 Hz, 1H), 1.39 (dd, *J* = 14.9, 7.5 Hz, 1H), 0.17 (s, 3H), 0.11 (s, 3H).
^{13}C NMR (126 MHz, CDCl$_3$) δ 198.5, 166.8, 146.2, 139.5, 138.1, 134.6, 133.8, 130.3, 130.1, 129.3, 129.1, 129.0, 128.3, 128.0, 52.2, 49.4, 21.3, -2.3, -2.6.
HRMS (ESI) [$C_{25}H_{25}O_3NaSiCl$] [M+Na]$^+$ calculated for: 459.1153, found: 459.1159.

methyl 4-(1-(4-bromophenyl)-3-(dimethyl(phenyl)silyl)-1-oxopropan-2-yl)benzoate (3ia)

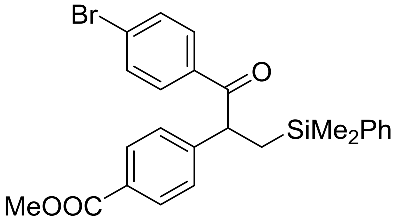

Following the general procedure **B**, the title compound was purified by flash column chromatography (EtOAc/Hexane: 1/15) to afford **3ia** as colorless oil. (92.1 mg, 96% yield).
^{1}H NMR (500 MHz, CDCl$_3$) δ 7.90 (d, *J* = 8.4 Hz, 2H), 7.62 (d, *J* = 8.6 Hz, 2H), 7.47 – 7.42 (m, 4H), 7.36 – 7.33 (m, 3H), 7.25 (d, *J* = 8.4 Hz, 2H), 4.53 (t, *J* = 7.4 Hz, 1H), 3.87 (s, 3H), 1.77 (dd, *J* = 14.9, 7.4 Hz, 1H), 1.38 (dd, *J* = 14.9, 7.4 Hz, 1H), 0.16 (s, 3H), 0.11 (s, 3H).
^{13}C NMR (126 MHz, CDCl$_3$) δ 198.7, 166.8, 146.2, 138.1, 135.0, 133.8, 131.9, 130.3, 130.2, 129.3, 129.1, 128.3, 128.2, 128.0, 52.2, 49.4, 21.3, -2.3, -2.6.
HRMS (ESI) [$C_{25}H_{25}O_3NaSiBr$] $[M+Na]^+$ calculated for: 503.0648, found: 503.0659.

methyl 4-(3-(dimethyl(phenyl)silyl)-1-oxo-1-(4-(trifluoromethoxy)phenyl)propan-2-yl)benzoate (3ja)

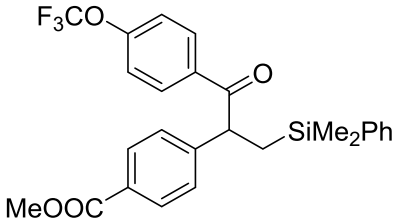

Following the general procedure **B**, the title compound was purified by flash column chromatography (EtOAc/Hexane: 1/15) to afford **3ja** as colorless oil. (79.8 mg, 82% yield).
^{1}H NMR (500 MHz, CDCl$_2$) δ 7.92 (d, *J* = 8.5 Hz, 2H), 7.81 (d, *J* = 8.9 Hz, 2H), 7.44 – 7.42 (m, 2H), 7.35 – 7.30 (m, 3H), 7.27 (d, *J* = 8.6 Hz, 2H), 7.15 (d, *J* = 7.9 Hz, 2H), 4.56 (t, *J* = 7.4 Hz, 1H), 3.88 (s, 3H), 1.78 (dd, *J* = 14.9, 7.5 Hz, 1H), 1.39 (dd, *J* = 14.9, 7.4 Hz, 1H), 0.17 (s, 3H), 0.12 (s, 3H).
^{13}C NMR (126 MHz, CDCl$_3$) δ 198.2, 166.8, 152.5, 146.2, 138.1, 134.5, 133.8, 130.7, 130.3, 129.3, 129.1, 128.3, 128.0, 120.4, 120.3, 52.2, 49.5, 21.4, -2.3, -2.6.
^{19}F NMR (282 MHz, CDCl$_3$) δ -58.1 (s, 3F)
HRMS (ESI) [$C_{26}H_{25}O_4NaSiF_3$] $[M+Na]^+$ calculated for: 509.1366, found: 509.1375.

methyl 4-(3-(dimethyl(phenyl)silyl)-1-(naphthalen-1-yl)-1-oxopropan-2-yl)benzoate (3ka)

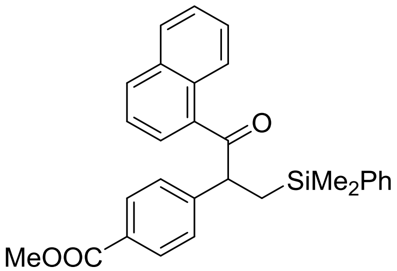

Following the general procedure **B**, the title compound was purified by flash column chromatography (EtOAc/Hexane: 1/10) to afford **3ka** as colorless oil. (69.7 mg, 77% yield).

^{1}H NMR (500 MHz, CDCl$_3$) δ 8.19 – 8.15 (m, 1H), 7.87 – 7.83 (m, 3H), 7.78 – 7.76 (m, 1H), 7.48 – 7.42 (m, 5H), 7.37 – 7.31 (m, 4H), 7.27 – 7.25 (m, 2H), 4.63 – 4.60 (m, 1H), 3.83 (s, 3H), 1.88 (dd, *J* = 14.9, 6.8 Hz, 1H), 1.51 (dd, *J* = 14.9, 8.1 Hz, 1H), 0.19 (s, 3H), 0.14 (s, 3H).
^{13}C NMR (126 MHz, CDCl$_3$) δ 203.3, 166.9, 145.8, 138.2, 136.4, 133.9, 133.8, 132.3, 130.5, 130.1, 129.3, 128.9, 128.5, 128.4, 128.0, 127.9, 126.6, 126.5, 125.5, 124.2, 53.2, 52.1, 20.4, -2.2, -2.5.

HRMS (ESI) [$C_{29}H_{28}O_3NaSi$] $[M+Na]^+$ calculated for: 475.1699, found: 475.1714.

methyl 4-(3-(dimethyl(phenyl)silyl)-1-(naphthalen-2-yl)-1-oxopropan-2-yl)benzoate (3la)

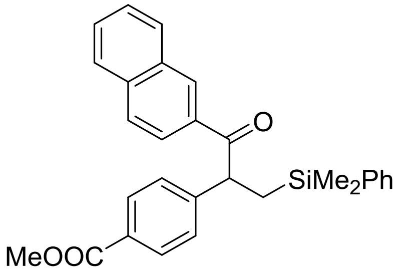

Following the general procedure **B**, the title compound was purified by flash column chromatography (EtOAc/Hexane: 1/10) to afford **3la** as colorless oil. (74.6 mg, 82% yield).
^{1}H NMR (500 MHz, CDCl$_3$) δ 8.29 – 8.22 (m, 1H), 7.91 (d, *J* = 8.1 Hz, 2H), 7.88 (d, *J* = 8.7 Hz, 1H), 7.83 (d, *J* = 8.1 Hz, 1H), 7.79 (t, *J* = 7.7 Hz, 2H), 7.57 – 7.52 (m, 1H), 7.51 (d, *J* = 8.2 Hz, 1H), 7.47 (d, *J* = 5.6 Hz, 2H), 7.39 – 7.30 (m, 5H), 4.79 (t, *J* = 7.4 Hz, 1H), 3.85 (s, 3H), 1.85 (dd, *J* = 14.9, 7.4 Hz, 1H), 1.46 (dd, *J* = 14.9, 7.5 Hz, 1H), 0.18 (s, 3H), 0.14 (s, 3H).
^{13}C NMR (126 MHz, CDCl$_3$) δ 199.8, 166.9, 146.7, 138.3, 135.5, 133.8, 133.7, 132.5, 130.3, 130.2, 129.7, 129.3, 128.9, 128.6, 128.6, 128.4, 128.0, 127.8, 126.8, 124.5, 52.2, 49.3, 21.5, -2.3, -2.5.
HRMS (ESI) [$C_{29}H_{28}O_3NaSi$] $[M+Na]^+$ calculated for: 475.1699, found: 475.1695.

methyl 4-(1-([1,1'-biphenyl]-4-yl)-3-(dimethyl(phenyl)silyl)-1-oxopropan-2-yl)benzoate (3ma)

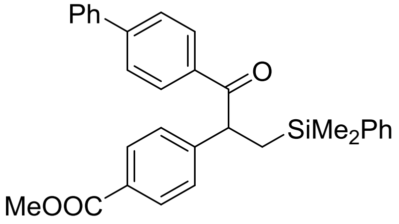

Following the general procedure **B**, the title compound was purified by flash column chromatography (EtOAc/Hexane: 1/10) to afford **3ma** as colorless oil. (77.7 mg, 81% yield).
^{1}H NMR (500 MHz, CDCl$_3$) δ 7.92 (d, *J* = 8.4 Hz, 2H), 7.87 (d, *J* = 8.5 Hz, 2H), 7.55 (dd, *J* = 9.2, 2.9 Hz, 4H), 7.48 – 7.41 (m, 4H), 7.38 – 7.30 (m, 6H), 4.66 (t, *J* = 7.4 Hz, 1H), 3.86 (s, 3H), 1.81 (dd, *J* = 14.9, 7.4 Hz, 1H), 1.43 (dd, *J* = 14.8, 7.5 Hz, 1H), 0.18 (s, 3H), 0.13 (s, 3H).
^{13}C NMR (126 MHz, CDCl$_3$) δ 199.2, 166.8, 146.6, 145.7, 139.8, 138.2, 134.9, 133.7, 130.2, 129.2, 129.0, 128.9, 128.4, 127.9, 127.2, 52.1, 49.3, 21.3, -2.4, -2.6.
HRMS (ESI) [$C_{31}H_{30}O_3NaSi$] $[M+Na]^+$ calculated for: 501.1856, found: 501.1865.

methyl 4-(1-([1,1'-biphenyl]-2-yl)-3-(dimethyl(phenyl)silyl)-1-oxopropan-2-yl)benzoate (3na)

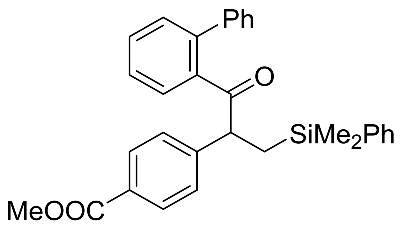

Following the general procedure **B**, the title compound was purified by flash column chromatography (EtOAc/Hexane: 1/10) to afford **3na** as colorless oil. (78.0 mg, 81% yield).
^{1}H NMR (500 MHz, $CDCl_3$) δ 7.74 (d, *J* = 8.4 Hz, 2H), 7.40 – 7.39 (m, 4H), 7.34 – 7.28 (m, 2H), 7.25 – 7.20 (m, 5H), 7.11 – 7.08 (m, 3H), 6.78 (d, *J* = 8.5 Hz, 2H), 3.86 (s, 3H), 3.60 (dd, *J* = 12.2, 3.4 Hz, 1H), 1.49 (dd, *J* = 15.0, 12.2 Hz, 1H), 1.16 (s, 1H), -0.24 (s, 3H), -0.27 (s, 3H).
^{13}C NMR (126 MHz, $CDCl_3$) δ 208.1, 166.9, 144.2, 140.6, 140.1, 139.4, 138.1, 133.4, 130.3, 129.9, 129.5, 129.0, 128.8, 128.7, 128.0, 127.7, 127.3, 53.8, 52.1, 19.5, -2.4, -3.4.
HRMS (ESI) [$C_{31}H_{30}O_3NaSi$] $[M+Na]^+$ calculated for: 501.1856, found: 501.1855.

methyl 4-(3-(dimethyl(phenyl)silyl)-1-oxo-1-(thiophen-2-yl)propan-2-yl)benzoate (3oa)

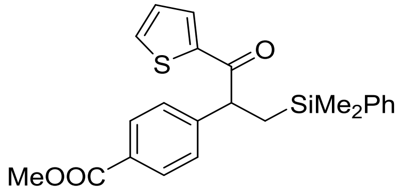

Following the general procedure **B**, the title compound was purified by flash column chromatography (EtOAc/Hexane: 1/10) to afford **3oa** as colorless oil. (79.3 mg, 97% yield).
^{1}H NMR (500 MHz, $CDCl_3$) δ 7.91 (d, *J* = 8.4 Hz, 2H), 7.56 – 7.52 (m, 2H), 7.44 – 7.42 (m, 2H), 7.34 – 7.31 (m, 5H), 7.01 -7.00 (m, 1H), 4.43 (t, *J* = 7.5 Hz, 1H), 3.87 (s, 3H), 1.78 (dd, *J* = 14.9, 7.3 Hz, 1H), 1.43 (dd, *J* = 14.9, 7.7 Hz, 1H), 0.16 (s, 3H), 0.11 (s, 3H).
^{13}C NMR (126 MHz, $CDCl_3$) δ 192.6, 166.9, 146.4, 143.5, 138.1, 134.2, 133.7, 132.3, 130.1, 129.3, 129.0, 128.3, 128.2, 128.0, 52.2, 50.9, 21.2, -2.4, -2.6.
HRMS (ESI) [$C_{23}H_{24}O_3NaSiS$] $[M+Na]^+$ calculated for: 431.1107, found: 431.1102.

methyl 4-(1-(benzofuran-2-yl)-3-(dimethyl(phenyl)silyl)-1-oxopropan-2-yl)benzoate (3pa)

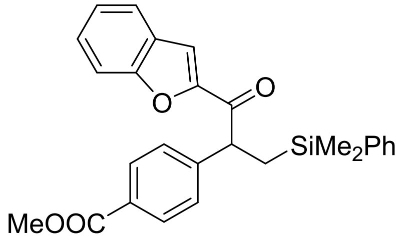

Following the general procedure **B**, the title compound was purified by flash column chromatography (EtOAc/Hexane: 1/15) to afford **3pa** as colorless oil. (51.0 mg, 58% yield).
^{1}H NMR (500 MHz, $CDCl_3$) δ 7.92 (d, *J* = 8.4 Hz, 2H), 7.62 (d, *J* = 7.9 Hz, 1H), 7.52 (d, *J* = 8.5 Hz, 1H), 7.45 – 7.42 (m, 3H), 7.38 (d, *J* = 8.4 Hz, 2H), 7.34 – 7.30 (m, 4H), 7.28 – 7.24 (m, 1H), 4.59 (t, *J* = 7.6 Hz, 1H), 3.87 (s, 3H), 1.81 (dd, *J* = 14.9, 7.3 Hz, 1H), 1.46 (dd, *J* = 14.9, 7.8 Hz, 1H), 0.17 (s, 3H), 0.14 (s, 3H).

^{13}C NMR (126 MHz, CDCl$_3$) δ 190.7, 166.9, 155.7, 151.9, 145.7, 138.1, 133.8, 130.1, 129.3, 129.1, 128.6, 128.4, 128.0, 127.1, 124.0, 123.4, 113.8, 112.5, 52.2, 50.0, 20.3, -2.3, -2.6.
HRMS (ESI) [$C_{27}H_{26}O_4NaSi$] [M+Na]$^+$ calculated for: 465.1492, found: 465.1498.

methyl 4-(1-(dimethyl(phenyl)silyl)-3-oxo-5-phenylpentan-2-yl)benzoate (3qa)

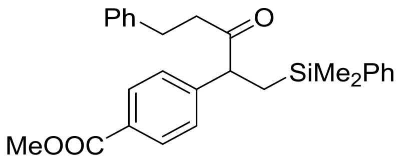

Following the general procedure **B**, the title compound was purified by flash column chromatography (EtOAc/Hexane: 1/15) to afford **3qa** as colorless oil. (62.1 mg, 72% yield).
^{1}H NMR (500 MHz, CDCl$_3$) δ 7.89 (d, *J* = 8.3 Hz, 2H), 7.40 – 7.37 (m, 2H), 7.36 – 7.31 (m, 3H), 7.18 (t, *J* = 7.1 Hz, 2H), 7.13 (d, *J* = 8.4 Hz, 3H), 7.00 – 6.96 (m, 2H), 3.90 (s, 3H), 3.64 – 3.60 (m, 1H), 2.75 – 2.62 (m, 2H), 2.59 – 2.51 (m, 1H), 2.50 – 2.40 (m, 1H), 1.61 (dd, *J* = 14.9, 6.7 Hz, 1H), 1.22 (dd, *J* = 14.9, 8.3 Hz, 1H), 0.11 (s, 3H), 0.06 (s, 3H).
^{13}C NMR (126 MHz, CDCl$_3$) δ 208.8, 166.9, 145.7, 140.9, 138.2, 133.8, 130.2, 129.2, 129.1, 128.5, 128.4, 128.3, 128.0, 126.1, 55.1, 52.2, 42.9, 29.9, 19.0, -2.3, -2.7.
HRMS (ESI) [$C_{27}H_{30}O_3NaSi$] [M+Na]$^+$ calculated for: 453.1856, found: 453.1865.

methyl 4-(3-(dimethyl(phenyl)silyl)-1-oxo-1-(2-phenylcyclopropyl)propan-2-yl)benzoate (3ra)

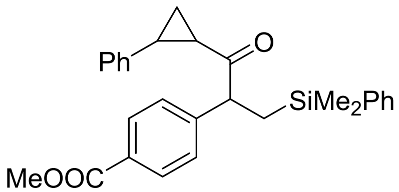

Following the general procedure **B**, the title compound was purified by flash column chromatography (EtOAc/Hexane: 1/12) to afford **3ra** as colorless oil. (60.5 mg, 68% yield).
^{1}H NMR (500 MHz, CDCl$_3$) δ 7.95 (d, *J* = 8.3 Hz, 1H), 7.91 (d, *J* = 8.4 Hz, 1H), 7.41 (dd, *J* = 12.0, 7.8 Hz, 2H), 7.36 – 7.27 (m, 3H), 7.27 – 7.16 (m, 4H), 7.10 (d, *J* = 7.2 Hz, 1H), 6.98 – 6.93 (m, 1H), 6.71 – 6.67 (m, 1H), 3.91 (d, *J* = 4.3 Hz, 3H), 3.89 – 3.83 (m, 1H), 2.46 – 2.21 (m, 1H), 2.01 – 1.89 (m, 1H), 1.74 – 1.65 (m, 1H), 1.60 – 1.49 (m, 1H), 1.33 – 1.15 (m, 2H), 0.14 (d, *J* = 5.5 Hz, 3H), 0.09 (s, 3H).
^{13}C NMR (126 MHz, CDCl$_3$) δ 207.8, 167.0, 145.9, 140.0, 138.3, 133.7, 130.2, 129.2, 128.6, 128.4, 128.0, 126.7, 126.5, 126.2, 55.8, 52.3, 31.8, 30.2, 19.3, 18.6, -2.3, -2.6.
HRMS (ESI) [$C_{28}H_{30}O_3NaSi$] [M+Na]$^+$ calculated for: 465.1856, found: 465.1850.

methyl 4-(1-(3,4-diethoxyphenyl)-3-(dimethyl(phenyl)silyl)-1-oxopropan-2-yl)benzoate (3sa)

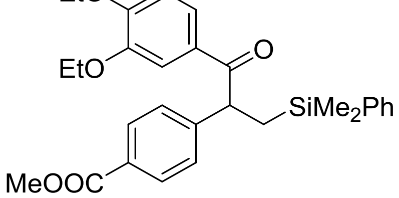

Following the general procedure **B**, the title compound was purified by flash column chromatography (EtOAc/Hexane: 1/10) to afford **3sa** as colorless oil. (74.1 mg, 76% yield).
^{1}H NMR (500 MHz, CDCl$_3$) δ 7.90 (d, *J* = 8.4 Hz, 2H), 7.45 – 7.43 (m, 2H), 7.40 - 7.39 (m, 2H), 7.35 – 7.29 (m, 5H), 6.75 (d, *J* = 8.9 Hz, 1H), 4.60 (t, *J* = 7.5 Hz, 1H), 4.12 – 4.03 (m, 4H), 3.87 (s, 3H), 1.77 (dd, *J* = 14.8, 7.4 Hz, 1H), 1.46 – 1.38 (m, 7H), 0.15 (s, 3H), 0.10 (s, 3H).
^{13}C NMR (126 MHz, CDCl$_3$) δ 198.3, 167.0, 153.1, 148.5, 147.2, 138.4, 133.8, 130.1, 129.2, 128.8, 128.3, 127.9, 123.1, 112.7, 111.3, 64.5, 52.2, 48.7, 21.5, 14.8, 14.7, -2.3, -2.5.
HRMS (ESI) [$C_{29}H_{34}O_5NaSi$] [M+Na]$^+$ calculated for: 513.2067, found: 513.2075.

methyl 4-(1-(4-bromo-3-methylphenyl)-3-(dimethyl(phenyl)silyl)-1-oxopropan-2-yl)benzoate (3ta)

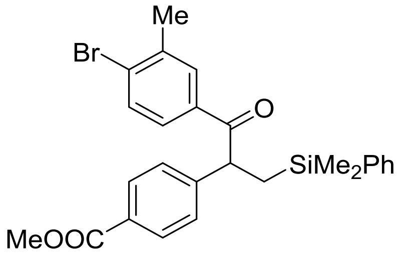

Following the general procedure, the title compound was purified by flash column chromatography (EtOAc/Hexane: 1/10) to afford **3ta** as colorless oil. (92.3 mg, 93% yield).
^{1}H NMR (500 MHz, CDCl$_3$) δ 7.91 (d, *J* = 8.4 Hz, 2H), 7.61 (d, *J* = 2.7 Hz, 1H), 7.48 (d, *J* = 8.4 Hz, 1H), 7.42 (dd, *J* = 14.7, 8.1 Hz, 3H), 7.37 – 7.31 (m, 3H), 7.26 (d, *J* = 8.4 Hz, 2H), 4.54 (t, *J* = 7.4 Hz, 1H), 3.87 (s, 3H), 2.35 (s, 3H), 1.77 (dd, *J* = 14.9, 7.5 Hz, 1H), 1.38 (dd, *J* = 14.9, 7.4 Hz, 1H), 0.17 (s, 3H), 0.11 (s, 3H).
^{13}C NMR (126 MHz, CDCl$_3$) δ 199.1, 166.8, 146.3, 138.6, 138.1, 135.3, 133.8, 132.7, 130.8, 130.2, 129.3, 129.0, 128.3, 128.0, 127.3, 52.2, 49.3, 23.1, 21.3, -2.3, -2.5.
HRMS (ESI) [$C_{26}H_{27}O_3NaSiBr$] [M+Na]$^+$ calculated for: 517.0805, found: 517.0804.

methyl 4-(1-(4-((tert-butyldimethylsilyl)oxy)phenyl)-3-(dimethyl(phenyl)silyl)-1-oxopropan-2-yl)benzoate (3ua)

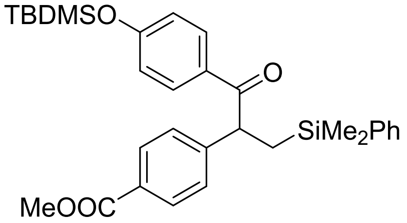

Following the general procedure, the title compound was purified by flash column chromatography (EtOAc/*n*-hexane: 1/20) to afford **3ua** as colorless oil. (59.8 mg, 56% yield).
^{1}H NMR (500 MHz, CDCl$_3$) δ 7.90 (d, *J* = 8.4 Hz, 2H), 7.73 (d, *J* = 8.8 Hz, 2H), 7.43 (dd, *J* = 7.6, 1.7 Hz, 2H), 7.35 – 7.32 (m, 3H), 7.29 (d, *J* = 8.4 Hz, 2H), 6.75 (d, *J* = 8.8 Hz, 2H), 4.58 (t, *J* = 7.5 Hz, 1H), 3.87 (s, 3H), 1.77 (dd, *J* = 14.9, 7.4 Hz, 1H), 1.39 (dd, *J* = 14.8, 7.4 Hz, 1H), 0.96 (s, 9H), 0.19 (s, 6H), 0.15 (s, 3H), 0.11 (s, 3H).
^{13}C NMR (176 MHz, CDCl$_3$) δ 198.4, 167.0, 160.3, 147.1, 138.4, 133.8, 130.9, 130.2, 129.8, 129.2, 128.8, 128.4, 128.0, 120.0, 52.2, 48.9, 25.7, 21.5, 18.3, -2.3, -2.5, -4.3.
HRMS (ESI) [$C_{31}H_{40}O_4Si_2Na$] [M+Na]$^+$, calculated for: 555.2357, found: 555.2371.

methyl 4-(1-(4-(butylcarbamoyl)phenyl)-3-(dimethyl(phenyl)silyl)-1-oxopropan-2-yl)benzoate (3va)

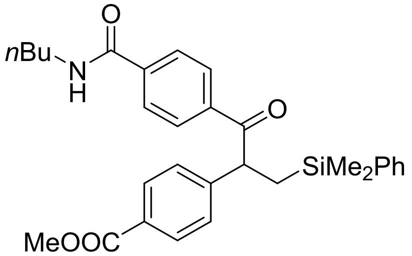

Following the general procedure, the title compound was purified by flash column chromatography (EtOAc/*n*-hexane: 1/3) to afford **3va** as colorless oil. (63.6 mg, 63% yield).

^{1}H NMR (500 MHz, CDCl$_3$) δ 7.89 (d, *J* = 8.4 Hz, 2H), 7.78 (d, *J* = 8.6 Hz, 2H), 7.70 (d, *J* = 8.5 Hz, 2H), 7.44 – 7.42 (m, 2H), 7.35 – 7.30 (m, 3H), 7.25 (d, *J* = 8.4 Hz, 2H), 6.26 (t, *J* = 5.8 Hz, 1H), 4.59 (t, *J* = 7.4 Hz, 1H), 3.86 (s, 3H), 3.45 – 3.34 (m, 2H), 1.78 (dd, *J* = 14.9, 7.4 Hz, 1H), 1.60 – 1.52 (m, 2H), 1.41 – 1.35 (m, 3H), 0.92 (t, *J* = 7.4 Hz, 3H), 0.17 (s, 3H), 0.12 (s, 3H).

^{13}C NMR (176 MHz, CDCl$_3$) δ 199.2, 166.8, 166.6, 146.1, 138.7, 138.4, 138.1, 133.8, 130.3, 129.3, 129.1, 128.8, 128.4, 128.0, 127.2, 52.2, 49.7, 40.0, 31.7, 21.2, 20.2, 13.8, -2.3, -2.6.

HRMS (ESI) [$C_{30}H_{35}NO_4SiNa$] [M+Na]$^+$, calculated for: 524.2227, found: 524.2239.

methyl 4-(1-(4-((tert-butoxycarbonyl)amino)phenyl)-3-(dimethyl(phenyl)silyl)-1-oxopropan-2-yl)benzoate (3wa)

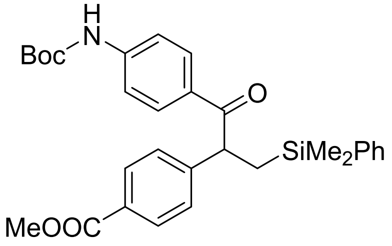

Following the general procedure, the title compound was purified by flash column chromatography (EtOAc/*n*-hexane: 1/5) to afford **3wa** as colorless oil. (23.5 mg, 23% yield).

^{1}H NMR (500 MHz, CDCl$_3$) δ 7.88 (d, *J* = 8.38 Hz, 2H), 7.76 (d, *J* = 8.80 Hz, 2H), 7.43 (dd, *J* = 7.56, 1.76 Hz, 2H), 7.36 – 7.32 (m, 5H), 7.27 (d, *J* = 8.38 Hz, 2H), 6.67 (s, 1H), 4.58 (t, *J* = 7.43 Hz, 1H), 3.87 (s, 3H), 1.77 (dd, *J* = 14.88, 7.42 Hz, 1H), 1.50 (s, 9H), 1.38 (dd, *J* = 14.86, 7.42 Hz, 1H), 0.15 (s, 3H), 0.10 (s, 3H).

^{13}C NMR (176 MHz, CDCl$_3$) δ 198.4, 167.0, 152.2, 146.9, 142.9, 138.4, 133.8, 130.9, 130.3, 130.2, 129.2, 128.8, 128.4, 128.0, 117.5, 81.5, 52.2, 48.9, 28.4, 21.4, -2.3, -2.5.

HRMS (ESI) [$C_{30}H_{35}NO_5SiNa$] [M+Na]$^+$, calculated for: 540.2176, found: 540.2185.

methyl 4-(3-(dimethyl(phenyl)silyl)-1-(4-(hydroxymethyl)phenyl)-1-oxopropan-2-yl)benzoate (3xa)

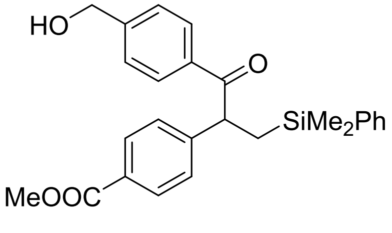

Following the general procedure, the title compound was purified by flash column chromatography (EtOAc/*n*-hexane: 1/3) to afford **3xa** as colorless oil. (31.0 mg, 36% yield).
^{1}H NMR (500 MHz, CDCl$_3$) δ 7.89 (d, *J* = 8.45 Hz, 2H), 7.78 (d, *J* = 8.46 Hz, 2H), 7.44 (dd, *J* = 7.64, 1.79 Hz, 2H), 7.38 – 7.31 (m, 5H), 7.27 (d, *J* = 8.47 Hz, 2H), 4.70 (s, 2H), 4.61 (t, *J* = 7.44 Hz, 1H), 3.87 (s, 3H), 1.78 (dd, *J* = 14.91, 7.37 Hz, 1H), 1.39 (dd, *J* = 14.86, 7.44 Hz, 1H), 0.16 (s, 3H), 0.11 (s, 3H).
^{13}C NMR (176 MHz, CDCl$_3$) δ 199.4, 167.0, 146.6, 146.1, 138.3, 135.6, 133.8, 130.2, 129.3, 129.0, 128.9, 128.4, 128.0, 126.8, 64.7, 52.2, 49.4, 21.3, -2.3, -2.5.
HRMS (ESI) [$C_{26}H_{28}O_4NaSi$] $[M+Na]^+$, calculated for: 455.1649, found: 455.1661.

methyl 4-(1-(2-acetoxyphenyl)-3-(dimethyl(phenyl)silyl)-1-oxopropan-2-yl)benzoate (3ya)

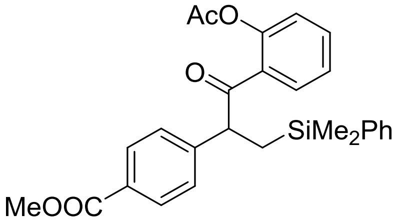

Following the general procedure B, the title compound was purified by flash column chromatography (EtOAc/n-*h*exane: 1/5) to afford **3ya** as colorless oil. (67.9 mg, 74% yield).
^{1}H NMR (500 MHz, CDCl$_3$) δ 7.88 (d, *J* = 8.3 Hz, 2H), 7.44 – 7.30 (m, 7H), 7.19 (d, *J* = 8.3 Hz, 2H), 7.15 – 7.11 (m, 1H), 7.01 (d, *J* = 8.1 Hz, 1H), 4.45 – 4.37 (m, 1H), 3.87 (s, 3H), 2.21 (s, 3H), 1.68 (dd, *J* = 15.0, 6.1 Hz, 1H), 1.42 (dd, *J* = 15.0, 8.9 Hz, 1H), 0.14 (s, 3H), 0.05 (s, 3H).
^{13}C NMR (126 MHz, CDCl$_3$) δ 199.8, 169.4, 166.9, 148.9, 145.6, 138.2, 133.8, 132.9, 130.8, 130.1, 129.6, 129.3, 129.1, 128.6, 128.0, 125.9, 124.1, 52.4, 52.2, 21.1, 20.5, -2.2, -2.7.
HRMS (ESI) [$C_{27}H_{28}O_5NaSi$] $[M+Na]^+$ calculated for: 483.1598, found: 483.1605.

methyl 4-(1-(2-(3-cyano-4-isobutoxyphenyl)-4-methylthiazol-5-yl)-3-(dimethyl(phenyl)silyl)-1-oxopropan-2-yl)benzoate (3za)

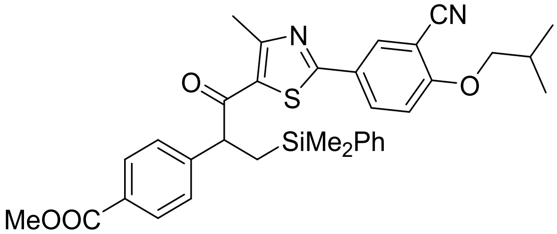

Following the general procedure **B**, the title compound was purified by flash column chromatography (EtOAc/Hexane: 1/5) to afford **3za** as pale yellow solid. (50.9 mg, 43% yield).
m.p. = 137.8 – 138.5 °C.
^{1}H NMR (500 MHz, CDCl$_3$) δ 8.09 (d, *J* = 2.3 Hz, 1H), 8.05 (dd, *J* = 8.8, 2.3 Hz, 1H), 7.95 (d, *J* = 8.4 Hz, 2H), 7.47 – 7.42 (m, 2H), 7.37 – 7.31 (m, 5H), 6.99 (d, *J* = 8.9 Hz, 1H), 4.17 (t, *J* = 7.3 Hz, 1H), 3.89 (s, 5H), 2.67 (s, 3H), 2.20 (sep, 1H), 1.81 (dd, *J* = 14.9, 7.5 Hz, 1H), 1.38 (dd, *J* = 14.9, 7.2 Hz, 1H), 1.09 (d, *J* = 6.7 Hz, 6H), 0.20 (s, 3H), 0.15 (s, 3H).

^{13}C NMR (126 MHz, CDCl$_3$) δ 192.5, 166.8, 166.4, 162.7, 161.9, 145.7, 137.9, 133.8, 132.7, 132.3, 130.2, 129.3, 128.8, 128.4, 128.0, 125.8, 115.5, 112.7, 103.0, 75.8, 55.1, 52.3, 28.3, 21.6, 19.2, 18.6, -2.4, -2.7.
HRMS (ESI) [$C_{34}H_{36}O_4NaSiS$] $[M+Na]^+$ calculated for: 619.2057, found: 619.2062.

methyl 4-(3-(dimethyl(phenyl)silyl)-1-(4-(N,N-dipropylsulfamoyl)phenyl)-1-oxopropan-2-yl)benzoate (3zaa)

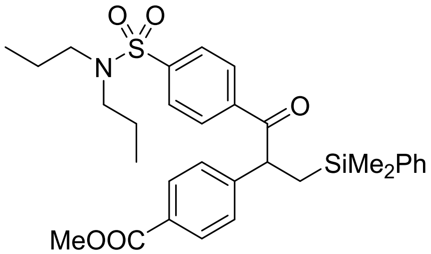

Following the general procedure **B**, the title compound was purified by flash column chromatography (EtOAc/*n*-hexane: 1/7) to afford **3zaa** as colorless oil. (95.4 mg, 84% yield).
^{1}H NMR (500 MHz, CDCl$_3$) δ 7.92 (d, *J* = 8.4 Hz, 2H), 7.82 (d, *J* = 8.6 Hz, 2H), 7.75 (d, *J* = 8.6 Hz, 2H), 7.44 – 7.40 (m, 2H), 7.36 – 7.30 (m, 3H), 7.26 (d, *J* = 8.4 Hz, 2H), 4.56 (t, *J* = 7.4 Hz, 1H), 3.88 (s, 3H), 3.08 – 3.00 (m, 4H), 1.78 (dd, *J* = 14.9, 7.4 Hz, 1H), 1.51 (dq, *J* = 14.9, 7.4 Hz, 4H), 1.40 (dd, *J* = 14.9, 7.4 Hz, 1H), 0.84 (t, *J* = 7.4 Hz, 6H), 0.18 (s, 3H), 0.12 (s, 3H).
^{13}C NMR (126 MHz, CDCl$_3$) δ 198.8, 166.7, 145.6, 143.9, 139.0, 137.8, 133.7, 130.3, 129.3, 129.1, 129.0, 128.3, 128.0, 127.1, 52.2, 50.0, 49.9, 22.0, 21.2, 11.2, -2.4, -2.7.
HRMS (ESI) [$C_{31}H_{39}O_5NaSiS$] $[M+Na]^+$ calculated for: 588.2210, found: 588.2224.

methyl 4-(1-(6-(3-(adamantan-1-yl)-4-methoxyphenyl)naphthalen-2-yl)-3-(dimethyl(phenyl)silyl)-1-oxopropan-2-yl)benzoate (3zba)

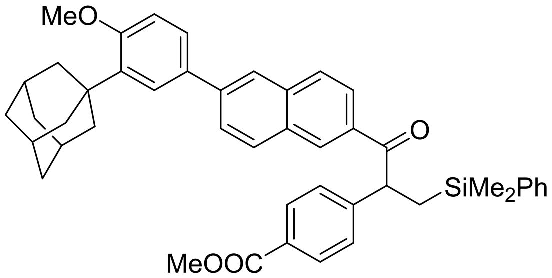

Following the general procedure **B** at reaction temperature of 100℃, the title compound was purified by flash column chromatography (DCM/EtOAc/Hexane: 1/1/10) to afford **3zba** as colorless oil. (112.2 mg, 81% yield).
^{1}H NMR (500 MHz, CDCl$_3$) δ 8.25 (s, 1H), 7.96 – 7.85 (m, 5H), 7.82 (d, *J* = 8.7 Hz, 1H), 7.76 (d, *J* = 10.3 Hz, 1H), 7.58 (s, 1H), 7.51 (dd, *J* = 8.4, 2.3 Hz, 1H), 7.49 – 7.46 (m, 2H), 7.40 – 7.34 (m, 5H), 6.98 (d, *J* = 8.5 Hz, 1H), 4.80 (t, *J* = 7.4 Hz, 1H), 3.89 (s, 3H), 3.86 (s, 3H), 2.17 (s, 6H), 2.10 (s, 3H), 1.86 (dd, *J* = 14.9, 7.4 Hz, 1H), 1.79 (s, 6H), 1.46 (dd, *J* = 14.8, 7.5 Hz, 1H), 0.19 (s, 3H), 0.14 (s, 3H).
^{13}C NMR (126 MHz, CDCl$_3$) δ 199.7, 166.9, 159.1, 146.8, 141.8, 139.1, 138.3, 136.0, 133.9, 133.3, 132.5, 131.2, 130.2, 130.1, 129.3, 128.9, 128.6, 128.5, 128.1, 126.6, 126.1, 125.9, 124.9, 124.7, 112.2, 55.3, 52.2, 49.2, 40.7, 37.3, 37.2, 29.2, 21.6, -2.3, -2.5, (a missed carbon).

HRMS (ESI) [$C_{46}H_{48}O_4NaSi$] $[M+Na]^+$ calculated for: 715.3214, found: 715.3224.

methyl 4-(3-(dimethyl(phenyl)silyl)-1-((8R,9S,13S,14S)-13-methyl-17-oxo-7,8,9,11,12,13,14,15,16,17-decahydro-6H-cyclopenta[a]phenanthren-3-yl)-1-oxopropan-2-yl)benzoate (3zca)

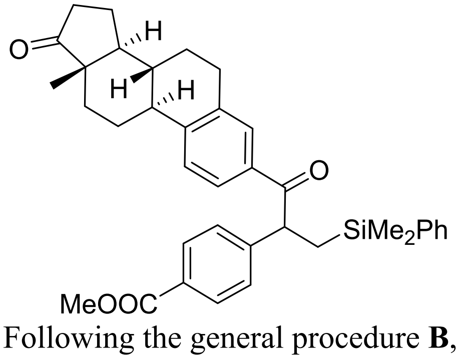

Following the general procedure **B**, the title compound was purified by flash column chromatography (DCM/EtOAc/Hexane: 1/1/5) to afford **3zca** as white solid. (85.8 mg, 74% yield).

m.p. = 54.9 – 55.7 °C.

^{1}H NMR (500 MHz, CDCl$_3$) δ 7.89 (d, *J* = 8.4 Hz, 2H), 7.60 – 7.55 (m, 1H), 7.55 (d, *J* = 1.9 Hz, 1H), 7.47 – 7.43 (m, 2H), 7.39 – 7.32 (m, 3H), 7.29 (d, *J* = 8.2 Hz, 2H), 7.26 (d, *J* = 9.7 Hz, 1H), 4.62 (t, *J* = 8.0 Hz, 1H), 3.87 (s, 3H), 2.92 – 2.83 (m, 2H), 2.50 (dd, *J* = 19.0, 8.8 Hz, 1H), 2.42 – 2.35 (m, 1H), 2.26 (tt, *J* = 10.5, 4.7 Hz, 1H), 2.18 – 2.09 (m, 1H), 2.09 – 1.98 (m, 2H), 1.95 (dd, *J* = 9.1, 2.9 Hz, 1H), 1.78 (dd, *J* = 14.9, 7.4 Hz, 1H), 1.67 – 1.35 (m, 7H), 0.88 (d, *J* = 4.6 Hz, 3H), 0.15 (s, 3H), 0.11 (s, 3H).

^{13}C NMR (126 MHz, CDCl$_3$) δ 199.5, 166.9, 146.8, 145.5, 138.4, 137.0, 133.9, 133.7, 130.1, 129.4, 129.2, 128.8, 128.4, 128.0, 126.1, 125.6, 52.2, 50.5, 49.0, 47.9, 44.7, 37.8, 35.9, 31.6, 29.4, 26.3, 25.5, 21.7, 21.3, 13.9, -2.3, -2.5.

HRMS (ESI) [$C_{37}H_{42}O_4NaSi$] $[M+Na]^+$ calculated for: 601.2746, found: 601.2745.

methyl 4-(3-(dimethyl(phenyl)silyl)-1-oxo-1-(3-vinylphenyl)propan-2-yl)benzoate (3zda)

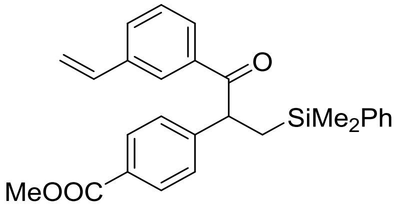

Following the general procedure, the title compound was purified by flash column chromatography (EtOAc/*n*-hexane: 1/15) to afford **3zda** as colorless oil. (68.2 mg, 80% yield).

^{1}H NMR (500 MHz, CDCl$_3$) δ 7.9 (d, *J* = 8.4 Hz, 2H), 7.8 (t, *J* = 1.8 Hz, 1H), 7.6 (dt, *J* = 7.8, 1.5 Hz, 1H), 7.5 (d, *J* = 7.7 Hz, 1H), 7.5 – 7.4 (m, 2H), 7.4 – 7.3 (m, 6H), 6.7 (dd, *J* = 17.6, 10.9 Hz, 1H), 5.7 (dd, *J* = 17.7, 0.7 Hz, 1H), 5.3 (dd, *J* = 10.9, 0.7 Hz, 1H), 4.6 (d, *J* = 7.5 Hz, 1H), 3.9 (s, 3H), 1.8 (dd, *J* = 14.9, 7.4 Hz, 1H), 1.4 (dd, *J* = 14.9, 7.5 Hz, 1H), 0.2 (s, 3H), 0.1 (s, 3H).

^{13}C NMR (126 MHz, CDCl$_3$) δ 199.7, 166.9, 146.5, 138.2, 138.1, 136.7, 136.0, 133.8, 130.6, 130.2, 129.3, 128.9, 128.8, 128.4, 128.0, 127.9, 126.5, 115.4, 52.2, 49.4, 21.4, -2.3, -2.5.

HRMS (ESI) [$C_{27}H_{28}O_3NaSi$] $[M+Na]^+$ calculated for: 452.1700, found: 451.1716.

7. Synthetic application

7. 1 Tamao-Fleming oxidation using compound **3ad**

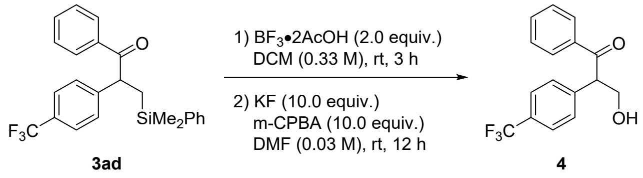

In a nitrogen filled glovebox, to a flame-dried screw-capped test tube was added **3da** (41.3 mg), DCM (0.3 mL), and boron trifluoride acetic acid complex (37.6 mg) sequentially. Then, the tube was sealed and moved out from the glovebox. The solution was stirred at room temperature for 3 hours. After the starting material fully consumed by checking TLC, the reaction mixture was quenched with 1 M aq. $NaHCO_3$. After this, the resulting mixture was extracted with Et_2O, washed with Brine, dried over Na_2SO_4, filtered, and concentrated under reduced pressure. The resulting residue was used directly for the next step without purification. To a flame-dried screw-capped test was charged with the crude and DMF (3.3 mL) under nitrogen atmosphere. To the solution was added KF (58.1 mg) and *m*-CPBA (224.1 mg) at room temperature sequentially. The reaction mixture was stirred for 12 hours at the same temperature. After the reaction was completed by checking TLC, the reaction mixture was diluted with EtOAc, quenched with sat. aq. $Na_2S_2O_4$, washed with water, sat. aq. $NaHCO_3$, and Brine sequentially. The combined organic layers were dried over Na_2SO_4, filtered, and concentrated under reduced pressure. The resulting residue was purified by column chromatography on silica gel (*n*-hexane/EtOAc = 9:1→3:7) to afford **4** as a colorless oil (25.9 mg, 88% yield).

^{1}H NMR (500 MHz, CDCl$_3$) δ 7.93 – 7.91 (m, 2H), 7.58 (d, *J* = 8.2 Hz, 2H), 7.53 – 7.50 (m, 1H), 7.42 – 7.38 (m, 4H), 4.86 (dd, *J* = 8.0, 4.7 Hz, 1H), 4.26 (dd, *J* = 11.3, 8.0 Hz, 1H), 3.93 (dd, *J* = 11.3, 4.7 Hz, 1H), 2.48 (brs, 1H).

^{13}C NMR (176 MHz, CDCl$_3$) δ 199.5, 140.4, 136.1, 133.8, 130.1 (q, *J* = 32.6 Hz), 129.0, 129.0, 128.9, 126.3 (q, *J* = 4.1 Hz), 124.1 (q, *J* = 272.2 Hz), 65.1, 56.0.

^{19}F NMR (658 MHz, CDCl$_3$) δ -63.17 (s, 3F).

HRMS (ESI) [$C_{16}H_{13}F_3O_2Na$] $[M+Na]^+$ calculated for: 317.0759, found: 317.0767.

7. 2 Desilylation of compound **3ad**

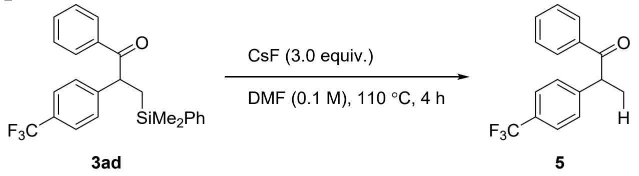

The Desilylation was performed according to the literature with modification.[21] In a nitrogen filled glovebox, to a flame-dried screw-capped test tube was added **3ad** (41.3 mg), DMF (1.0 mL), and cesium fluoride (45.6 mg) sequentially. Then, the tube was sealed and moved out from the glovebox. The solution warmed up to 110 °C and stirred for 4 hours. After the starting material fully consumed by checking TLC, the reaction mixture was cooled to room temperature, diluted with EtOAc, and quenched with water. After this, the resulting mixture was extracted with EtOAc, washed with Brine, dried over Na_2SO_4, filtered, and concentrated under reduced pressure. The resulting residue was purified by column chromatography on silica gel (*n*-hexane/EtOAc = 12:1) to afford the desired product **5** as a pale-yellow oil (19.4 mg, 70% yield).

^{1}H NMR (500 MHz, CDCl$_3$) δ 7.95 – 7.93 (m, 2H), 7.55 (d, J = 7.8 Hz, 2H), 7.53 – 7.49 (m, 1H), 7.42 – 7.39 (m, 4H), 4.77 (q, J = 6.9 Hz, 1H), 1.55 (d, J = 6.9 Hz, 3H).
^{13}C NMR (176 MHz, CDCl$_3$) δ 199.8, 145.5, 136.2, 133.3, 129.4 (q, J = 32.7 Hz), 128.9, 128.8, 128.3, 126.1 (d, J = 3.5 Hz), 124.2 (q, J = 271.9 Hz), 47.6, 19.6.
^{19}F NMR (658 MHz, CDCl$_3$) δ -63.06 (s, 3F).
MS(EI): m/z 278 $[M]^+$.
The chemical shifts were consistent with reported literature.[22]

7. 3 Olefination of compound **3ad**

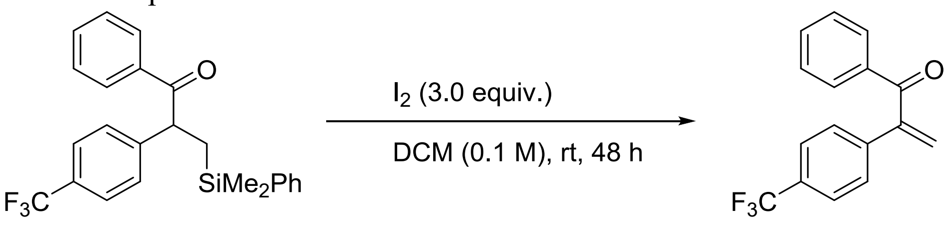

3ad **6**

To stirred solution of **3ad** (41.3 mg) in DCM (1.0 mL) under nitrogen atmosphere was added iodine (76.1 mg) at room temperature. The reaction mixture was stirred for 48 hours at the same temperature. The mixture was then diluted with Et_2O and quenched with sat. aq. $Na_2S_2O_4$ sequentially. The resulting mixture was extracted with Et_2O. The combined organic phases were washed with brine and dried over Na_2SO_4. After filtration, the filtrate was concentrated under reduced pressure and the resulting residue was purified by column chromatography on silica gel (*n*-hexane/DCM = 7:3) to afford the desired product **6** as a white solid (26.1 mg, 78% yield).
m.p. = 41.4 - 41.9℃
^{1}H NMR (500 MHz, CDCl$_3$) δ 7.91 – 7.88 (m, 2H), 7.62 (dd, J = 8.3, 0.7 Hz, 2H), 7.60 – 7.57 (m, 1H), 7.55 (dd, J = 8.7, 0.9 Hz, 2H), 7.48 – 7.44 (m, 2H), 6.18 (s, 1H), 5.79 (s, 1H).
^{13}C NMR (176 MHz, CDCl$_3$) 196.9, 147.1, 140.7, 137.0, 133.5, 130.5, 130.1, 128.7, 127.7, 125.7, 124.2, 123.8.
^{19}F NMR (658 MHz, CDCl$_3$) δ -63.22 (s, 3F).
HRMS (ESI) $[C_{16}H_{11}OF_3Na]$ $[M+Na]^+$ calculated for: 299.0654, found: 299.0654.

8. NMR experiment for detecting the generation of FBpin

In a nitrogen filled glovebox, to a flame-dried screw-capped test tube was added CuOAc (5 mol%), PCy_3 (6 mol%), DMF-d_7 (0.5 M), benzoyl fluoride (0.1 mmol), methyl 4-vinylbenzoate (1.3 equiv), $PhMe_2SiBpin$ (1.3 equiv) sequentially. The tube then was sealed and moved out from the glovebox. The solution was stirred at 40°C for 24 hours. The reaction mixture was directly transferred into a nmr tube to check ^{19}F NMR. The generated Fluorine peak at 152.7 ppm was assigned as FBpin according to the known literature.[23]

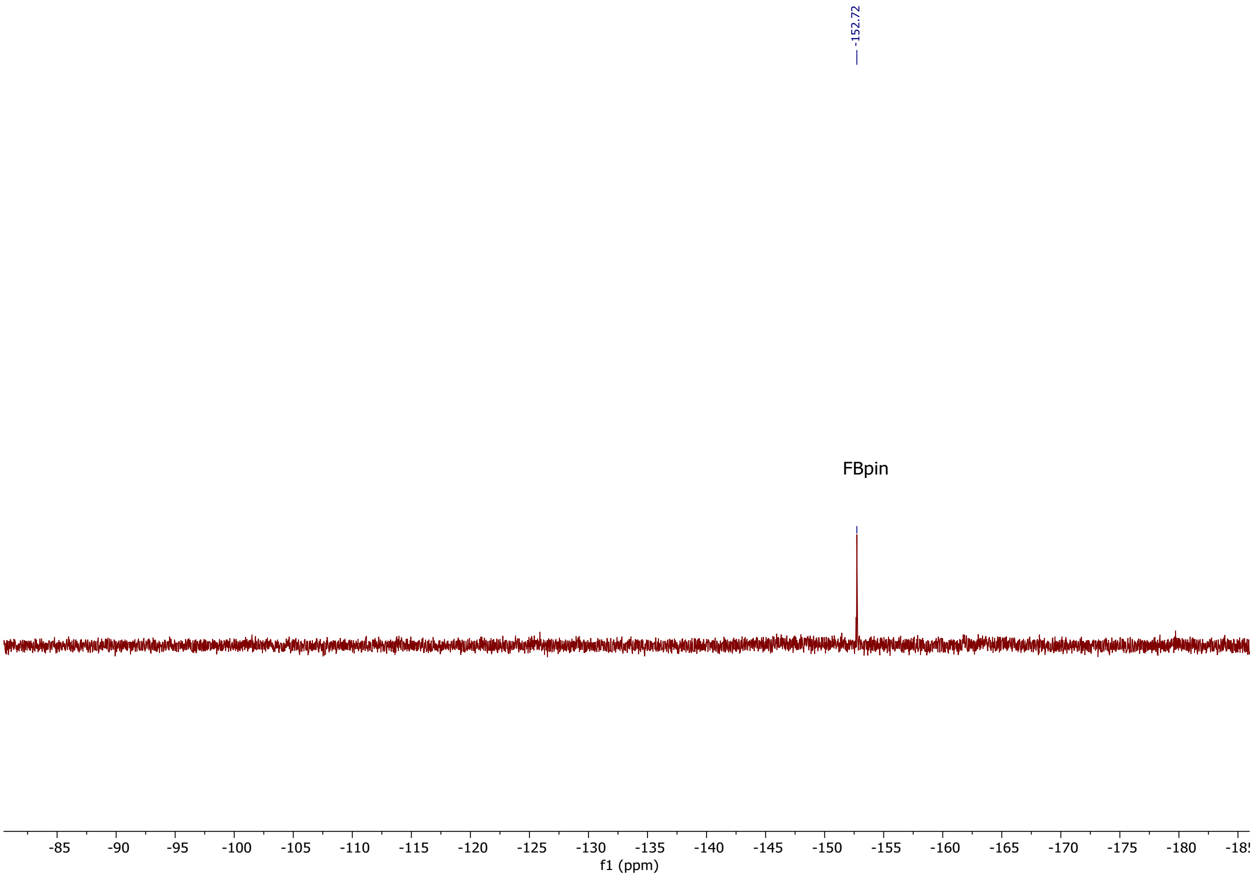

9. Chemoselectivity Studies

To investigate the chemoselectivity of acyl fluoride towards vinyl arenes, three competition studies below were conducted following the general procedure **B**. The reactions were carried out at 24 h, after which ^{1}H NMR analysis was conducted using trimethoxymethane as internal standard.

a).

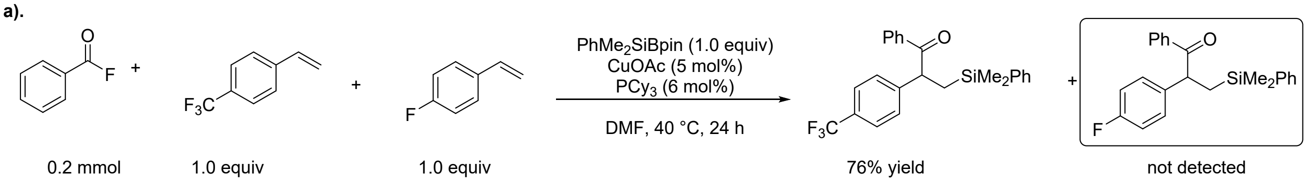
PhMe2SiBpin (1.0 equiv)
CuOAc (5 mol%)
PCy3 (6 mol%)
DMF, 40 °C, 24 h
0.2 mmol
1.0 equiv
1.0 equiv
76% yield
not detected

b).

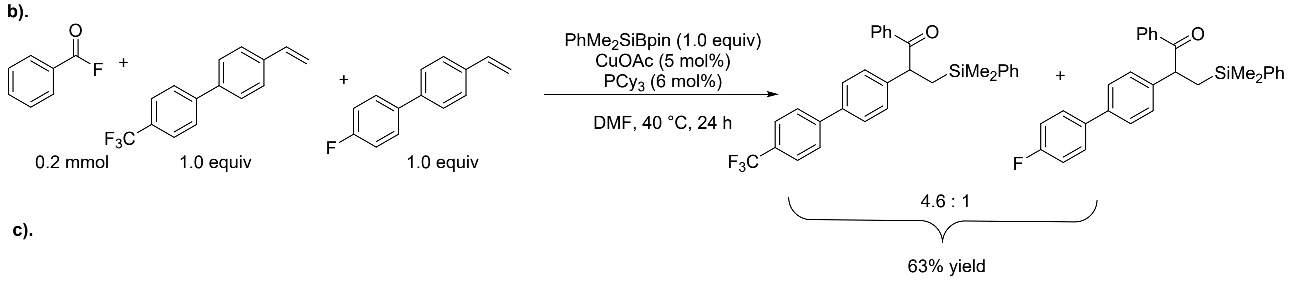
PhMe2SiBpin (1.0 equiv)
CuOAc (5 mol%)
PCy3 (6 mol%)
DMF, 40 °C, 24 h
0.2 mmol
1.0 equiv
1.0 equiv
4.6 : 1
63% yield

c).

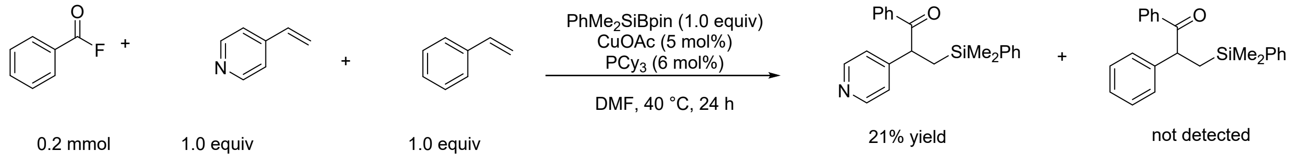
PhMe2SiBpin (1.0 equiv)
CuOAc (5 mol%)
PCy3 (6 mol%)
DMF, 40 °C, 24 h
0.2 mmol
1.0 equiv
1.0 equiv
21% yield
not detected

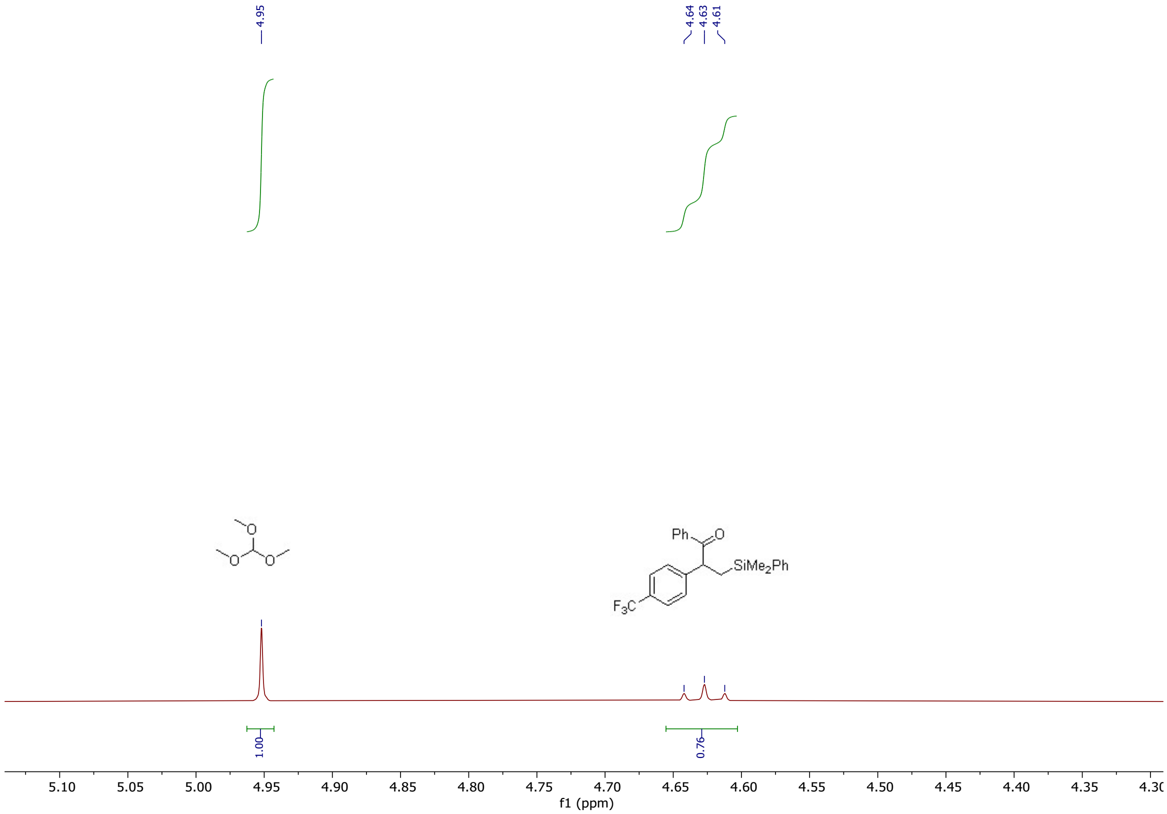
4.95
4.64
4.63
4.61
1.00
0.76
f1 (ppm)

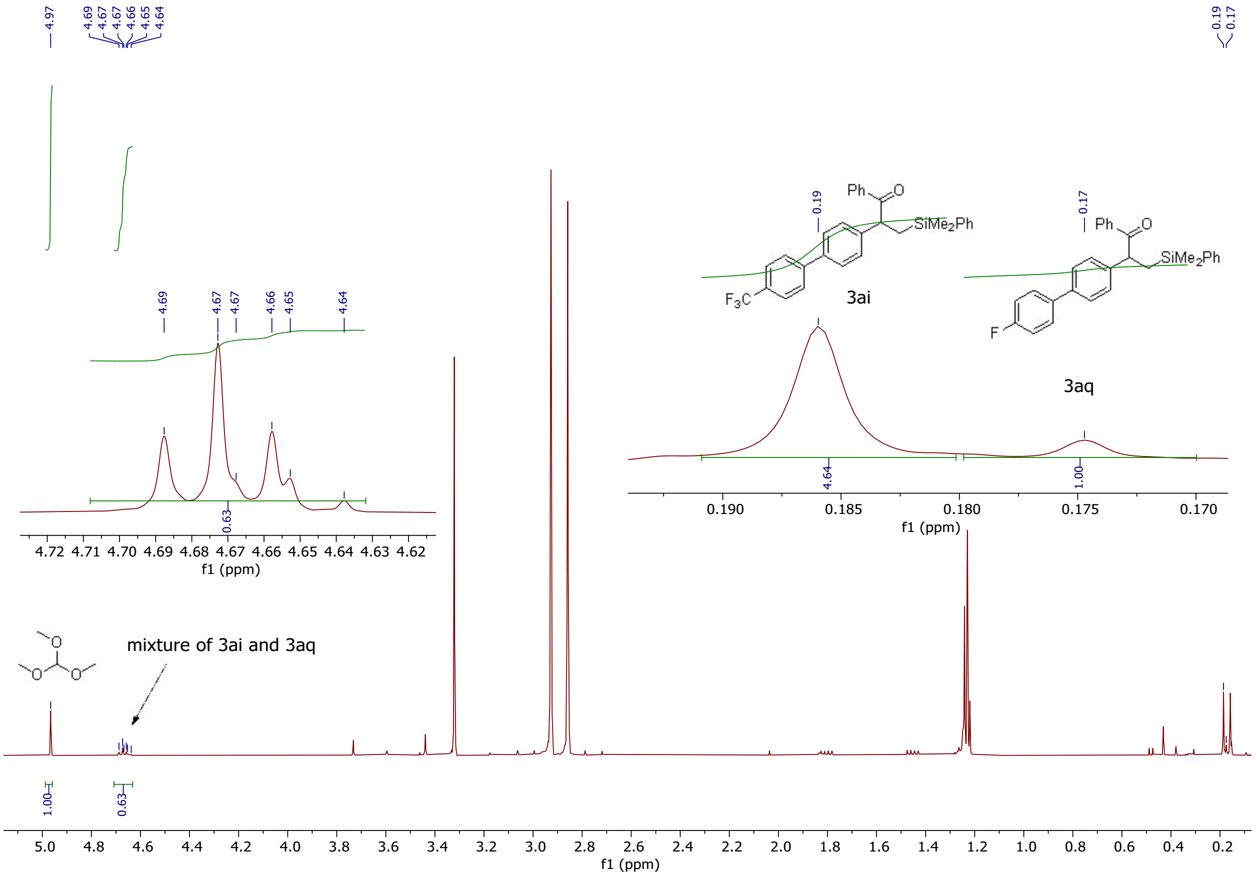

3ai
3aq
mixture of 3ai and 3aq
f1 (ppm)

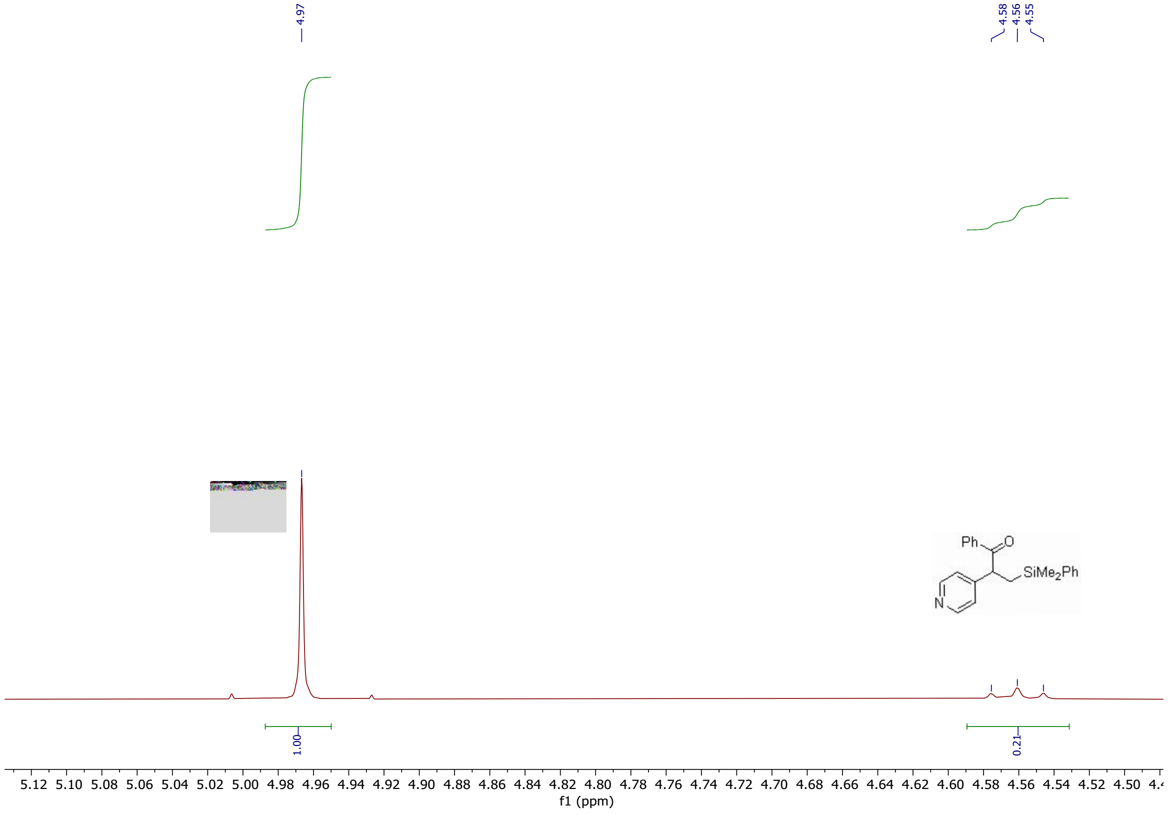

f1 (ppm)

10. References

1. Y. Liang, Z. Zhao, A. Taya, N. Shibata, Acyl Fluorides from Carboxylic Acids, Aldehydes, or Alcohols under Oxidative Fluorination. *Org. Lett.* 2021, **23**, 847–852.

2. Y. Liang, A. Taya, Z. Zhao, Z. N. Shibata, Deoxyfluorination of acyl fluorides to trifluoromethyl compounds by FLUOLEAD®/Olah's reagent under solvent-free conditions. *Beilstein J. Org. Chem*. 2020, **16**, 3052–3058.

3. H. Fujimoto, T. Kodama, M. Yamanaka, M. Tobisu, Phosphine-Catalyzed Intermolecular Acylfluorination of Alkynes via a P(V) Intermediate. *J. Am. Chem. Soc.* 2020, **142**, 17323–17328.

4. T. Yoshii, S. Tsuziki, S. Sakurai, R. Sakamoto, J. Jiang, M. Hatanaka, A. Matsumoto, K. Maruoka, *N*-Hydroxybenzimidazole as a structurally modifiable platform for N-oxyl radicals for direct C–H functionalization reactions. *Chem. Sci.* 2020, **11**, 5772-5778.

5. C. A. Malapit, J. R. Bour, S. R. Laursen, M. S. Sanford, Mechanism and Scope of Nickel-Catalyzed Decarbonylative Borylation of Carboxylic Acid Fluorides. *J. Am. Chem. Soc.* 2019, **141**, 17322–17330.

6. D. Kim, H. N. Lim, Synthesis of Acyl Fluorides via DAST-Mediated Fluorinative C–C Bond Cleavage of Activated Ketones, *Org. Lett*. 2020, **22**, 7465–7469.

7. N. S. Y. Loy, S. Kim, C. Park, Synthesis of Unsymmetrical Pyrazines Based on α-Diazo Oxime Ethers. *Org. Lett.* 2015, **17**, 395–397.

8. Y. Ano, S. Takahashi, N. Chatani, Palladium-Catalyzed 1,1-Alkynyloxygenation of 2-Vinylbenzoates with Alkynyl Bromides. *Org. Lett.* 2023, **25**, 3266–3270.

9. L. Hu, Y. Liu, X. Fang, Y. Zheng, R. Liao, M. Li, Y. Xie, An Intermolecular Hydroarylation of Highly Deactivated Styrenes Catalyzed by Re2O7/HReO4 in Hexafluoroisopropanol. *ACS Catal.* 2022, **12**, 5857–5863.

10. C. Wang, S. Gong, Z. Liang, Y. Sun, R. Cheng, B. Yang, Y. Liu, J. Yang, F. Sun, Ligand-Promoted Iridium-Catalyzed Transfer Hydrogenation of Terminal Alkynes with Ethanol and Its Application. *ACS Omega* 2019, **4**, 16045–16051.

11. Z. Wu, S. N. Gockel, K. L. Hull, Anti-Markovnikov hydro(amino)alkylation of vinylarenes via photoredox catalysis. *Nat Commun* 2021, **12**, 5956.

12.X. Yan, W. Wu, L. Xu, Y. Liu, H. Shi, Ni-catalyzed hydroalkylation of olefins with N-sulfonyl amines. *Nat Commun* 2021, **12**, 5881.

13. Y. Zhang, G. Wang, Y. Wu, C. Zhu, P. Wang, Construction of α-Amino Azines via Thianthrenation-Enabled Photocatalyzed Hydroarylation of Azine-Substituted Enamides with Arenes, *Org. Lett.* 2021, **23**, 8522–8526.

14. A. Yokoyama, T. Maruyama, K. Tagami, H. Masu, K. Katagiri, I. Azumaya, T. Yokozawa, One-Pot Synthesis of Cyclic Triamides with a Triangular Cavity from trans-Stilbene and Diphenylacetylene Monomers. *Org. Lett.* 2008, **10**, 3207–3210.

15. I. Mandal, A. F. M. Kilbinger, Practical Route for Catalytic Ring-Opening Metathesis Polymerization. *JACS Au* 2022, **2**, 2800–2808.

16. F. Scheidt, M. Schäfer, J. C. Sarie, C. G. Daniliuc, J. J. Molloy, R. Gilmour, Enantioselective, Catalytic Vicinal Difluorination of Alkenes. *Angew. Chem. Int. Ed.* 2018, **57**, 16431–16435.

17. X. Ma, Y. Liu, P. Liu, J. Xie, B. Dai, Z. Liu, A rapid and efficient catalysis system for the synthesis of 4-vinylbiphenyl derivatives. *Appl. Organometal. Chem*. 2013, **27**, 707–710.

18. C. Lei, Y. J. Yip. J. S. Zhou, Nickel-Catalyzed Direct Synthesis of Aryl Olefins from Ketones and Organoboron Reagents under Neutral Conditions. *J. Am. Chem. Soc.* 2017, **139**, 6086–6089.

19. R. Shishido, M. Uesugi, R. Takahashi, T. Mita, T. Ishiyama, K. Kubota, H. Ito, General Synthesis of Trialkyl- and Dialkylarylsilylboranes: Versatile Silicon Nucleophiles in Organic Synthesis, *J. Am. Chem. Soc.* 2020, **142**, 14125–14133.

20. D. Ni, M. K. Brown, Three-Component Ni-Catalyzed Silylacylation of Alkenes. *ACS Catal.* 2021, **11**, 1858–1862.

21. H. Xu, W. Zheng, W. D. Liu, Y. Zhou, L. Lin and J. Zhao, Silylacylation of Alkenes through N-heterocyclic carbene catalysis. *Org. Lett.* 2023, **25**, 5579-5584.

22. N. Marion, E. C. Ecarnot, O. Navarro, D. Amoroso, A. Bell and S. P. Nolan, (IPr)Pd(acac)Cl: An Easily Synthesized, Efficient, and Versatile Precatalyst for C−N and C−C Bond Formation. *J. Org. Chem.* 2006, **71**, 3816-3821.

23. A. J. Cresswell, S. G. Davies, A. L. Figuccia, A. M. Fletcher, D. Heijnen, J. A. Lee, M. J. Morris, A. M. R. Kennett, P. M. Roberts, J. E. Thomson, Pinacolatoboron fluoride (pinBF) is an efficient fluoride transfer agent for diastereoselective synthesis of benzylic fluorides. *Tetrahedron Letters*, 2015, **56**, 3373-3377.

11. NMR Data (^{1}H NMR, ^{13}C NMR and ^{19}F NMR)

methyl 2-(3-(dimethyl(phenyl)silyl)-1-oxo-1-phenylpropan-2-yl)benzoate (3ab)

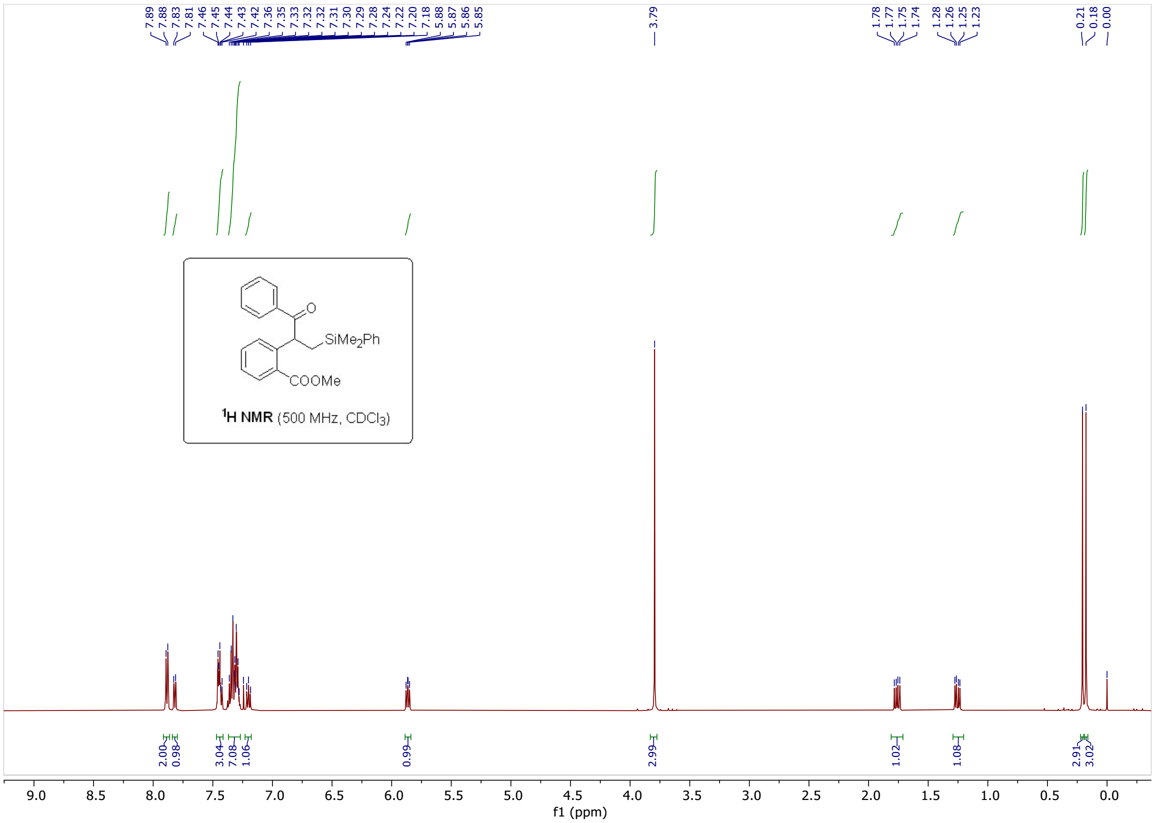

SiMe2Ph
COOMe
1H NMR (500 MHz, CDCl3)
7.89
7.88
7.83
7.81
7.46
7.45
7.44
7.43
7.42
7.36
7.35
7.33
7.32
7.32
7.31
7.30
7.29
7.28
7.24
7.22
7.20
7.18
5.88
5.87
5.86
5.85
3.79
1.78
1.77
1.75
1.74
1.28
1.26
1.25
1.23
0.21
0.18
0.00
2.00
0.98
3.04
7.08
1.06
0.99
2.99
1.02
1.08
2.91
3.02
f1 (ppm)

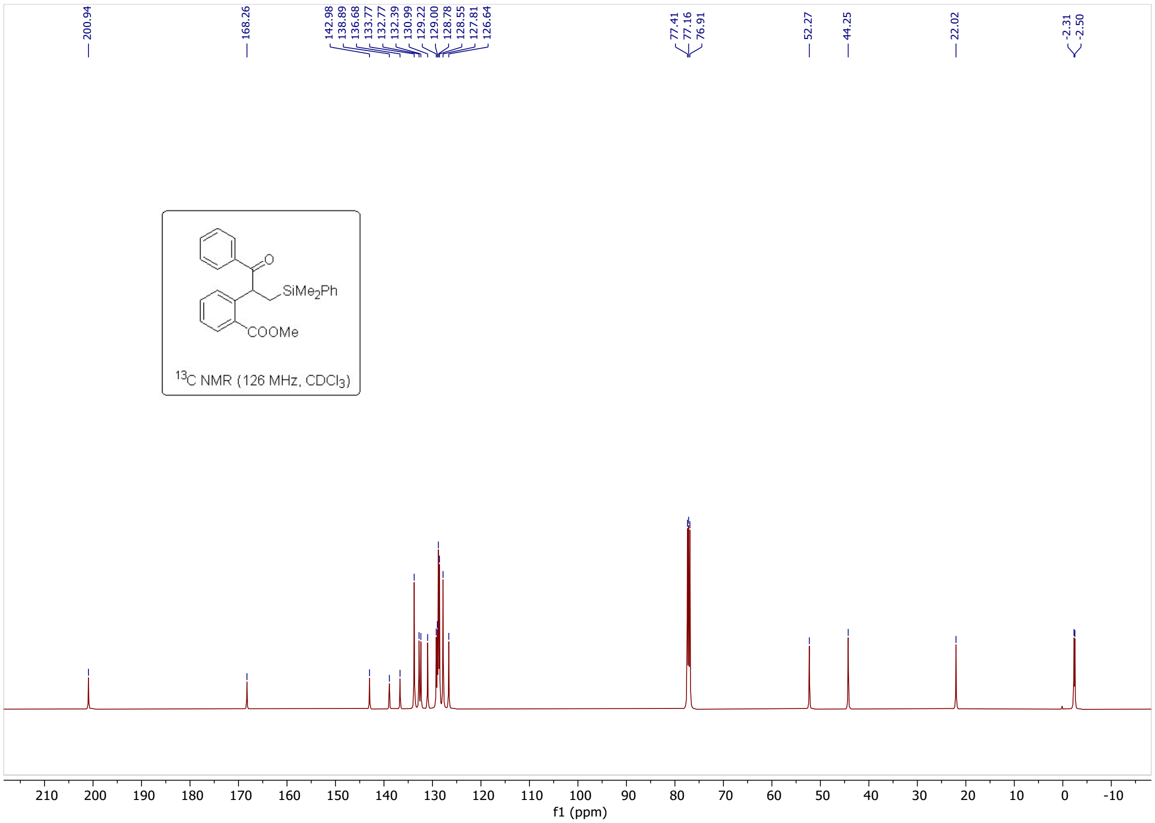

SiMe2Ph
COOMe
13C NMR (126 MHz, CDCl3)
200.94
168.26
142.98
138.89
136.68
133.77
132.77
132.39
130.99
129.22
129.00
128.78
128.55
127.81
126.64
77.41
77.16
76.91
52.27
44.25
22.02
-2.31
-2.50
f1 (ppm)

phenyl 4-(3-(dimethyl(phenyl)silyl)-1-oxo-1-phenylpropan-2-yl)benzoate (3ac)

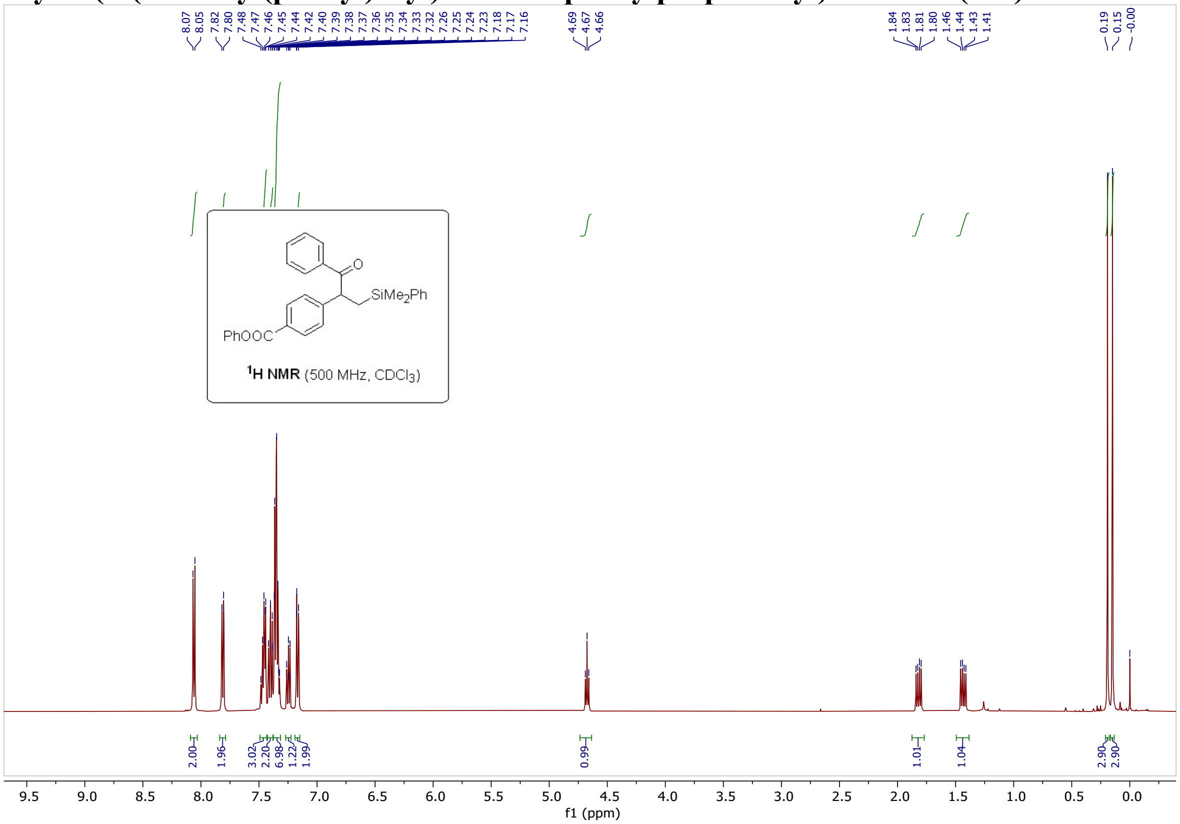

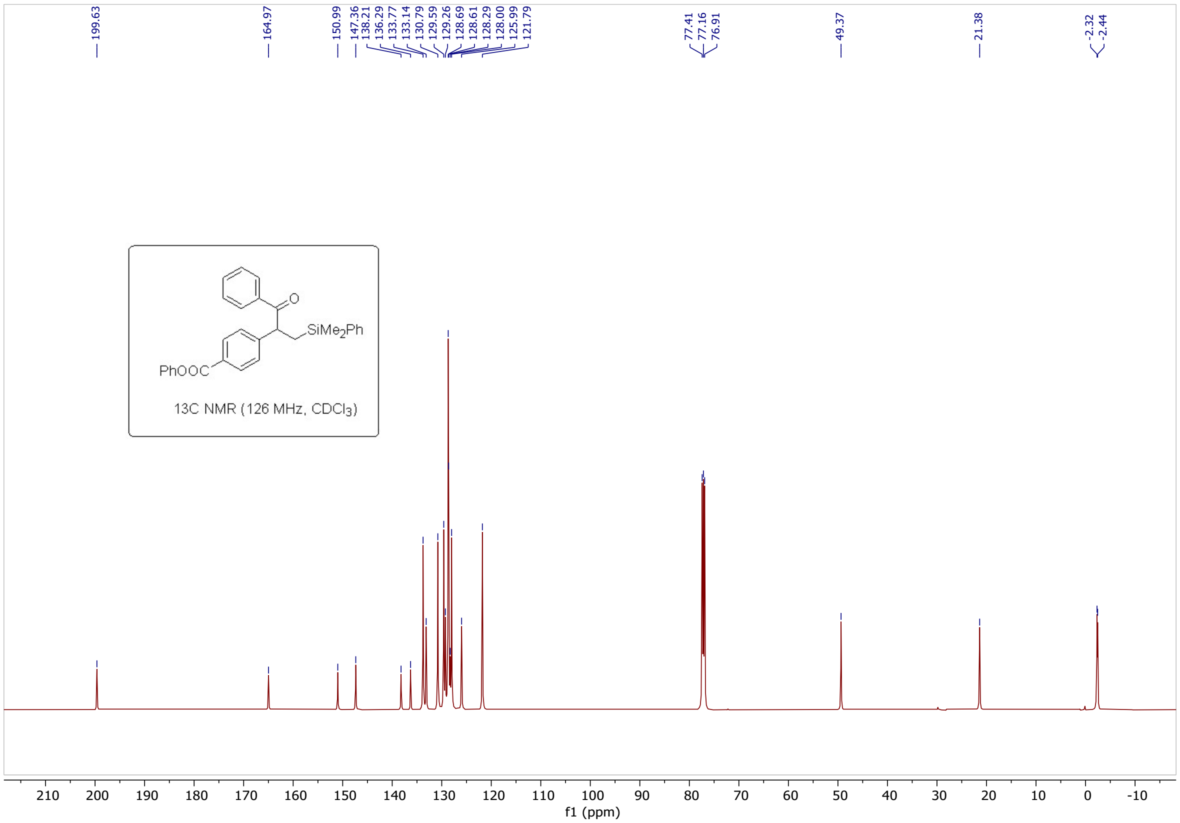

3-(dimethyl(phenyl)silyl)-1-phenyl-2-(4-(trifluoromethyl)phenyl)propan-1-one (3ad)

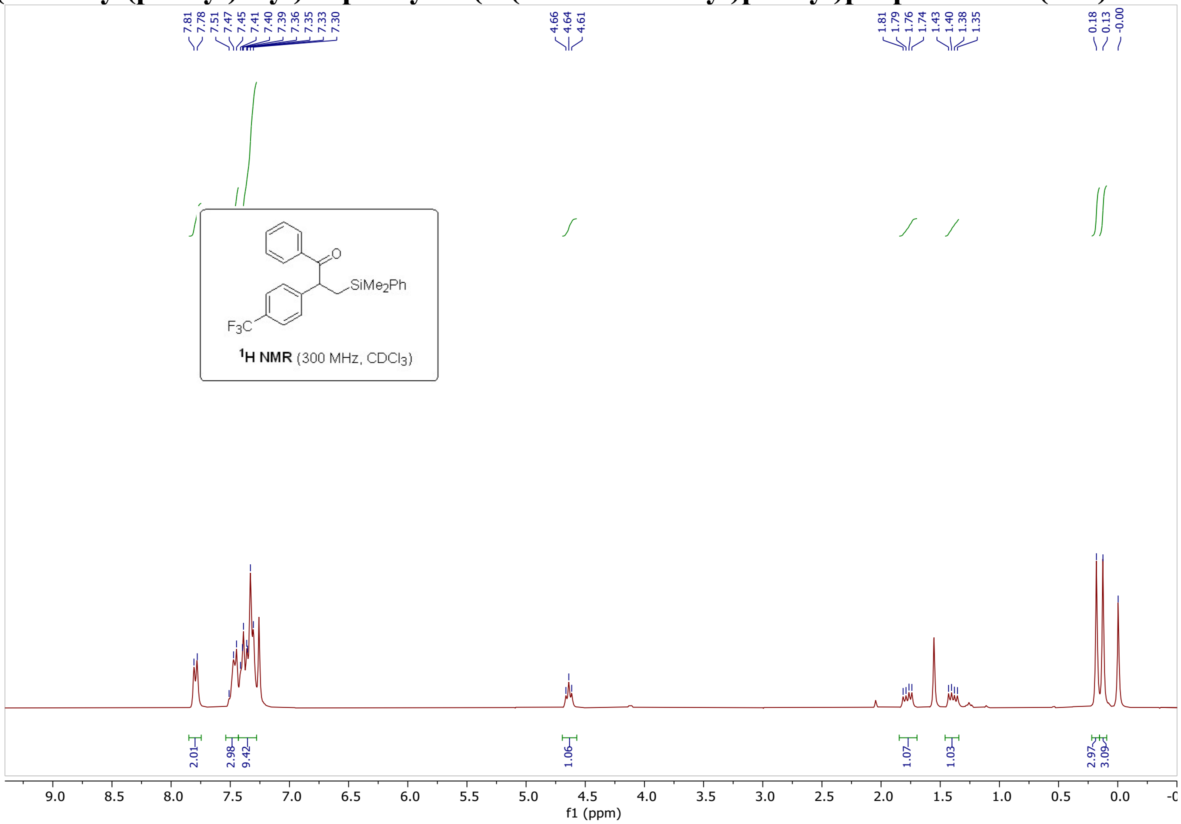

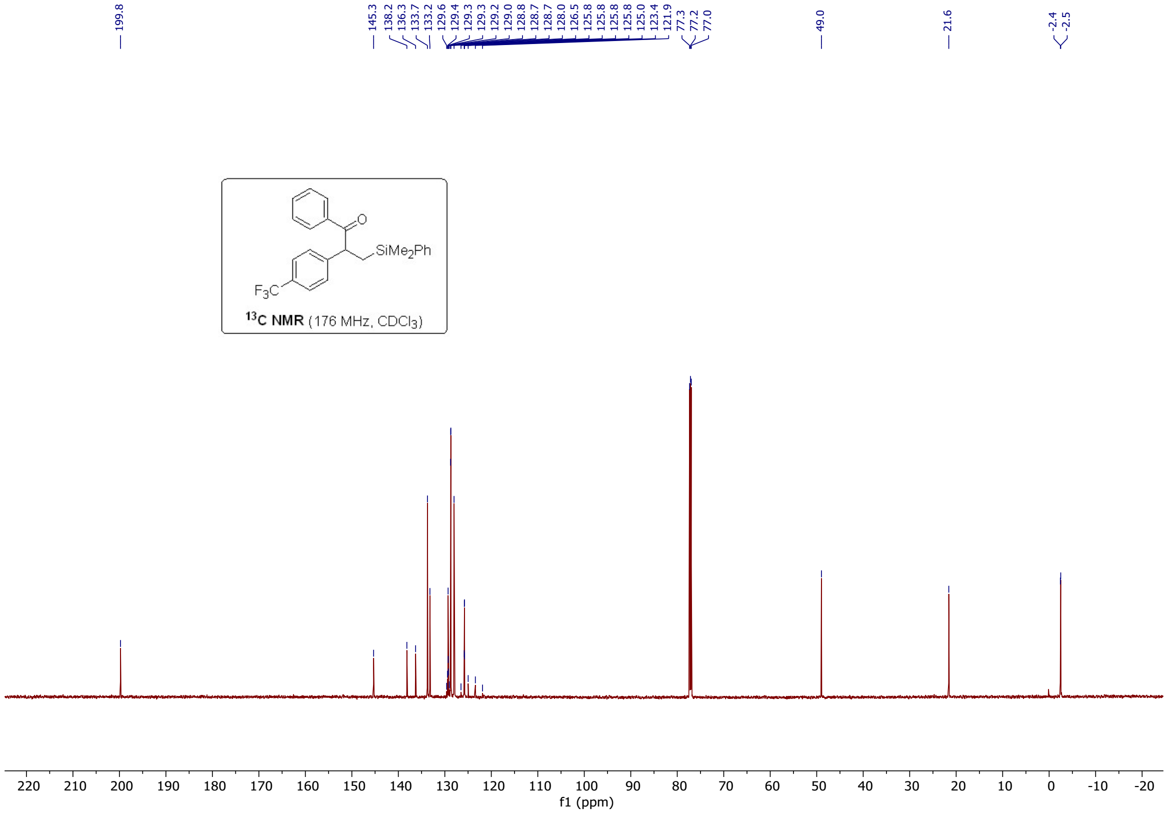

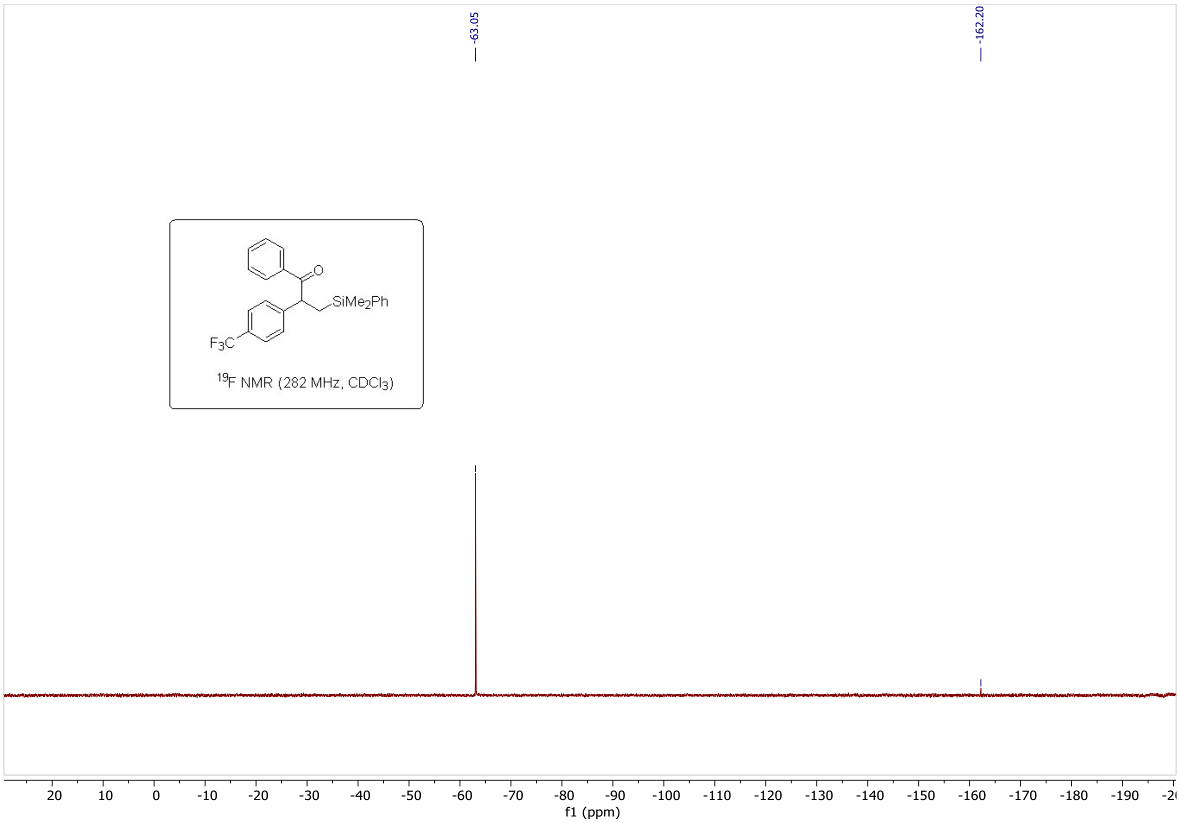

4-(3-(dimethyl(phenyl)silyl)-1-oxo-1-phenylpropan-2-yl)benzonitrile (3ae)

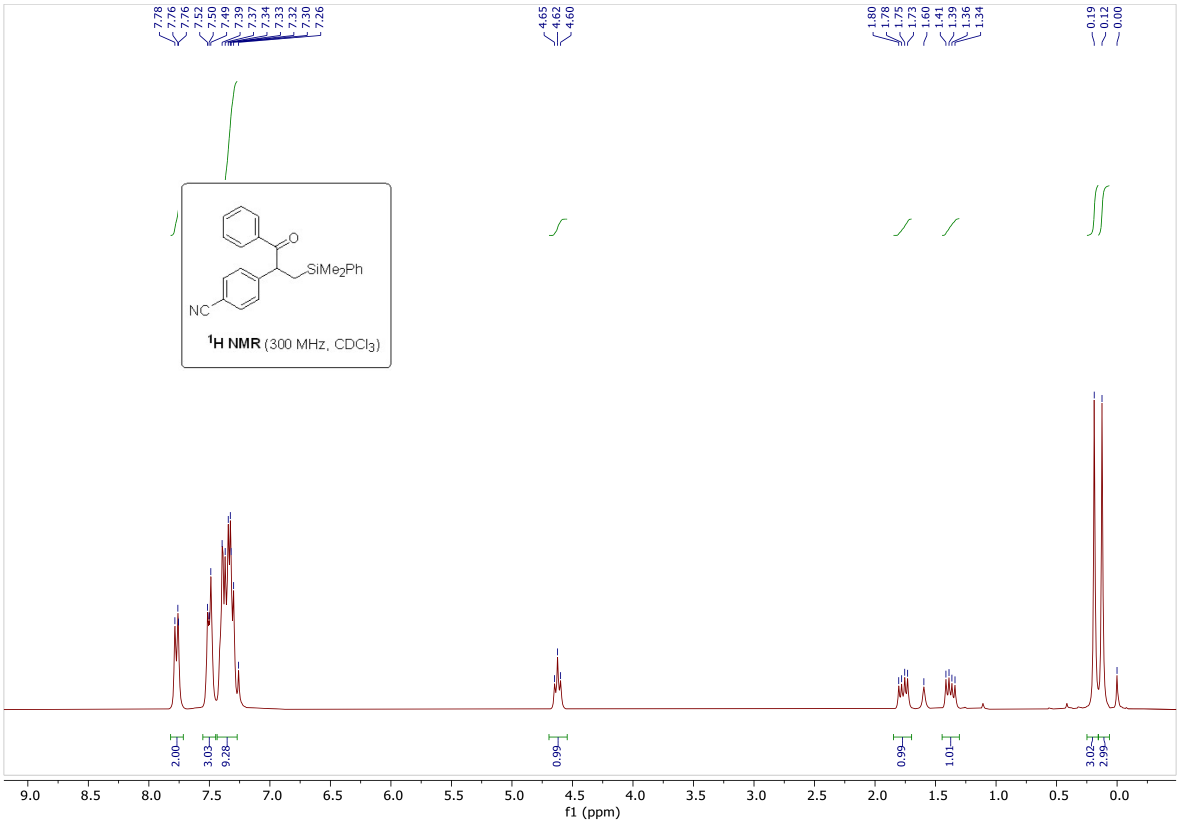

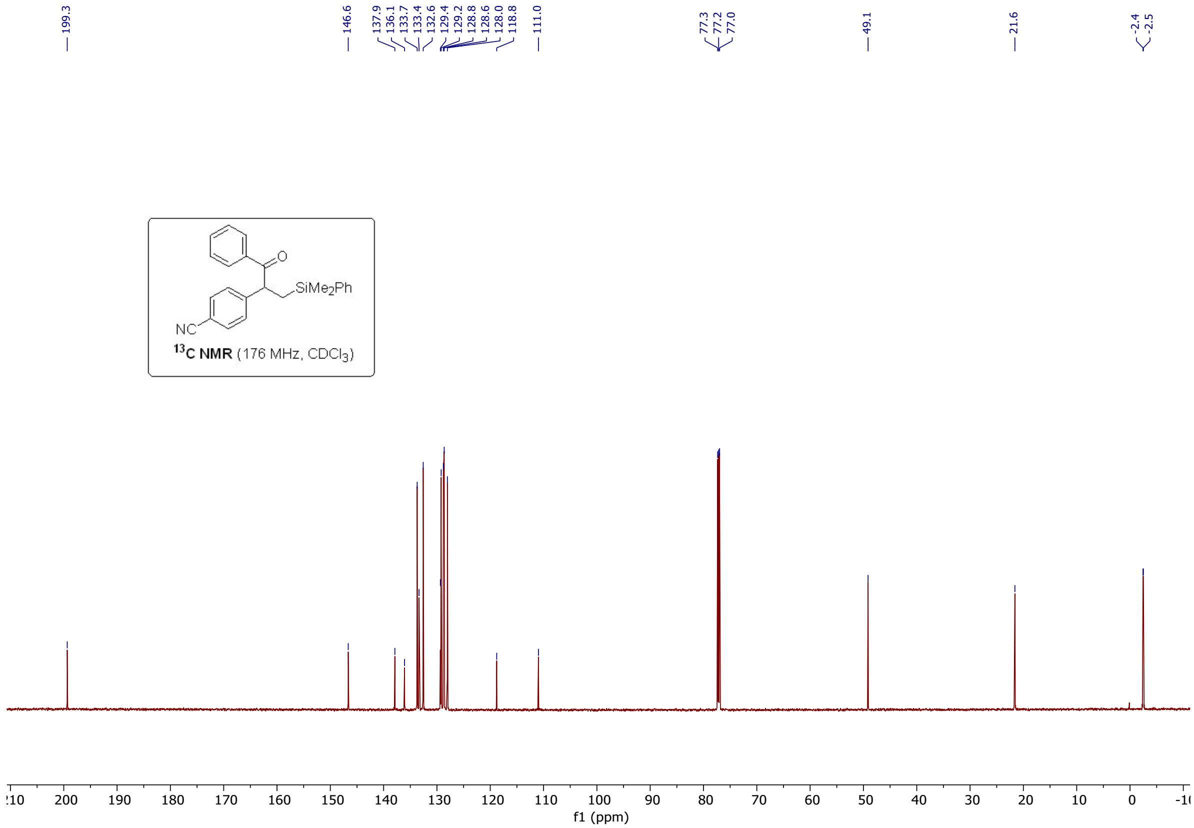

3-(dimethyl(phenyl)silyl)-1-phenyl-2-(4-((trifluoromethyl)sulfonyl)phenyl)propan-1-one (3af)

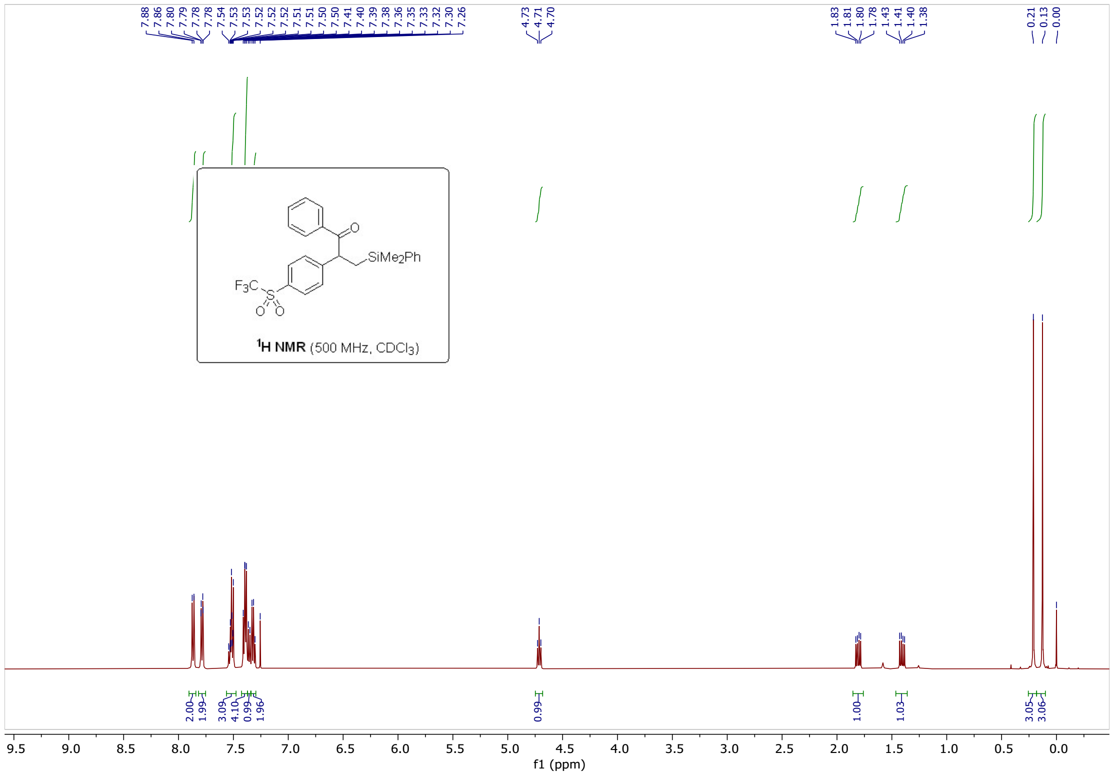

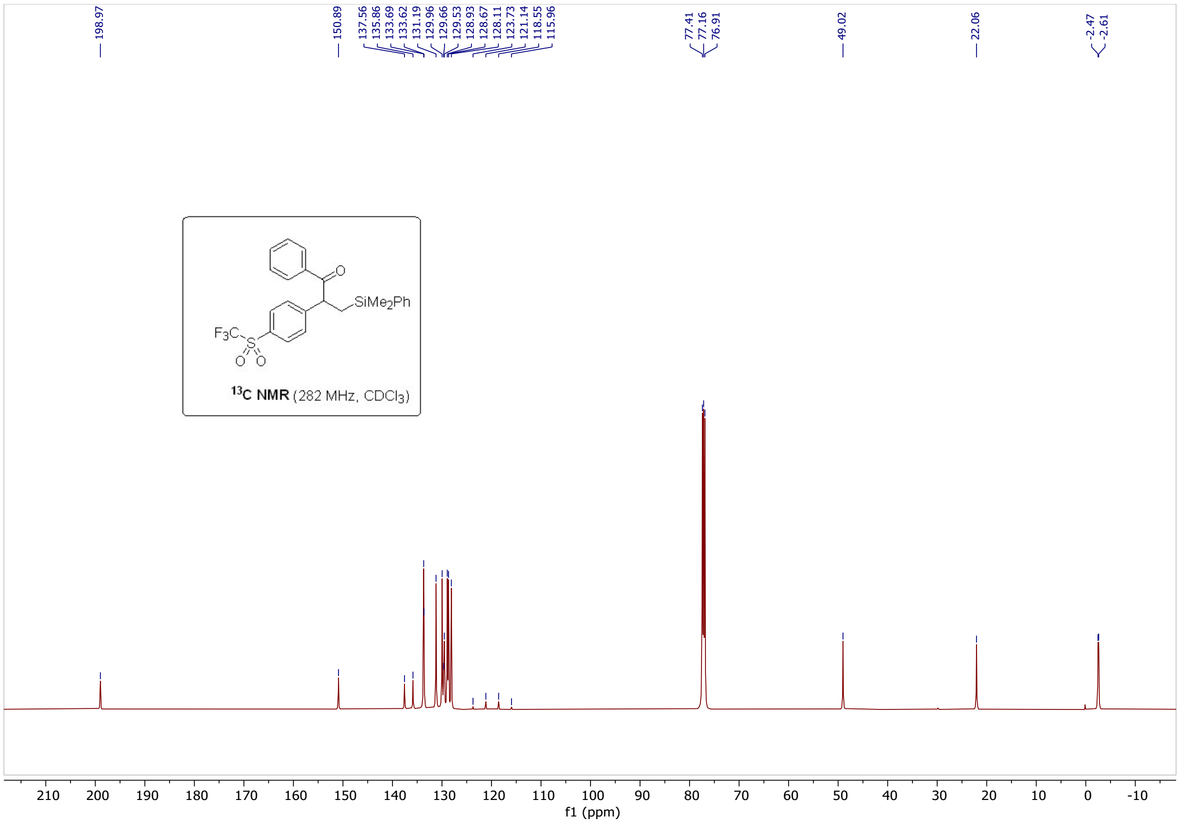

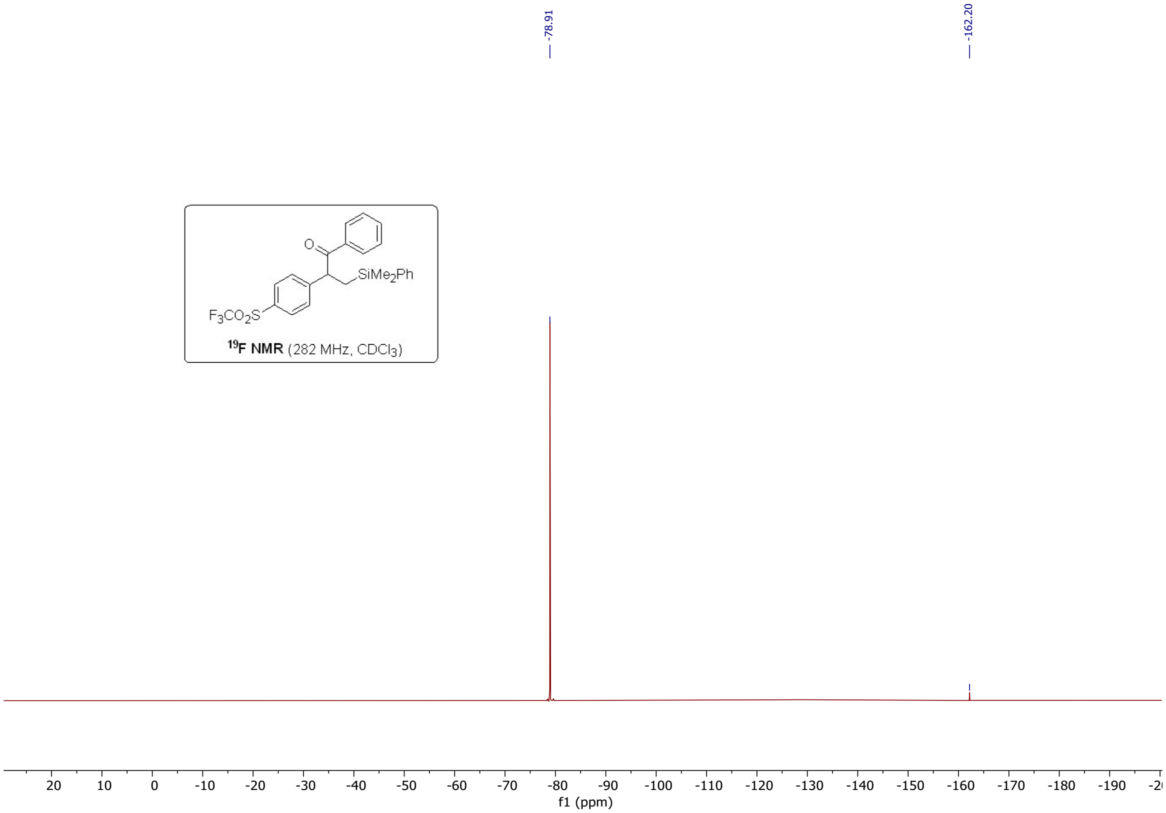

2-(3,5-bis(trifluoromethyl)phenyl)-3-(dimethyl(phenyl)silyl)-1-phenylpropan-1-one (3ag)

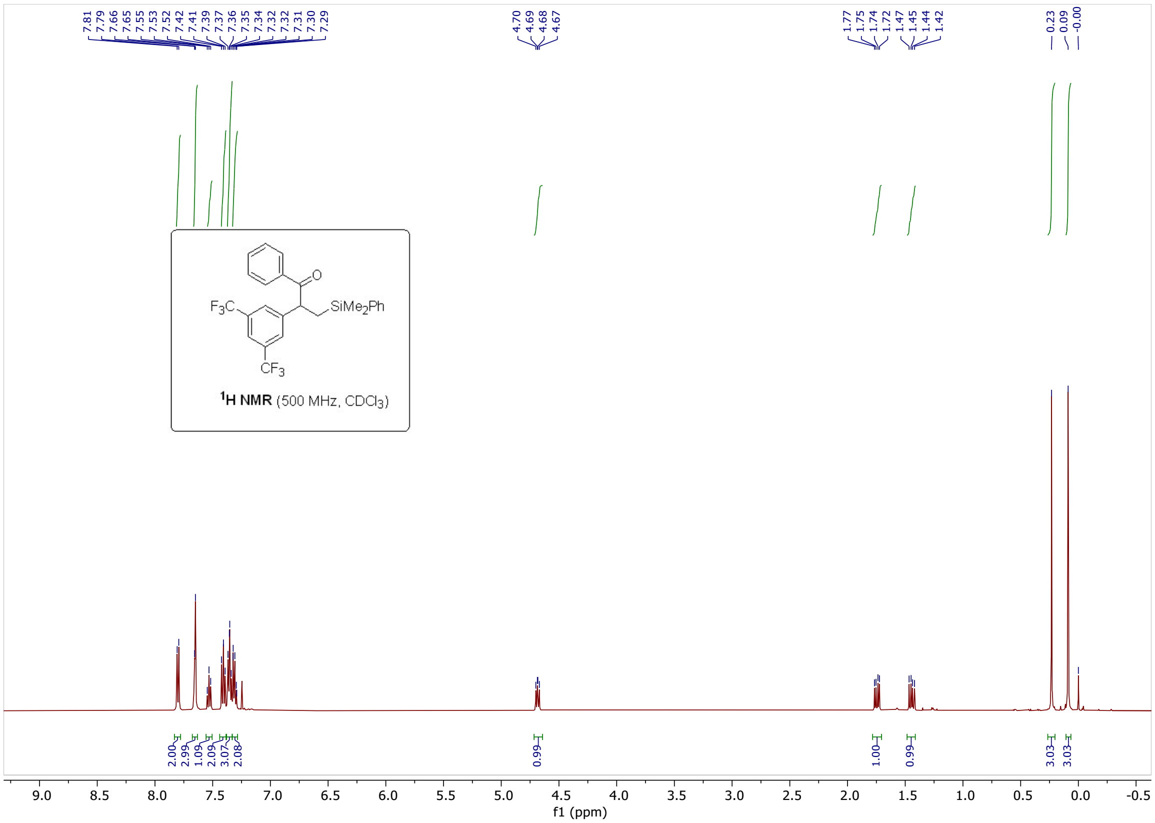

7.81
7.79
7.66
7.65
7.55
7.53
7.52
7.42
7.41
7.39
7.37
7.36
7.35
7.34
7.32
7.32
7.31
7.30
7.29
4.70
4.69
4.68
4.67
1.77
1.75
1.74
1.72
1.47
1.45
1.44
1.42
0.23
0.09
-0.00
F3C
SiMe2Ph
CF3
O
1H NMR (500 MHz, CDCl3)
2.00
2.99
1.09
2.09
3.07
2.08
0.99
1.00
0.99
3.03
3.03
f1 (ppm)

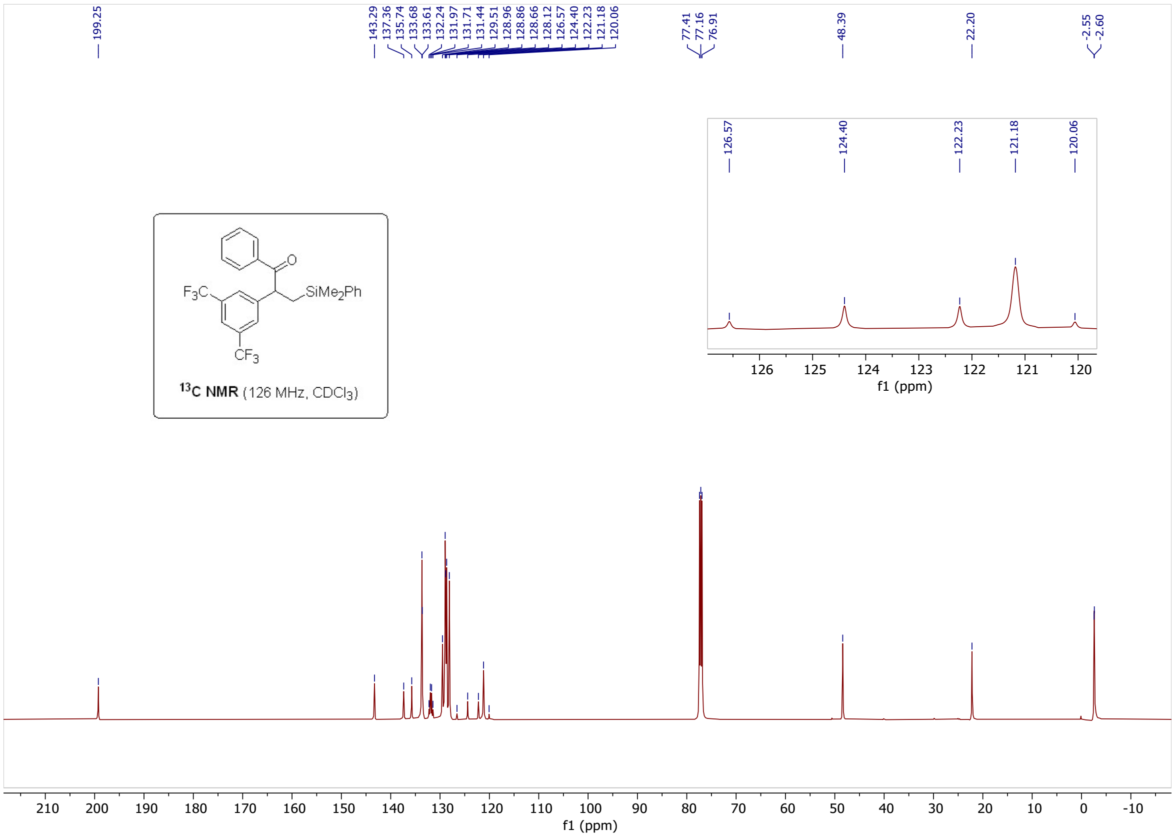

199.25
143.29
137.36
135.74
133.68
133.61
132.24
131.97
131.71
131.44
129.51
128.96
128.86
128.66
128.12
126.57
124.40
122.23
121.18
120.06
77.41
77.16
76.91
48.39
22.20
-2.55
-2.60
F3C
SiMe2Ph
CF3
O
13C NMR (126 MHz, CDCl3)
f1 (ppm)

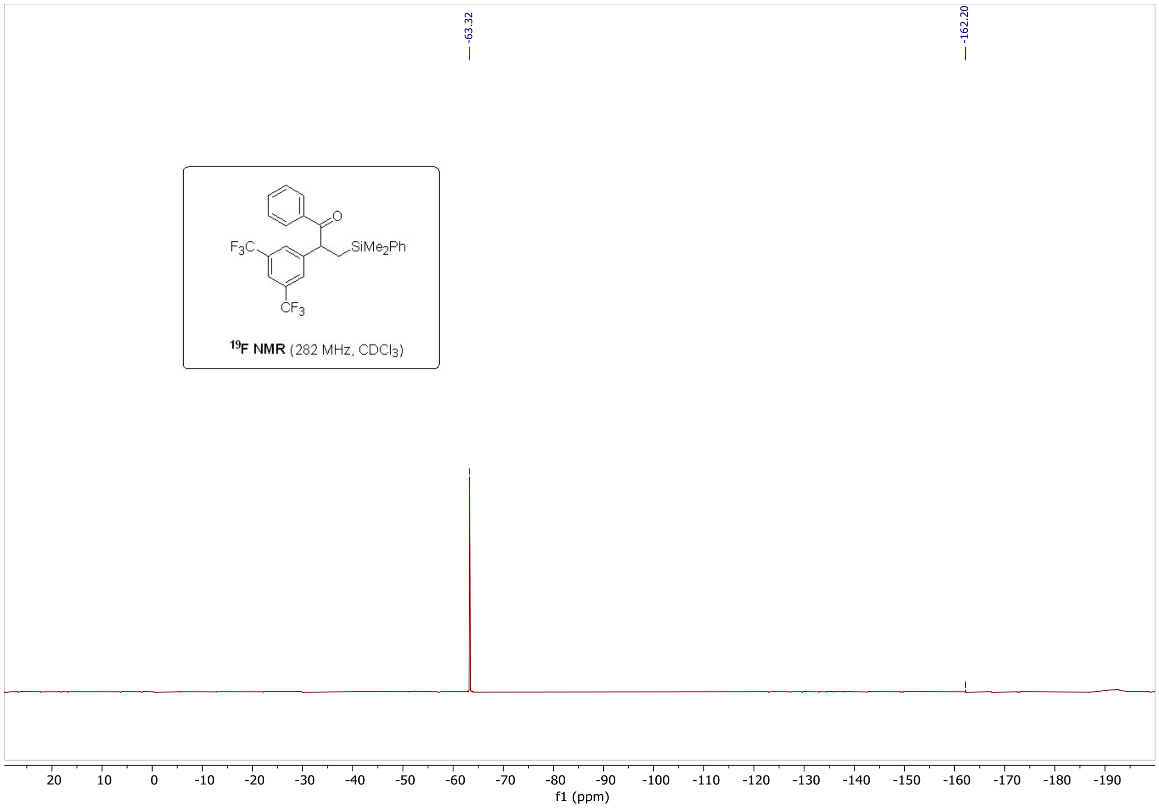

3-(dimethyl(phenyl)silyl)-2-(perfluorophenyl)-1-phenylpropan-1-one (3ah)

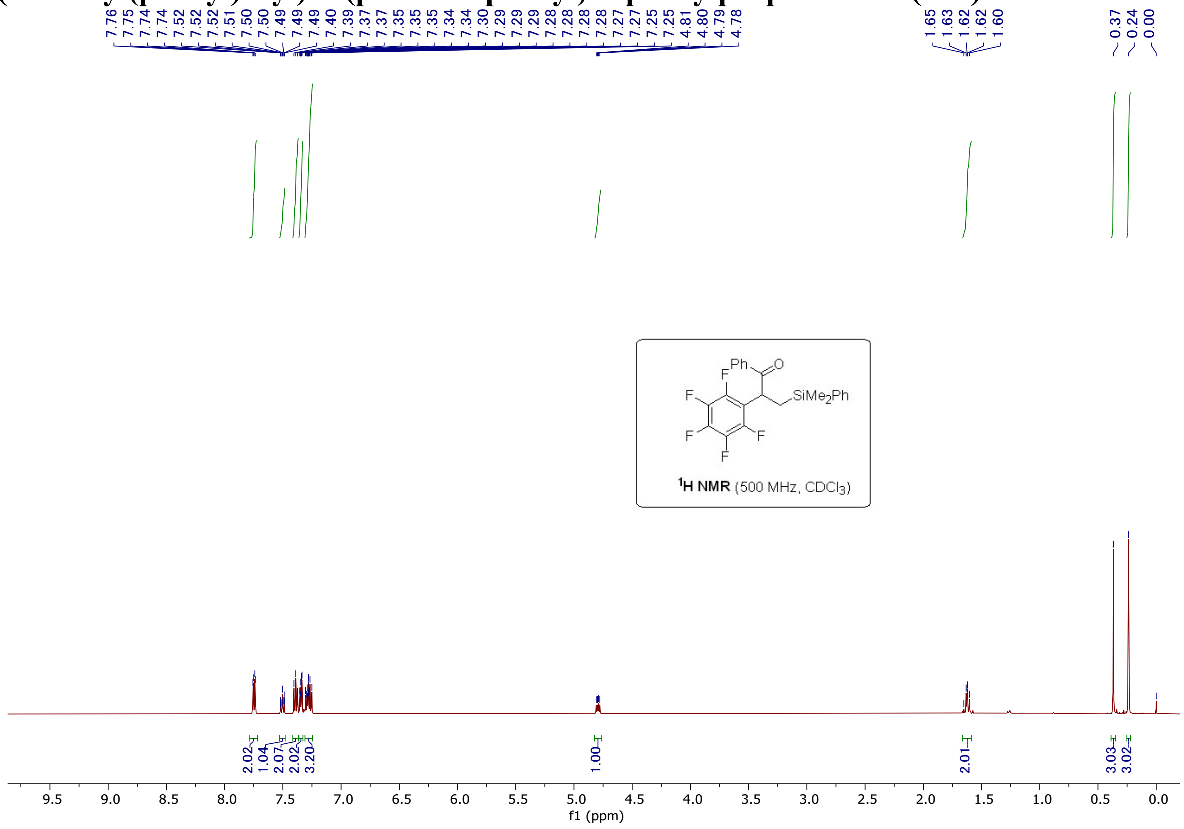

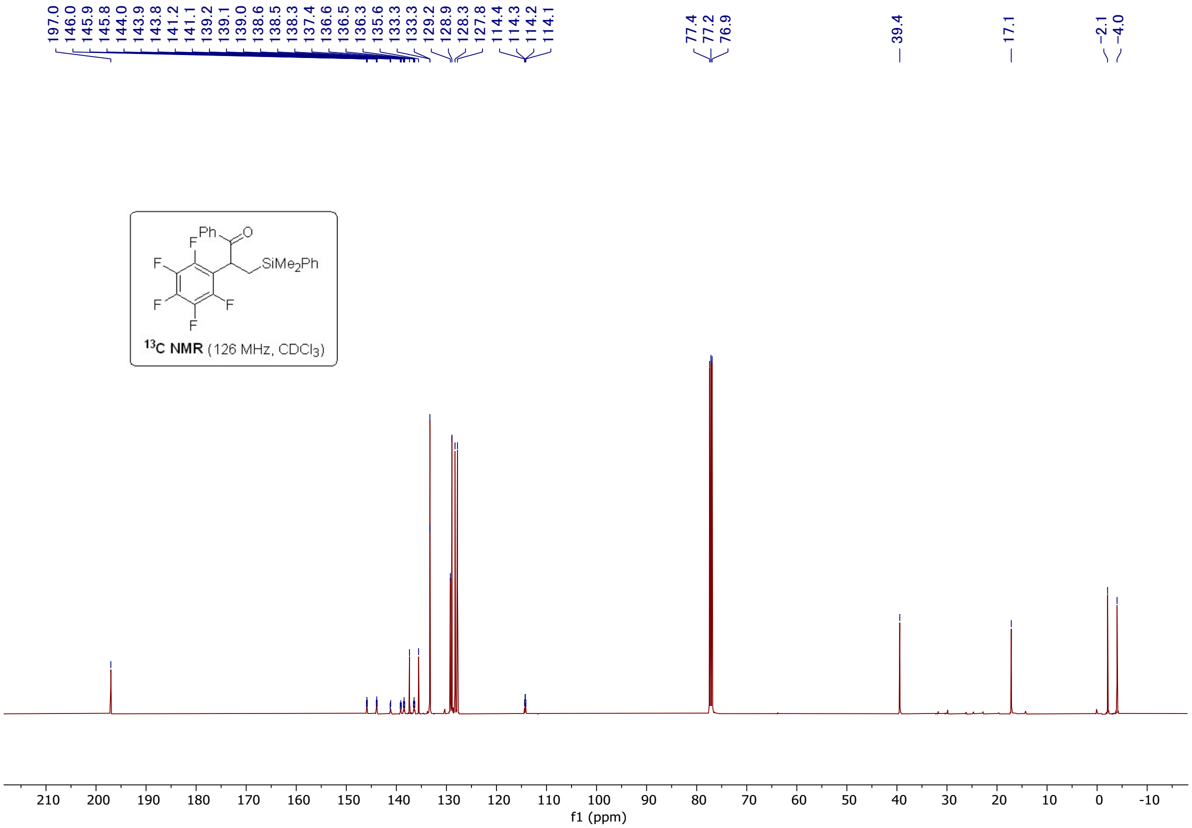
197.0
146.0
145.9
145.8
144.0
143.9
143.8
141.2
141.1
139.2
139.1
139.0
138.6
138.5
138.3
137.4
136.6
136.5
136.3
135.6
133.3
133.3
133.3
129.2
128.9
128.3
127.8
114.4
114.3
114.2
114.1
77.4
77.2
76.9
39.4
17.1
−2.1
−4.0
Ph
O
F
F
SiMe2Ph
F
F
F
F
13C NMR (126 MHz, CDCl3)
210
200
190
180
170
160
150
140
130
120
110
100
90
80
70
60
50
40
30
20
10
0
-10
f1 (ppm)

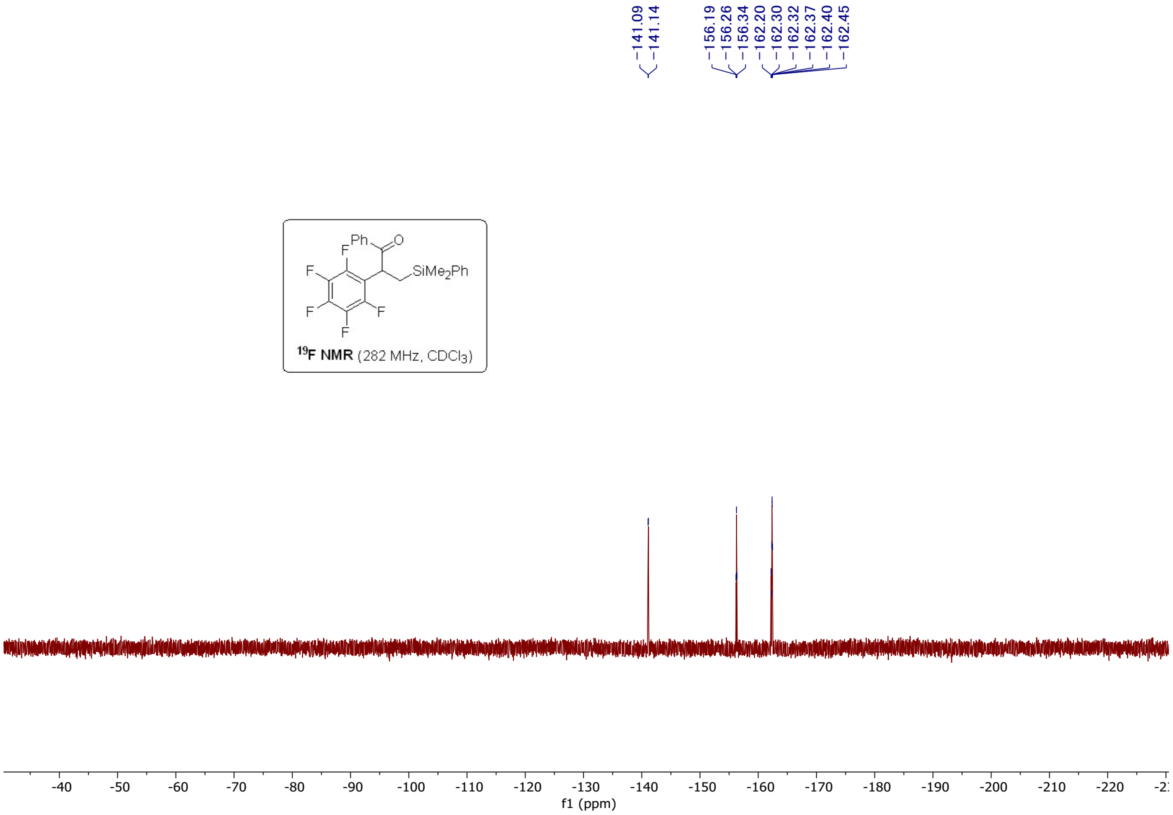
−141.09
−141.14
−156.19
−156.26
−156.34
−162.20
−162.30
−162.32
−162.37
−162.40
−162.45
Ph
O
F
F
SiMe2Ph
F
F
F
F
19F NMR (282 MHz, CDCl3)
-40
-50
-60
-70
-80
-90
-100
-110
-120
-130
-140
-150
-160
-170
-180
-190
-200
-210
-220
f1 (ppm)

3-(dimethyl(phenyl)silyl)-1-phenyl-2-(4'-(trifluoromethyl)-[1,1'-biphenyl]-4-yl)propan-1-one (3ai)

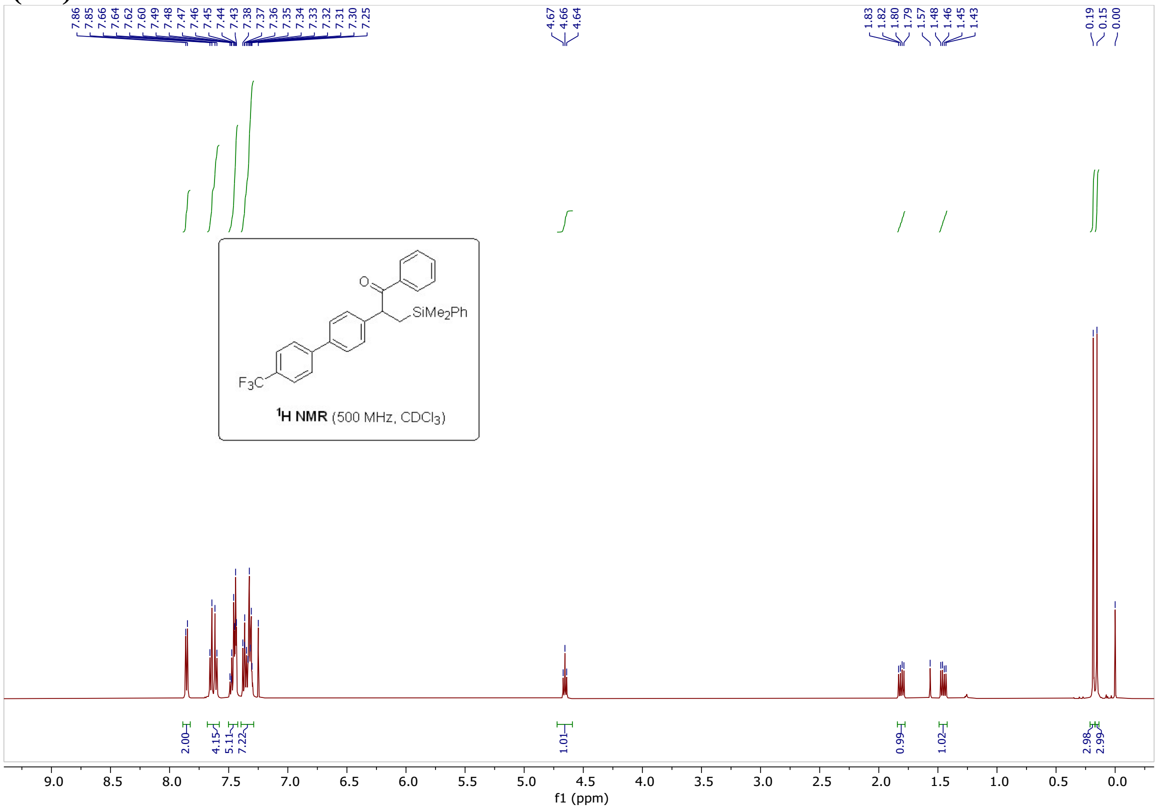

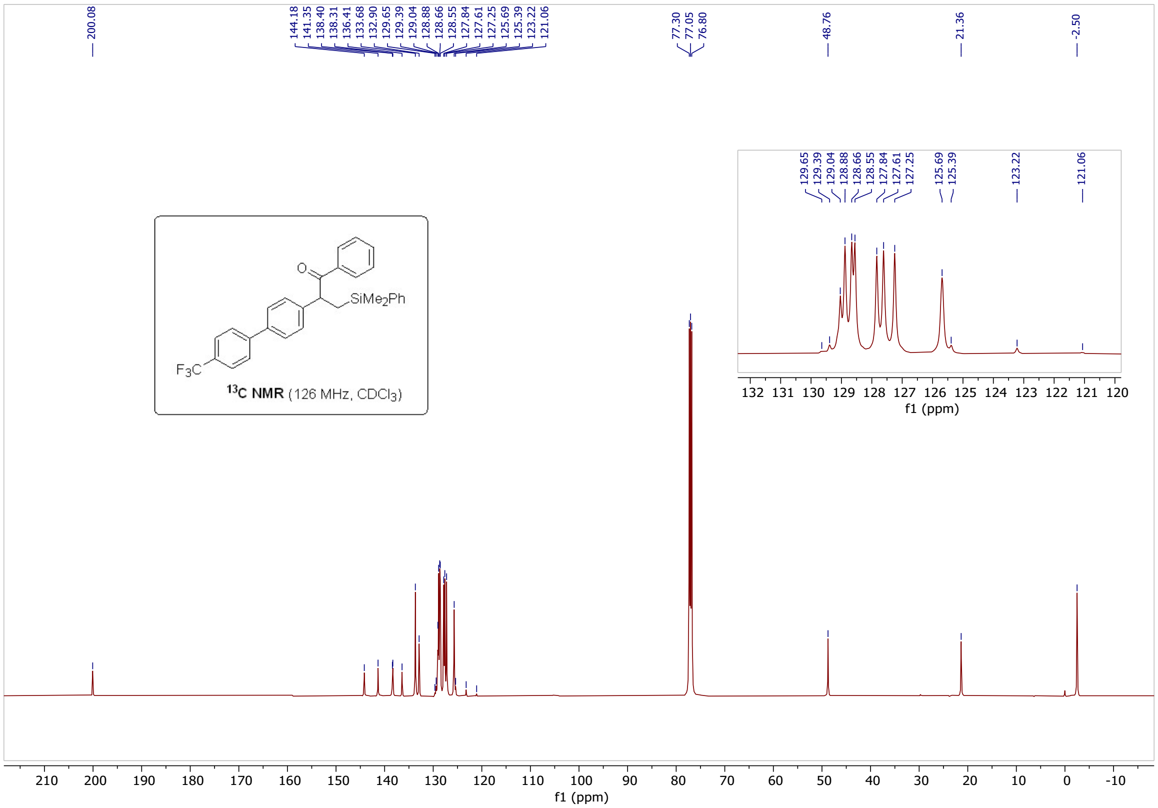

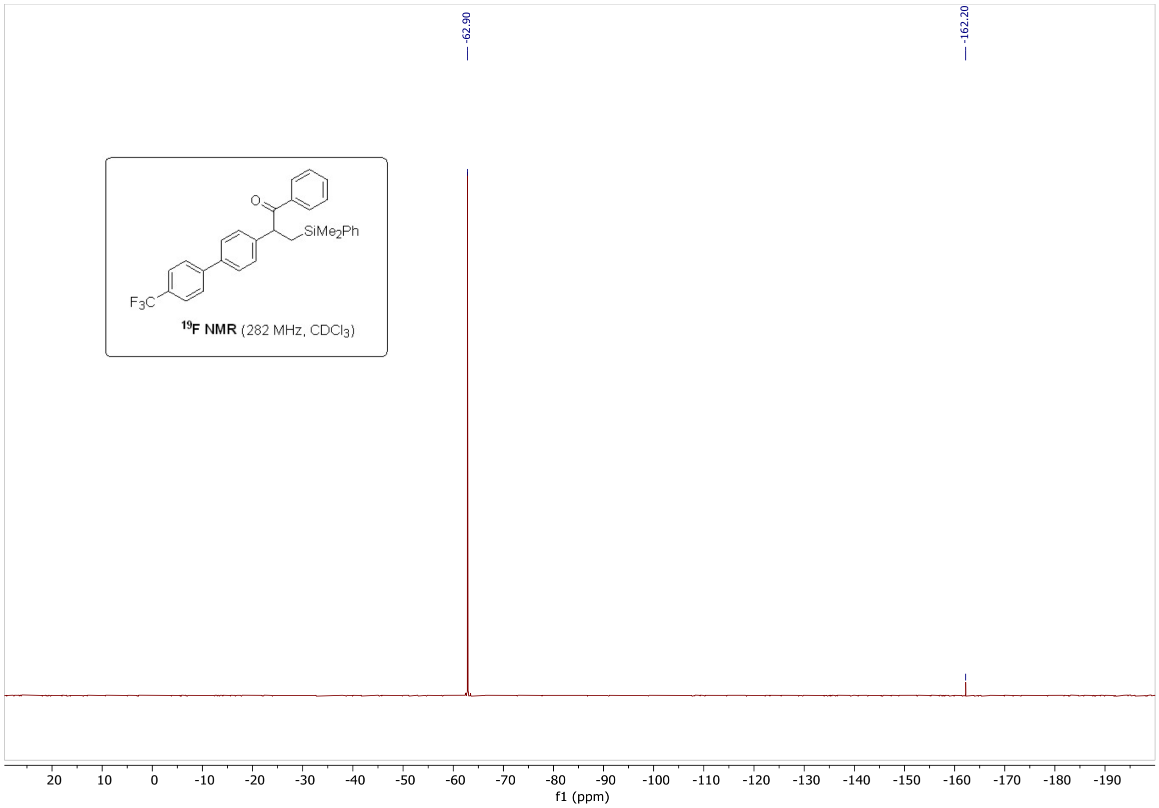

methyl 4-(3-(dimethyl(phenyl)silyl)-1-oxo-1,2-diphenylpropan-2-yl)benzoate (3aj)

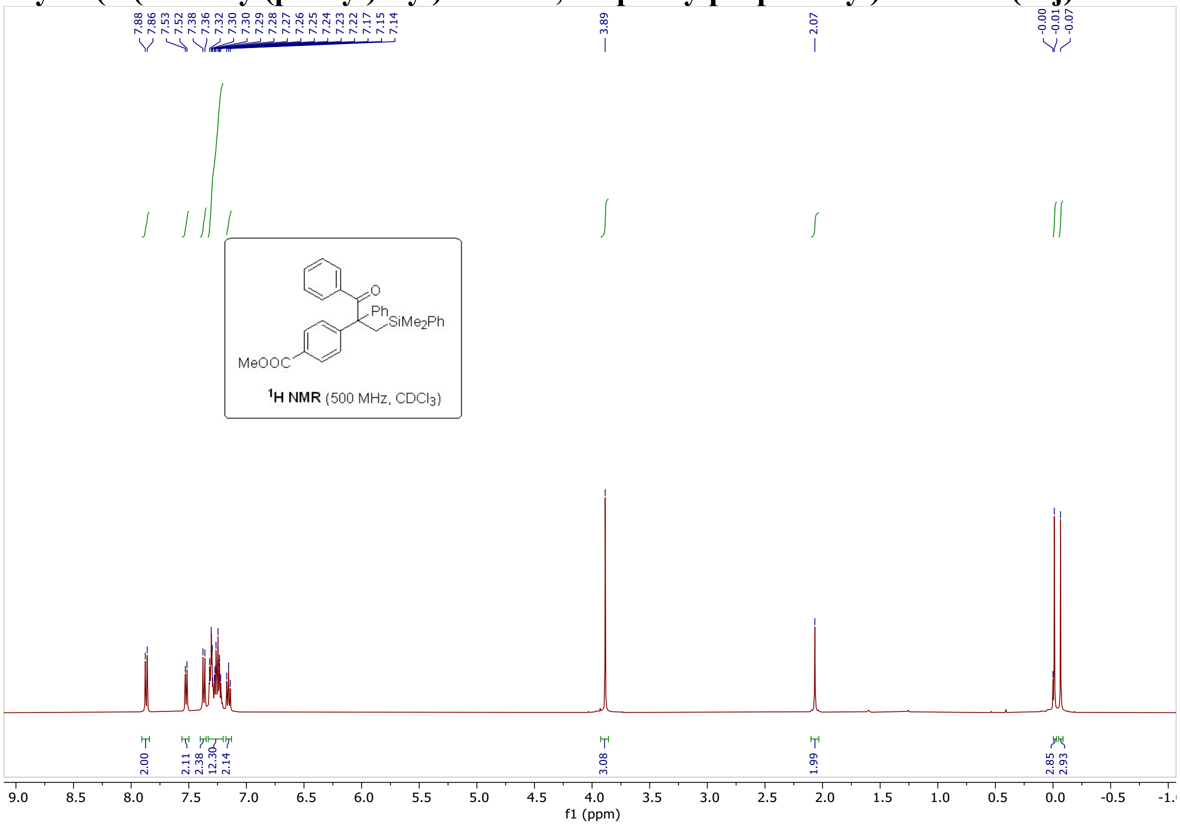

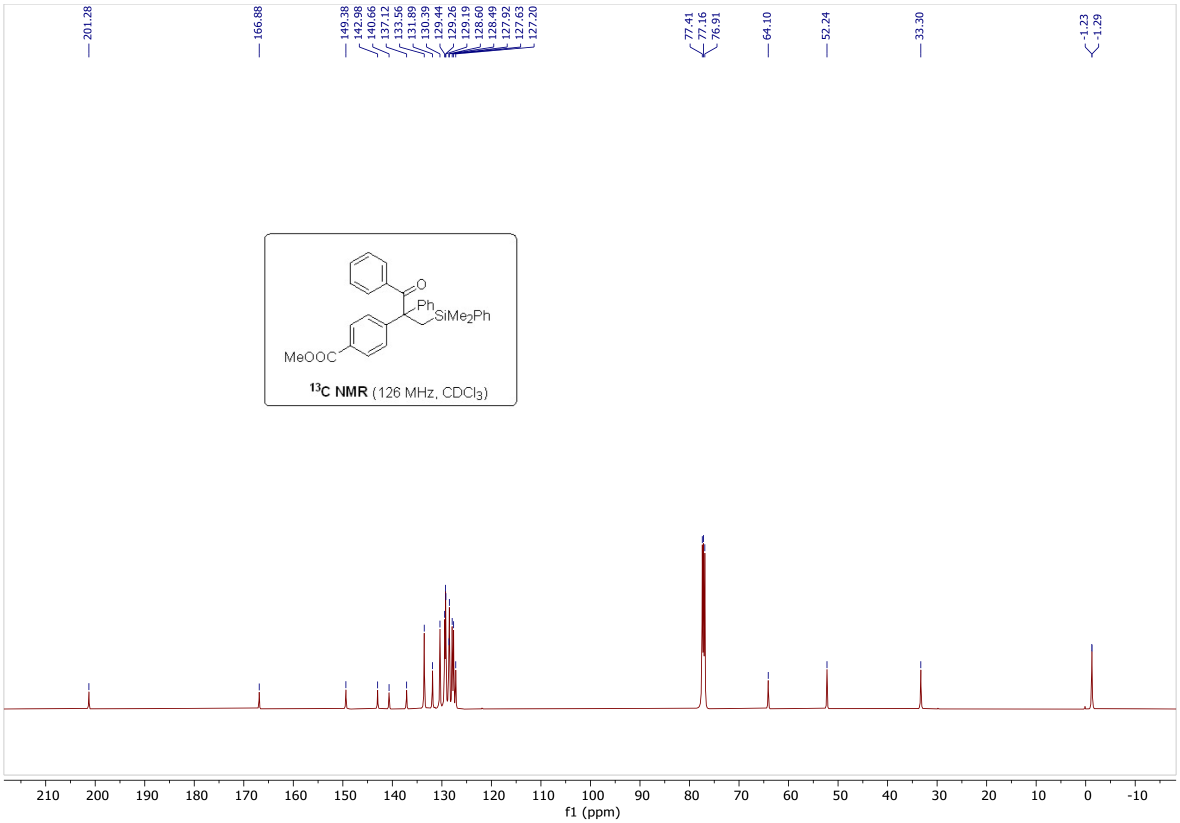

methyl 4-(3-(dimethyl(phenyl)silyl)-2-methyl-1-oxo-1-phenylpropan-2-yl)benzoate (3ak)

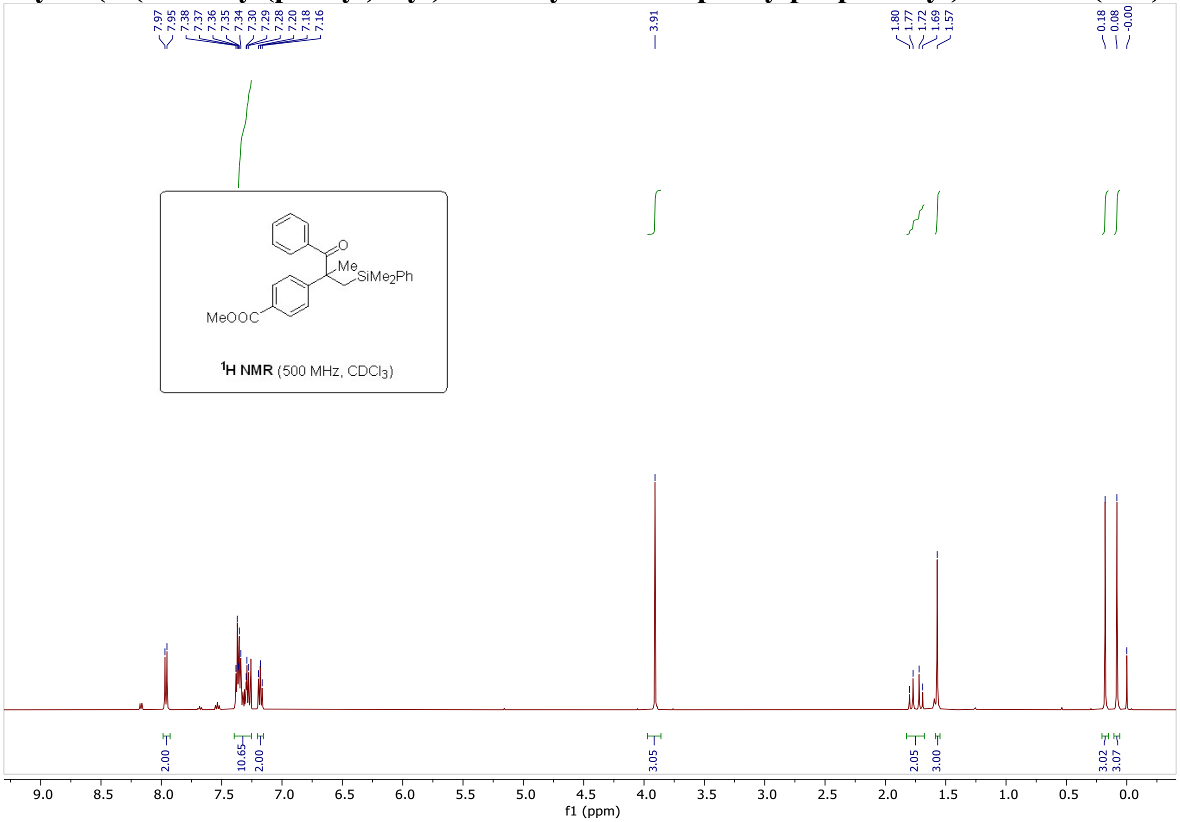

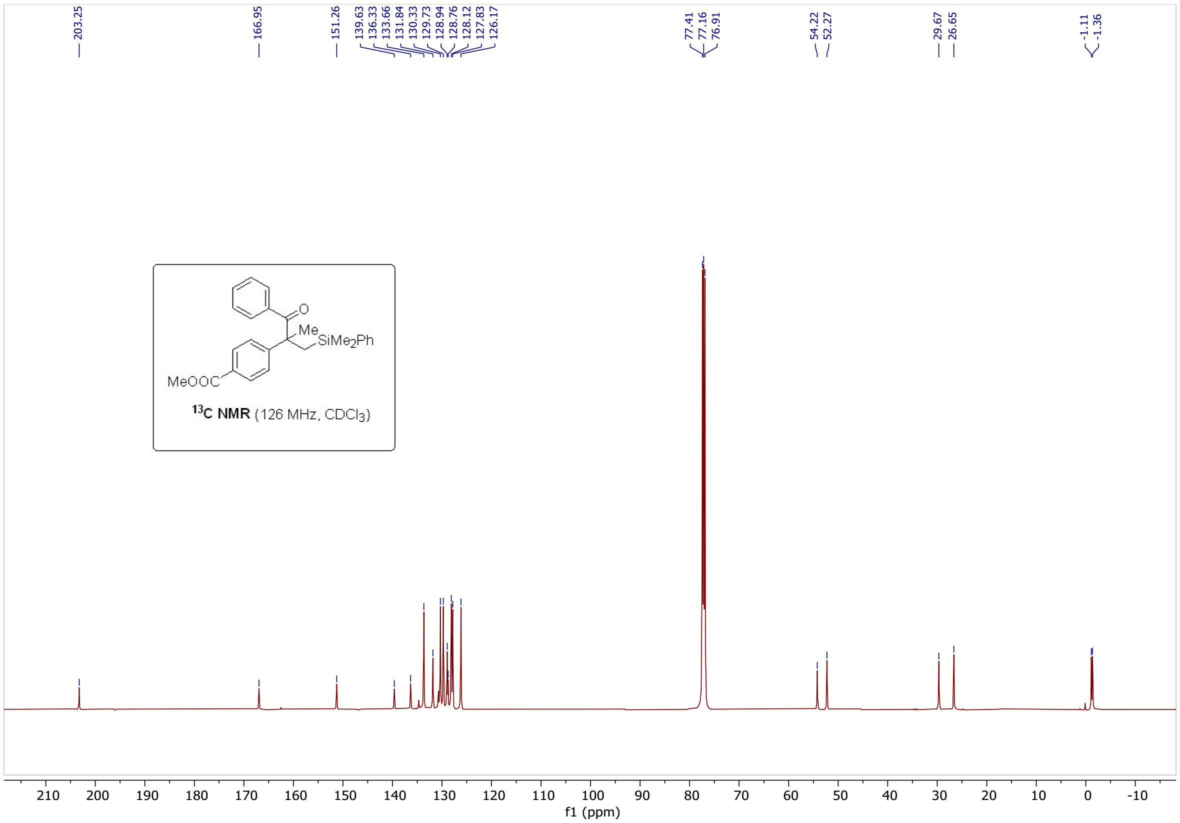

3-(dimethyl(phenyl)silyl)-1-phenyl-2-(pyridin-2-yl)propan-1-one (3al)

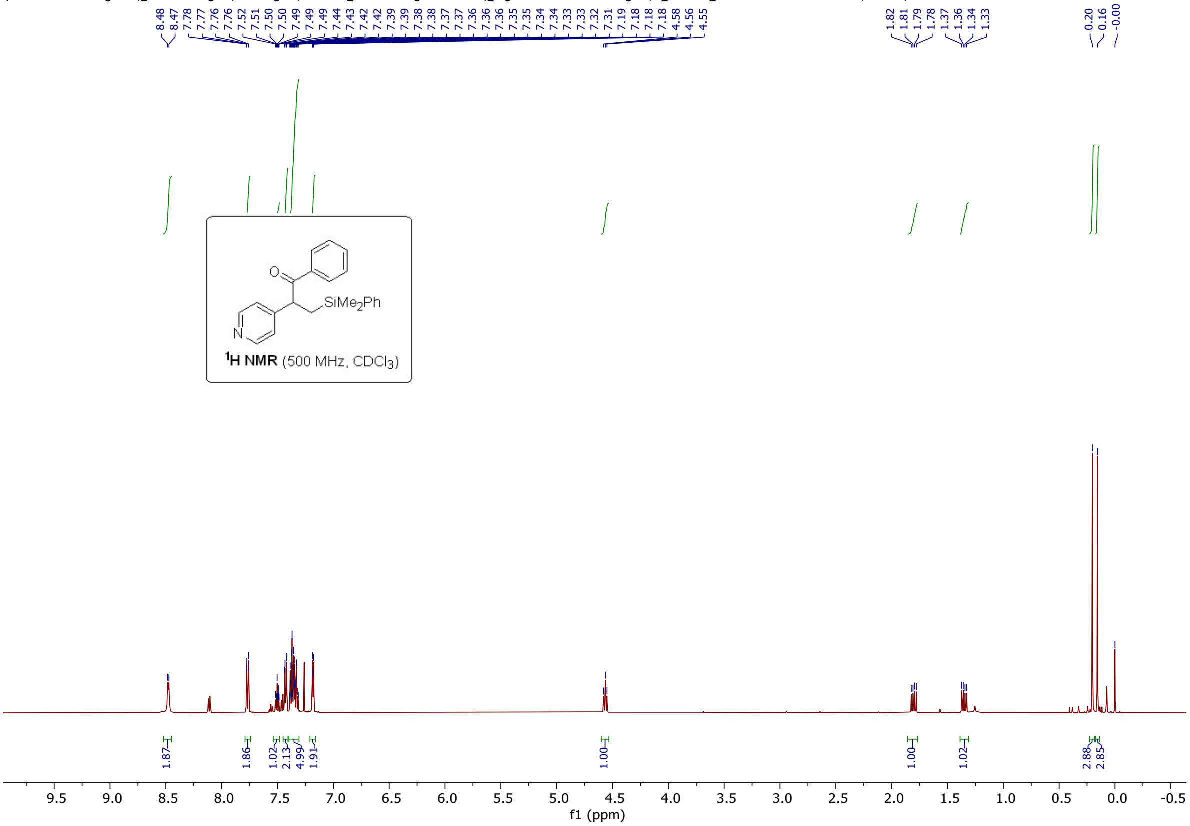

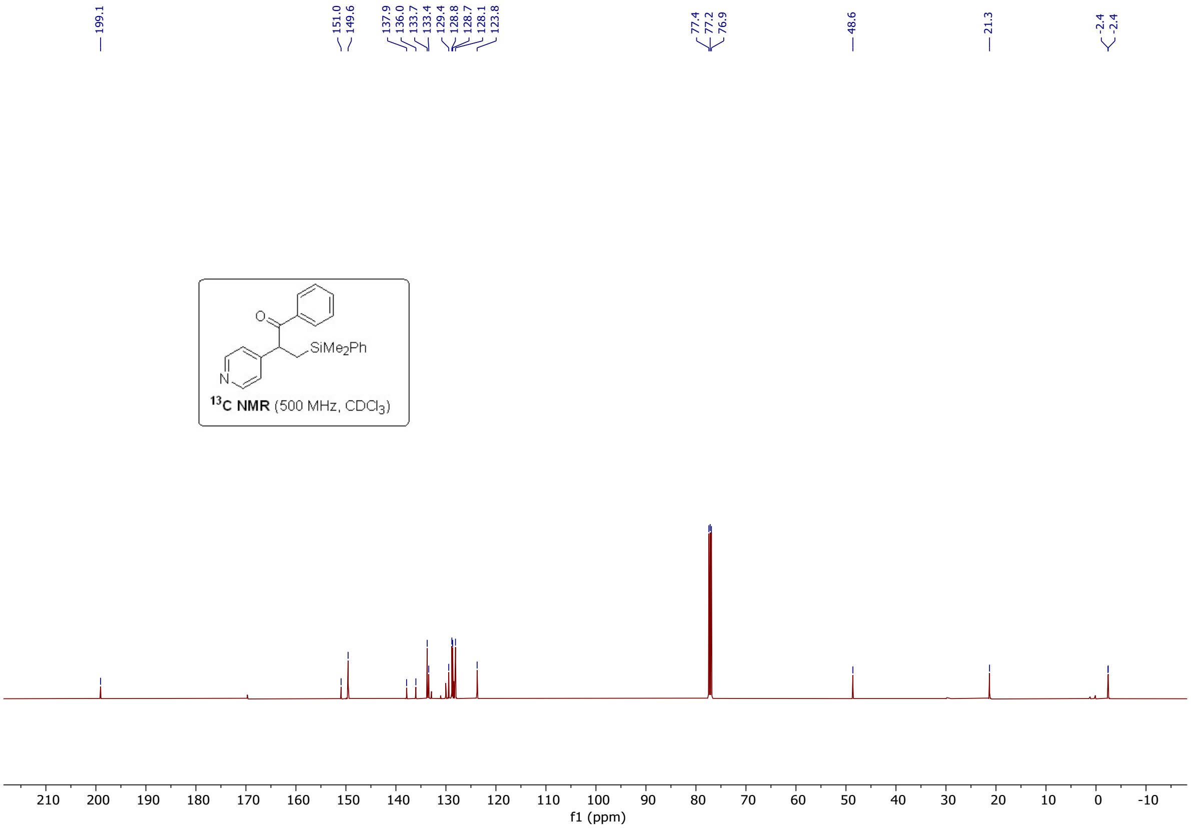

3-(dimethyl(phenyl)silyl)-1-phenyl-2-(quinolin-4-yl)propan-1-one (3am)

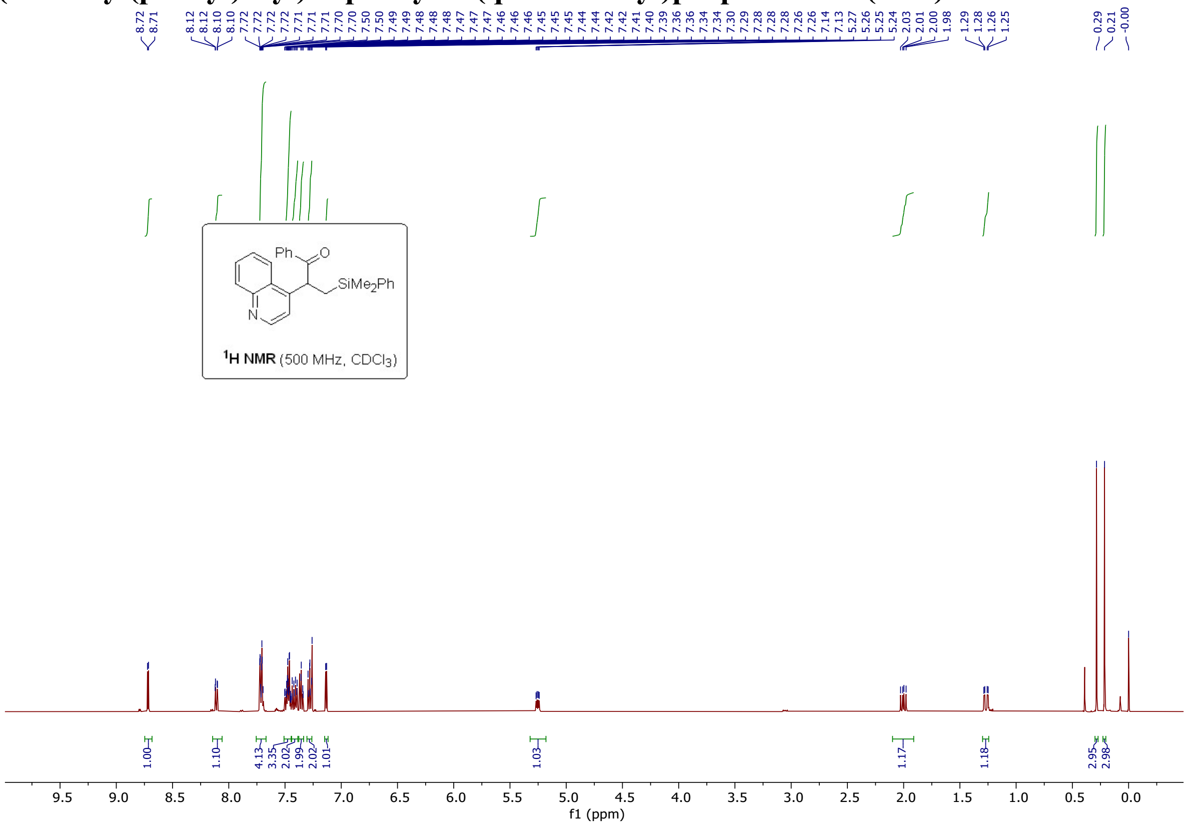

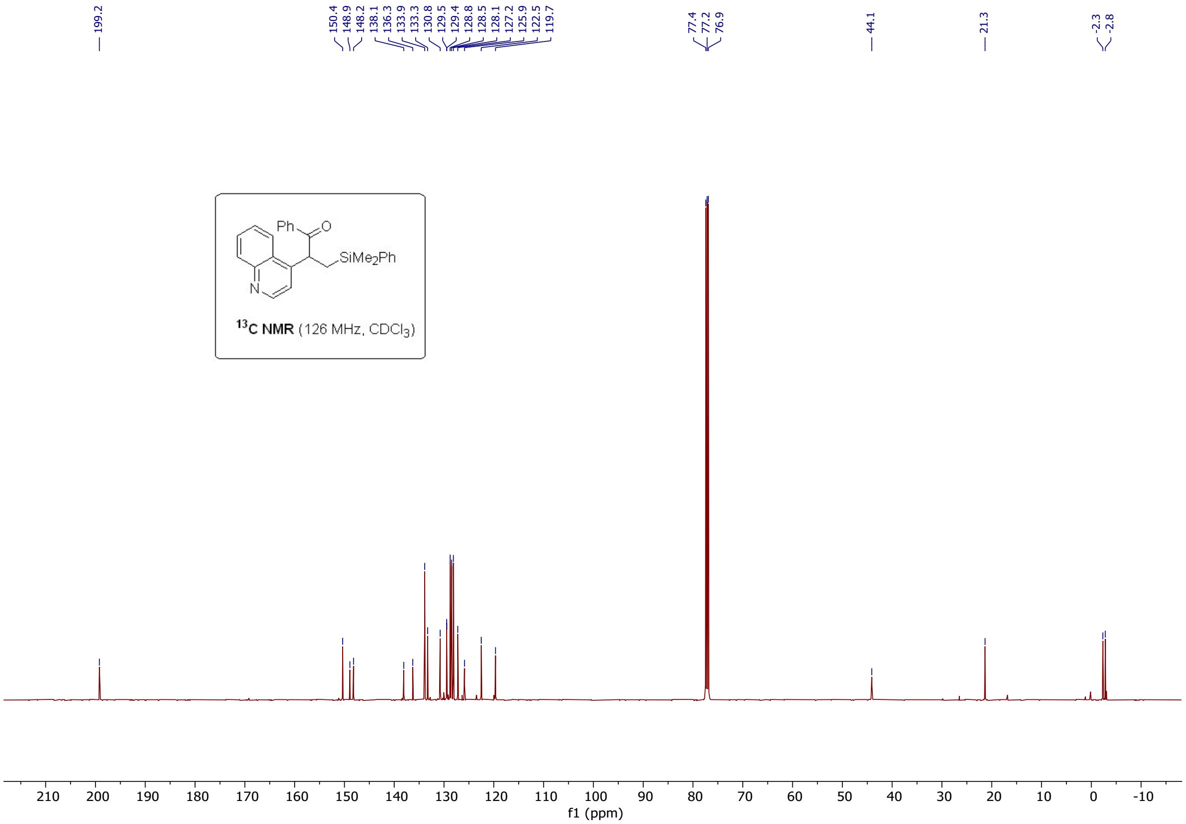

3-(dimethyl(phenyl)silyl)-2-methyl-1-phenyl-2-(pyrazin-2-yl)propan-1-one (3an)

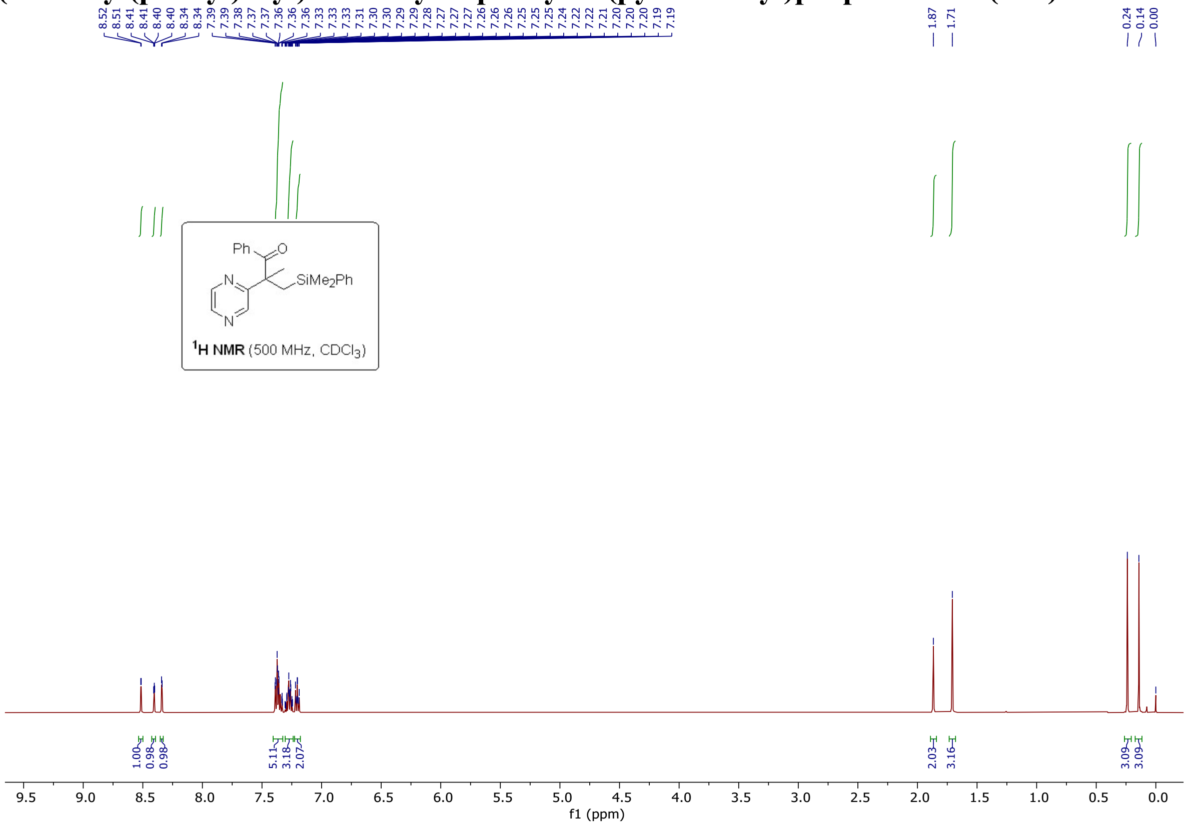

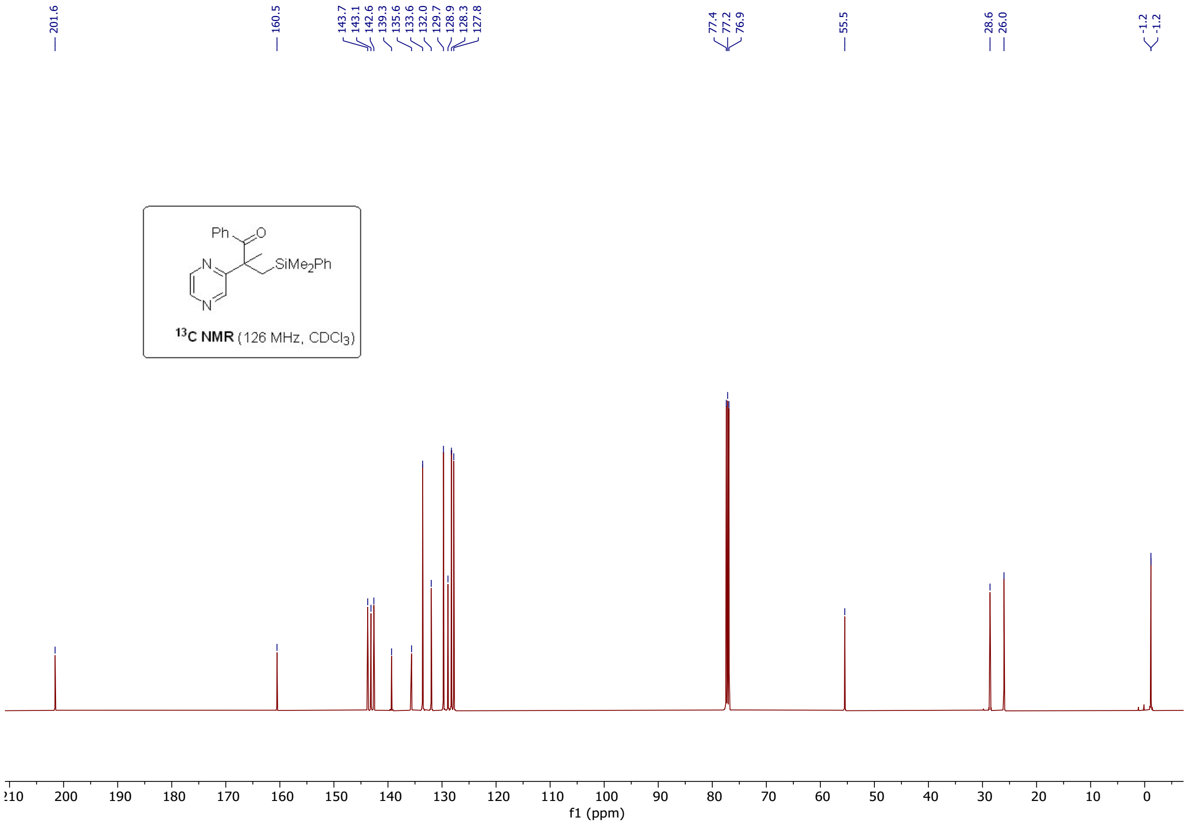

methyl 4-(3-(dimethyl(phenyl)silyl)-1-oxo-1-phenylpropan-2-yl)benzoate (3aa)

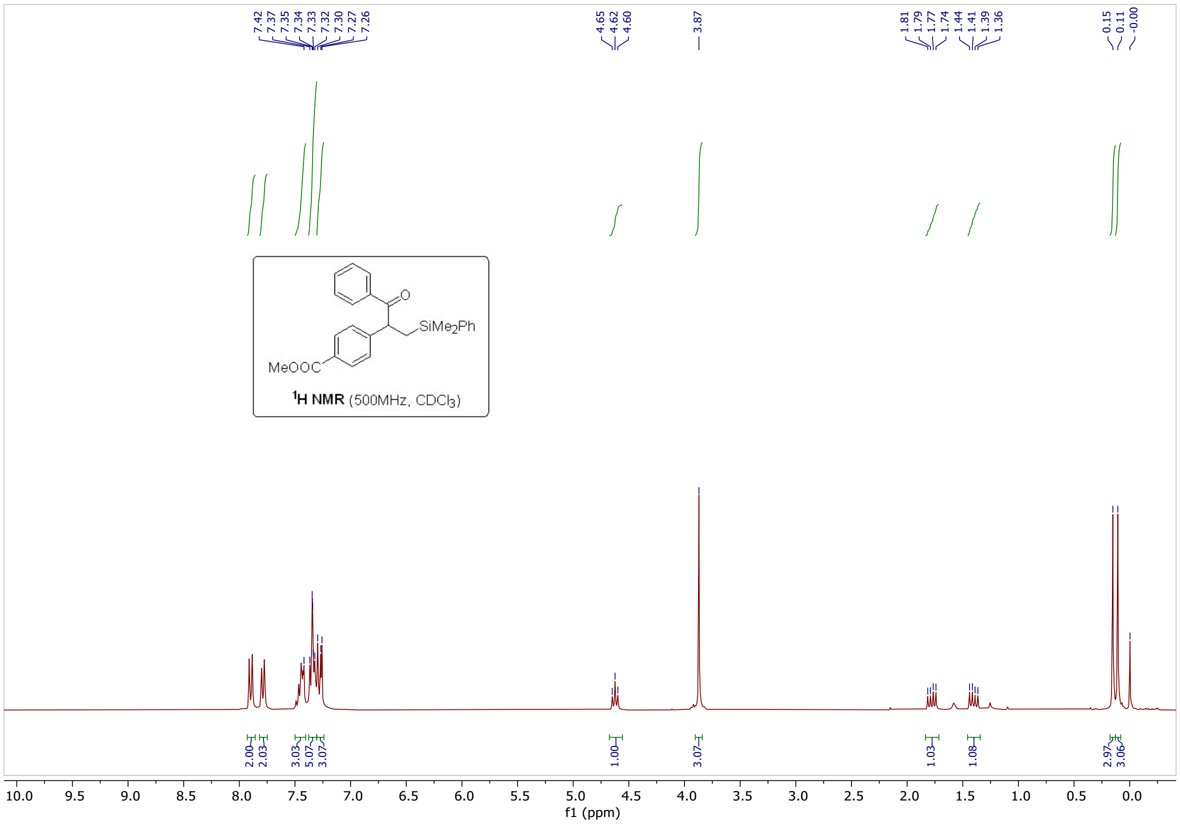

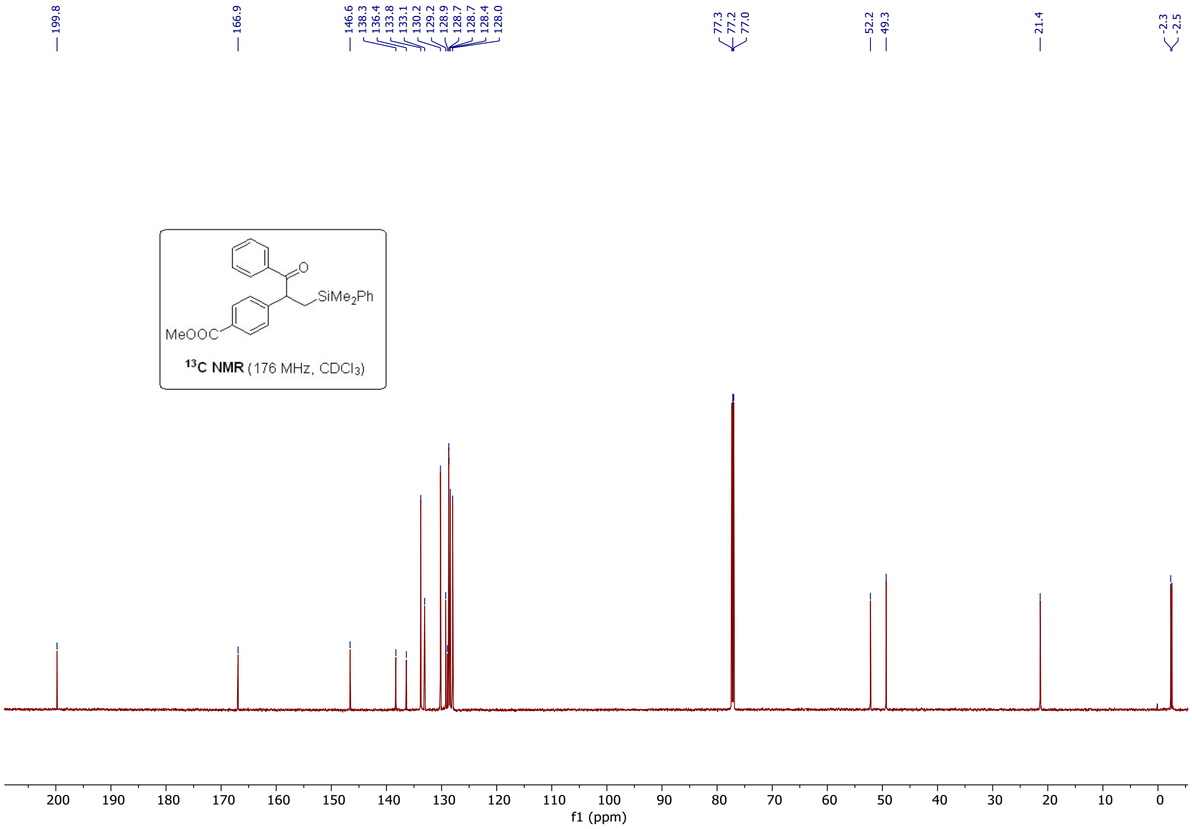

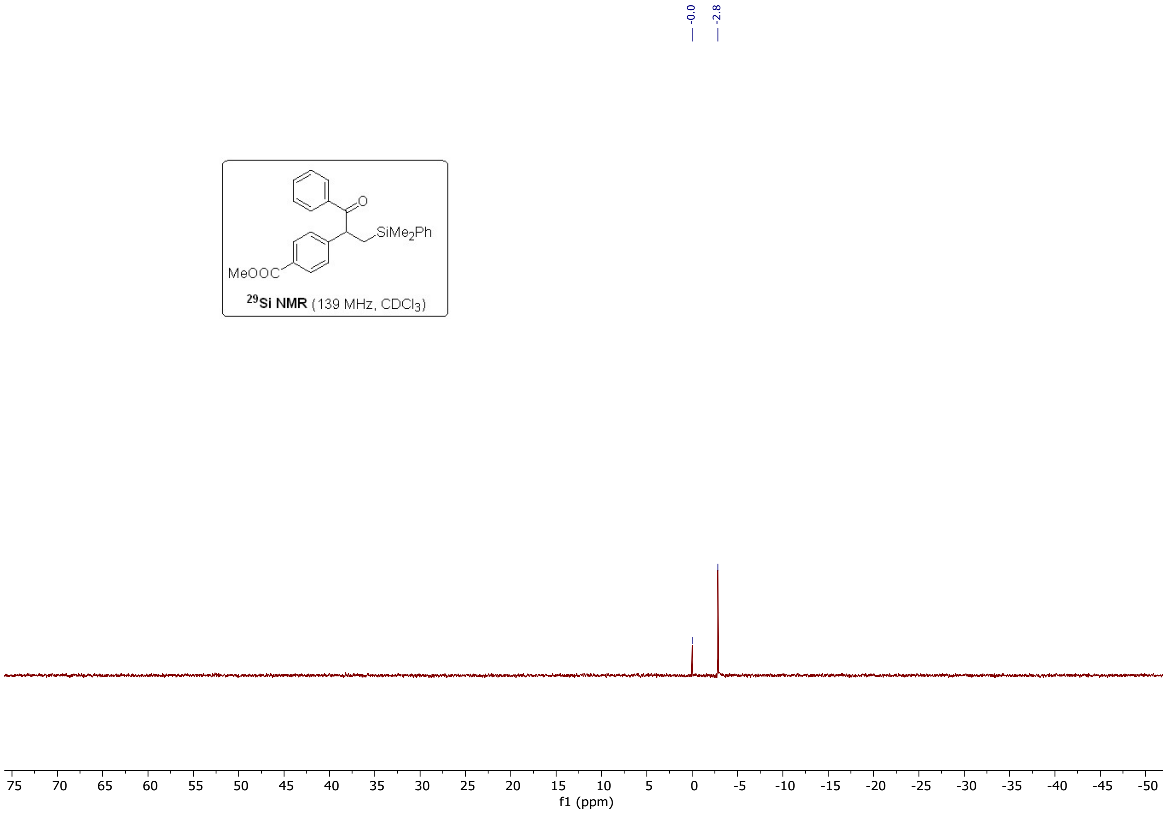

methyl 4-(3-(dimethyl(phenyl)silyl)-1-oxo-1-phenylpropan-2-yl)benzoate (3aa’)

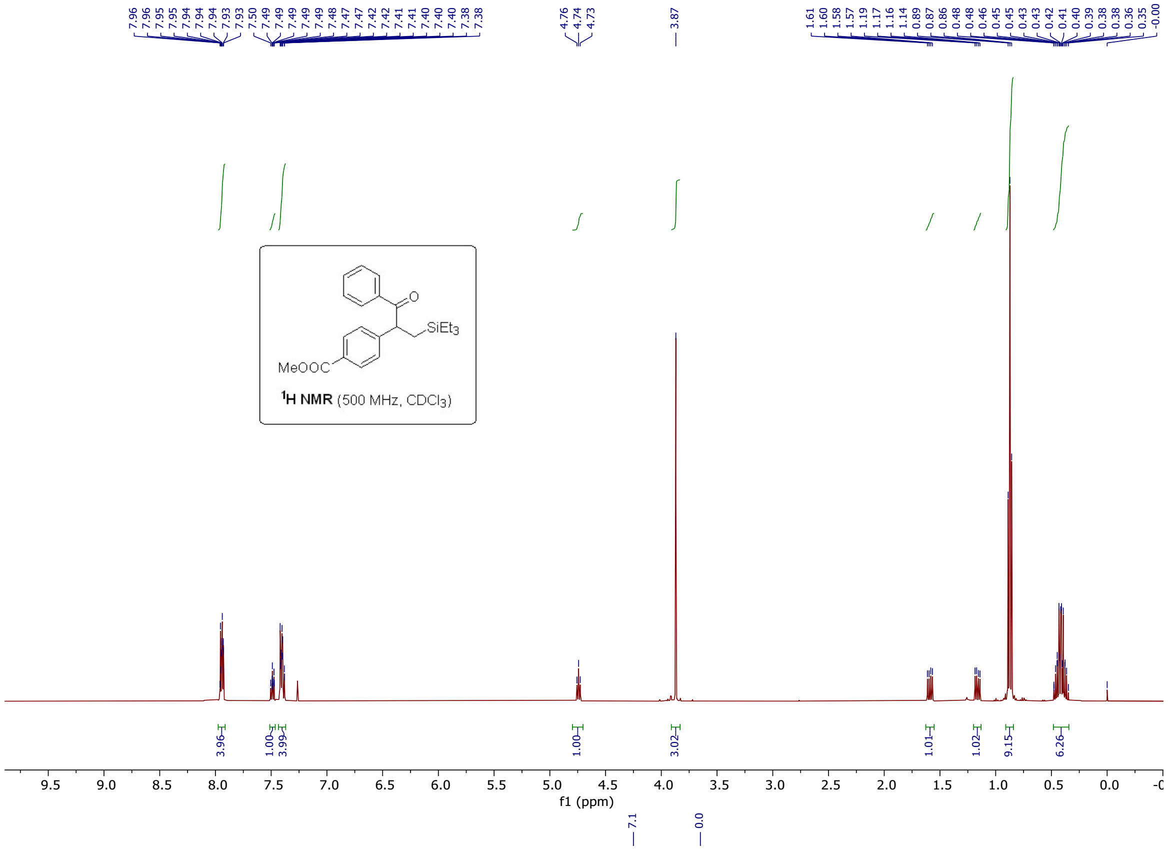

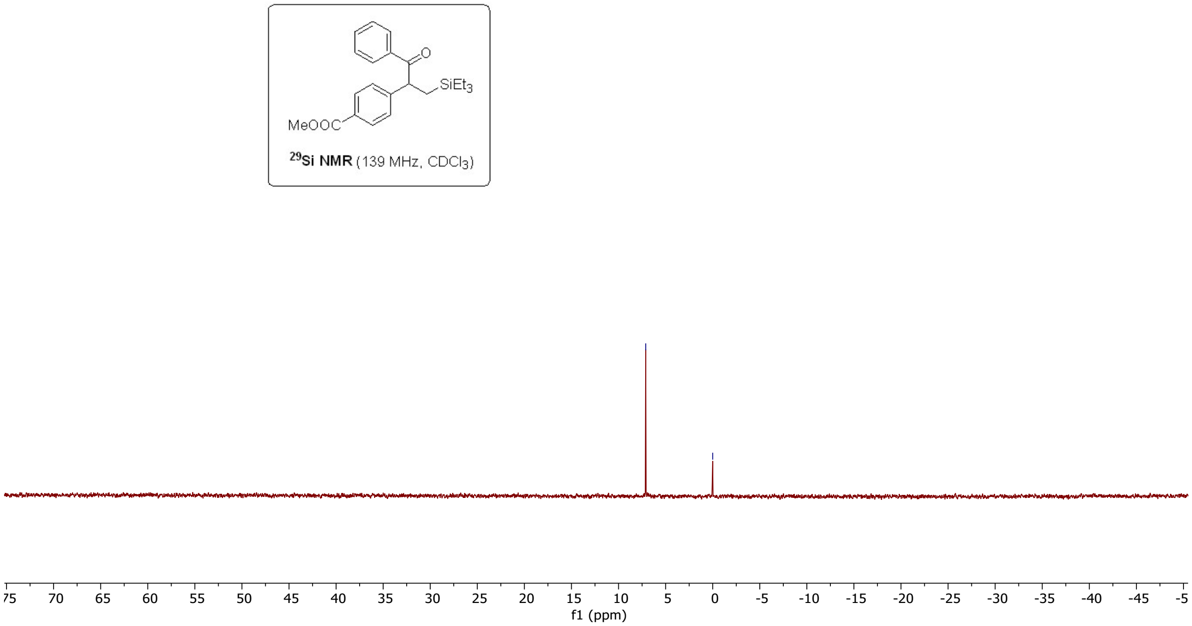

methyl 4-(1-oxo-1-phenyl-3-(tripropylsilyl)propan-2-yl)benzoate (3aa'')

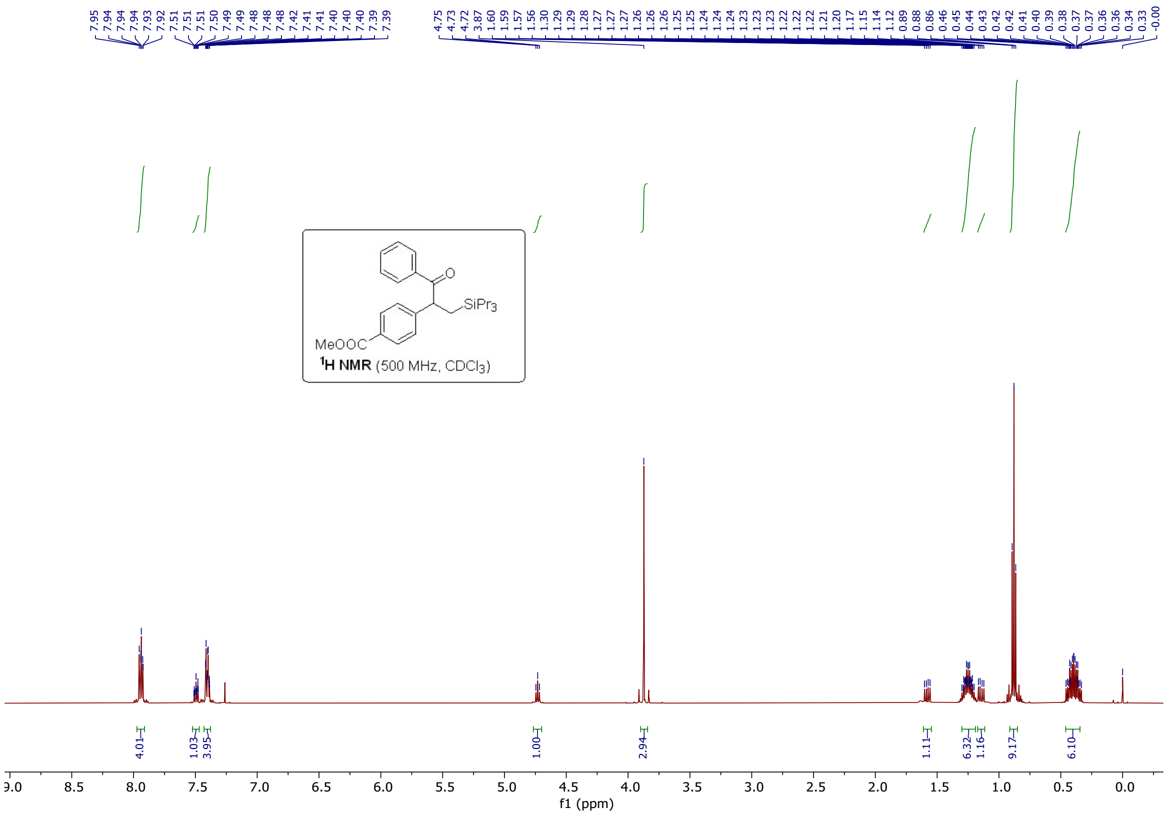
7.95 7.94 7.94 7.94 7.93 7.92 7.51 7.51 7.50 7.49 7.49 7.48 7.48 7.42 7.41 7.41 7.40 7.40 7.39 7.39
4.75 4.73 4.72 3.87 1.60 1.59 1.57 1.56 1.30 1.29 1.29 1.28 1.27 1.27 1.27 1.26 1.26 1.26 1.25 1.25 1.24 1.24 1.24 1.23 1.23 1.23 1.22 1.22 1.22 1.21 1.20 1.17 1.15 1.14 1.12 0.89 0.88 0.86 0.46 0.45 0.44 0.43 0.42 0.42 0.41 0.40 0.39 0.38 0.37 0.37 0.36 0.36 0.34 0.33 -0.00
MeOOC
SiPr3
O
^{1}H NMR (500 MHz, $CDCl_3$)
4.01 1.03 3.95 1.00 2.94 1.11 6.32 1.16 9.17 6.10
f1 (ppm)

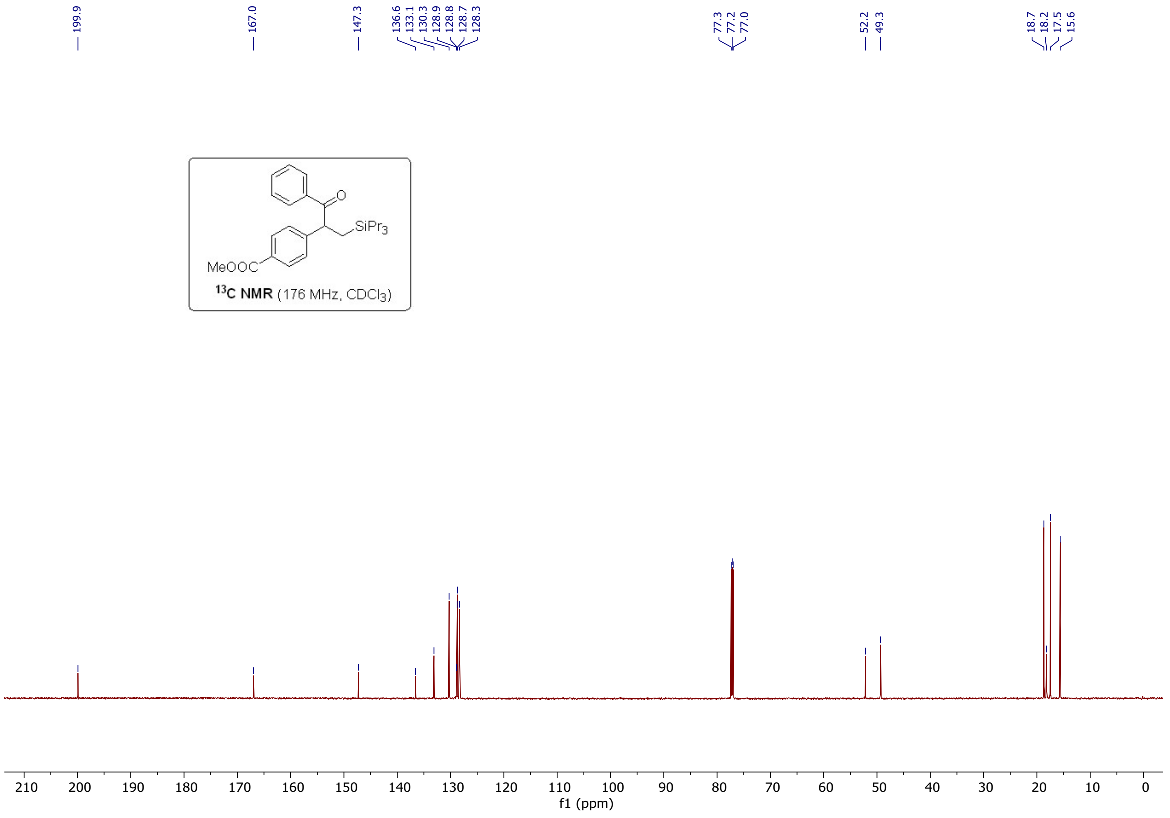
199.9 167.0 147.3 136.6 133.1 130.3 128.9 128.8 128.7 128.3 77.3 77.2 77.0 52.2 49.3 18.7 18.2 17.5 15.6
MeOOC
SiPr3
O
^{13}C NMR (176 MHz, $CDCl_3$)
f1 (ppm)

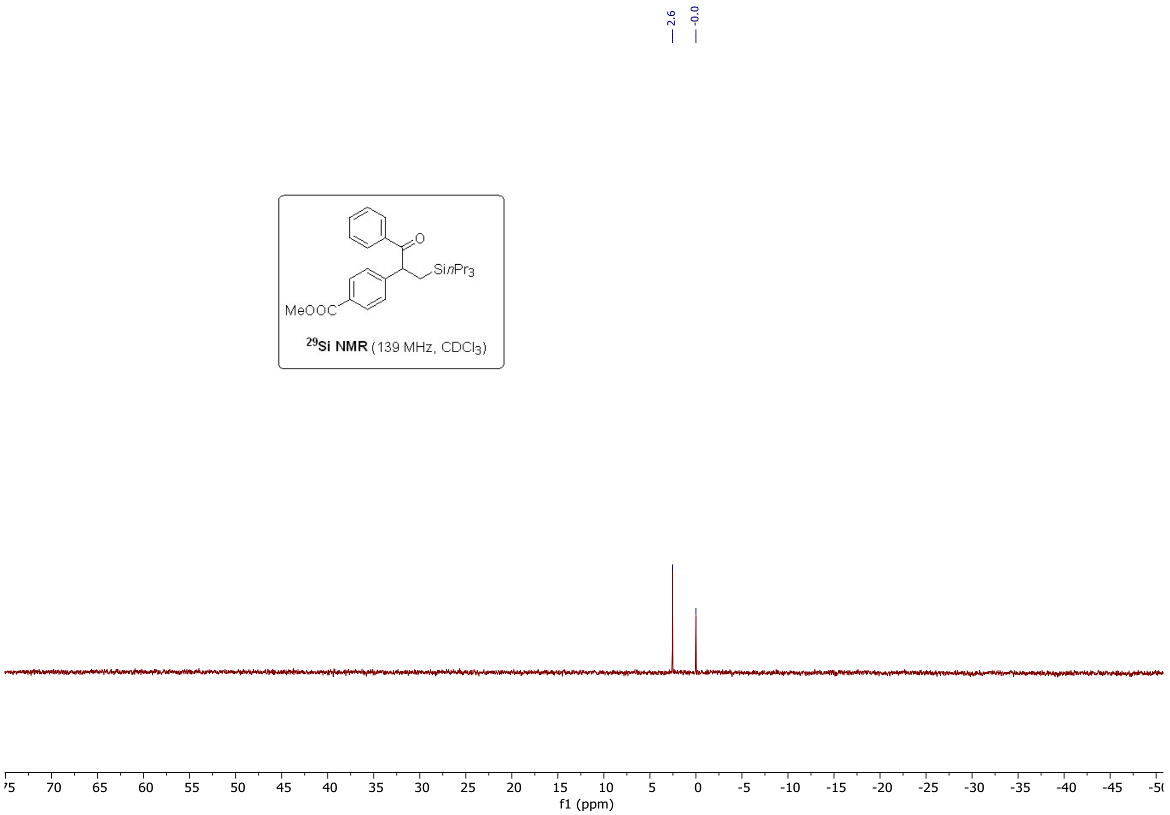

methyl 4-(3-(dimethyl(phenyl)silyl)-1-oxo-1-(p-tolyl)propan-2-yl)benzoate (3ba)

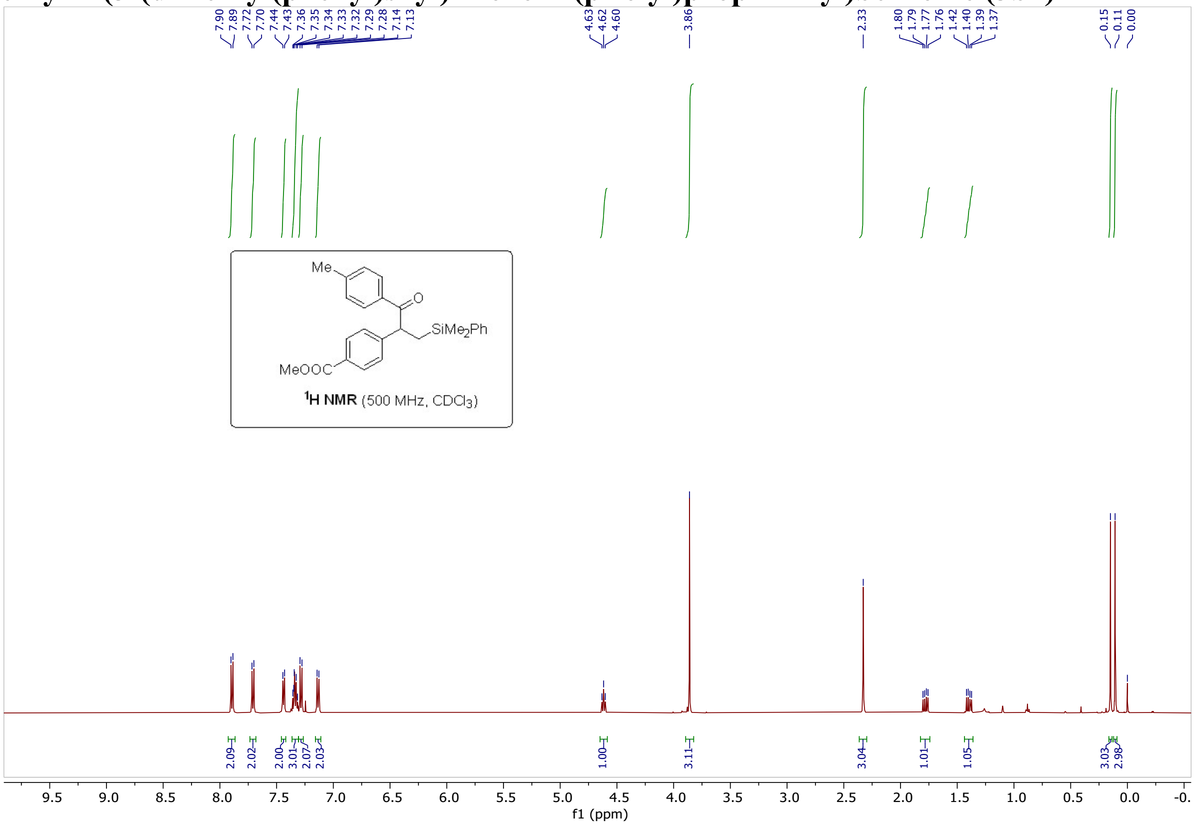

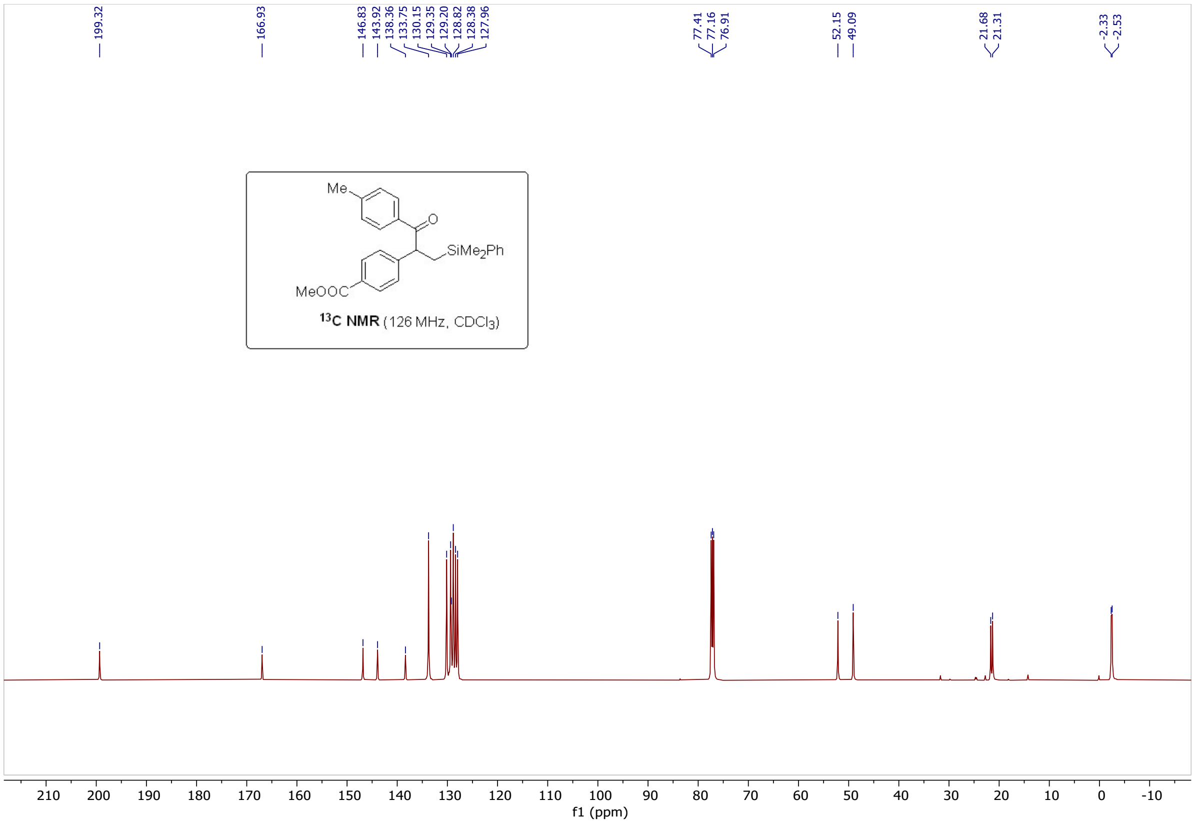

methyl 4-(3-(dimethyl(phenyl)silyl)-1-(4-methoxyphenyl)-1-oxopropan-2-yl)benzoate (3ca)

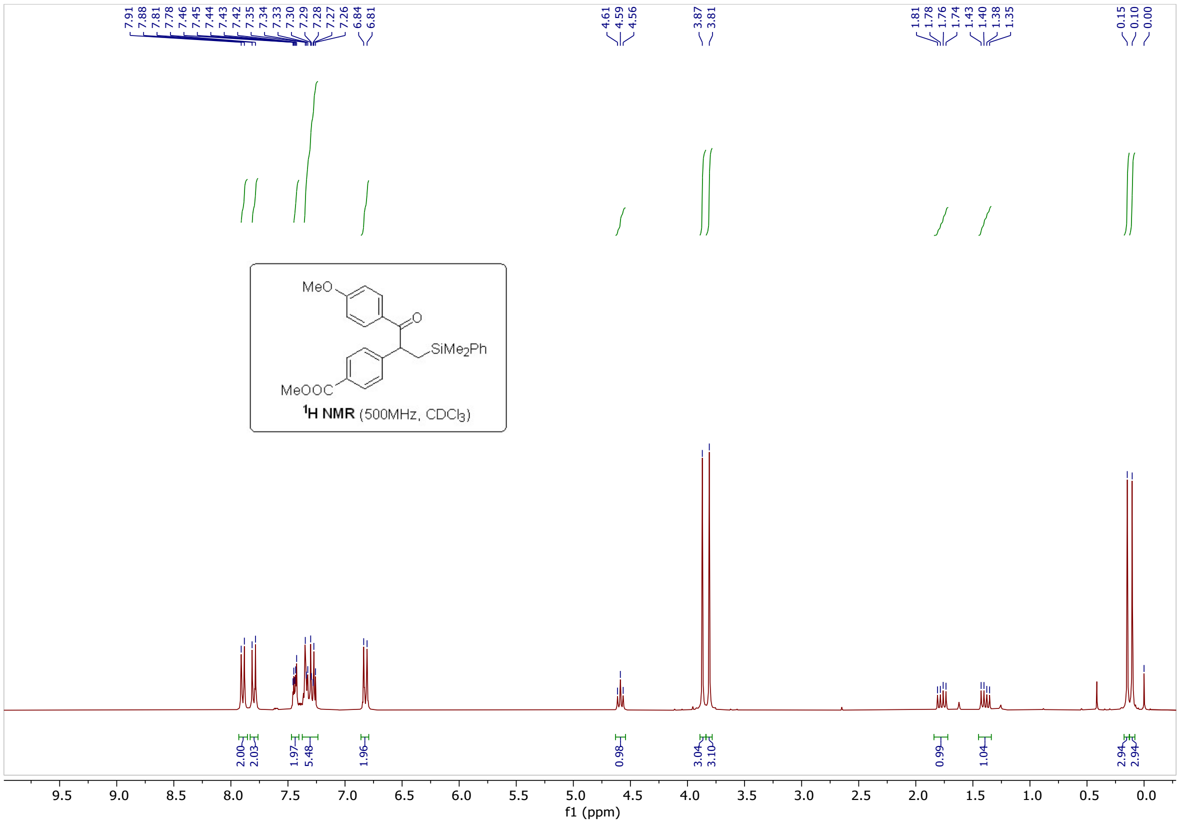

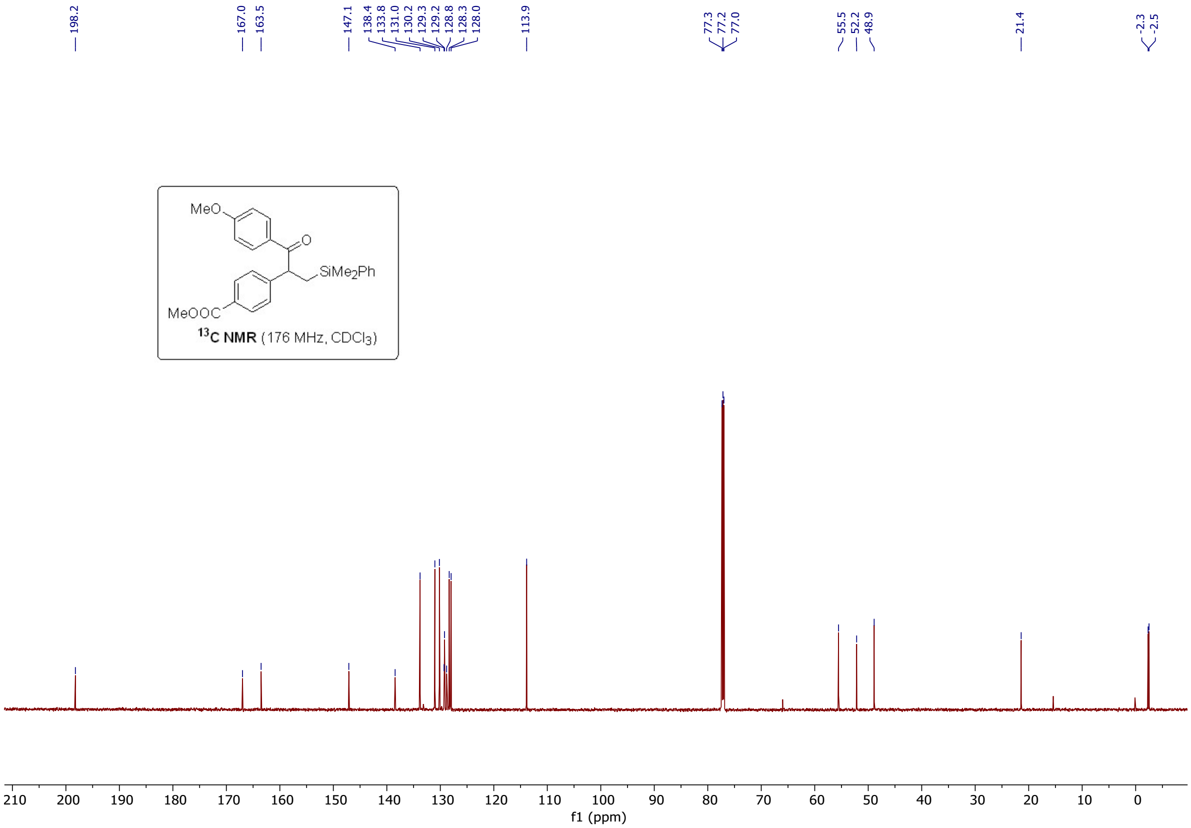

methyl 4-(1-(4-cyclohexylphenyl)-3-(dimethyl(phenyl)silyl)-1-oxopropan-2-yl)benzoate (3da)

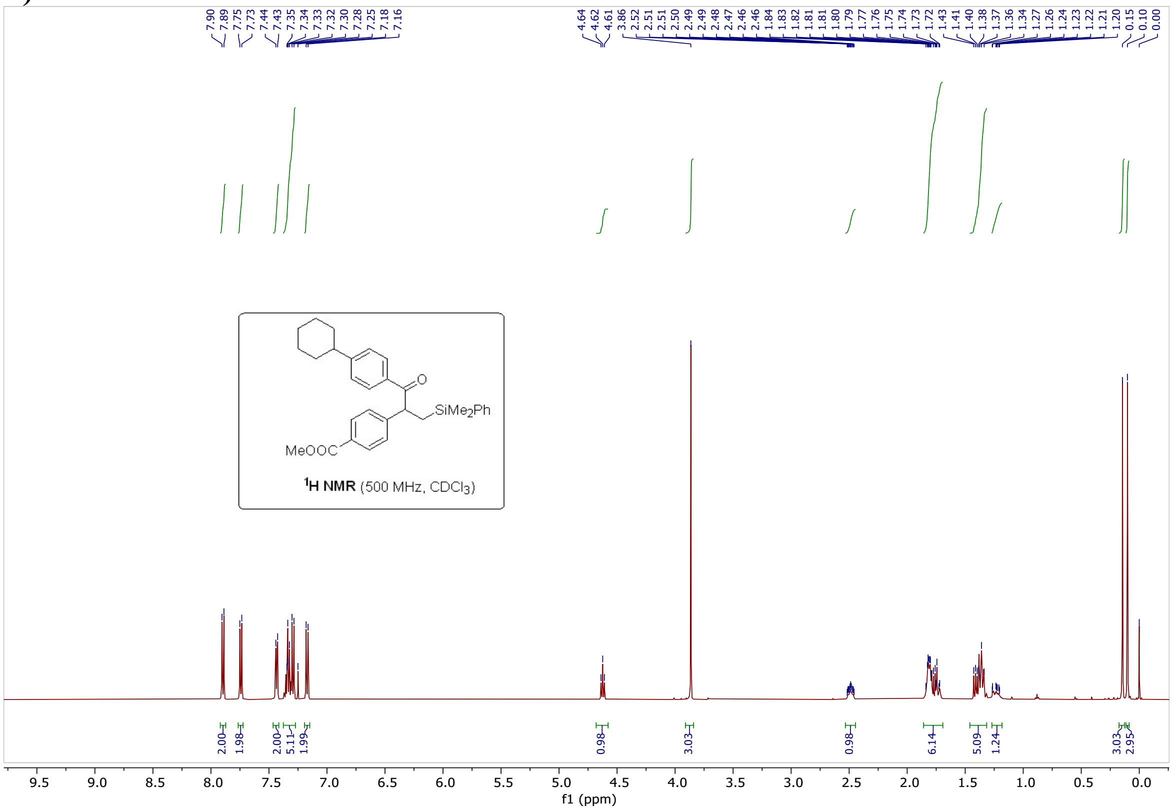

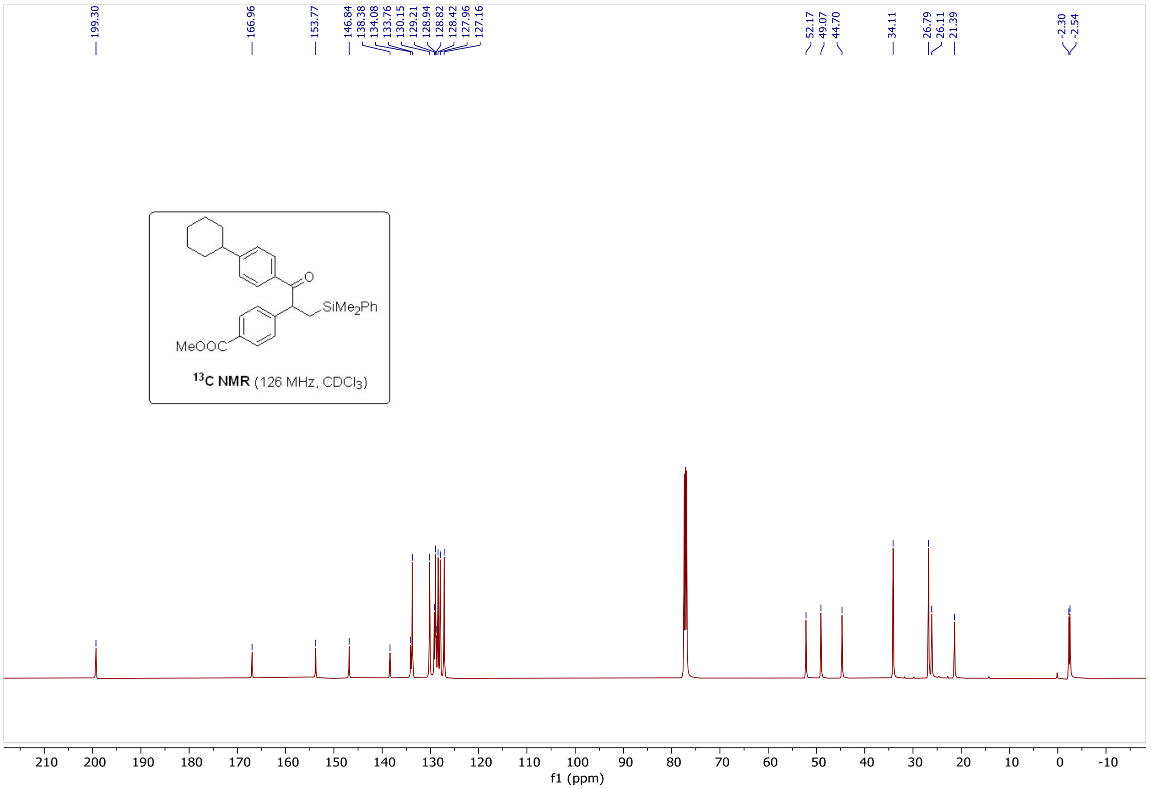

methyl 4-(1-(3,5-di-tert-butylphenyl)-3-(dimethyl(phenyl)silyl)-1-oxopropan-2-yl)benzoate (3ea)

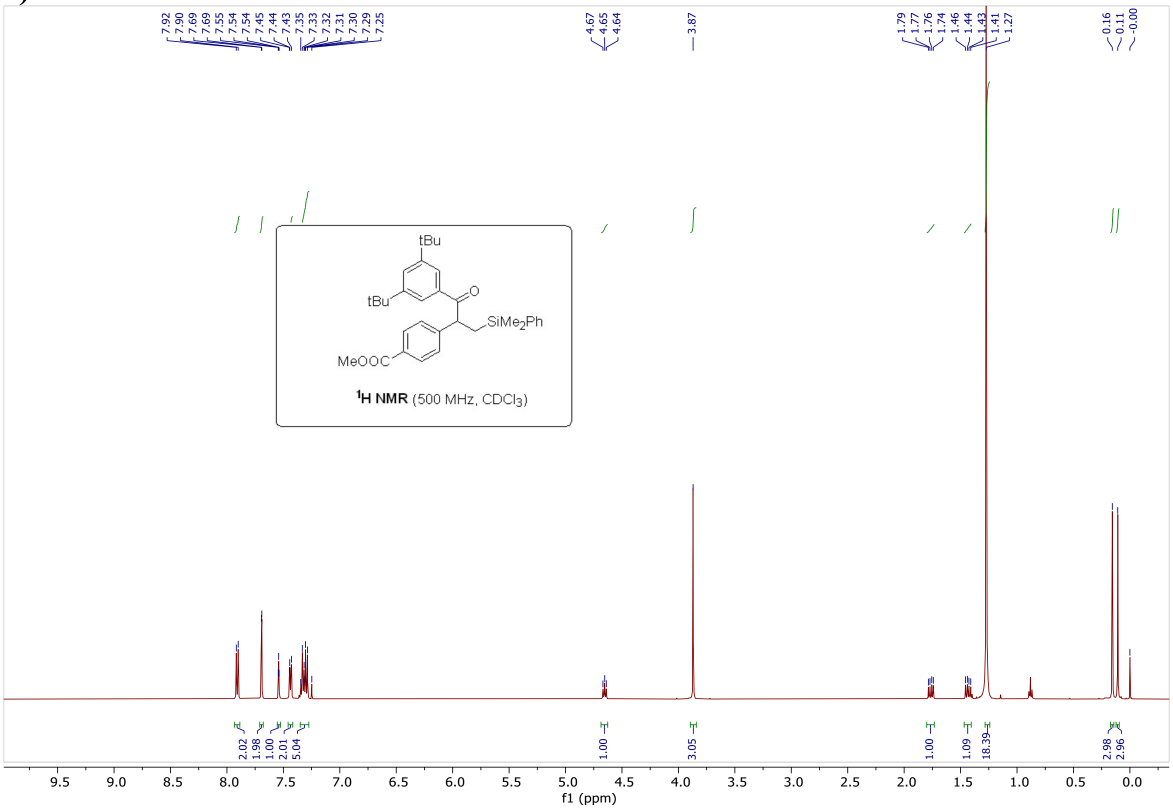

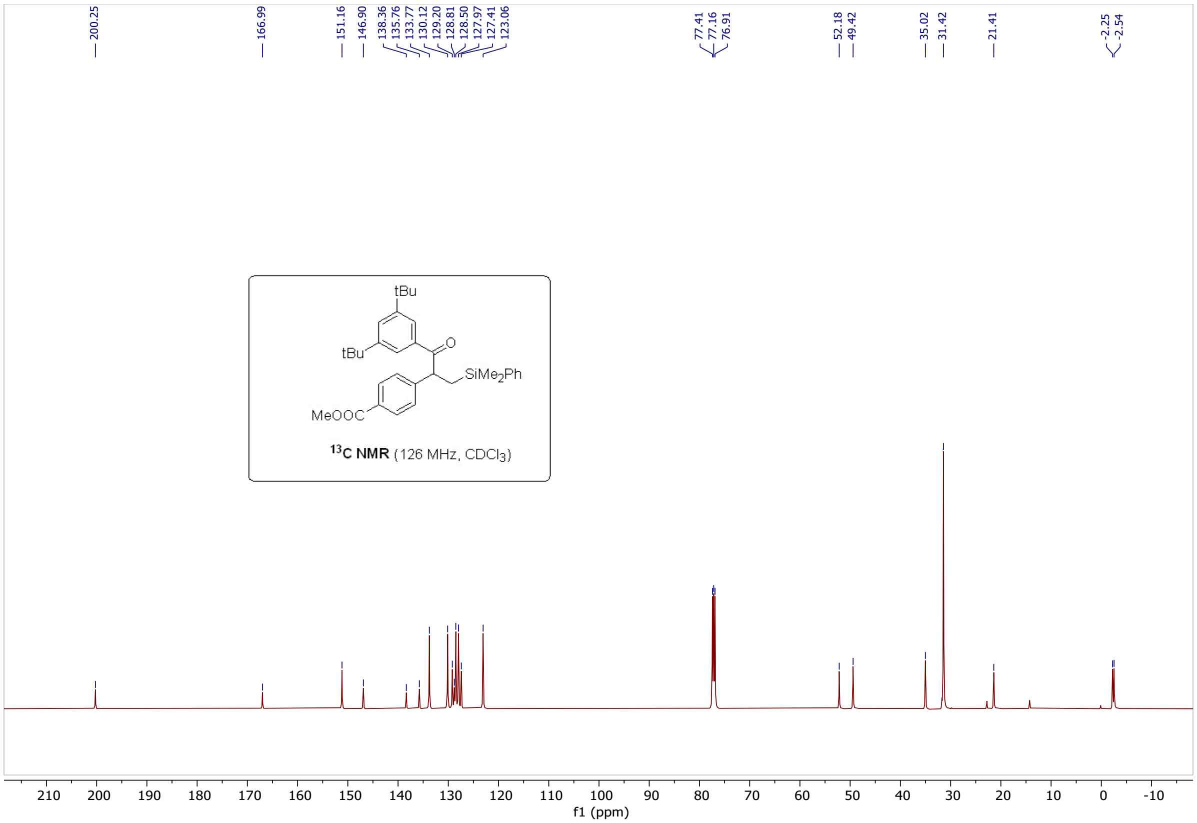

methyl 4-(3-(dimethyl(phenyl)silyl)-1-oxo-1-(4-(trifluoromethyl)phenyl)propan-2-yl)benzoate (3fa)

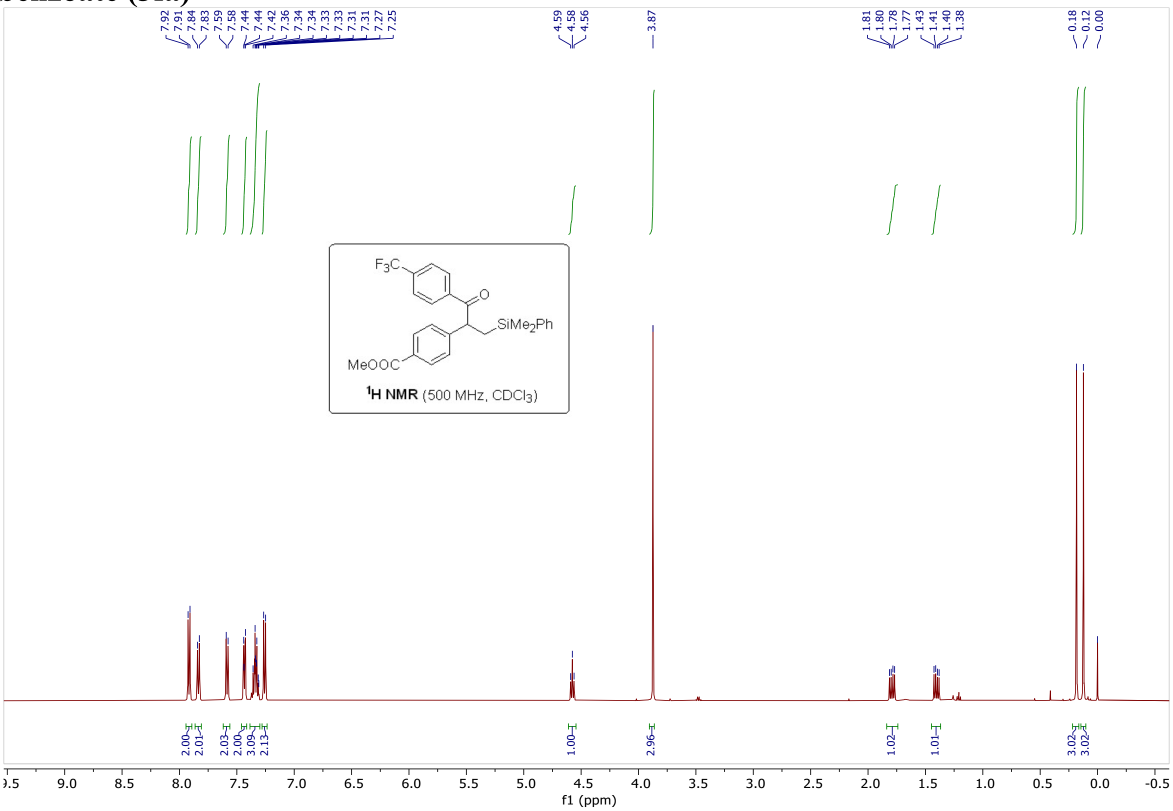

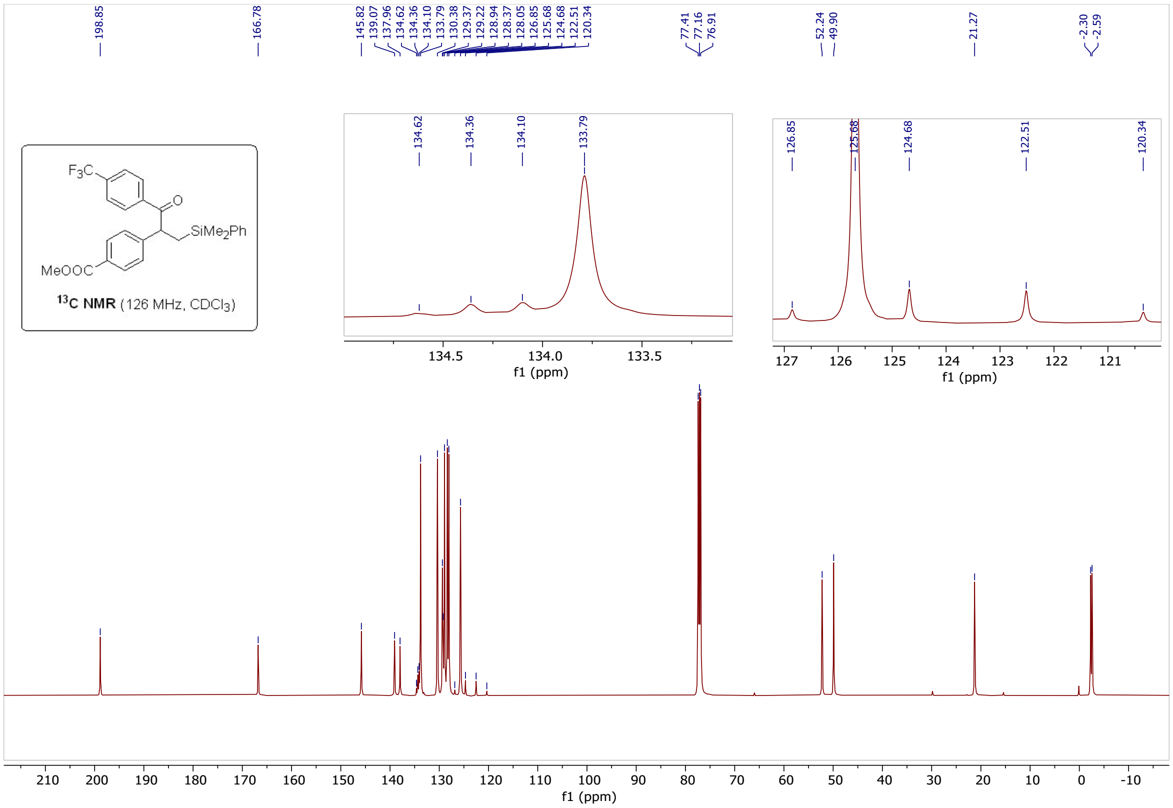

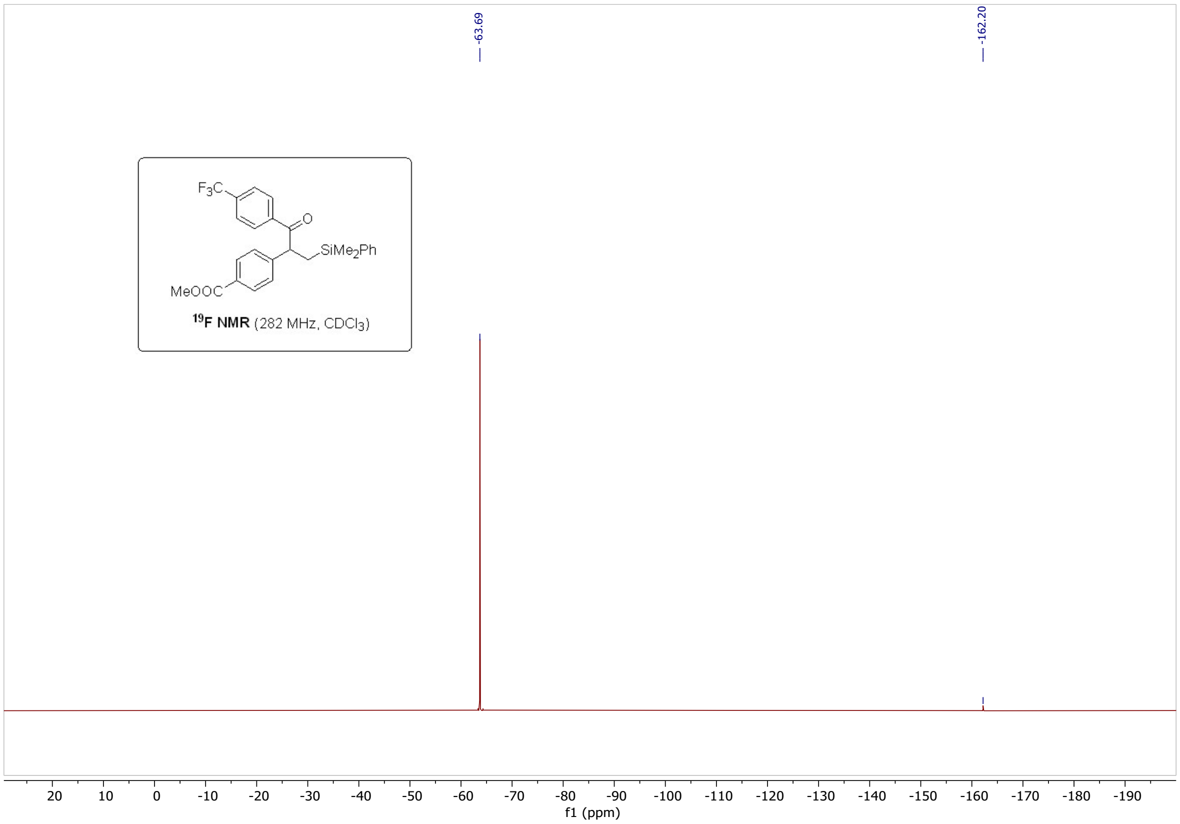

methyl 4-(3-(dimethyl(phenyl)silyl)-1-(4-fluorophenyl)-1-oxopropan-2-yl)benzoate (3ga)

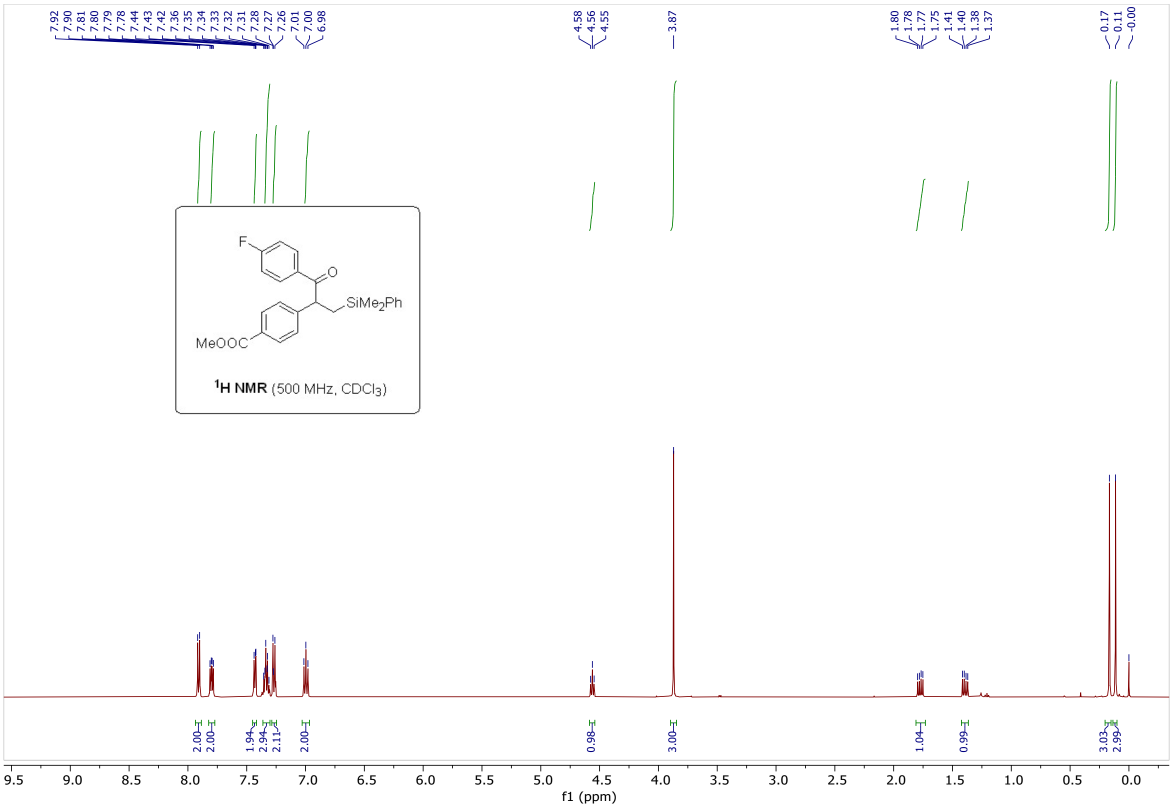

F
O
SiMe2Ph
MeOOC
1H NMR (500 MHz, CDCl3)
f1 (ppm)

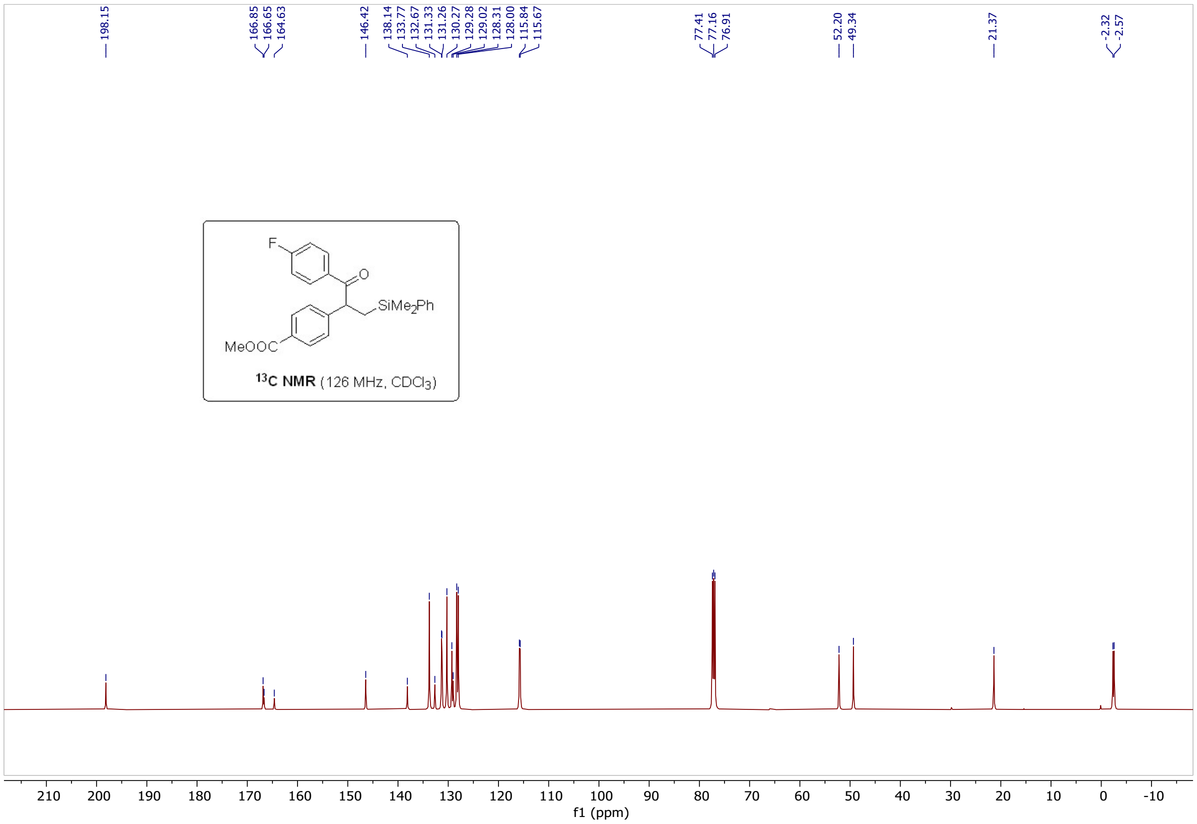

F
O
SiMe2Ph
MeOOC
13C NMR (126 MHz, CDCl3)
f1 (ppm)

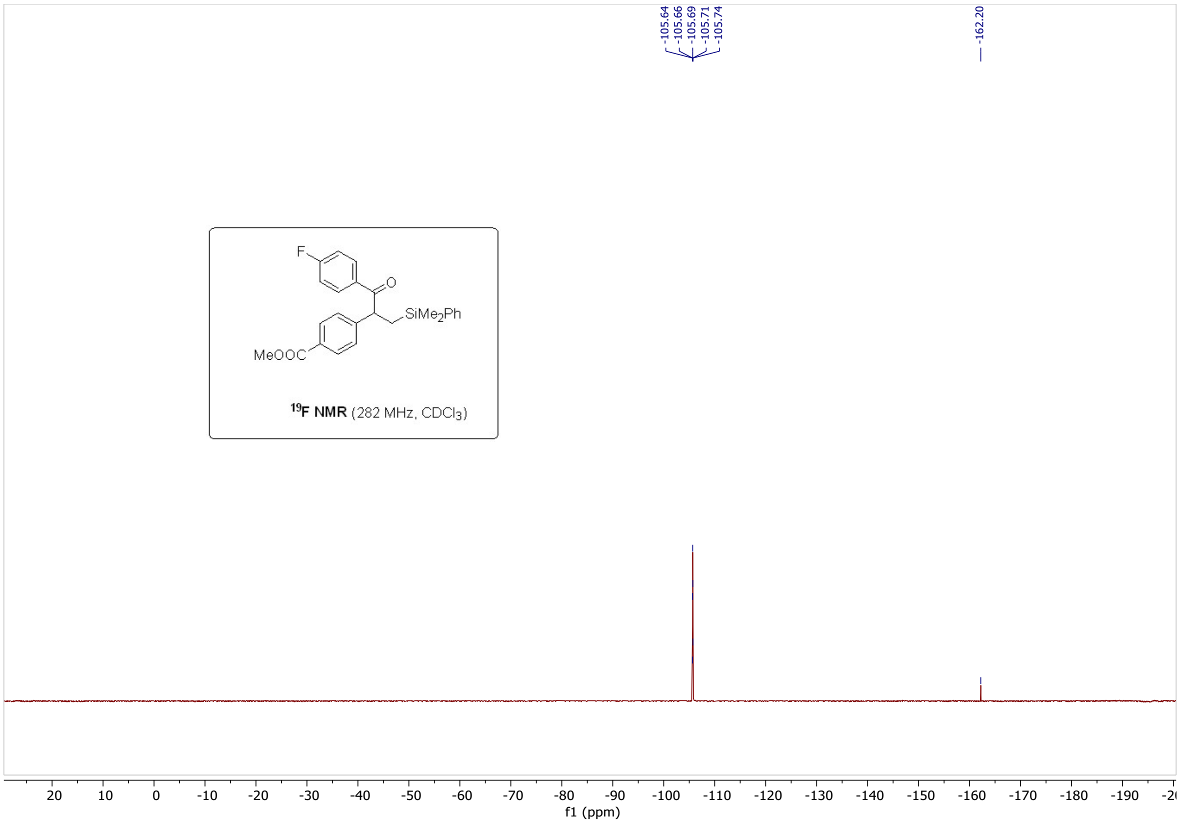

methyl 4-(1-(4-chlorophenyl)-3-(dimethyl(phenyl)silyl)-1-oxopropan-2-yl)benzoate (3ha)

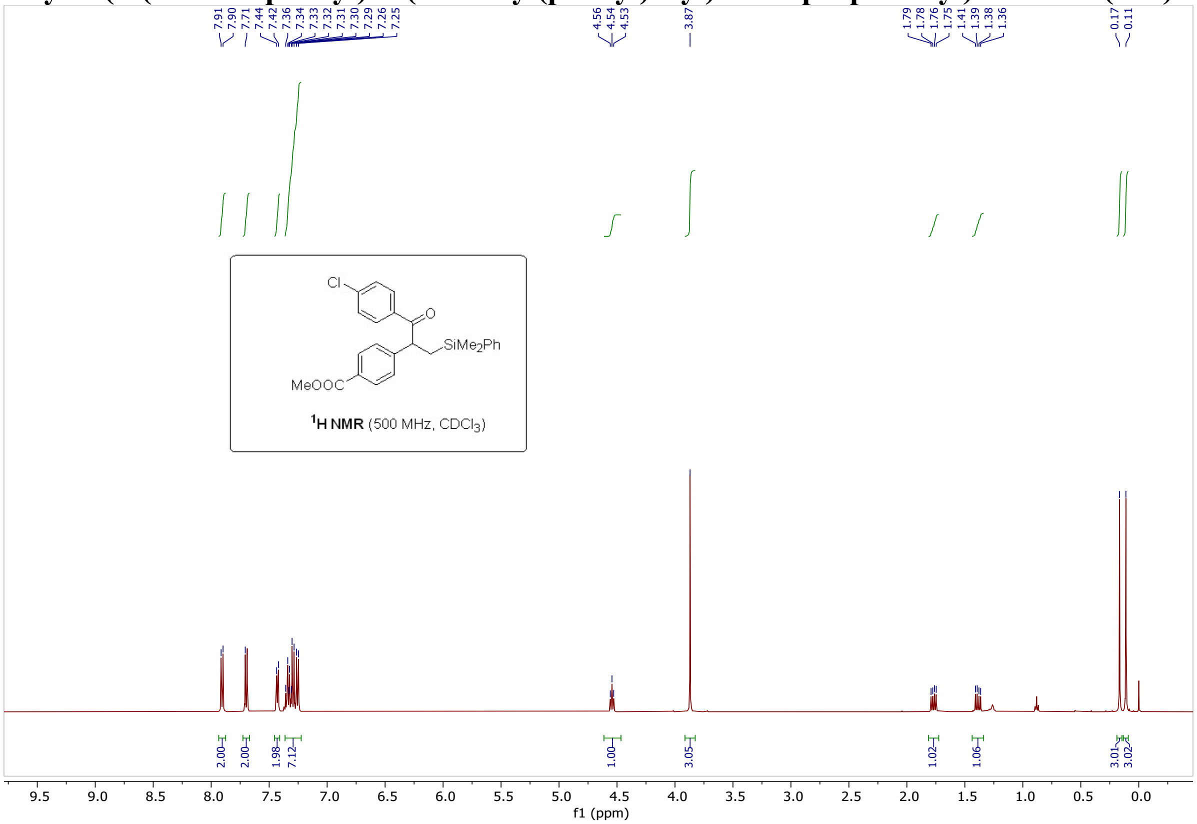

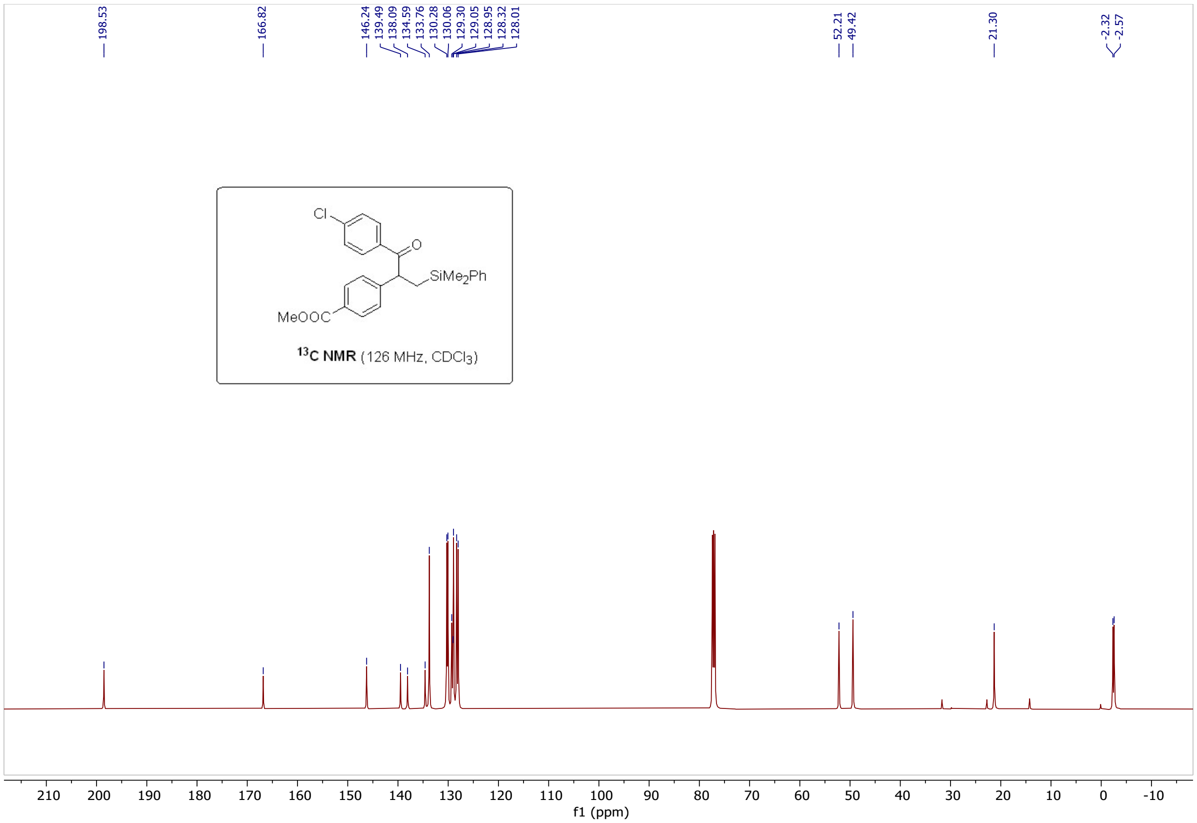

methyl 4-(1-(4-bromophenyl)-3-(dimethyl(phenyl)silyl)-1-oxopropan-2-yl)benzoate (3ia)

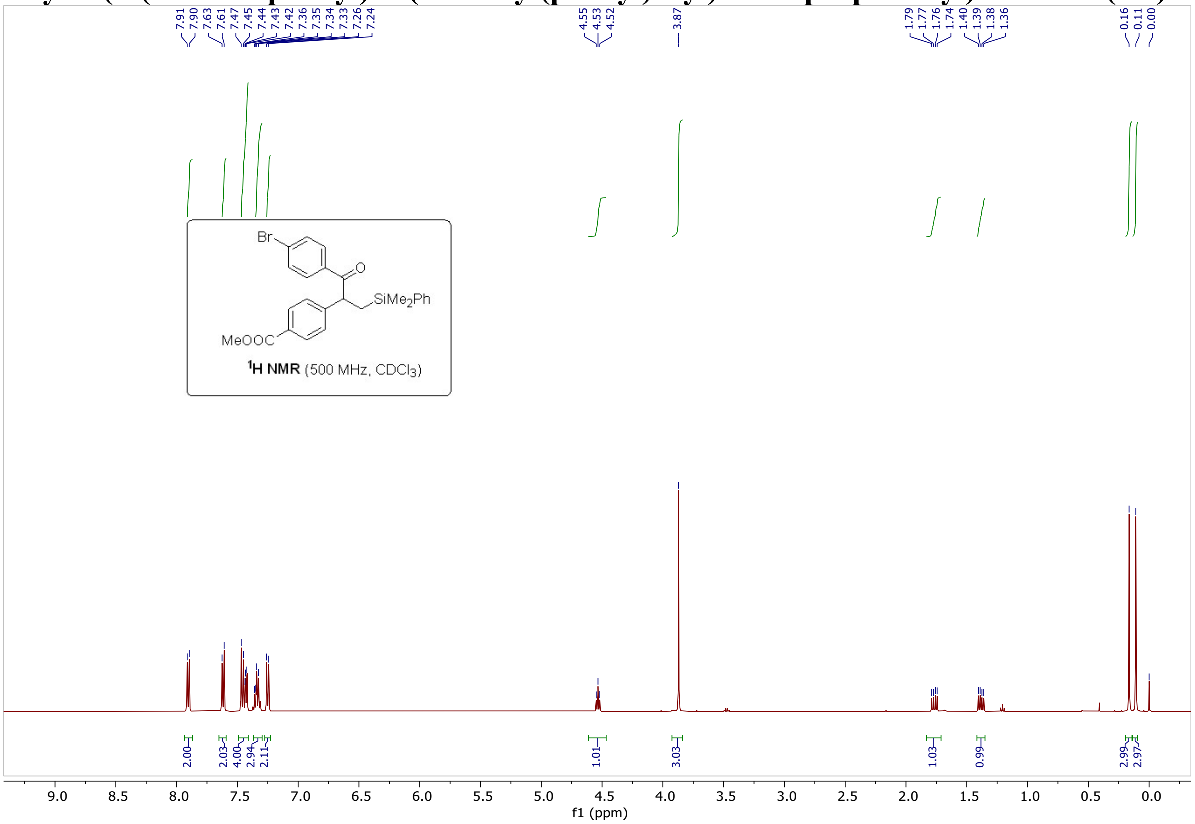

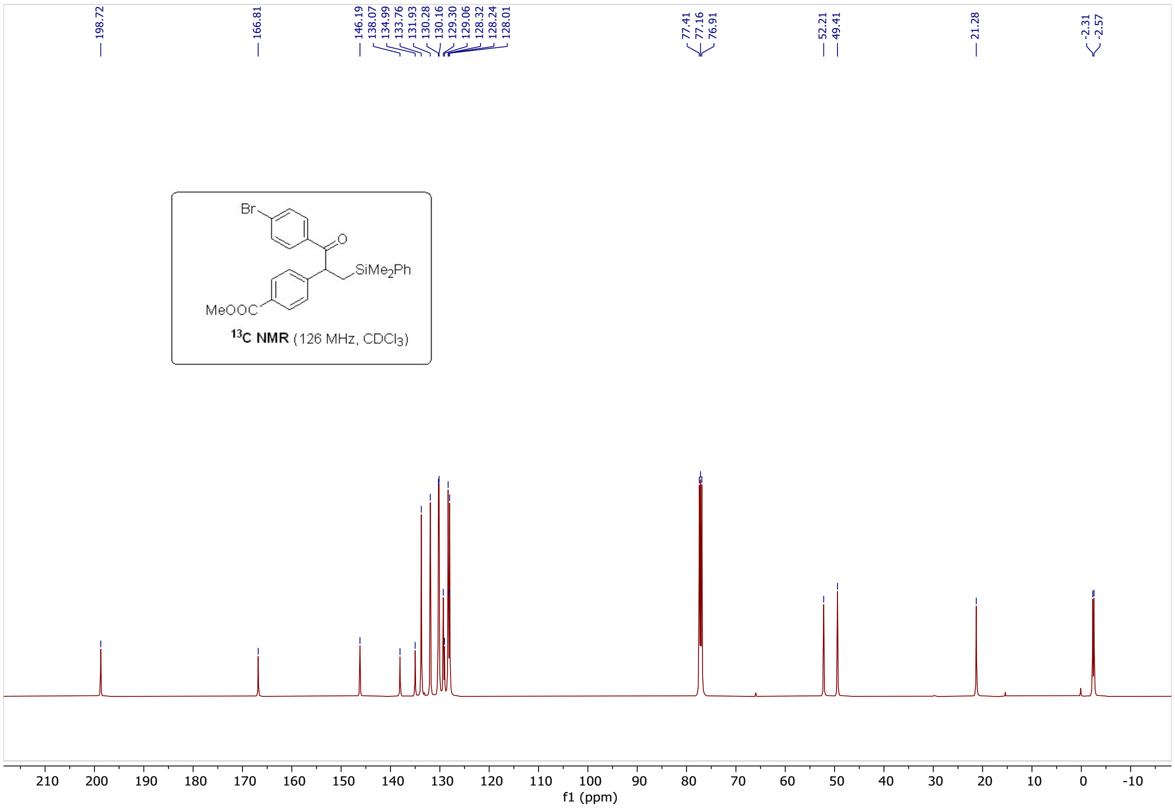

methyl 4-(3-(dimethyl(phenyl)silyl)-1-oxo-1-(4-(trifluoromethoxy)phenyl)propan-2-yl)benzoate (3ja)

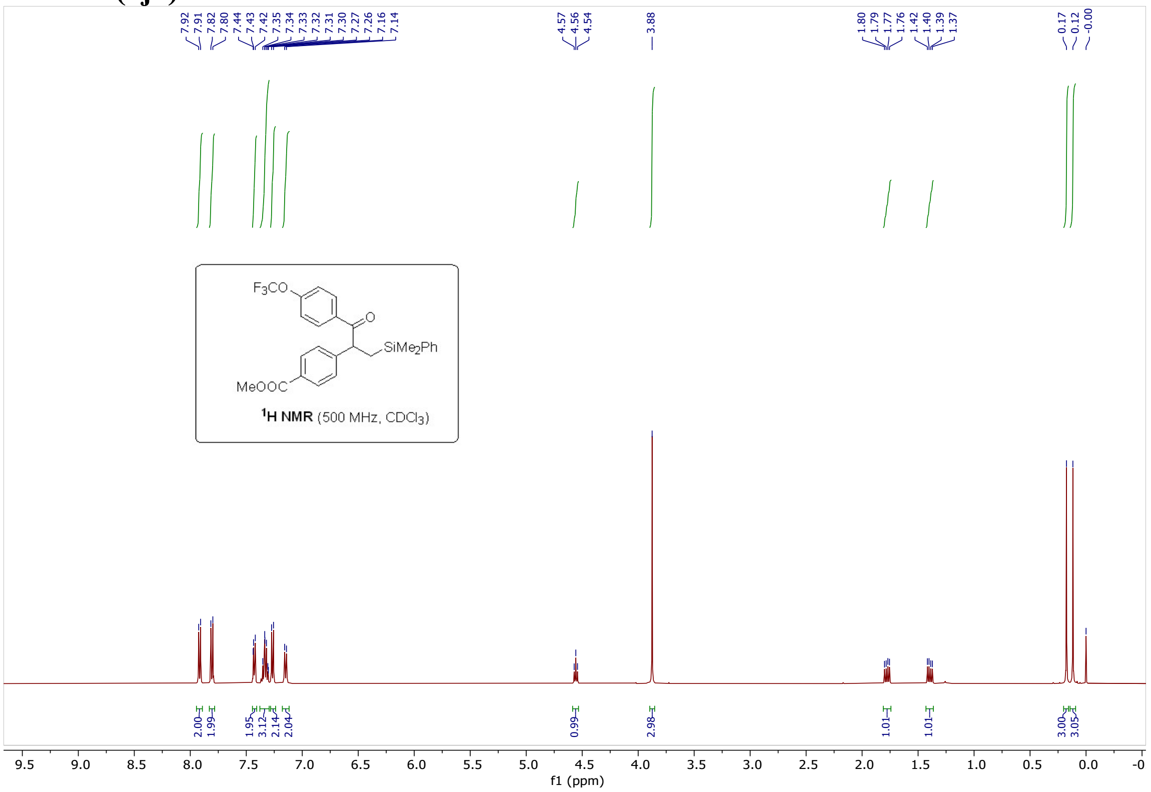

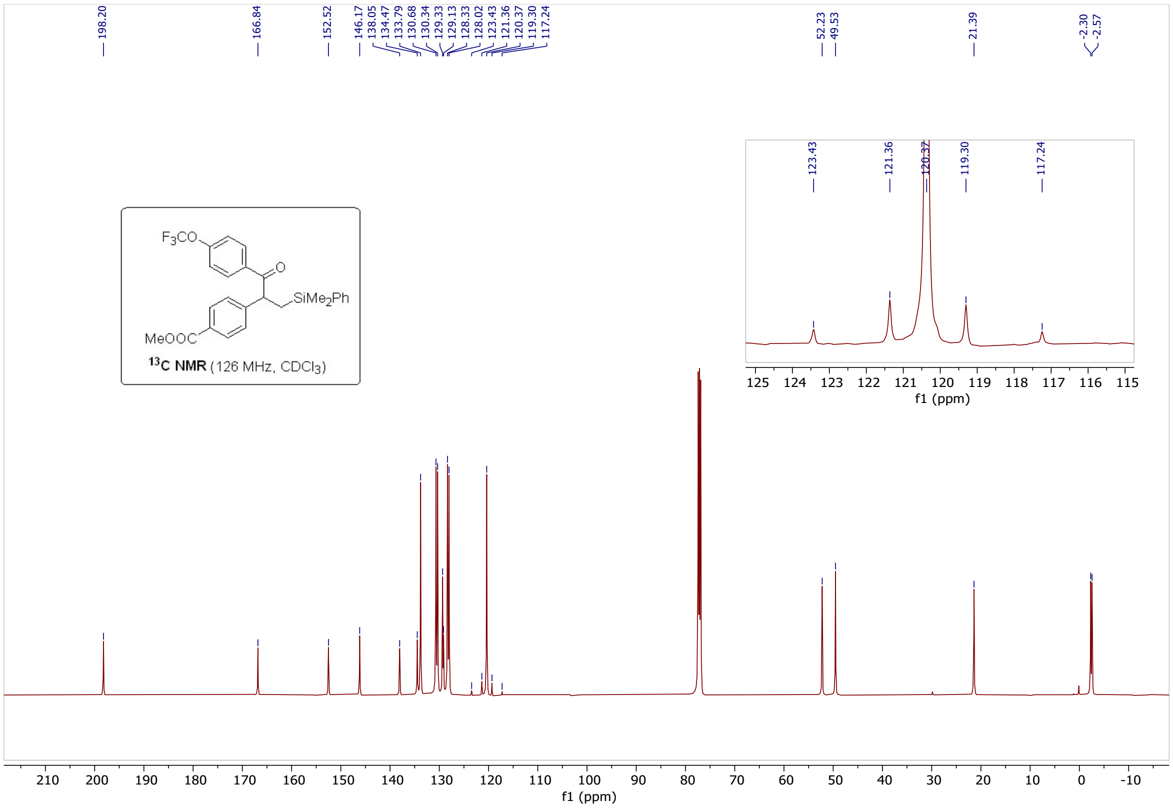
F3CO
O
SiMe2Ph
MeOOC
13C NMR (126 MHz, CDCl3)
f1 (ppm)

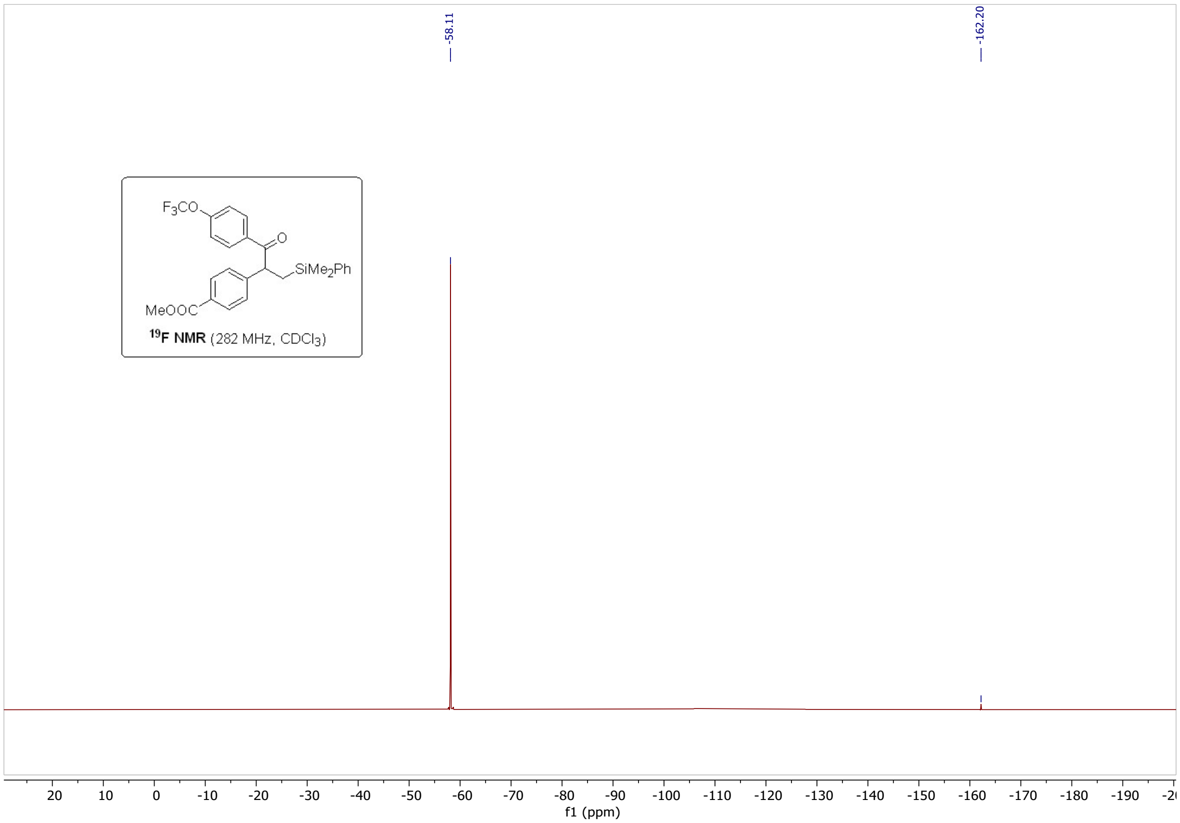
F3CO
O
SiMe2Ph
MeOOC
19F NMR (282 MHz, CDCl3)
f1 (ppm)

methyl 4-(3-(dimethyl(phenyl)silyl)-1-(naphthalen-1-yl)-1-oxopropan-2-yl)benzoate (3ka)

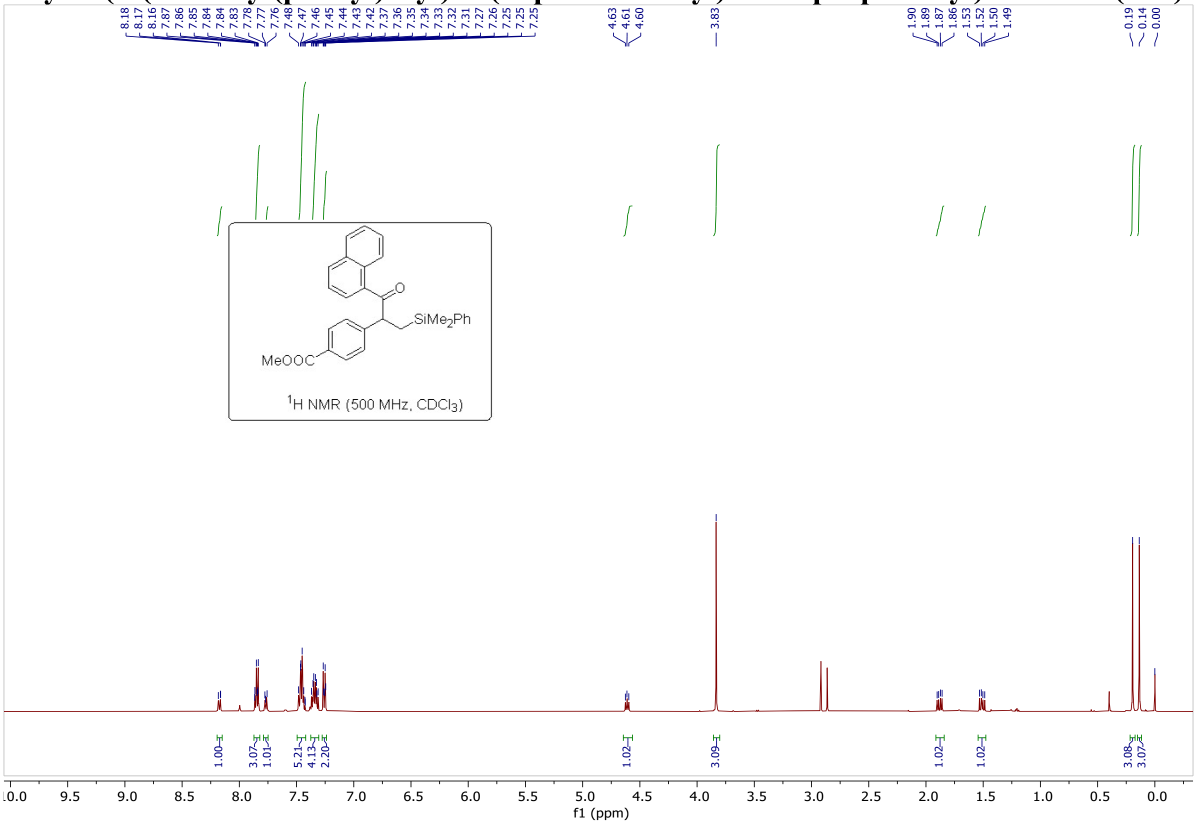

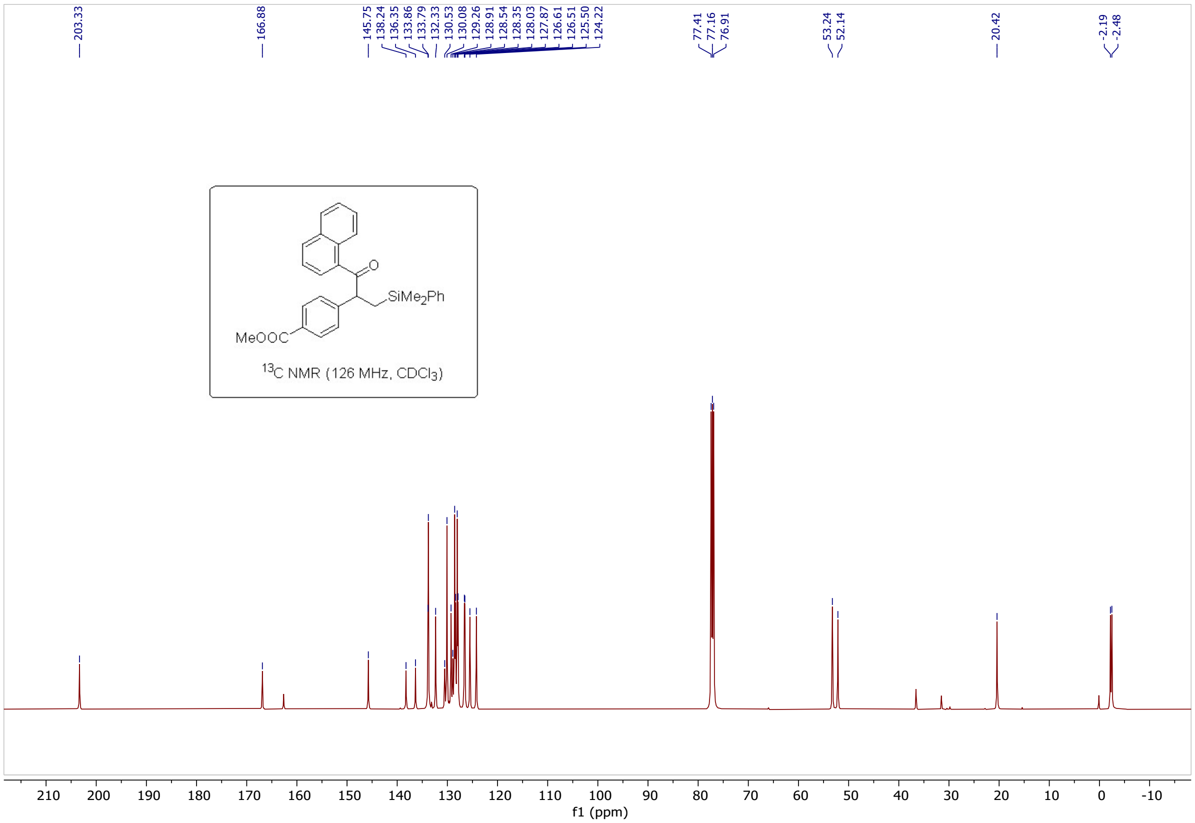

methyl 4-(3-(dimethyl(phenyl)silyl)-1-(naphthalen-2-yl)-1-oxopropan-2-yl)benzoate (3la)

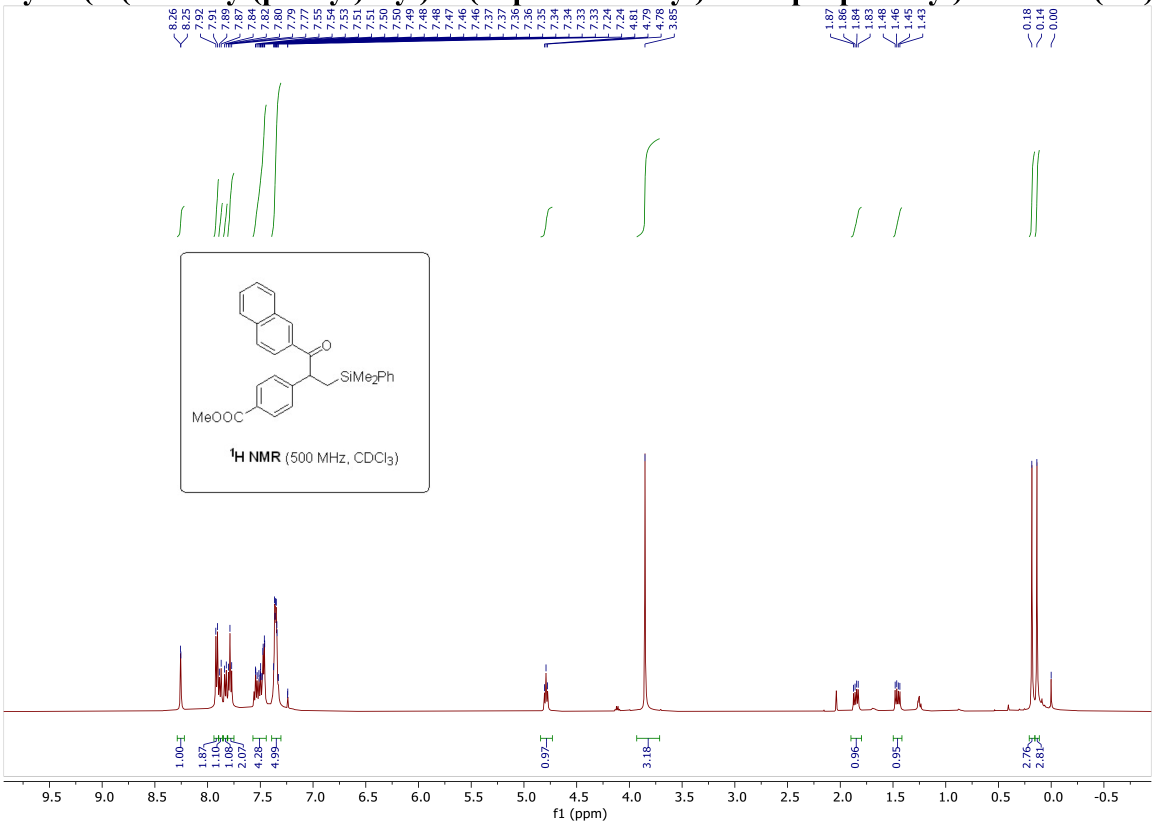

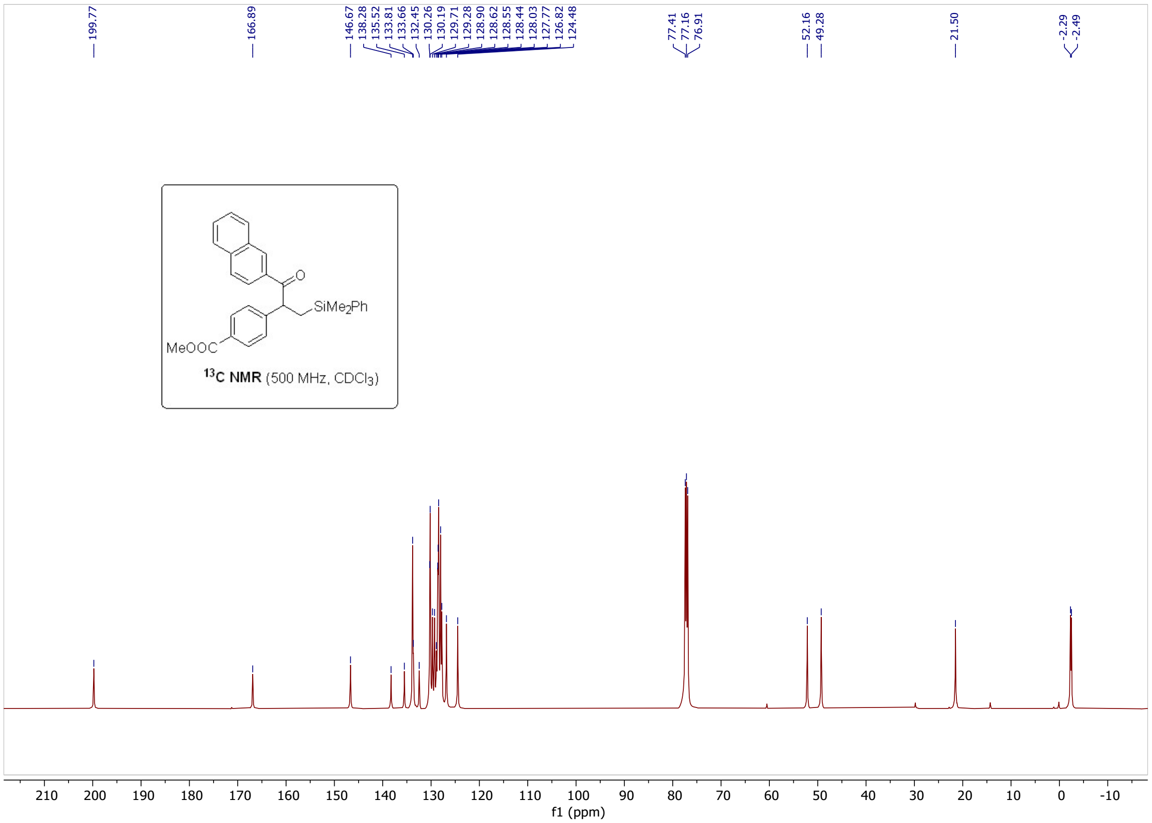

methyl 4-(1-([1,1'-biphenyl]-4-yl)-3-(dimethyl(phenyl)silyl)-1-oxopropan-2-yl)benzoate (3ma)

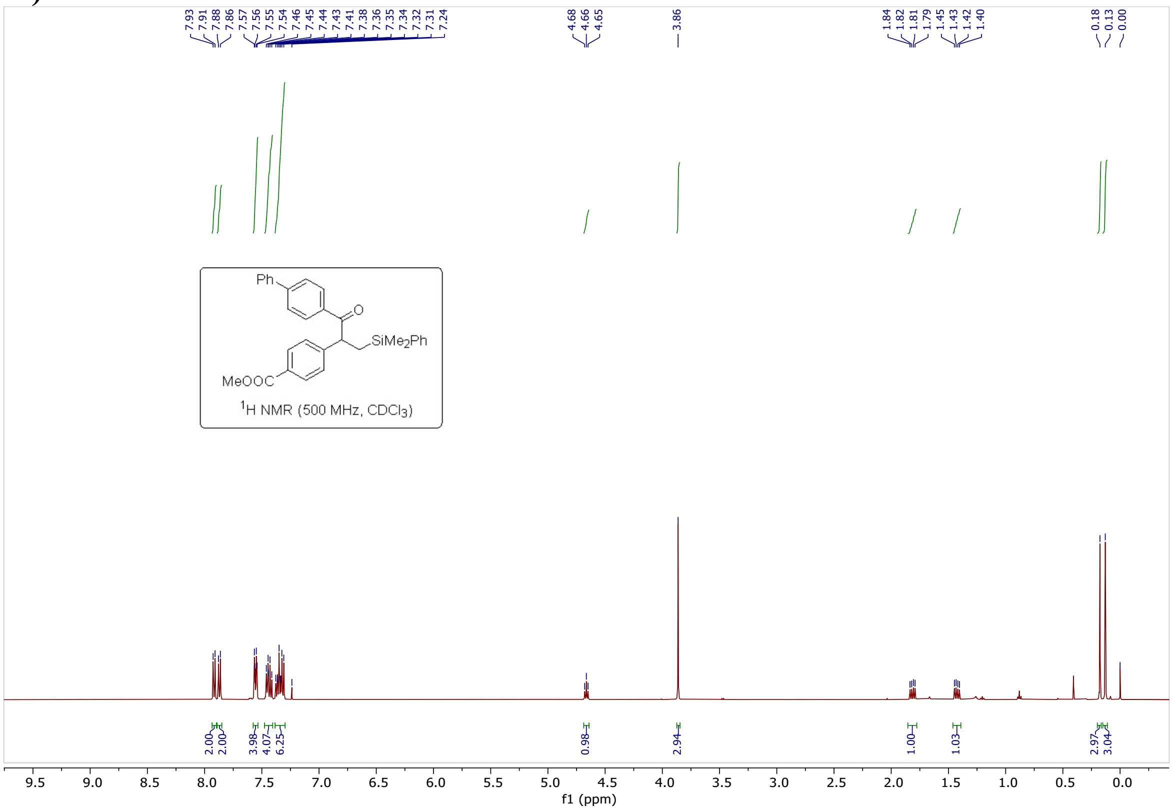

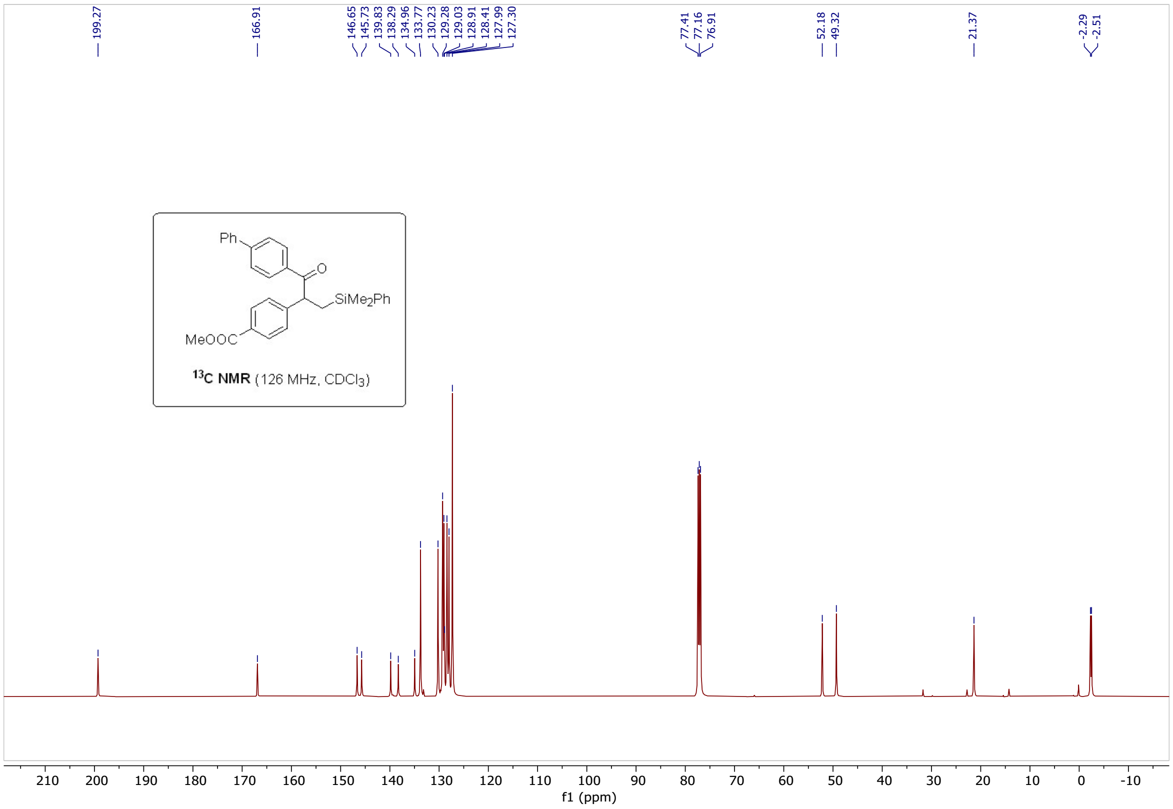

methyl 4-(1-([1,1'-biphenyl]-2-yl)-3-(dimethyl(phenyl)silyl)-1-oxopropan-2-yl)benzoate (3na)

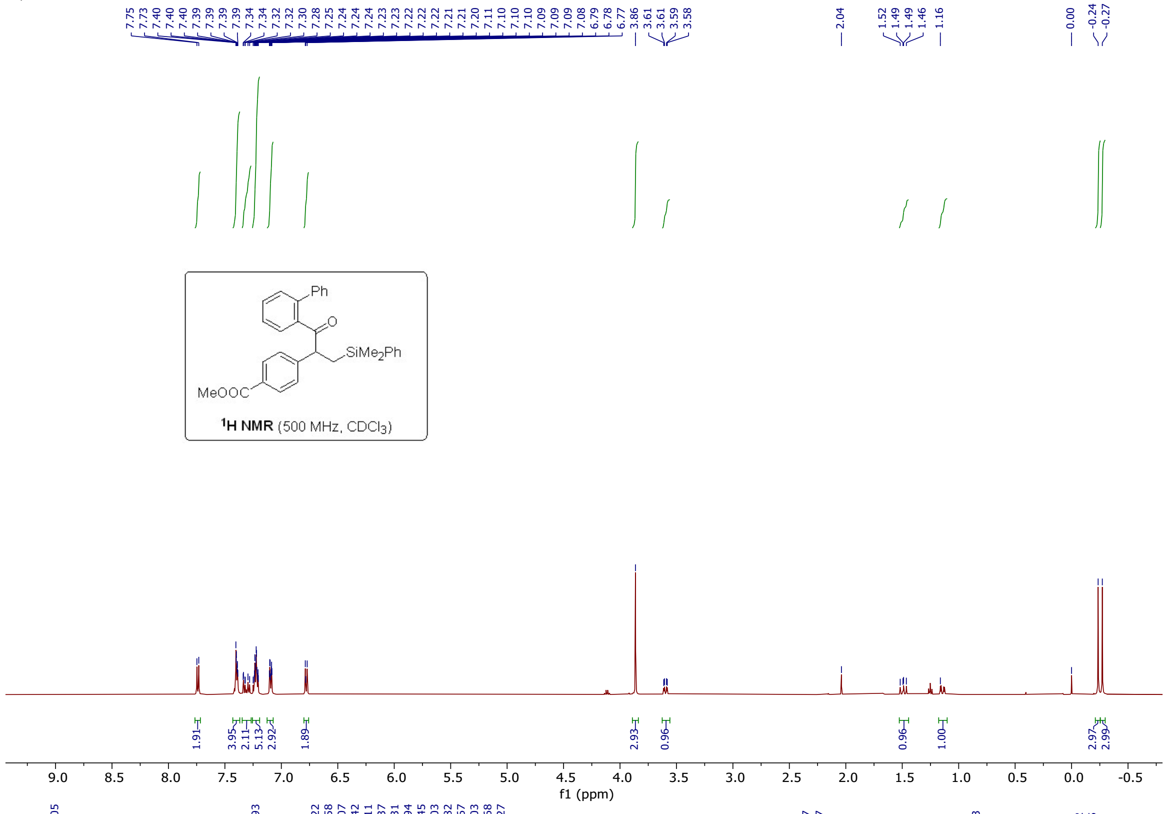

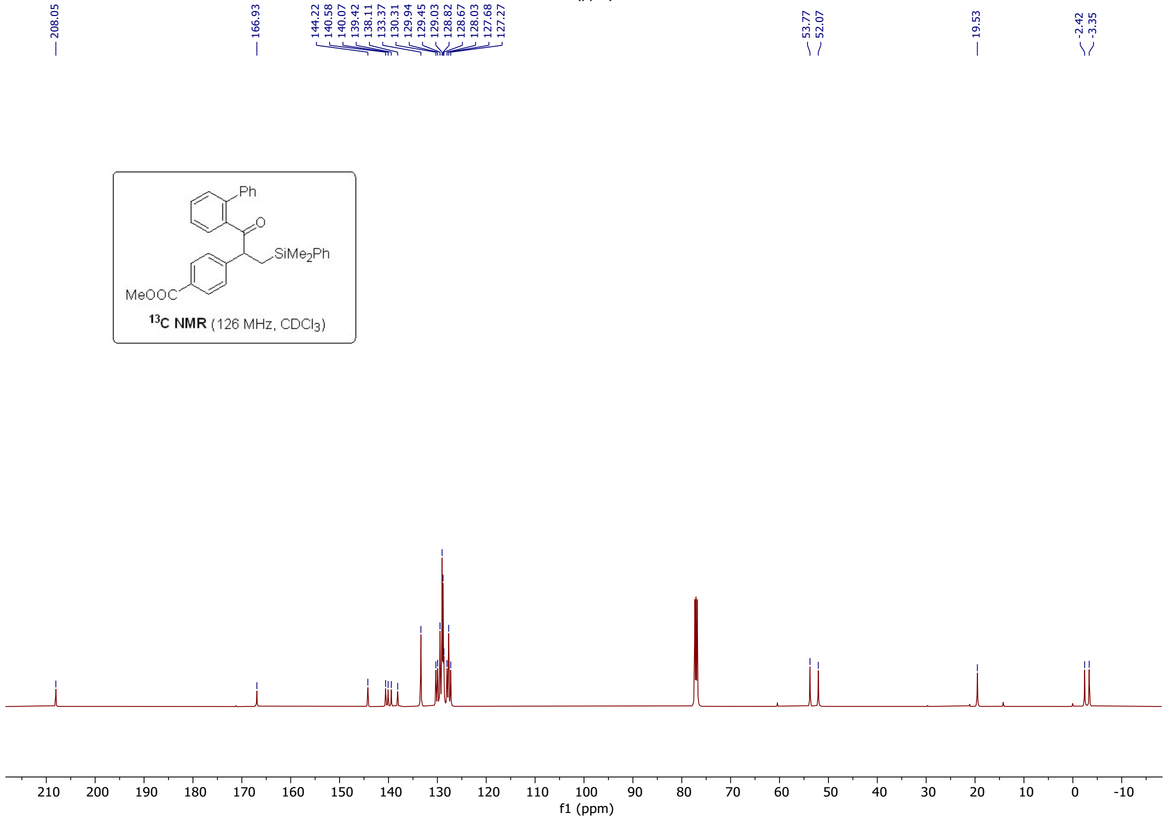

methyl 4-(3-(dimethyl(phenyl)silyl)-1-oxo-1-(thiophen-2-yl)propan-2-yl)benzoate (3oa)

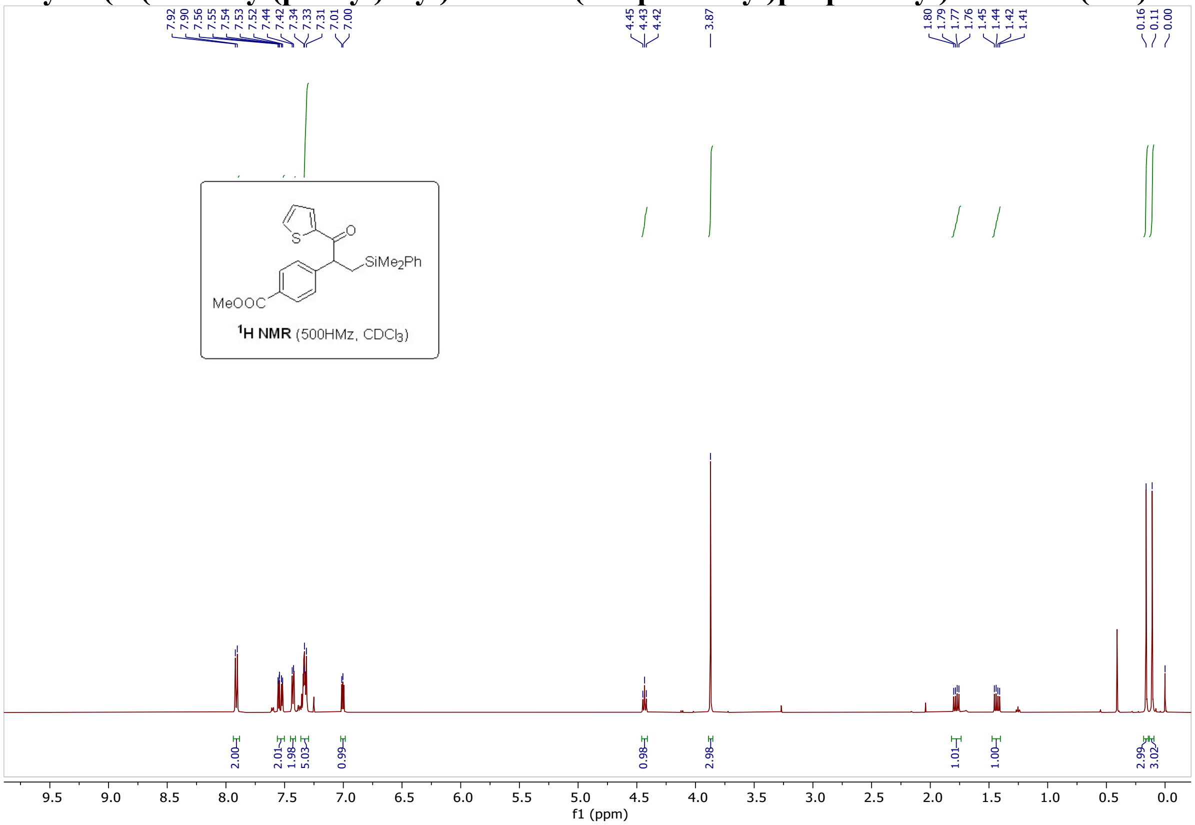

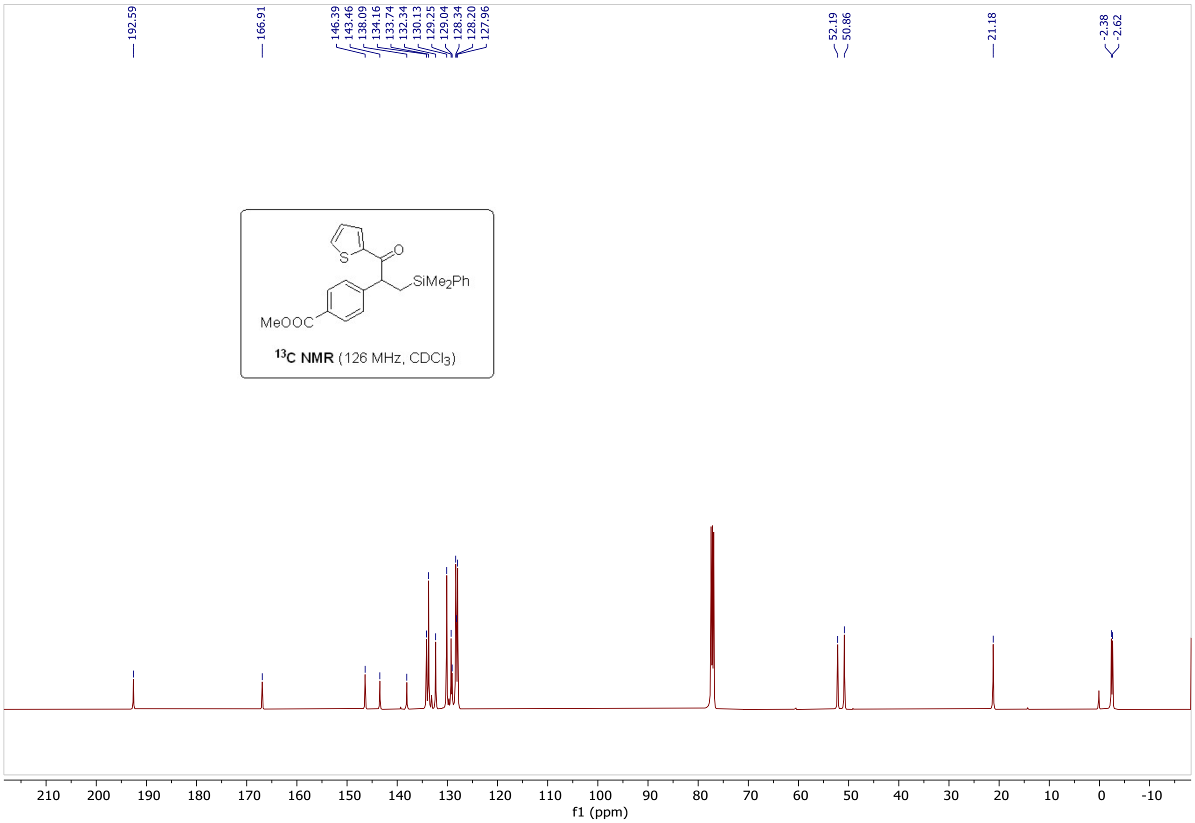

methyl 4-(1-(benzofuran-2-yl)-3-(dimethyl(phenyl)silyl)-1-oxopropan-2-yl)benzoate (3pa)

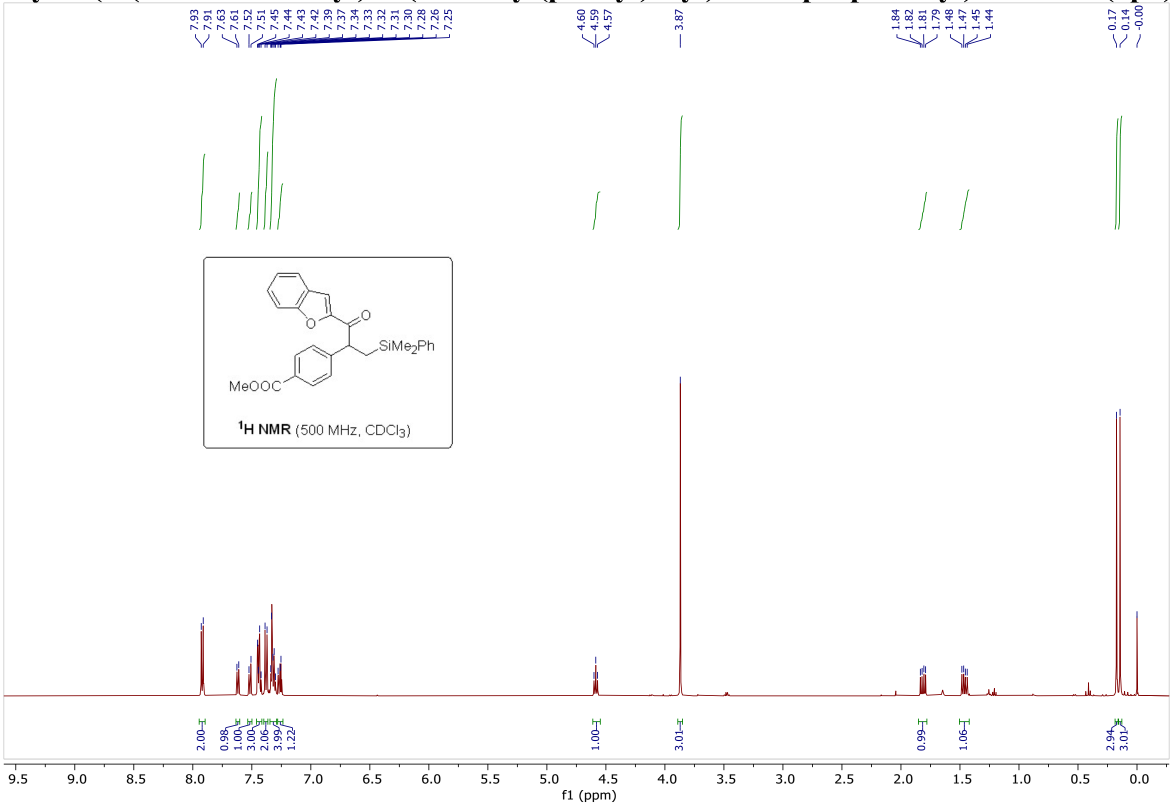

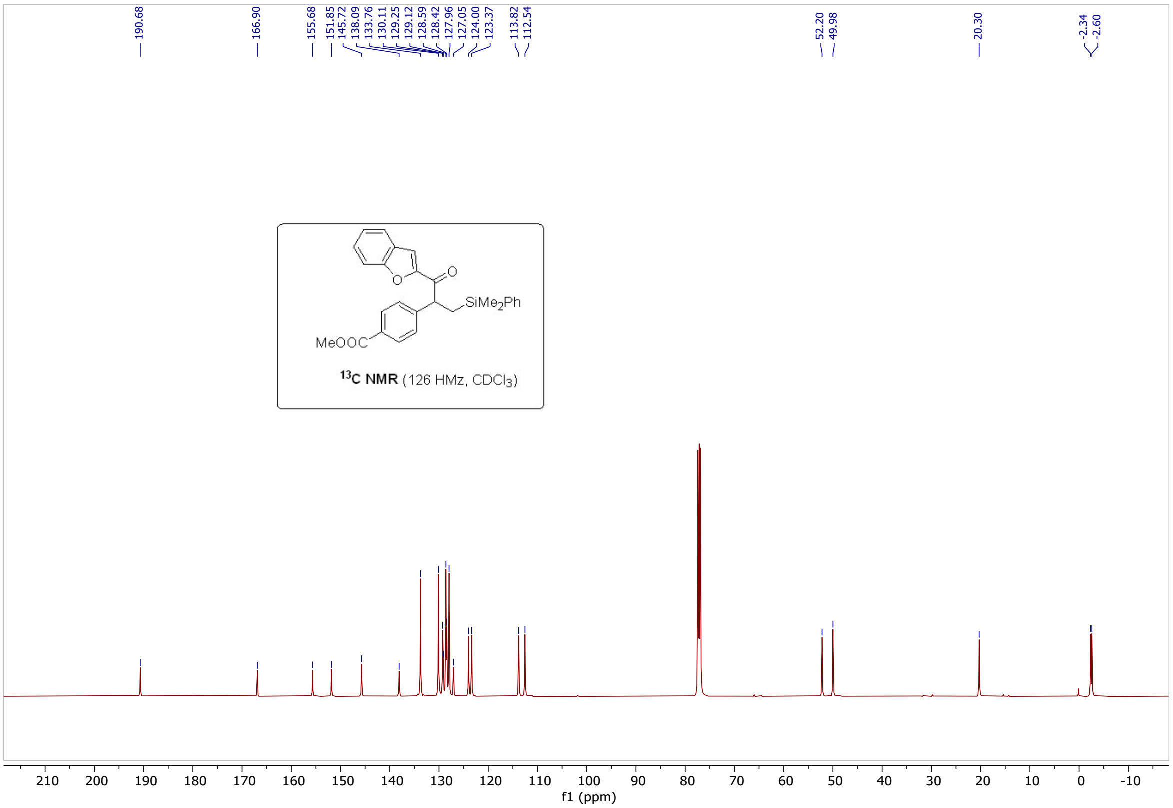

methyl 4-(1-(dimethyl(phenyl)silyl)-3-oxo-5-phenylpentan-2-yl)benzoate (3qa)

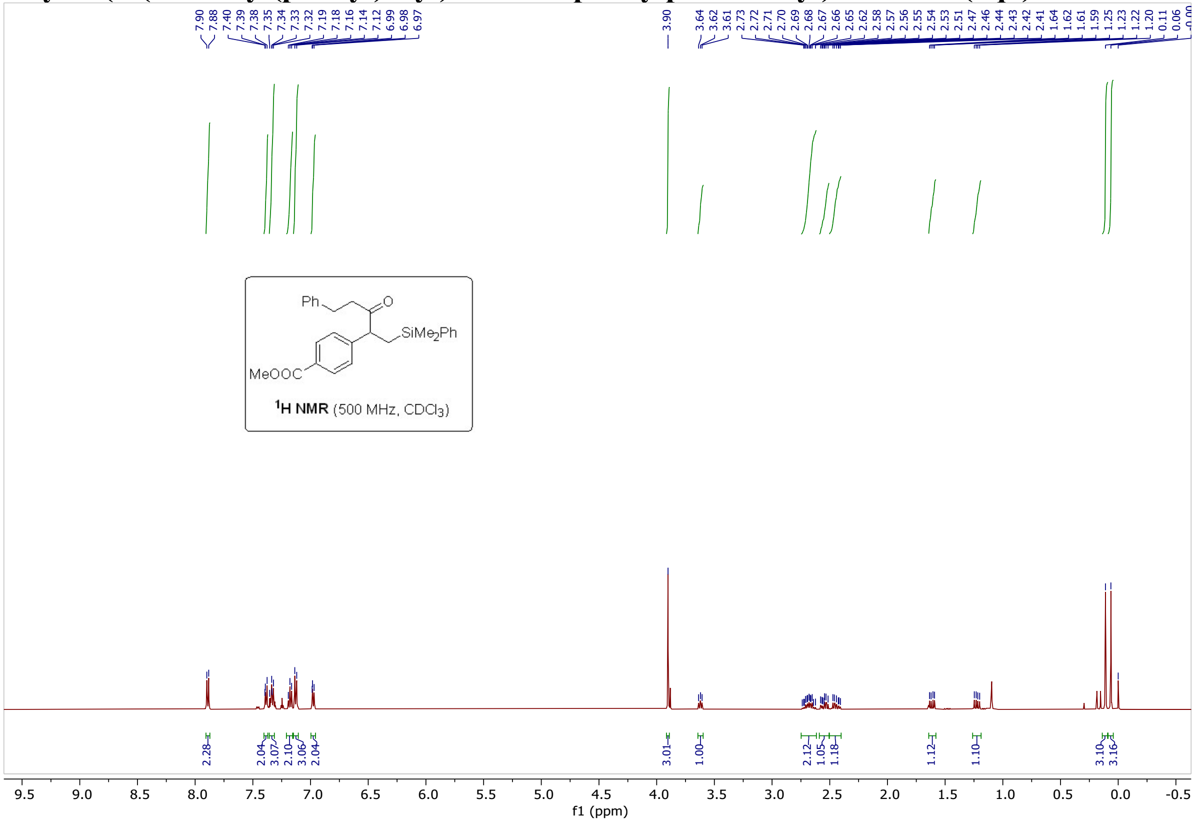

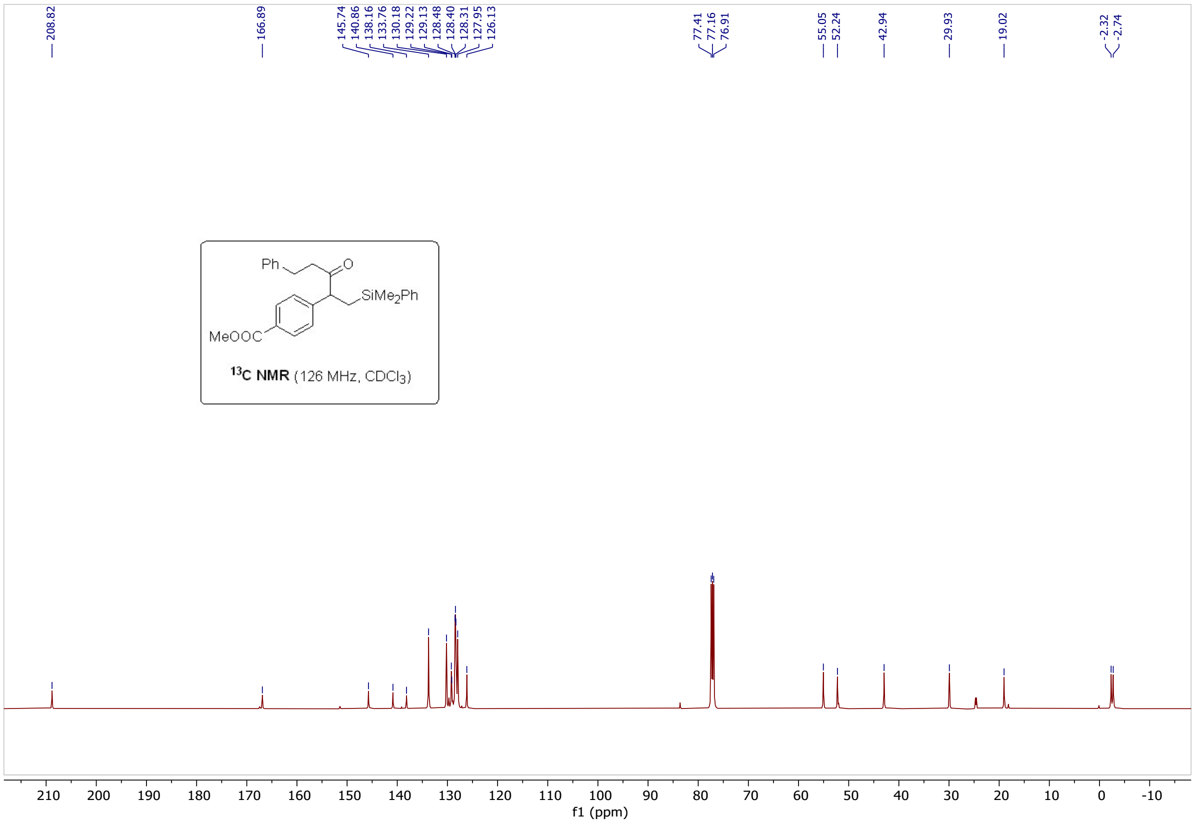

methyl 4-(3-(dimethyl(phenyl)silyl)-1-oxo-1-(2-phenylcyclopropyl)propan-2-yl)benzoate (3ra)

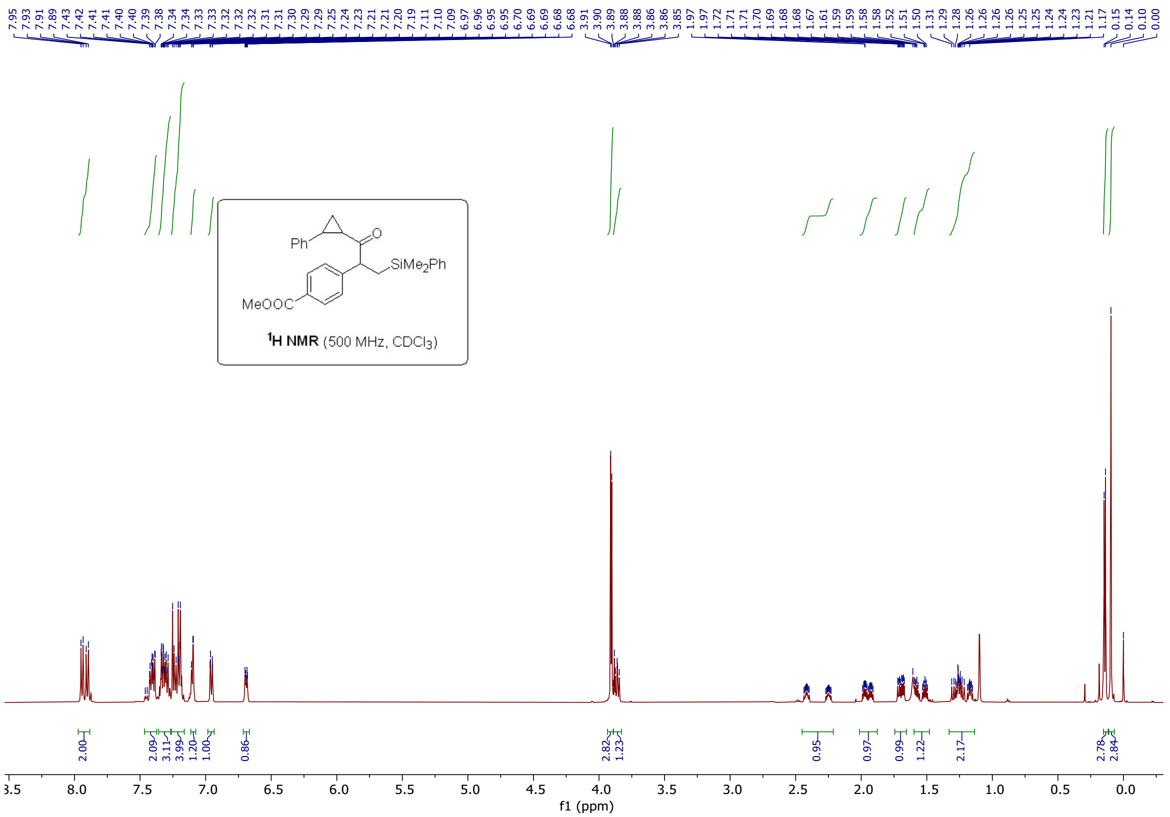

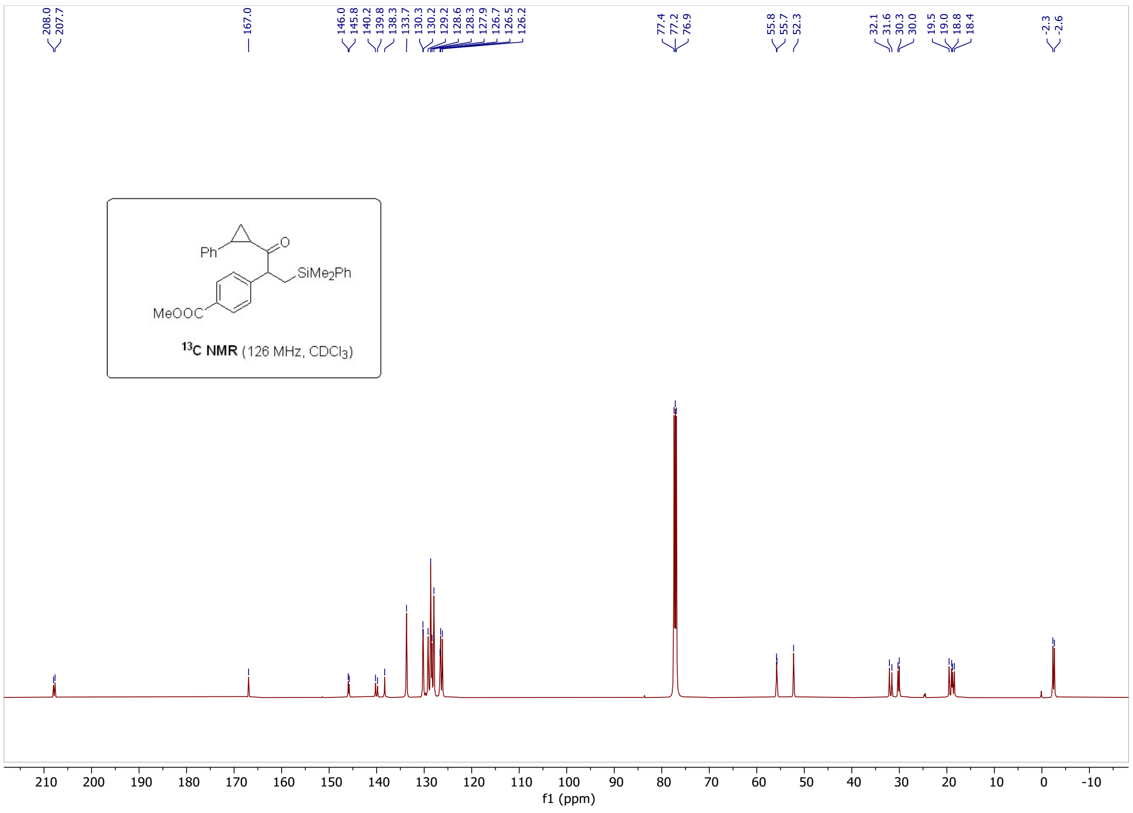

methyl 4-(1-(3,4-diethoxyphenyl)-3-(dimethyl(phenyl)silyl)-1-oxopropan-2-yl)benzoate (3sa)

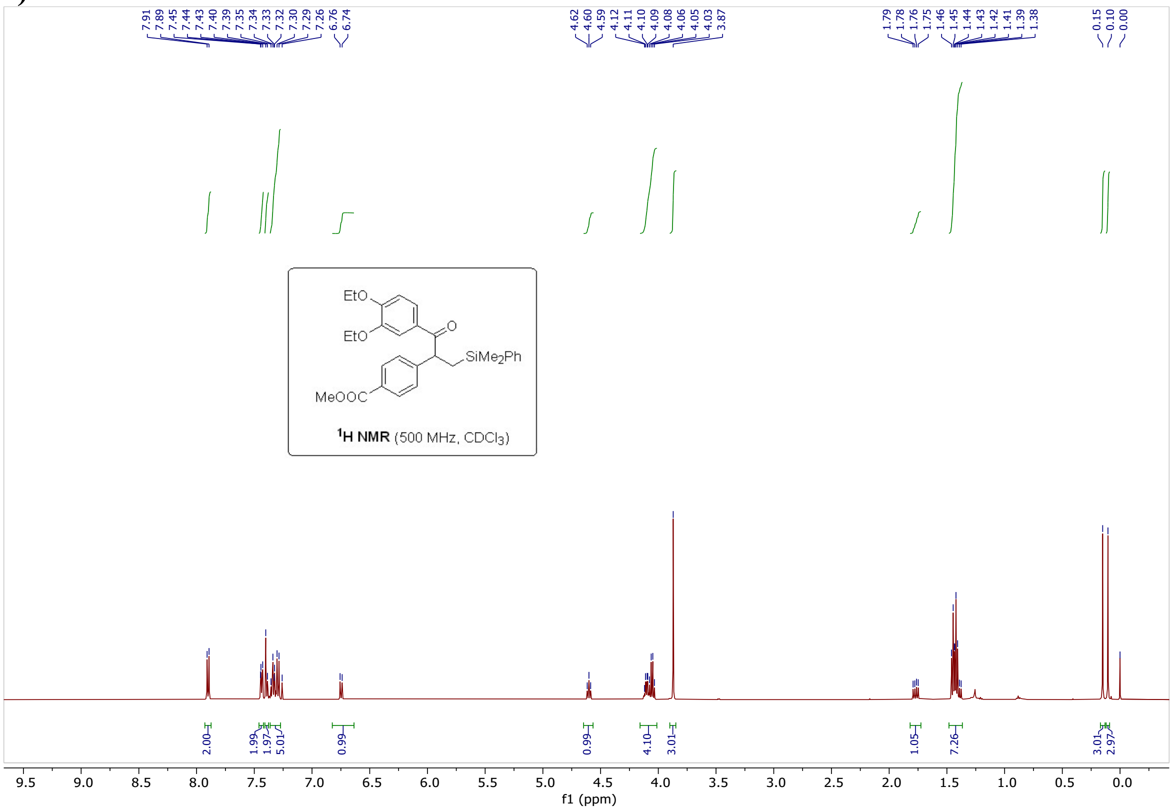

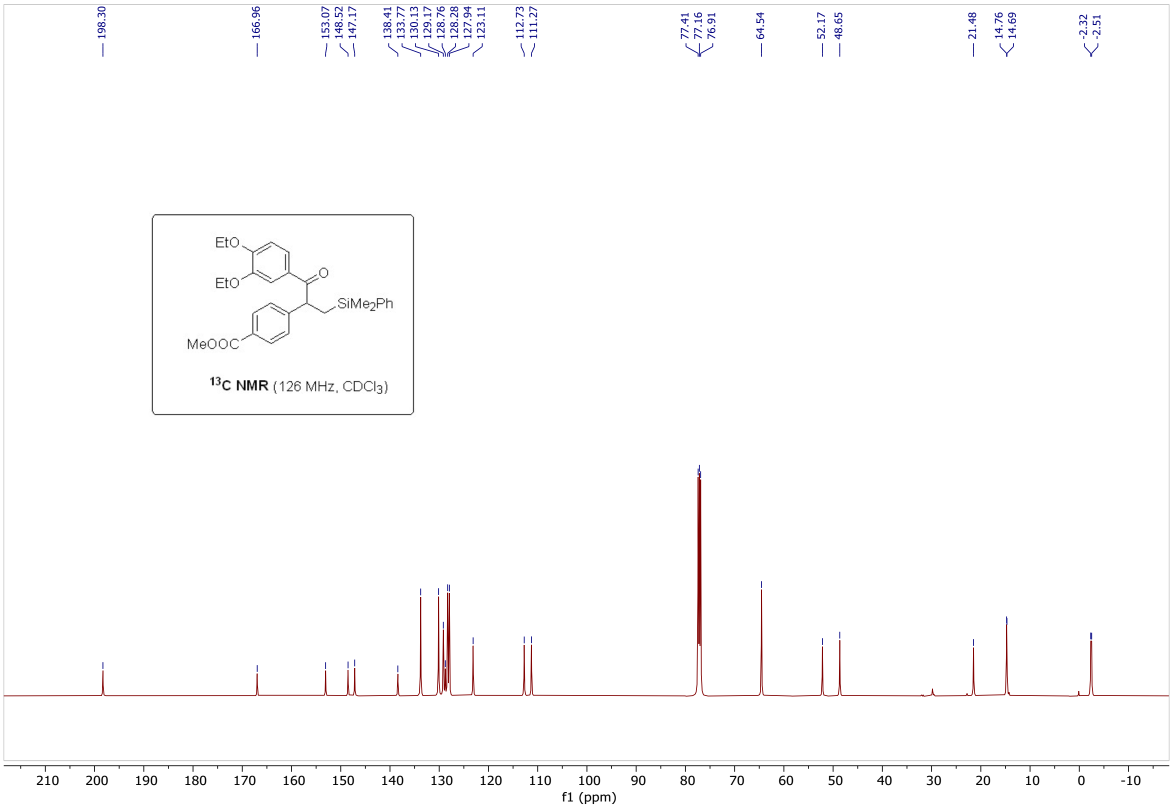

methyl 4-(1-(4-bromo-3-methylphenyl)-3-(dimethyl(phenyl)silyl)-1-oxopropan-2-yl)benzoate (3ta)

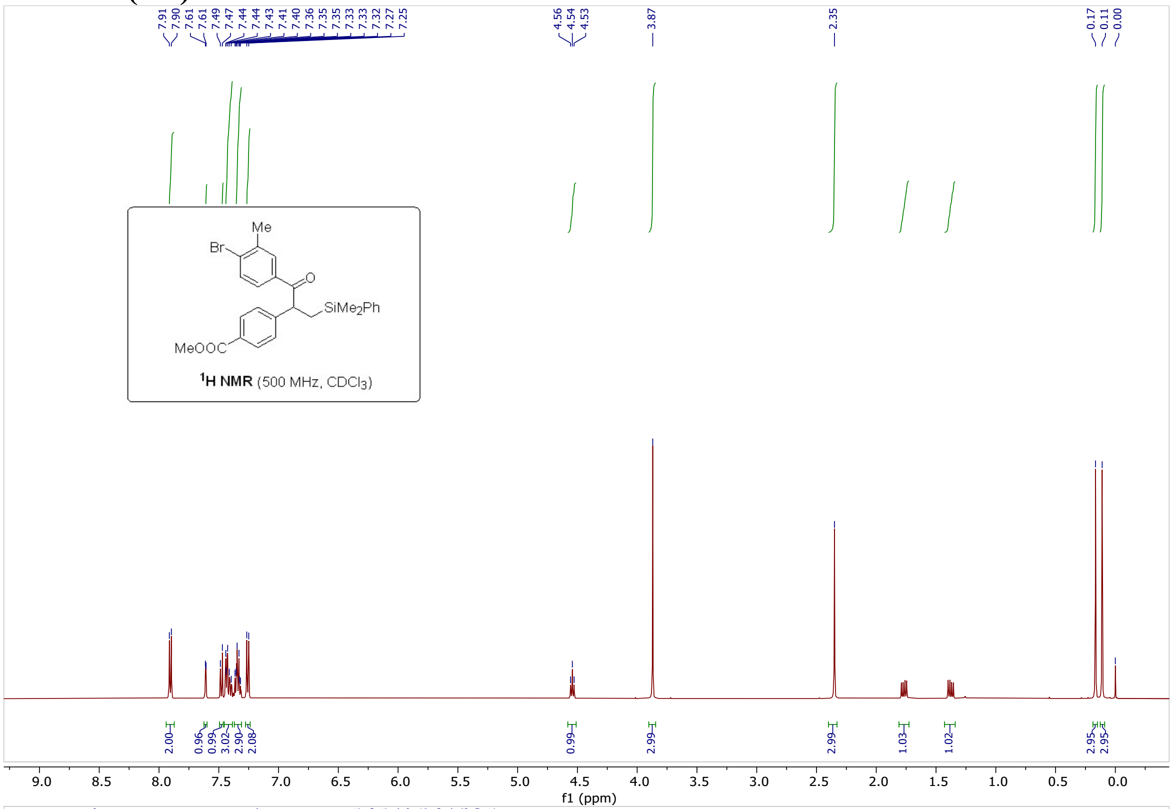

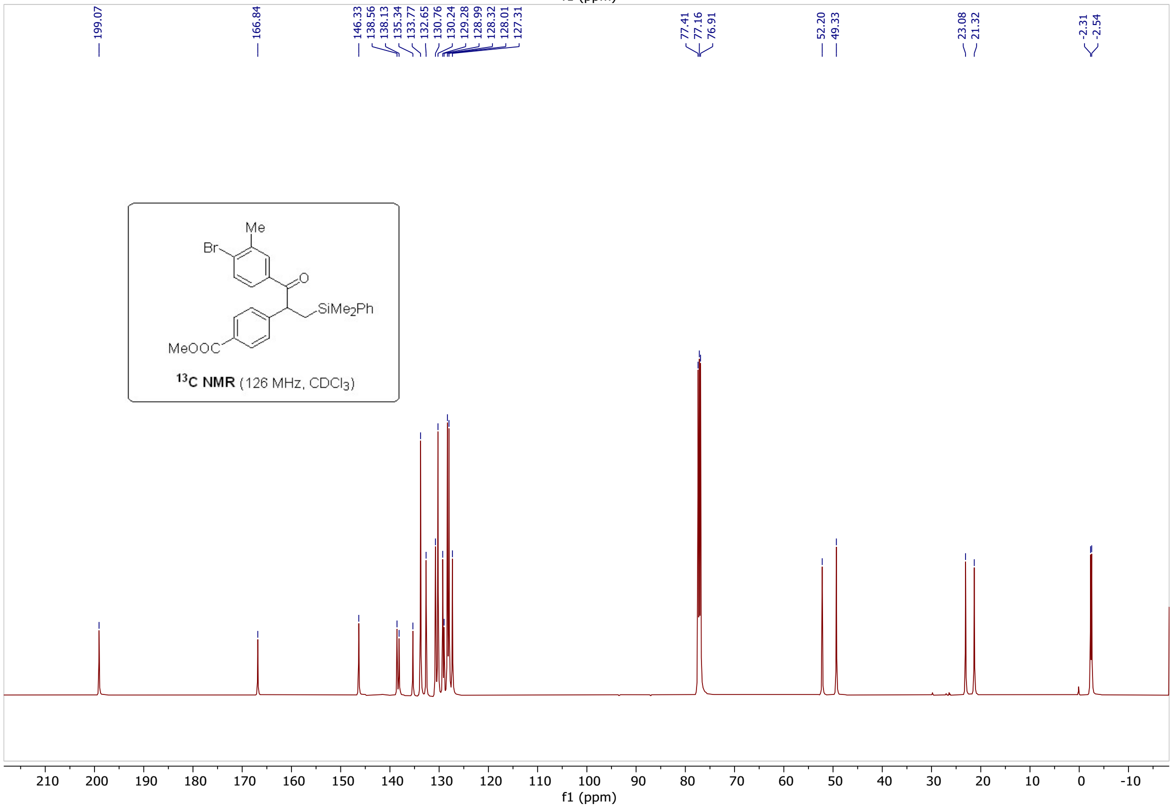

methyl 4-(1-(4-((*tert*-butyldimethylsilyl)oxy)phenyl)-3-(dimethyl(phenyl)silyl)-1-oxopropan-2-yl)benzoate (3ua)

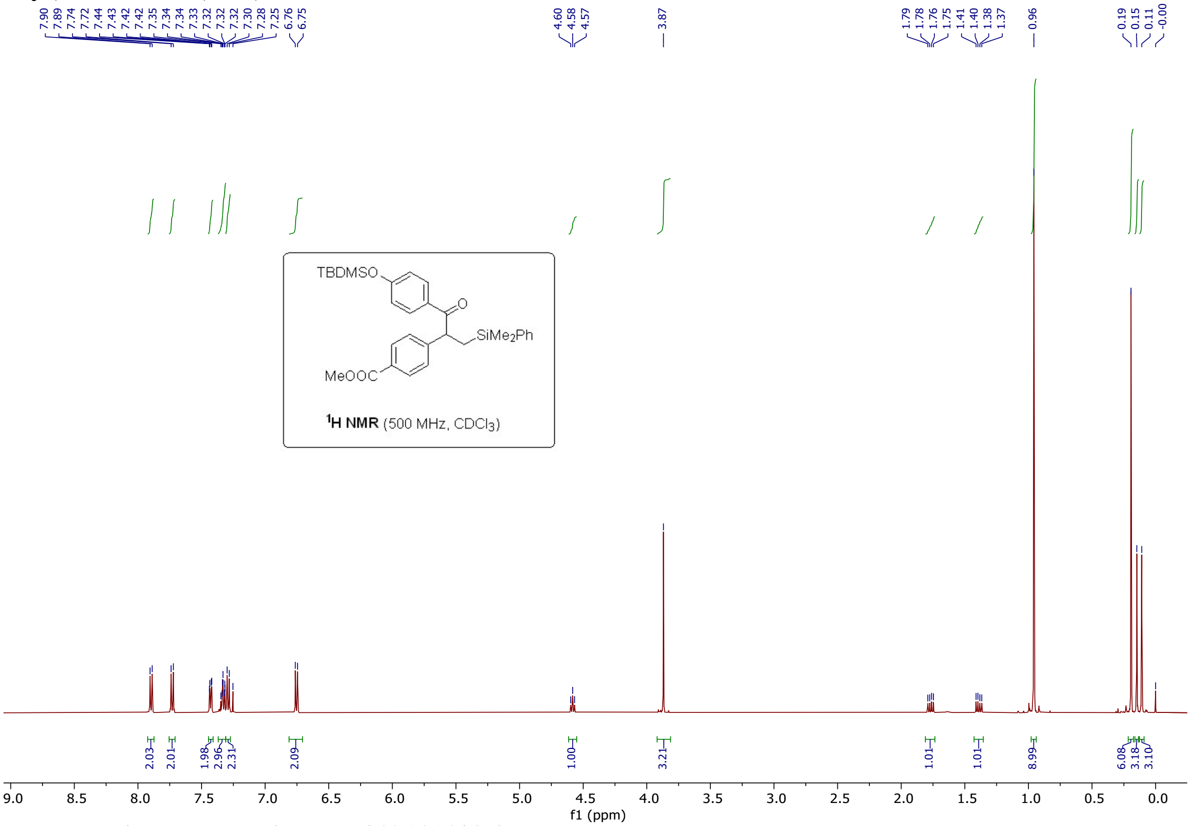

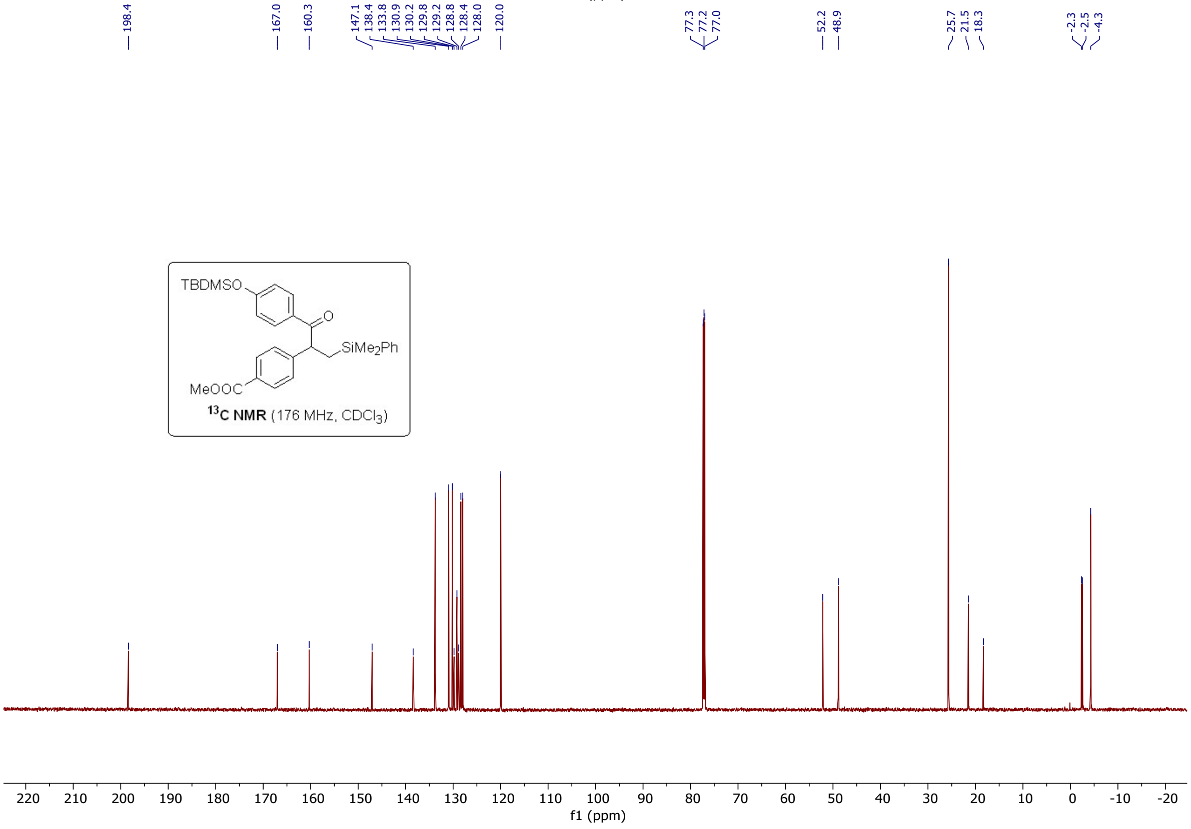

methyl 4-(1-(4-(butylcarbamoyl)phenyl)-3-(dimethyl(phenyl)silyl)-1-oxopropan-2-yl)benzoate (3va)

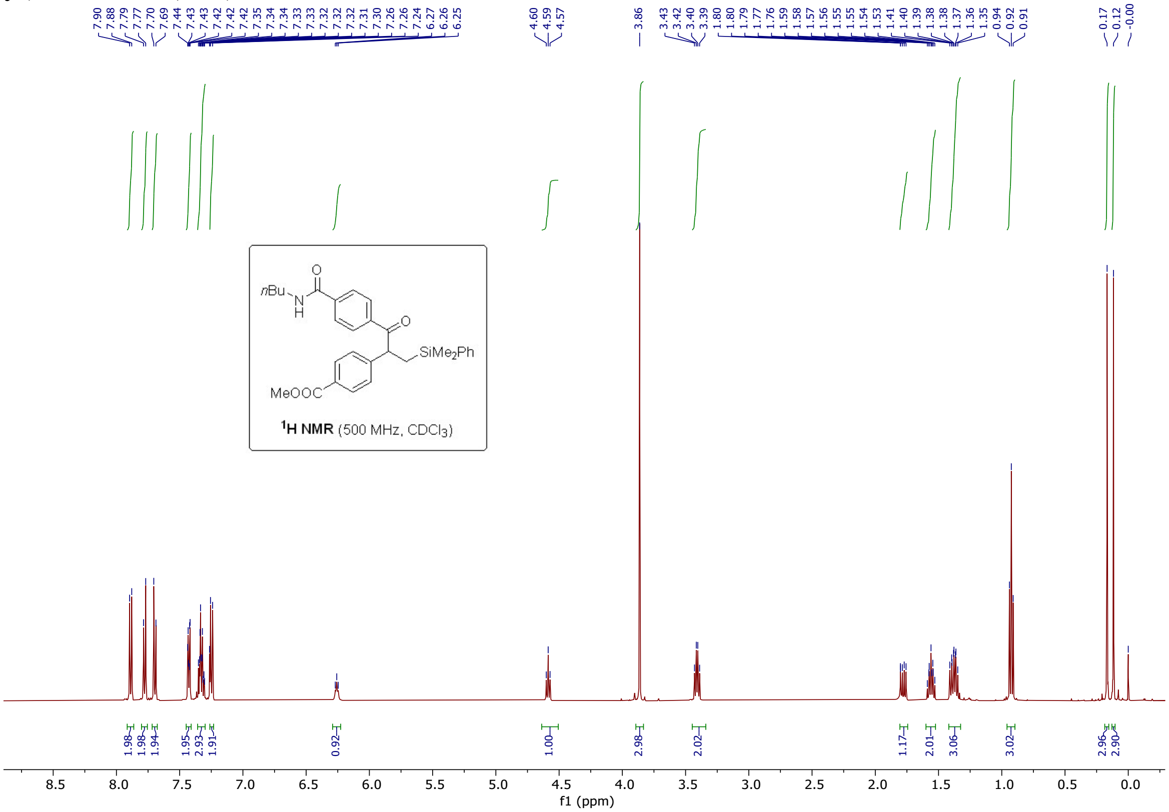

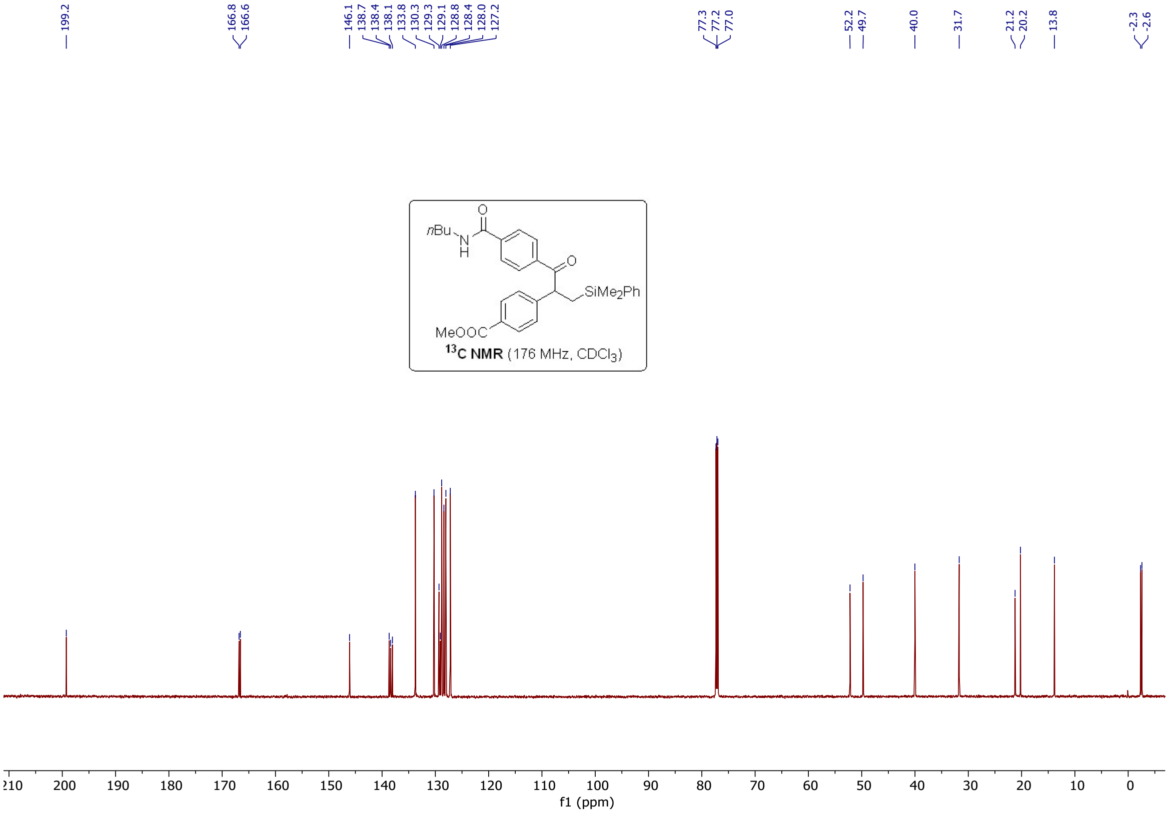

methyl 4-(1-(4-((*tert*-butoxycarbonyl)amino)phenyl)-3-(dimethyl(phenyl)silyl)-1-oxopropan-2-yl)benzoate (3wa)

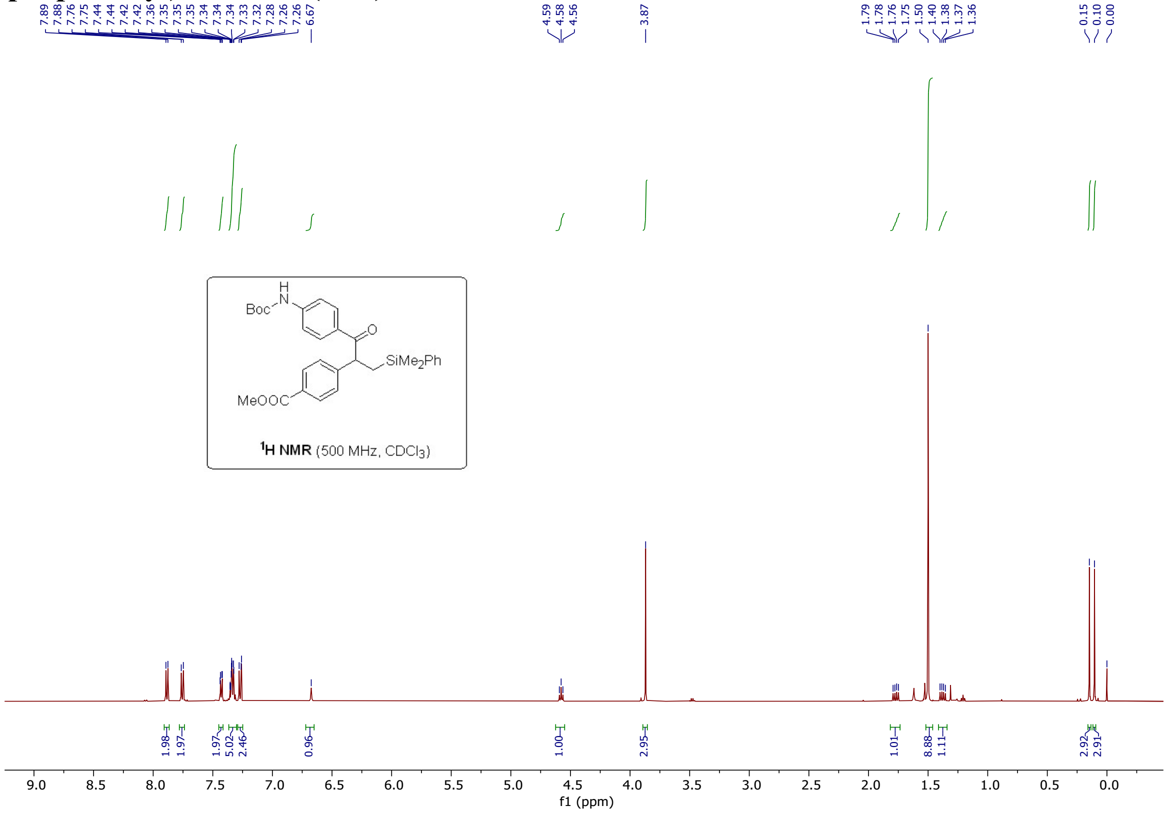

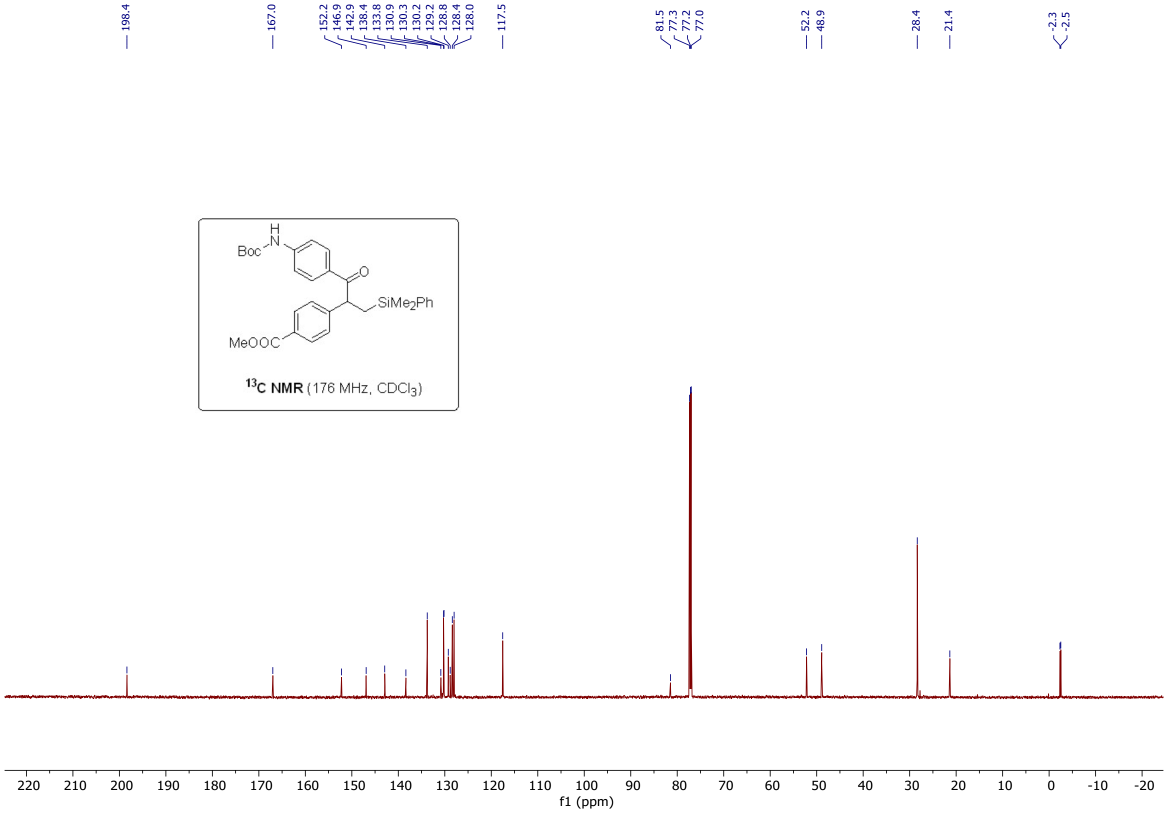

methyl 4-(3-(dimethyl(phenyl)silyl)-1-(4-(hydroxymethyl)phenyl)-1-oxopropan-2-yl)benzoate (3xa)

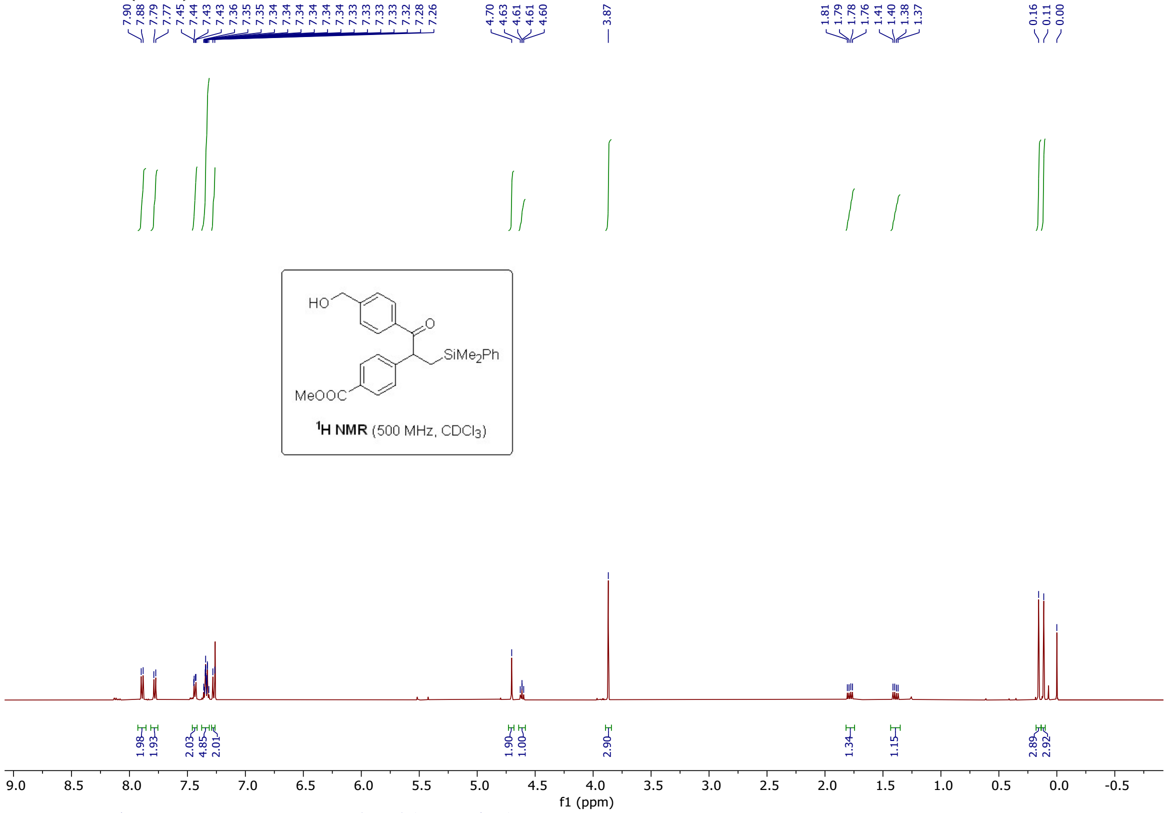

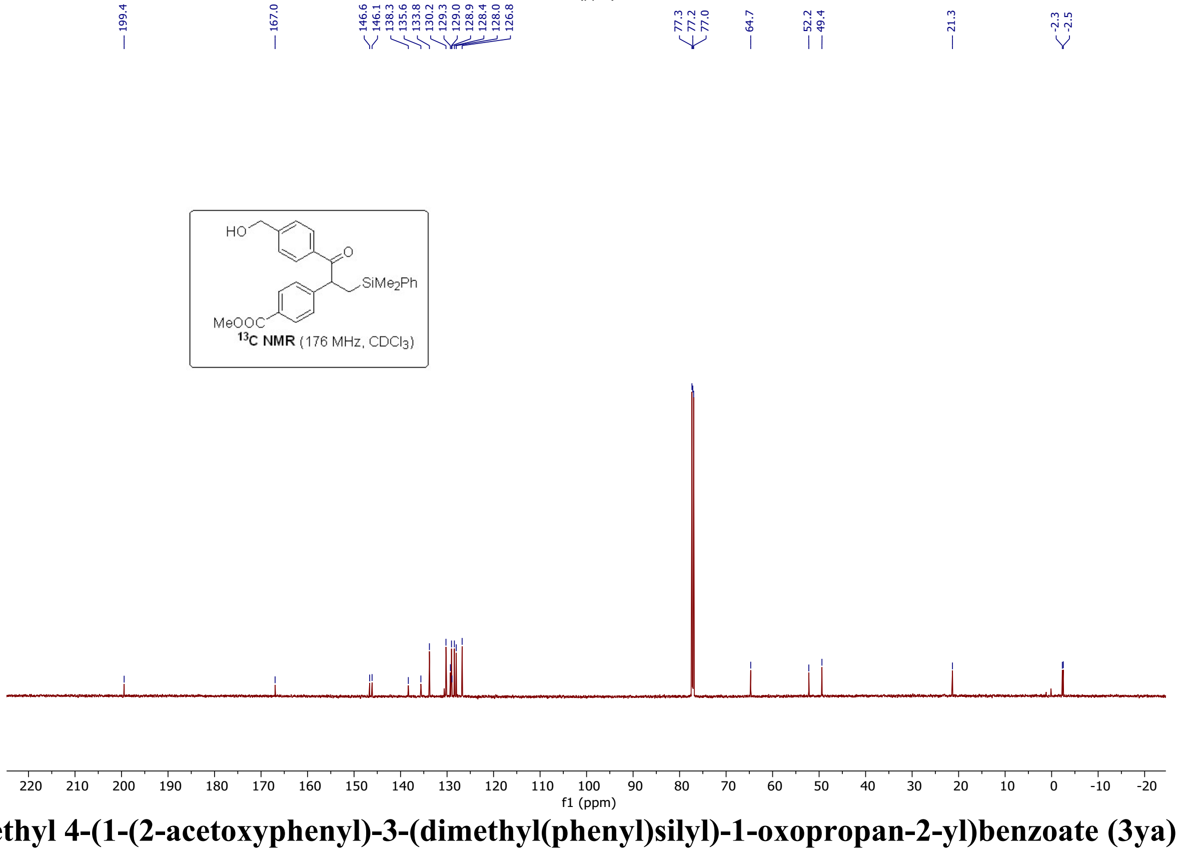

methyl 4-(1-(2-acetoxyphenyl)-3-(dimethyl(phenyl)silyl)-1-oxopropan-2-yl)benzoate (3ya)

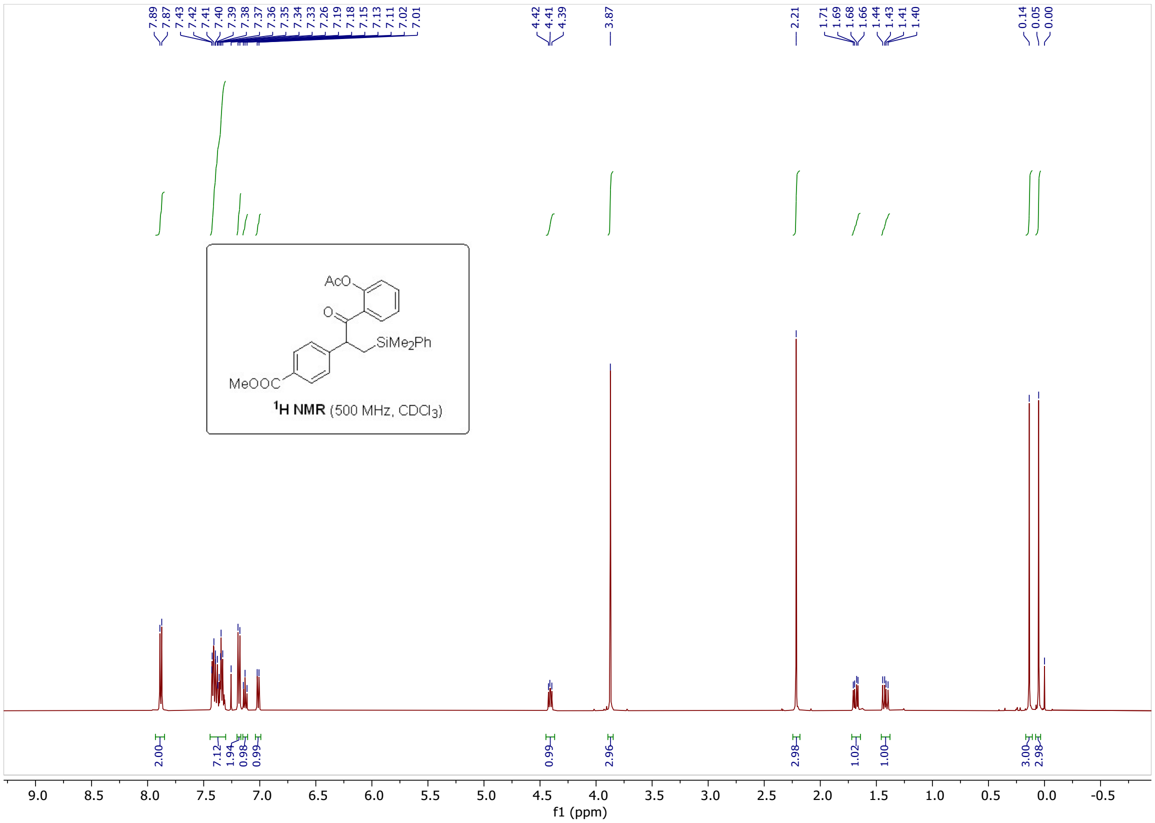
7.89
7.87
7.43
7.42
7.41
7.40
7.39
7.38
7.37
7.36
7.35
7.34
7.33
7.26
7.19
7.18
7.15
7.13
7.11
7.02
7.01
4.42
4.41
4.39
3.87
2.21
1.71
1.69
1.68
1.66
1.44
1.43
1.41
1.40
0.14
0.05
0.00
AcO
O
SiMe2Ph
MeOOC
1H NMR (500 MHz, CDCl3)
2.00
7.12
1.94
0.98
0.99
0.99
2.96
2.98
1.02
1.00
3.00
2.98
9.0
8.5
8.0
7.5
7.0
6.5
6.0
5.5
5.0
4.5
4.0
3.5
3.0
2.5
2.0
1.5
1.0
0.5
0.0
-0.5
f1 (ppm)

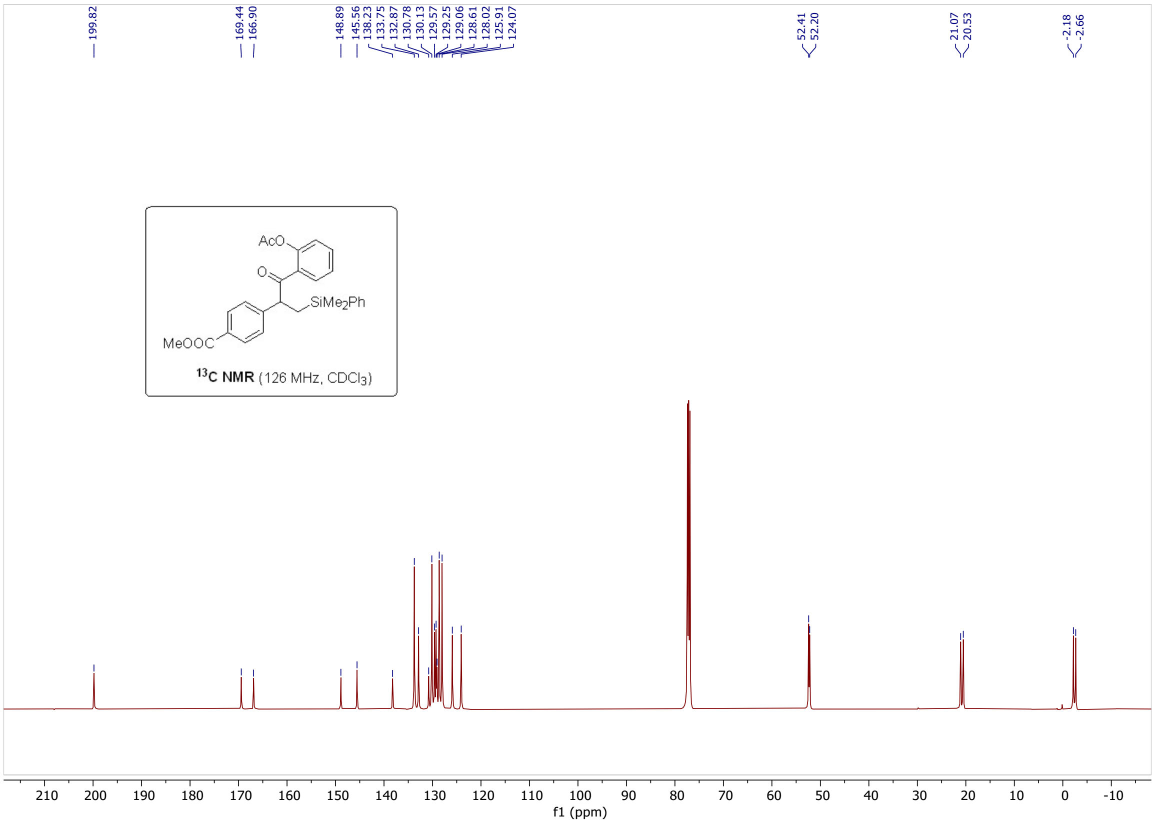
199.82
169.44
166.90
148.89
145.56
138.23
133.75
132.87
130.78
130.13
129.57
129.25
129.06
128.61
128.02
125.91
124.07
52.41
52.20
21.07
20.53
-2.18
-2.66
AcO
O
SiMe2Ph
MeOOC
13C NMR (126 MHz, CDCl3)
210
200
190
180
170
160
150
140
130
120
110
100
90
80
70
60
50
40
30
20
10
0
-10
f1 (ppm)

methyl 4-(1-(2-(3-cyano-4-isobutoxyphenyl)-4-methylthiazol-5-yl)-3-(dimethyl(phenyl)silyl)-1-oxopropan-2-yl)benzoate (3za)

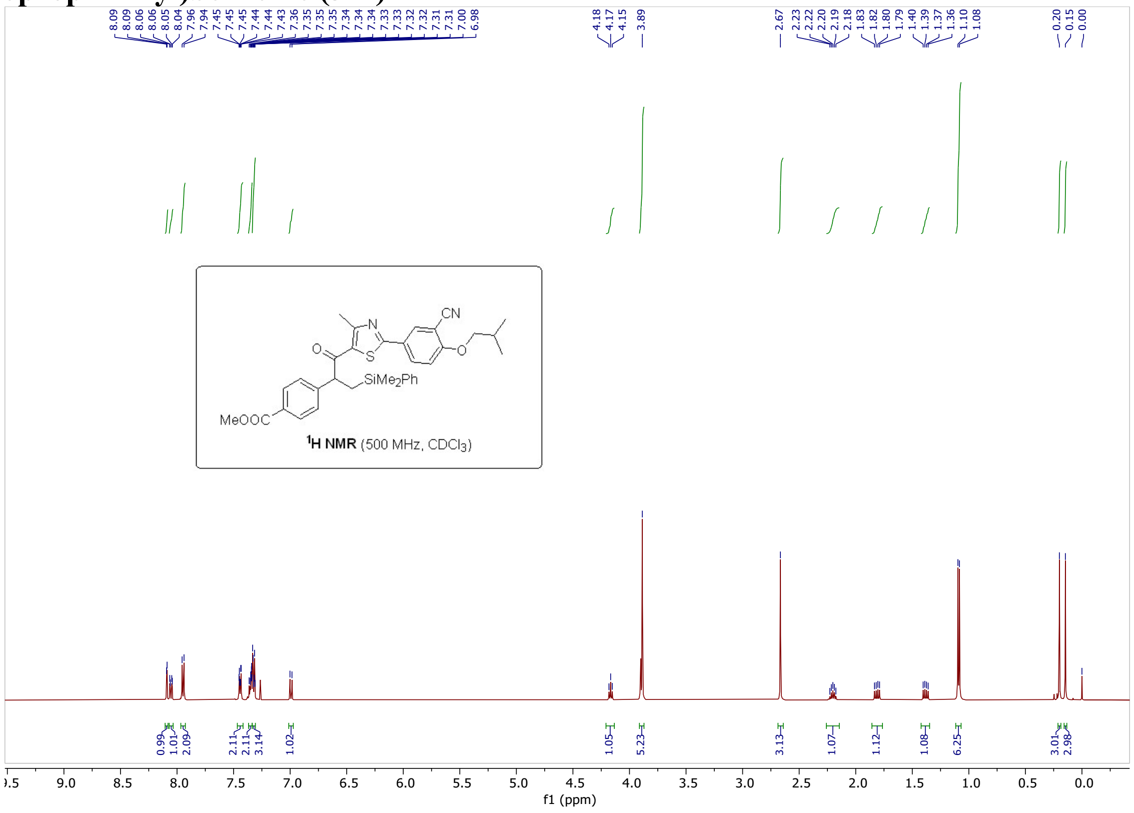

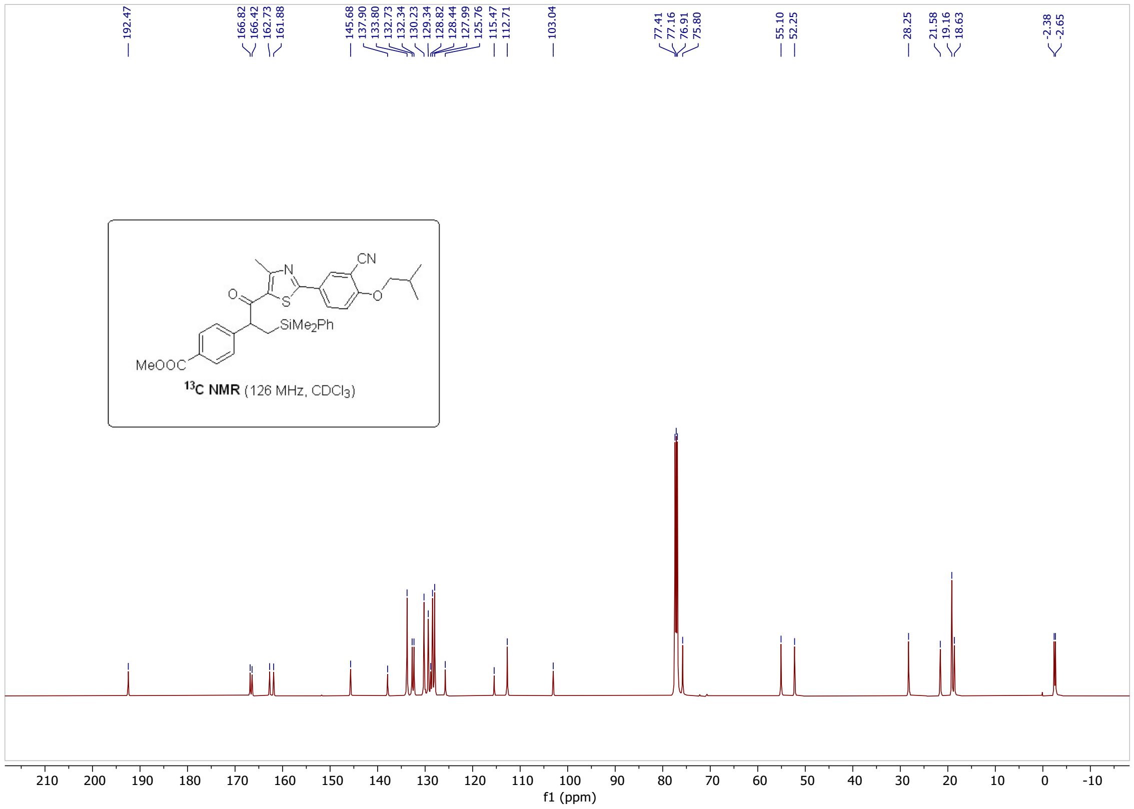

methyl 4-(3-(dimethyl(phenyl)silyl)-1-(4-(N,N-dipropylsulfamoyl)phenyl)-1-oxopropan-2-yl)benzoate (3zaa)

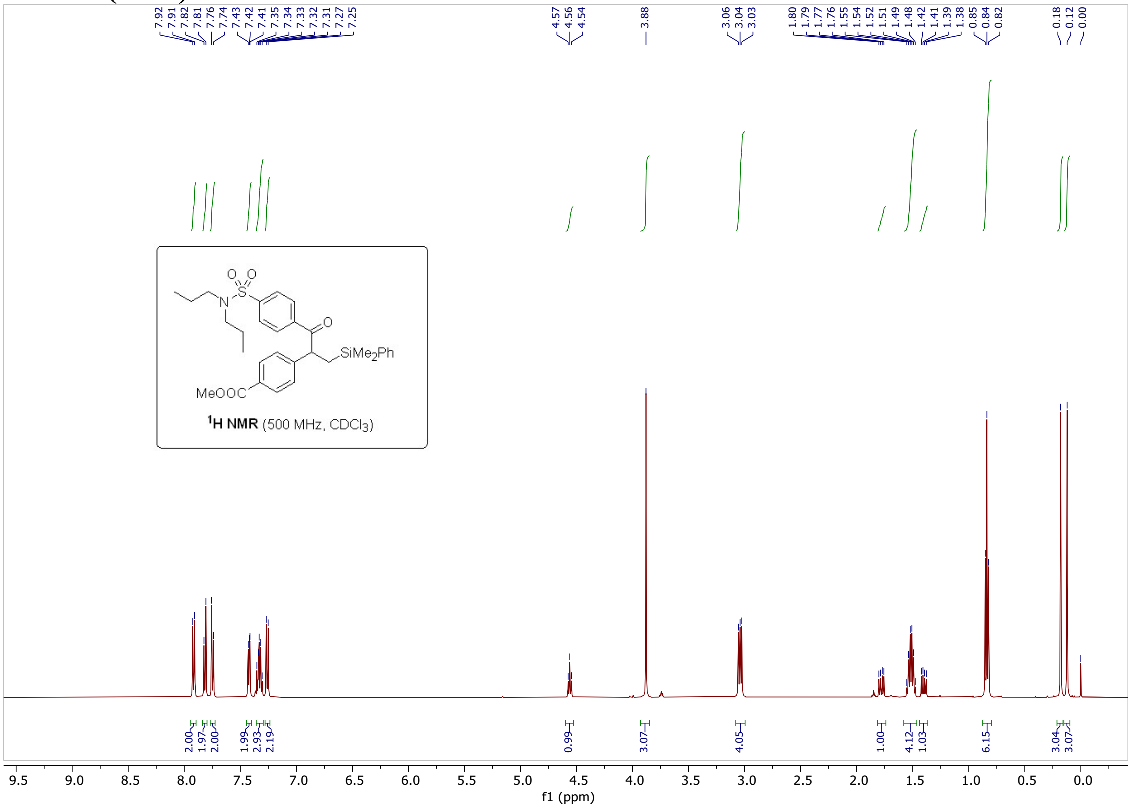

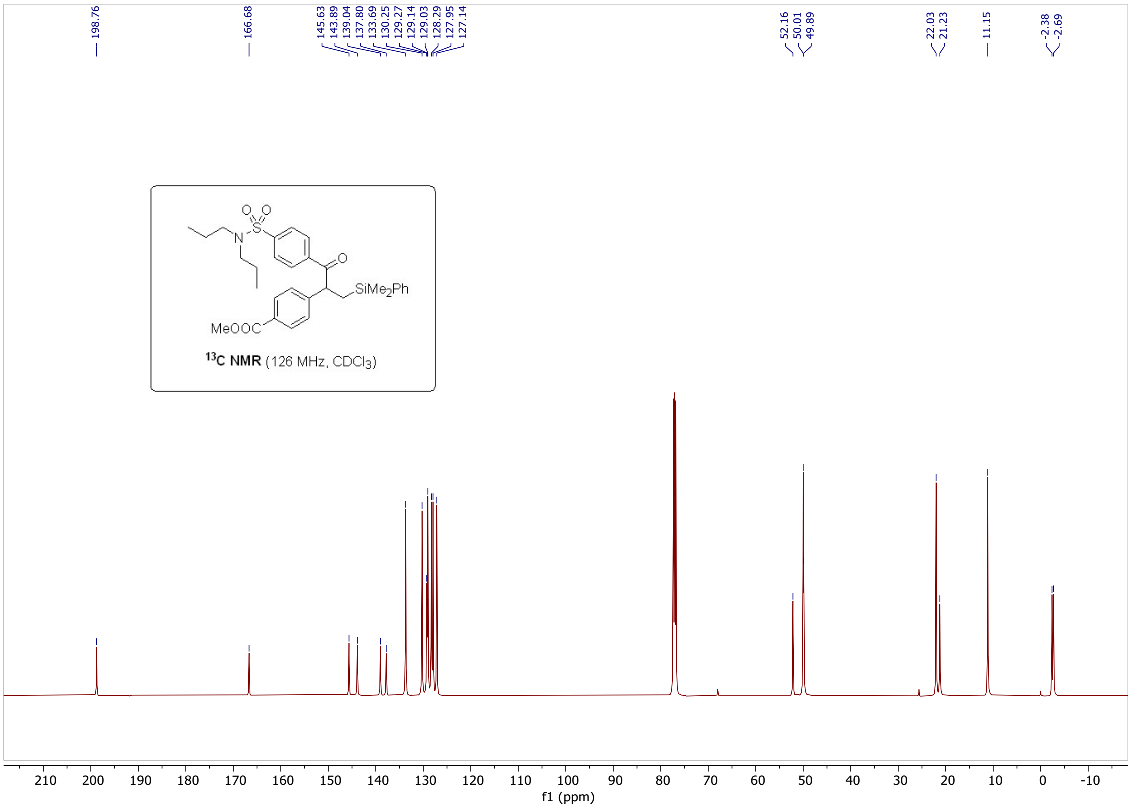

methyl 4-(1-(6-(3-(adamantan-1-yl)-4-methoxyphenyl)naphthalen-2-yl)-3-(dimethyl(phenyl)silyl)-1-oxopropan-2-yl)benzoate (3zba)

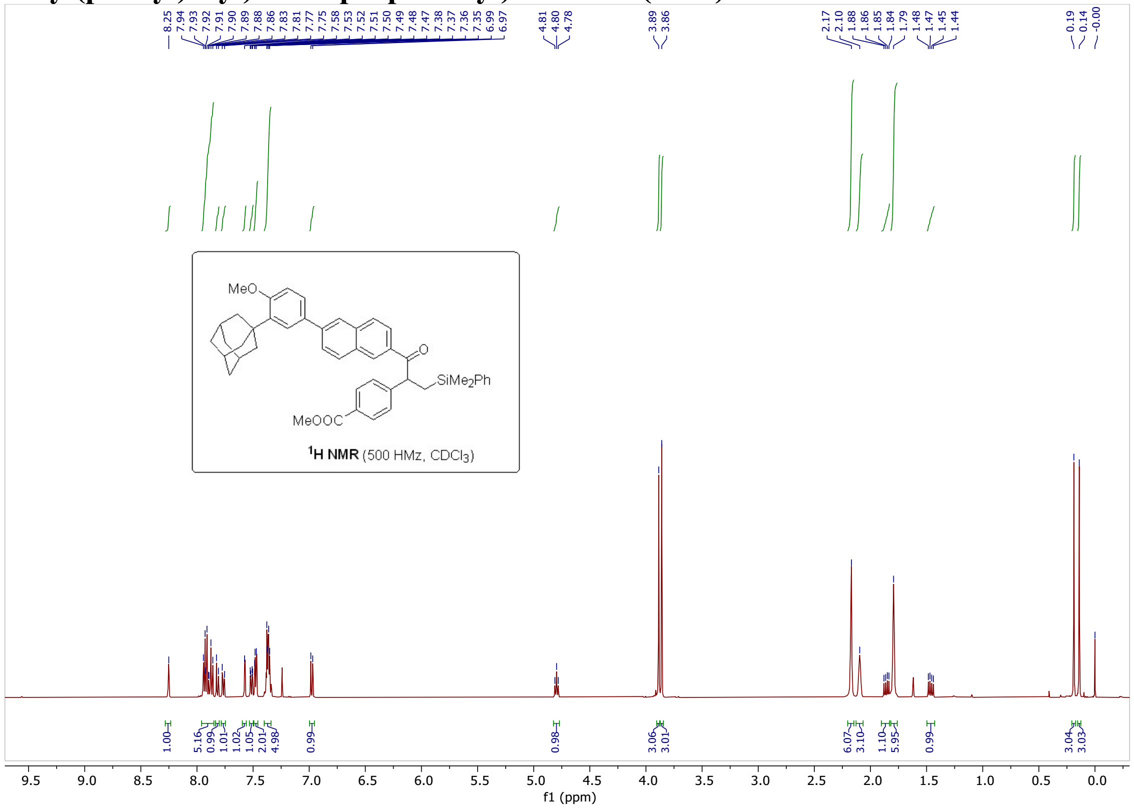

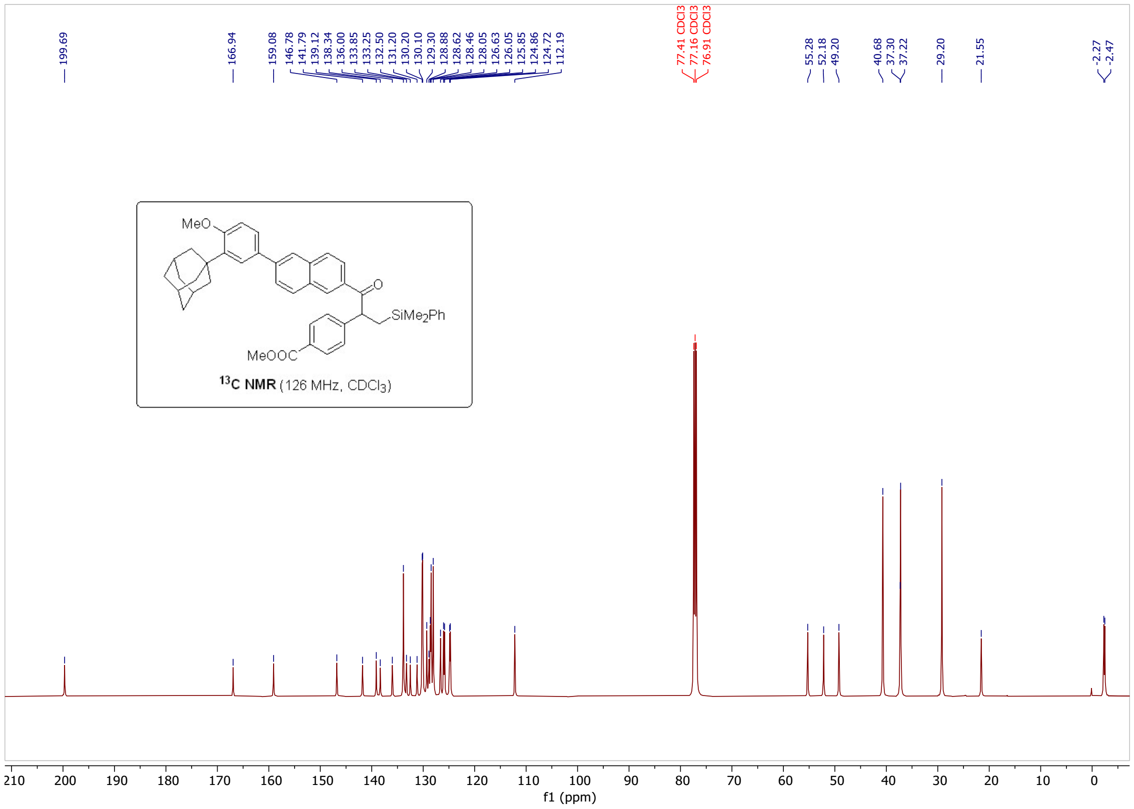

methyl 4-(3-(dimethyl(phenyl)silyl)-1-((8R,9S,13S,14S)-13-methyl-17-oxo-7,8,9,11,12,13,14,15,16,17-decahydro-6H-cyclopenta[a]phenanthren-3-yl)-1-oxopropan-2-yl)benzoate (3zca)

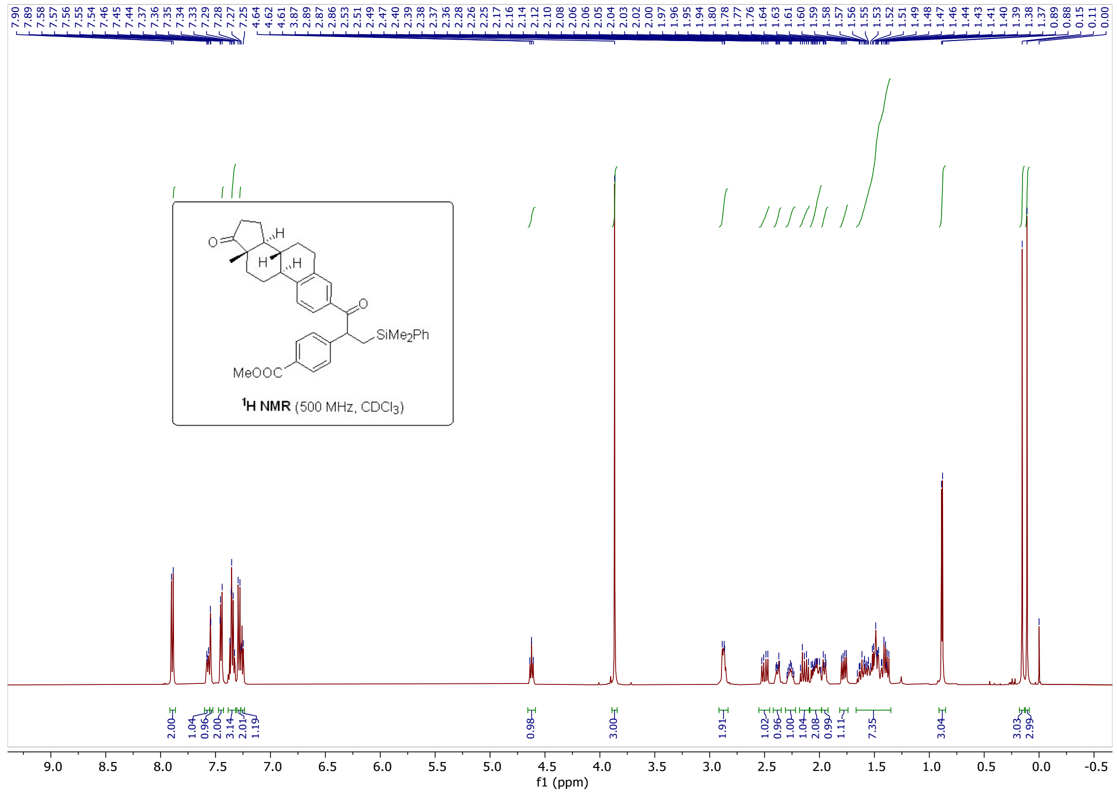

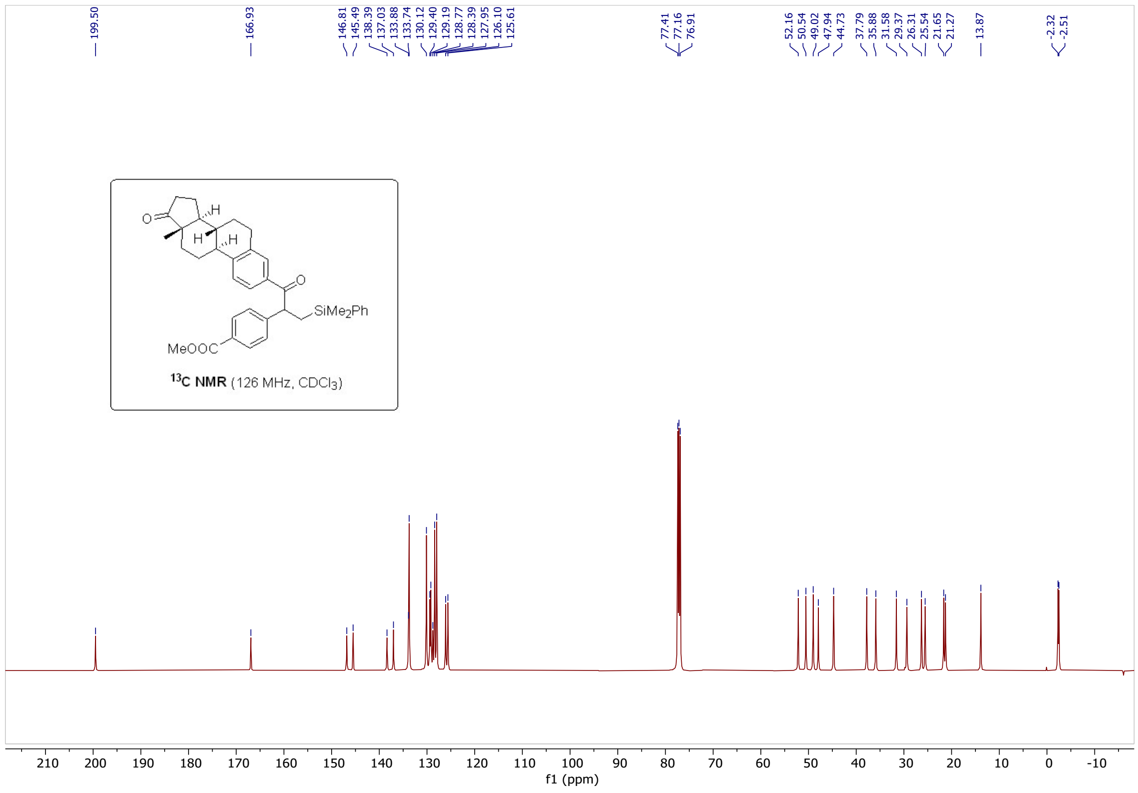

methyl 4-(3-(dimethyl(phenyl)silyl)-1-oxo-1-(3-vinylphenyl)propan-2-yl)benzoate (3zda)

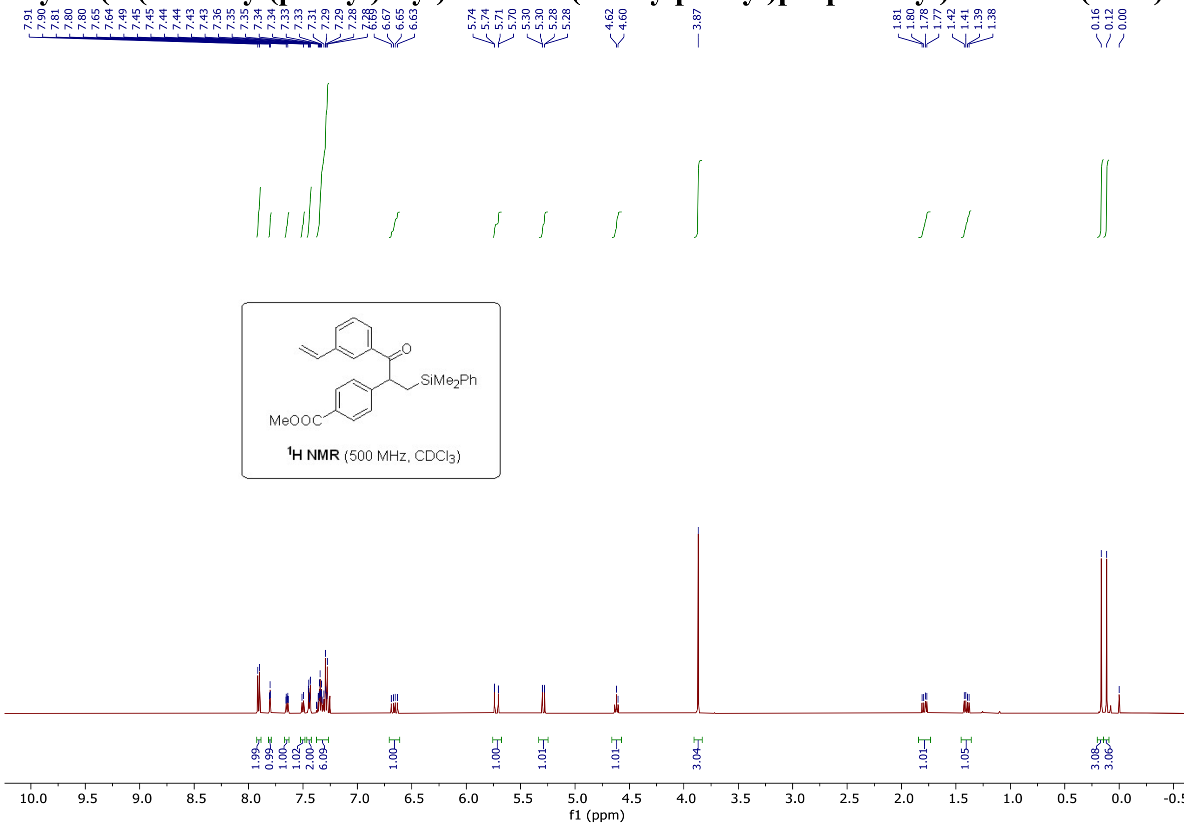

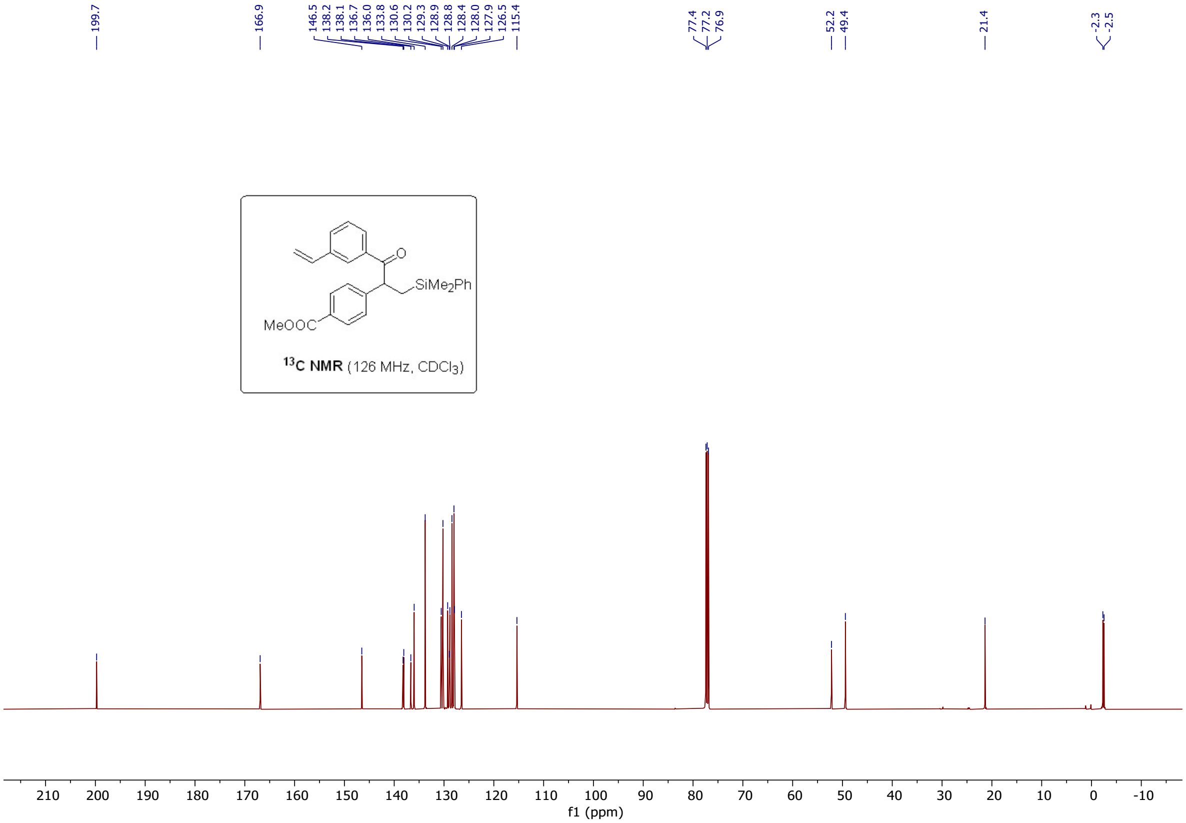

3-hydroxy-1-phenyl-2-(4-(trifluoromethyl)phenyl)propan-1-one (4)

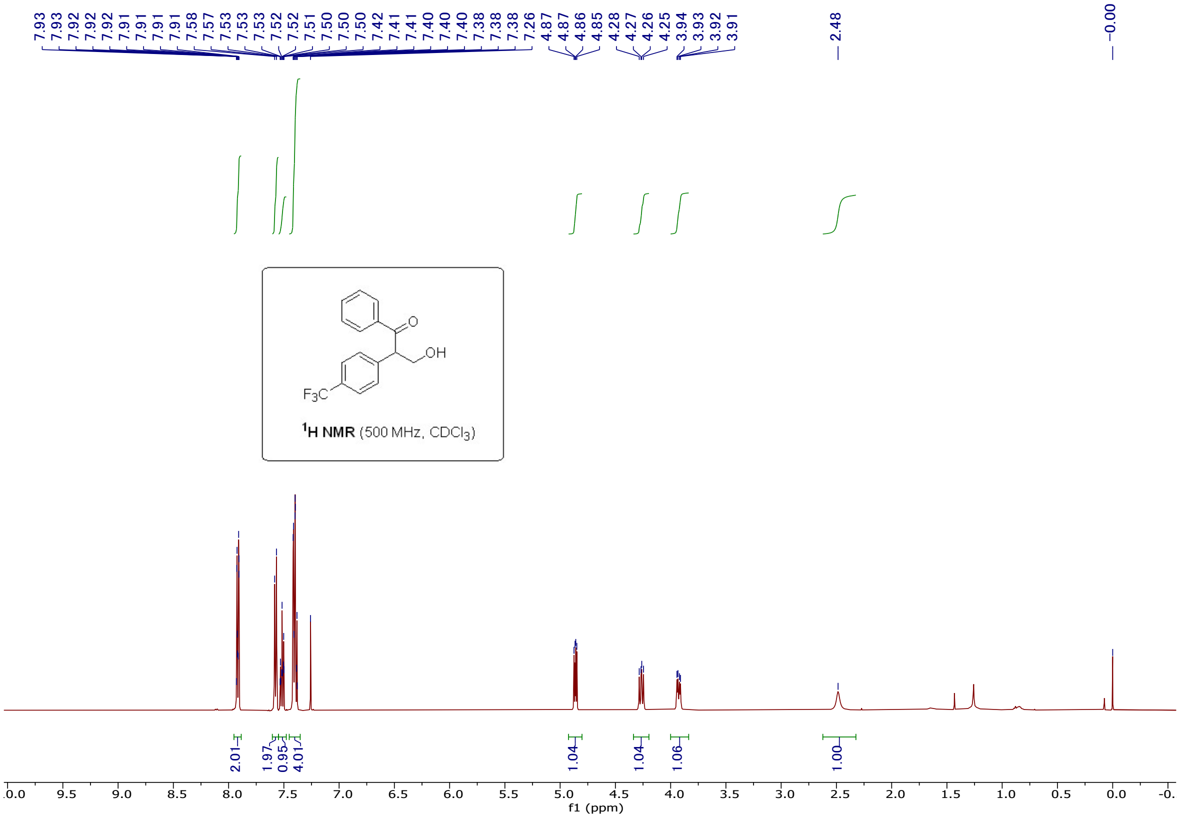

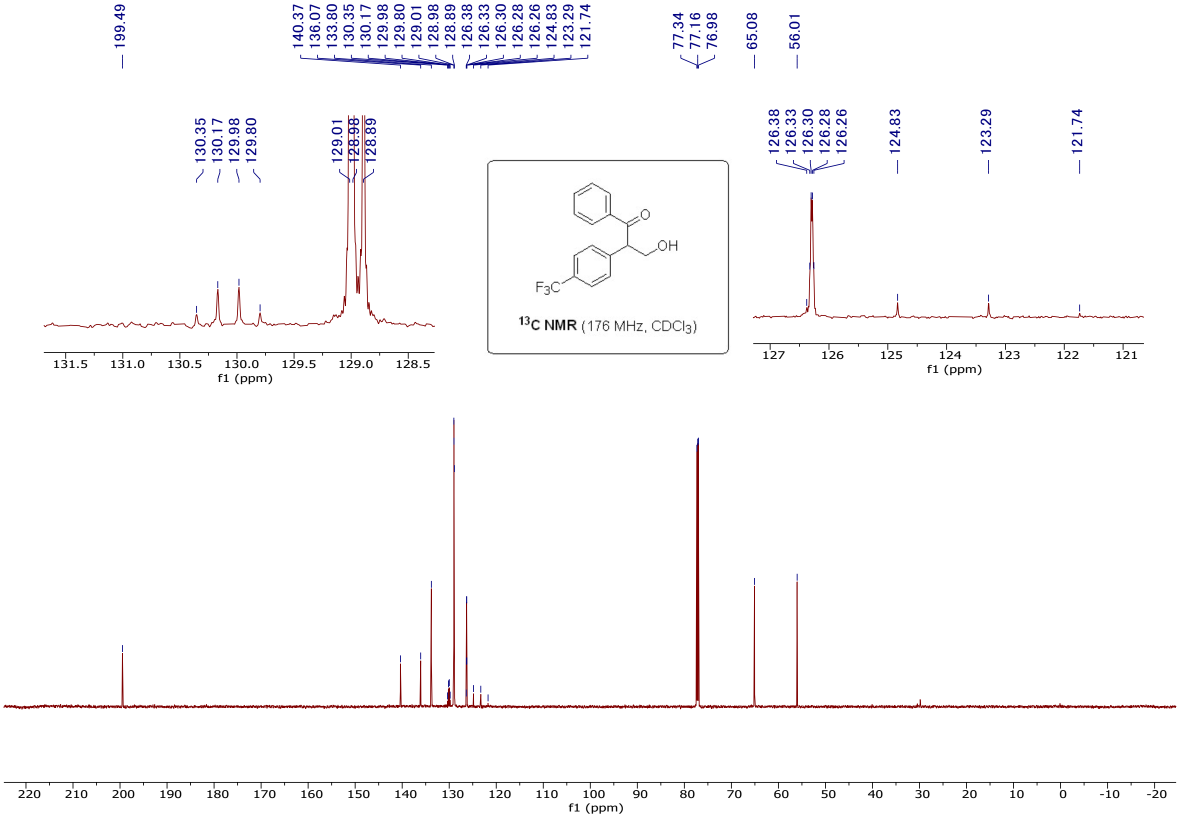

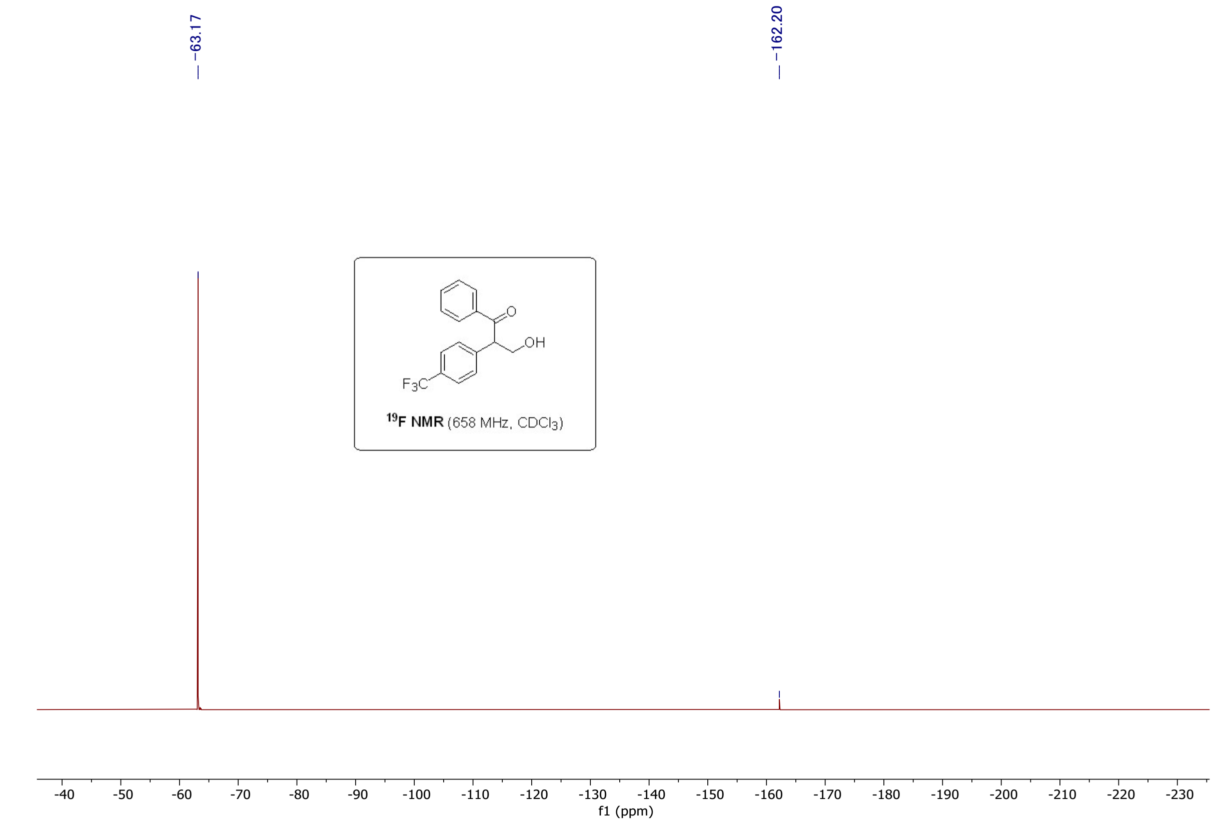

1-phenyl-2-(4-(trifluoromethyl)phenyl)propan-1-one (5)

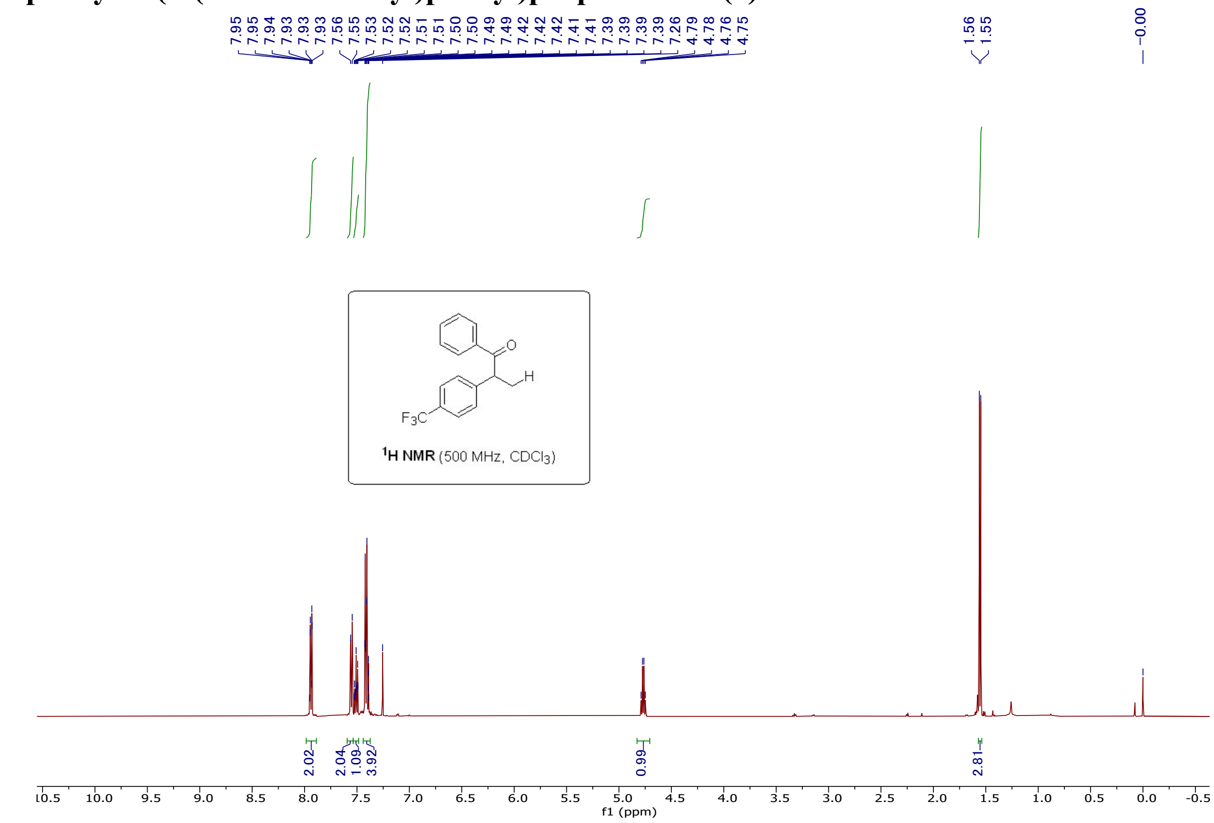

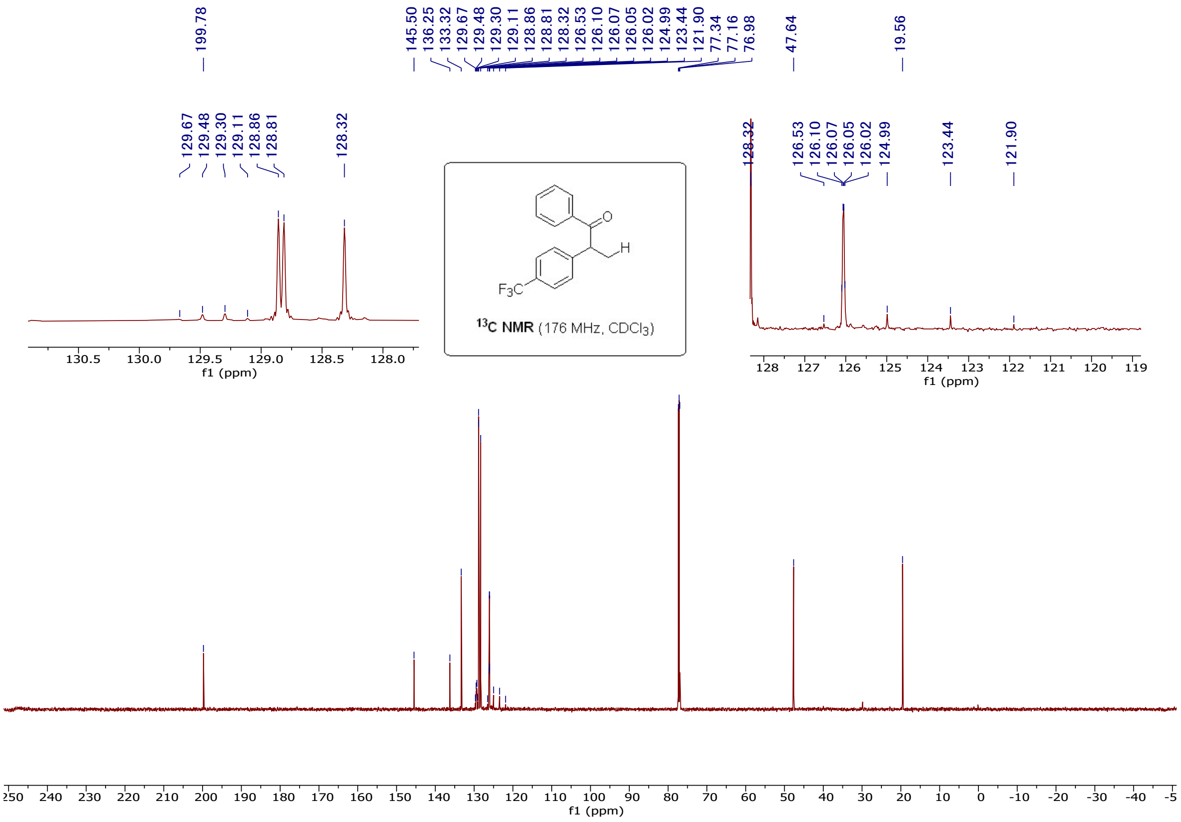
199.78
145.50
136.25
133.32
129.67
129.48
129.30
129.11
128.86
128.81
128.32
126.53
126.10
126.07
126.05
126.02
124.99
123.44
121.90
77.34
77.16
76.98
47.64
19.56
O
H
F3C
13C NMR (176 MHz, CDCl3)
f1 (ppm)

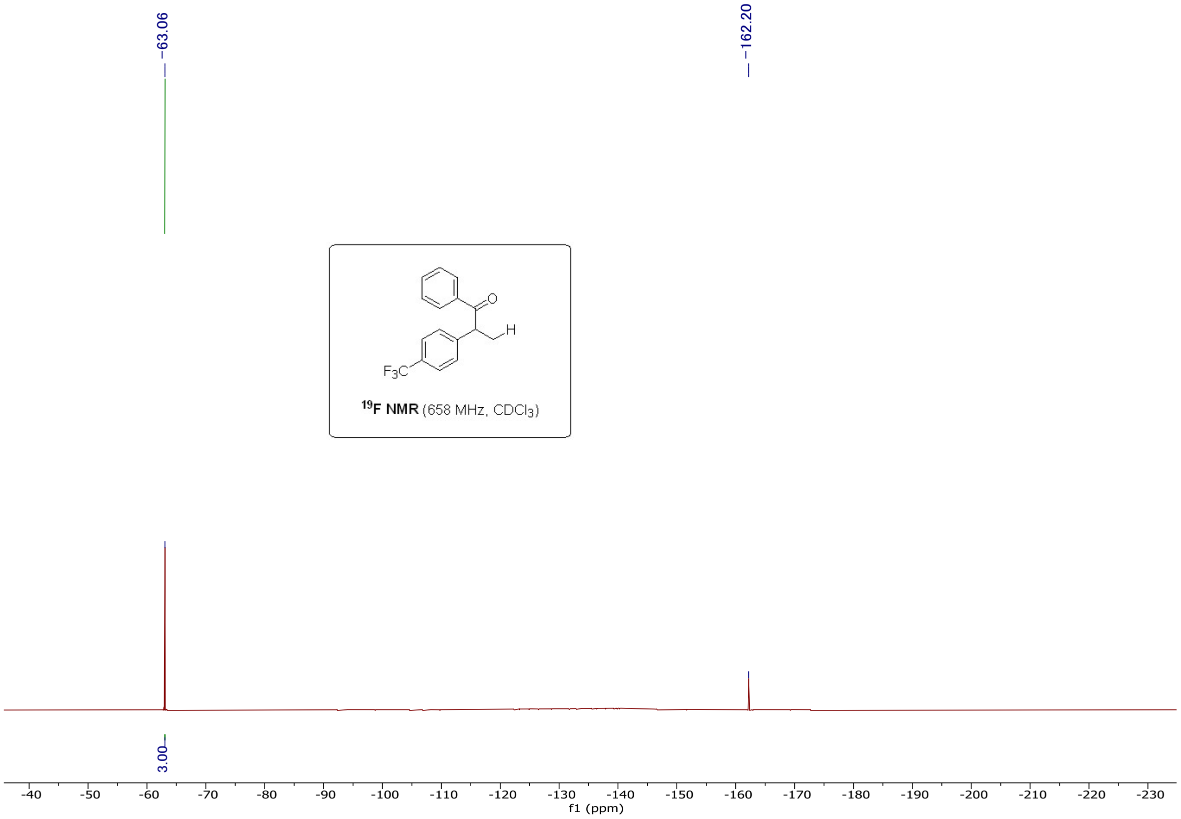
−63.06
−162.20
O
H
F3C
19F NMR (658 MHz, CDCl3)
3.00
f1 (ppm)

1-phenyl-2-(4-(trifluoromethyl)phenyl)prop-2-en-1-one (6)

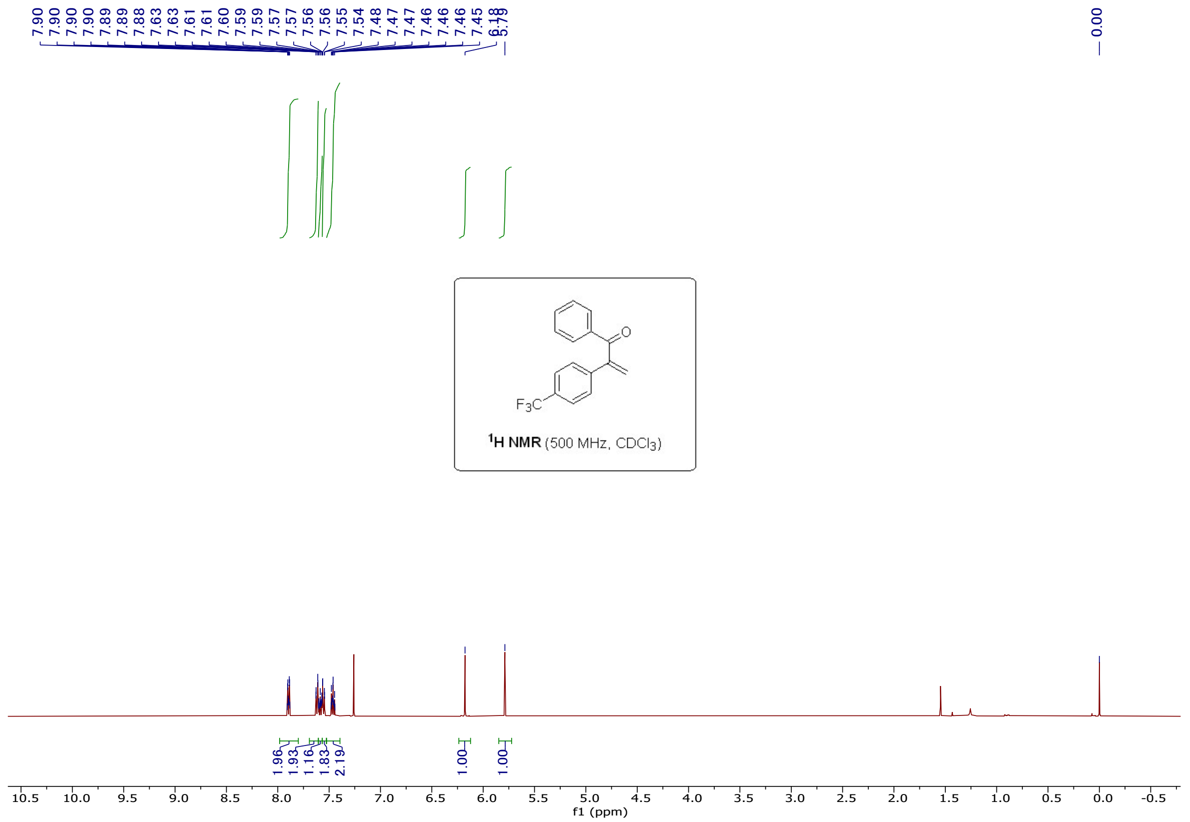

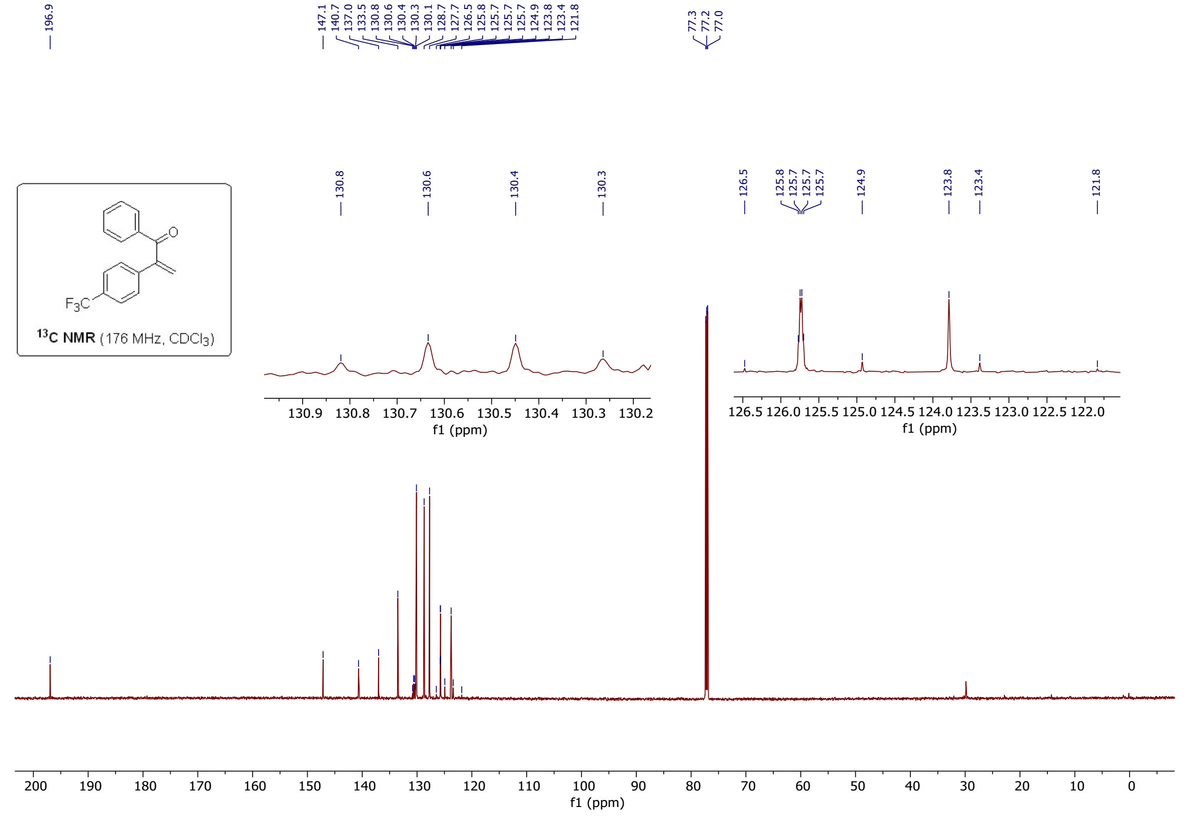

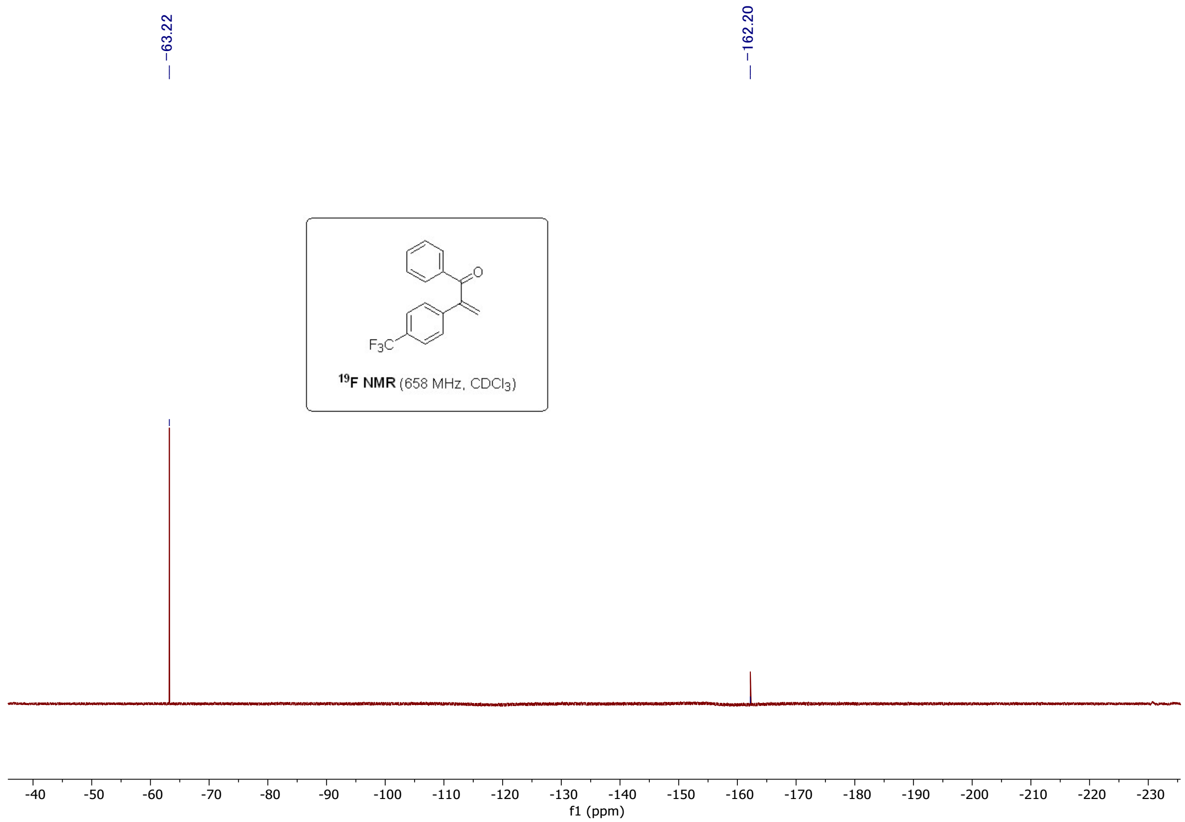

trans-2-phenylcyclopropane-1-carbonyl fluoride (1r)

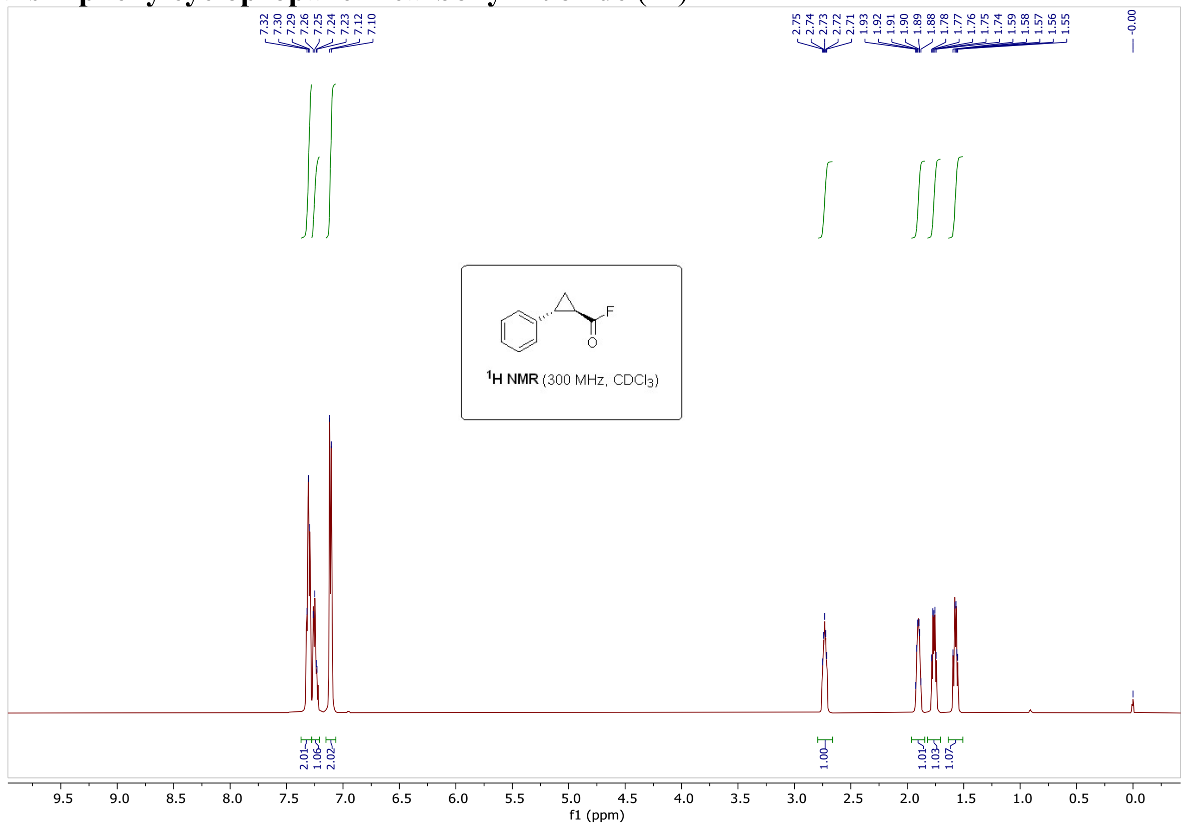

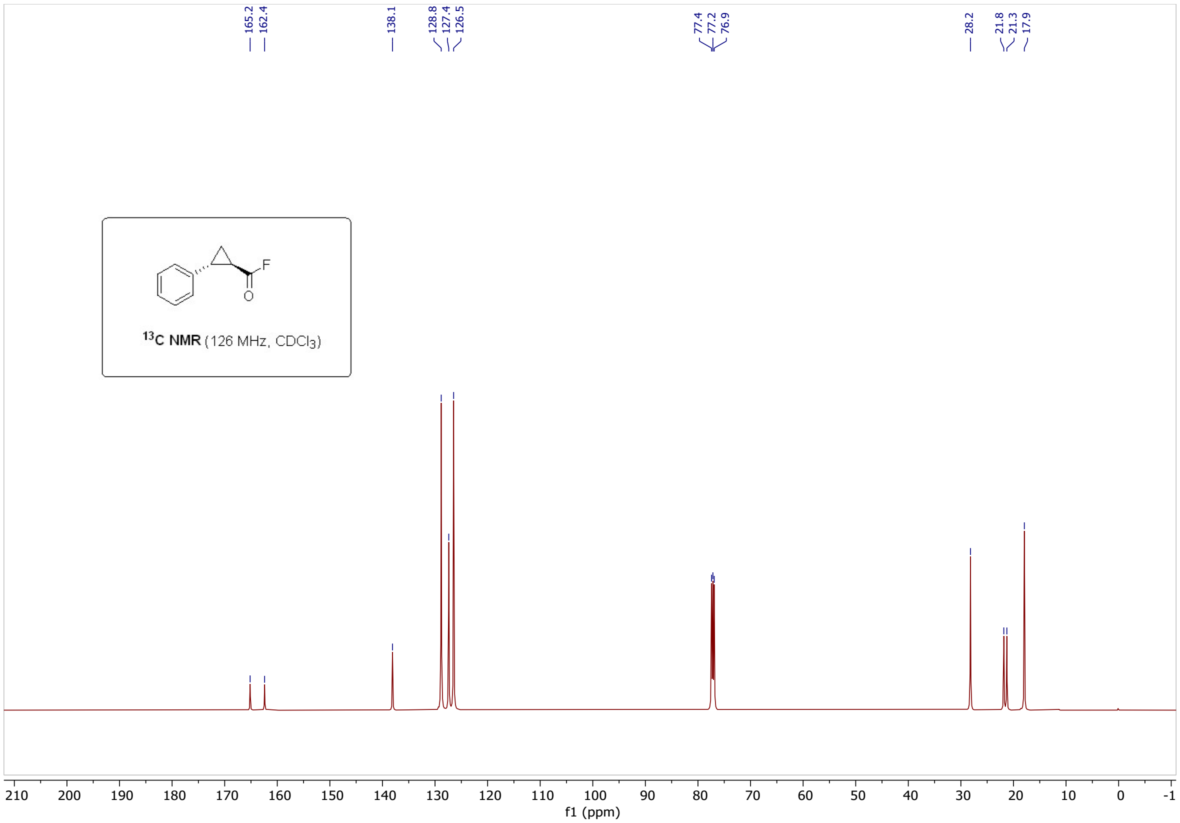

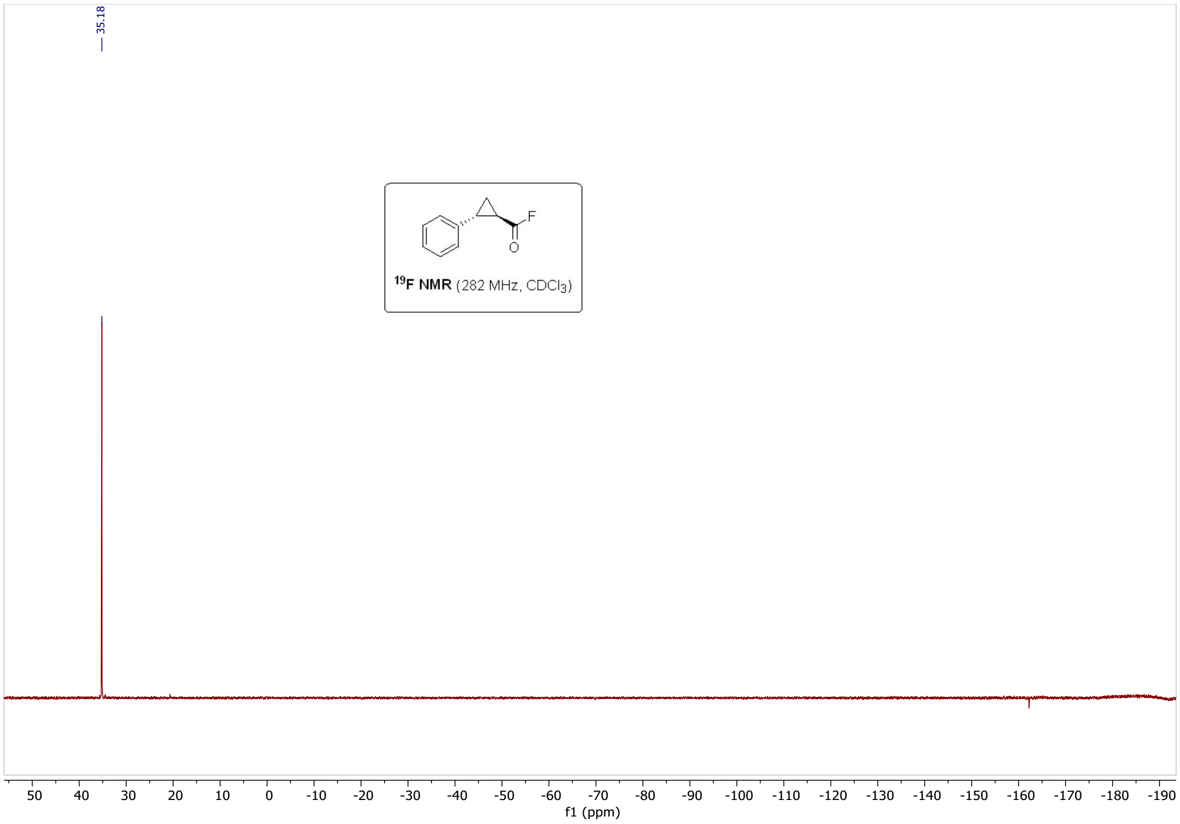

4-((*tert*-butyldimethylsilyl)oxy)benzoyl fluoride (1u)

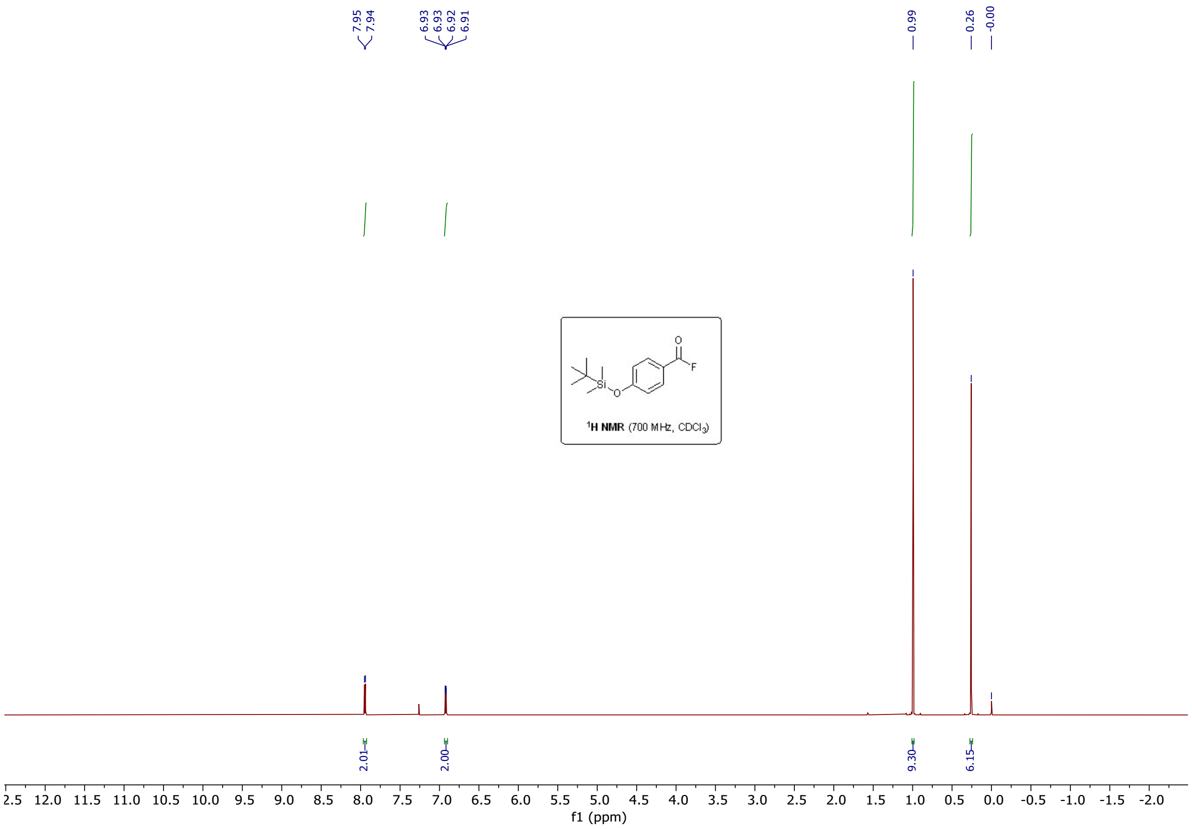
7.95
7.94
6.93
6.93
6.92
6.91
0.99
0.26
-0.00
O
F
Si
O
1H NMR (700 MHz, CDCl3)
2.01
2.00
9.30
6.15
2.5 12.0 11.5 11.0 10.5 10.0 9.5 9.0 8.5 8.0 7.5 7.0 6.5 6.0 5.5 5.0 4.5 4.0 3.5 3.0 2.5 2.0 1.5 1.0 0.5 0.0 -0.5 -1.0 -1.5 -2.0
f1 (ppm)

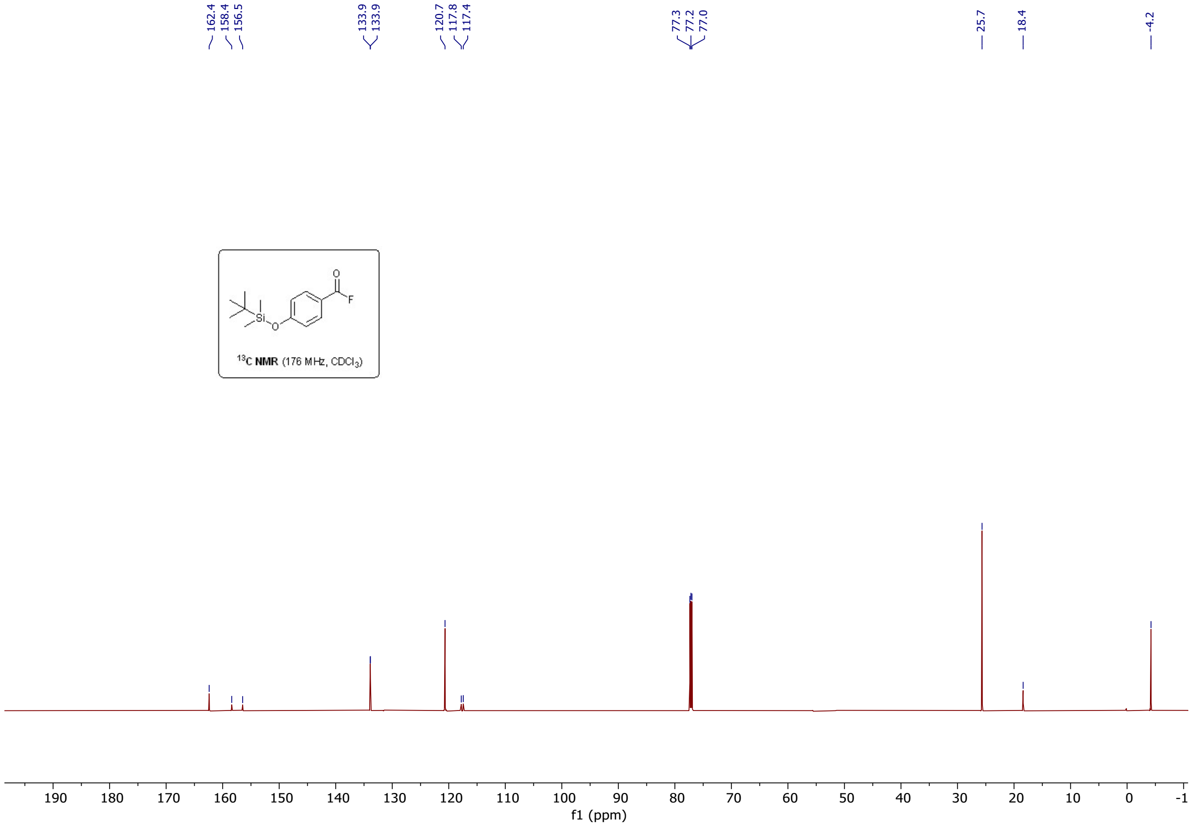
162.4
158.4
156.5
133.9
133.9
120.7
117.8
117.4
77.3
77.2
77.0
25.7
18.4
-4.2
O
F
Si
O
13C NMR (176 MHz, CDCl3)
190 180 170 160 150 140 130 120 110 100 90 80 70 60 50 40 30 20 10 0 -1
f1 (ppm)

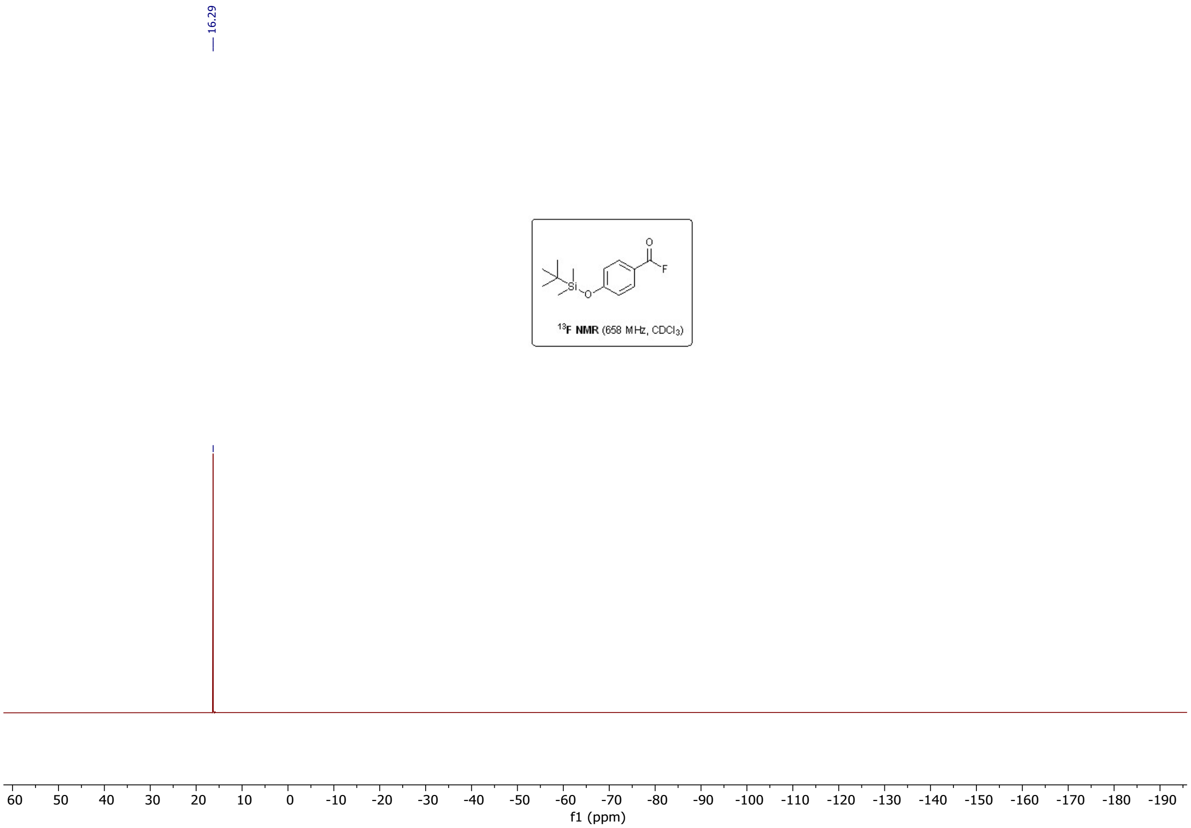

4-(butylamino)benzoyl fluoride (1v)

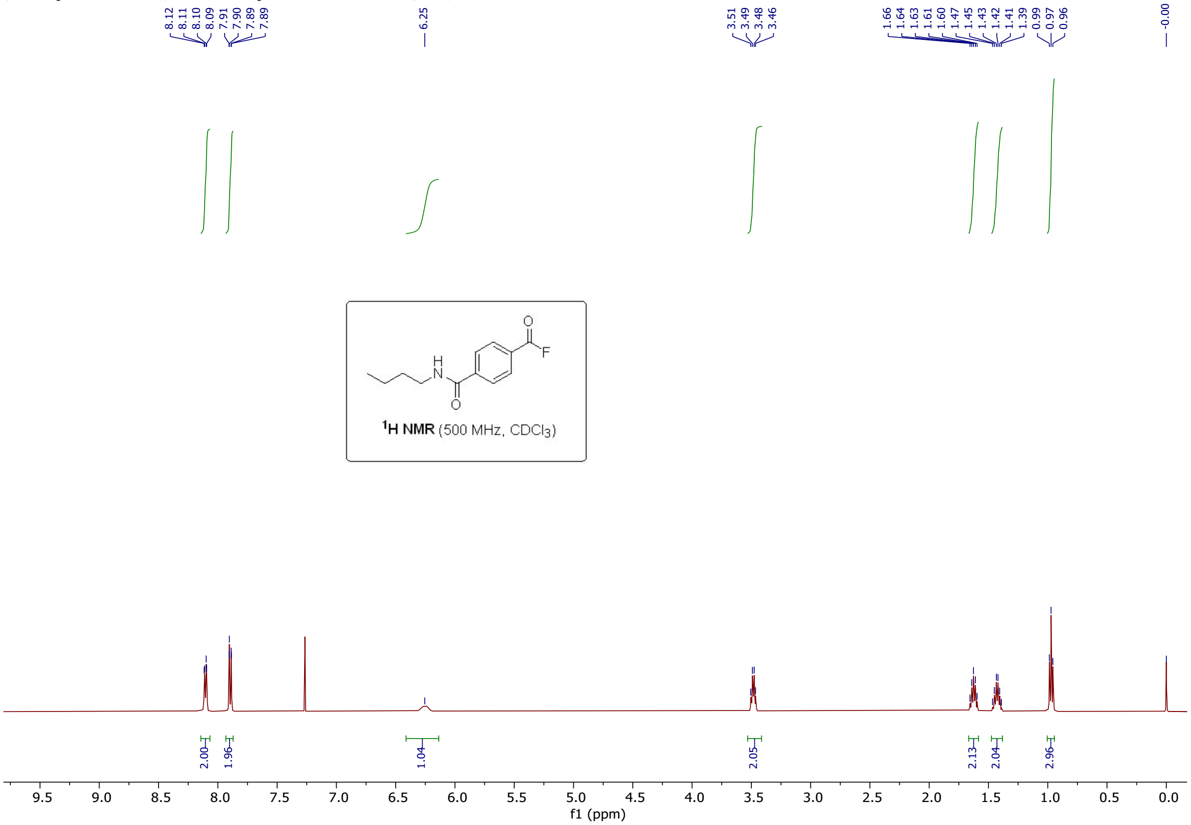

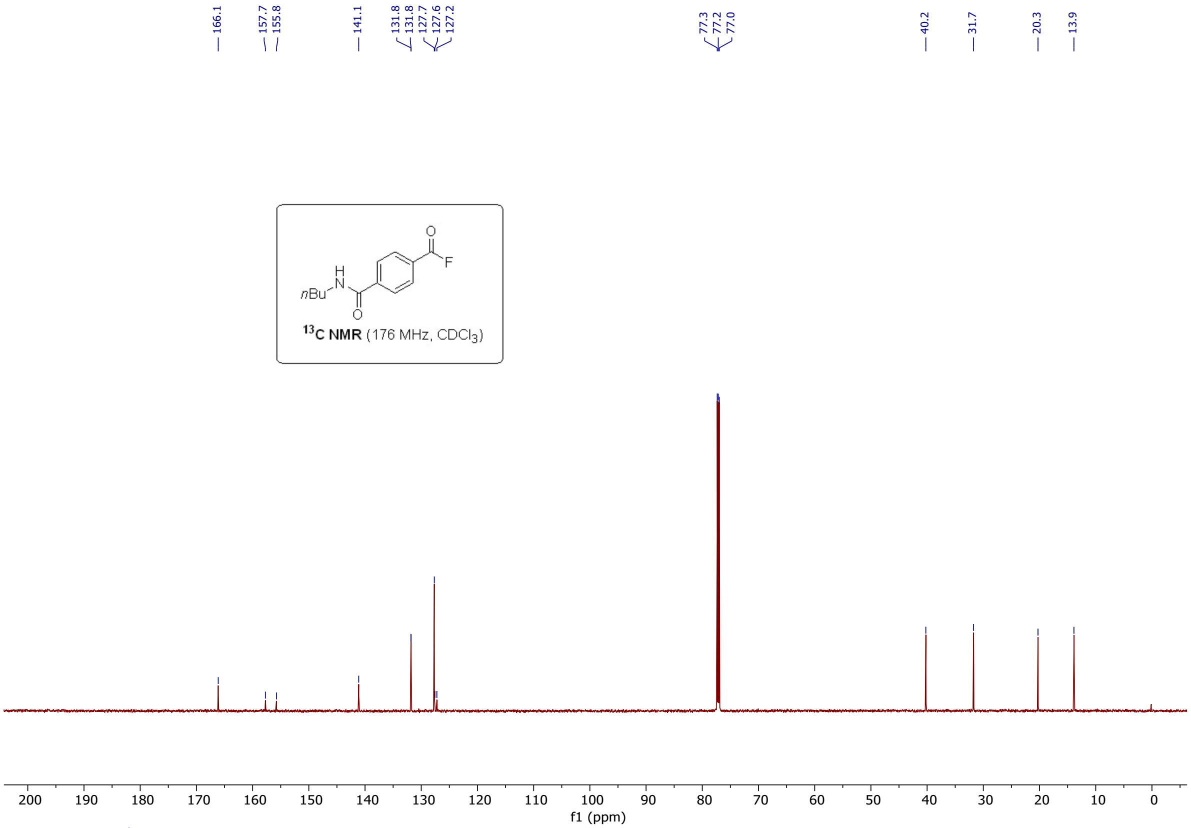

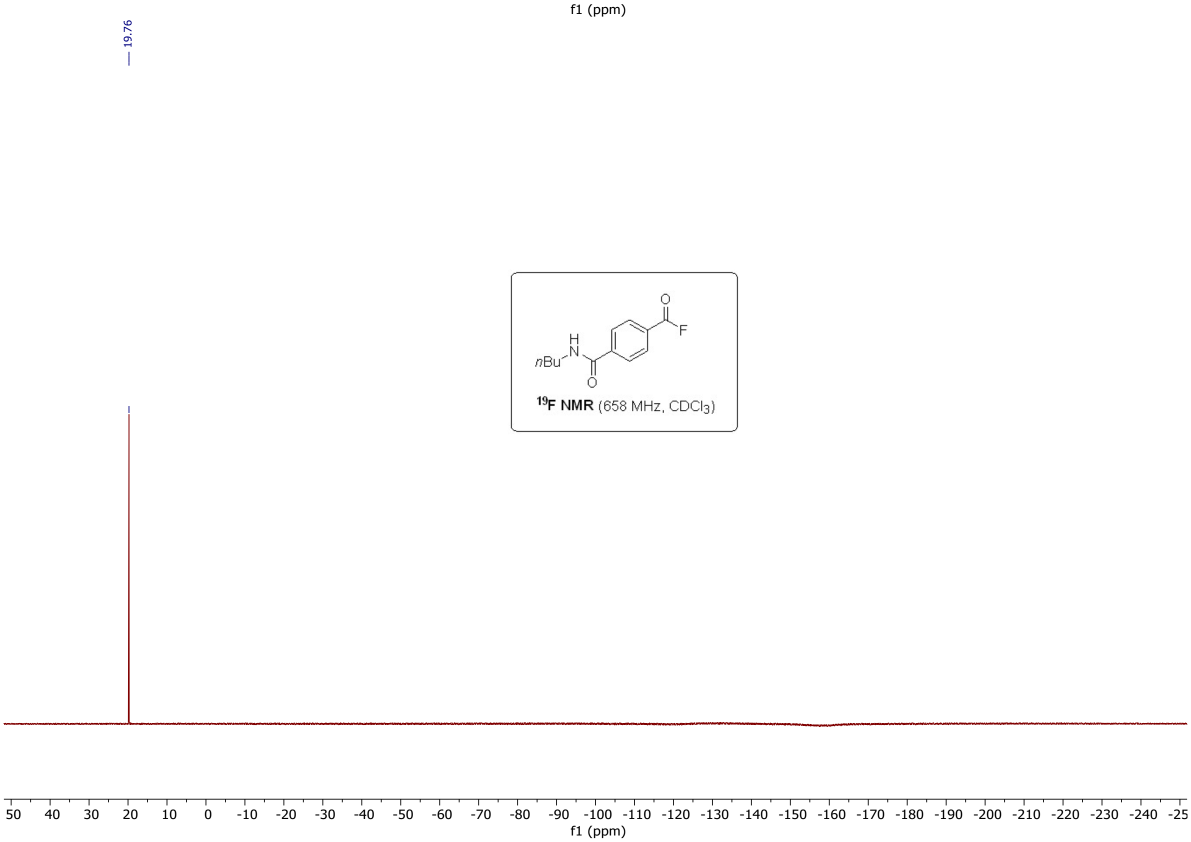

***tert*-butyl (4-(fluorocarbonyl)phenyl)carbamate (1w)**

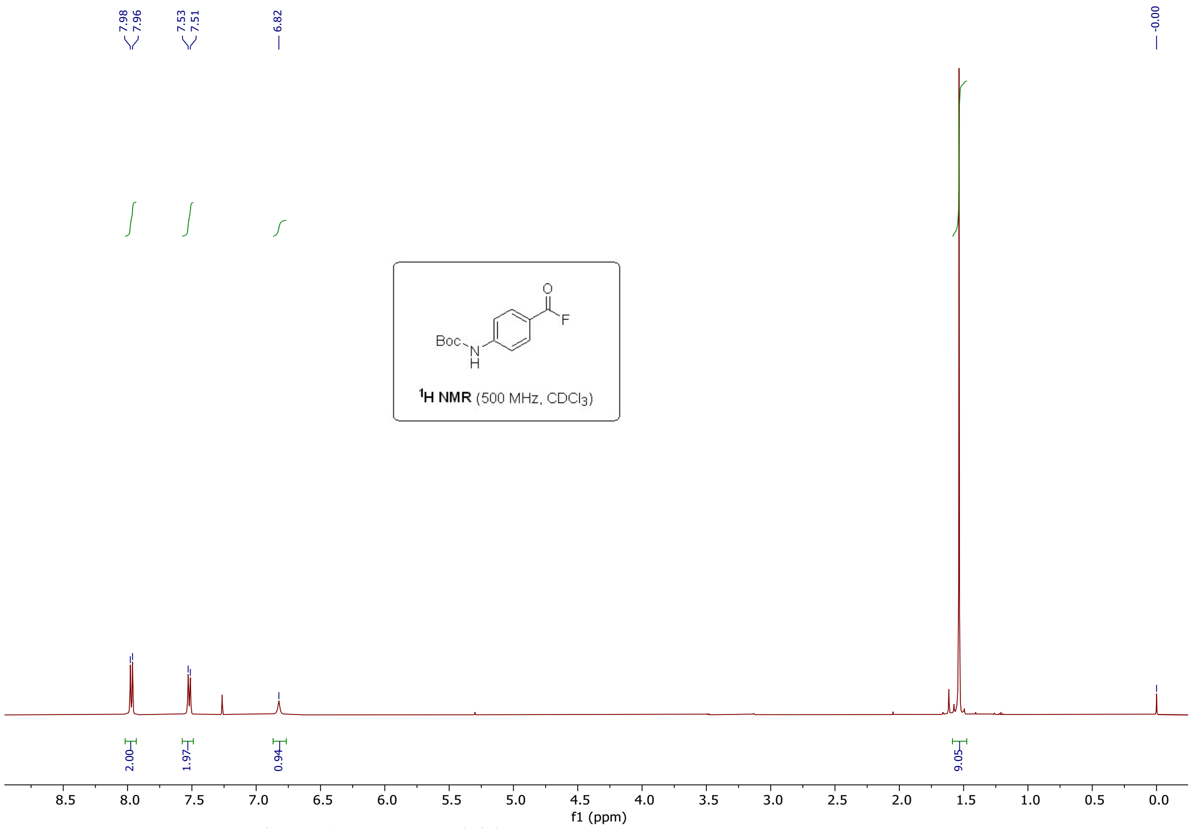
7.98
7.96
7.53
7.51
6.82
-0.00
Boc
N
H
O
F
1H NMR (500 MHz, CDCl3)
2.00
1.97
0.94
9.05
8.5
8.0
7.5
7.0
6.5
6.0
5.5
5.0
4.5
4.0
3.5
3.0
2.5
2.0
1.5
1.0
0.5
0.0
f1 (ppm)

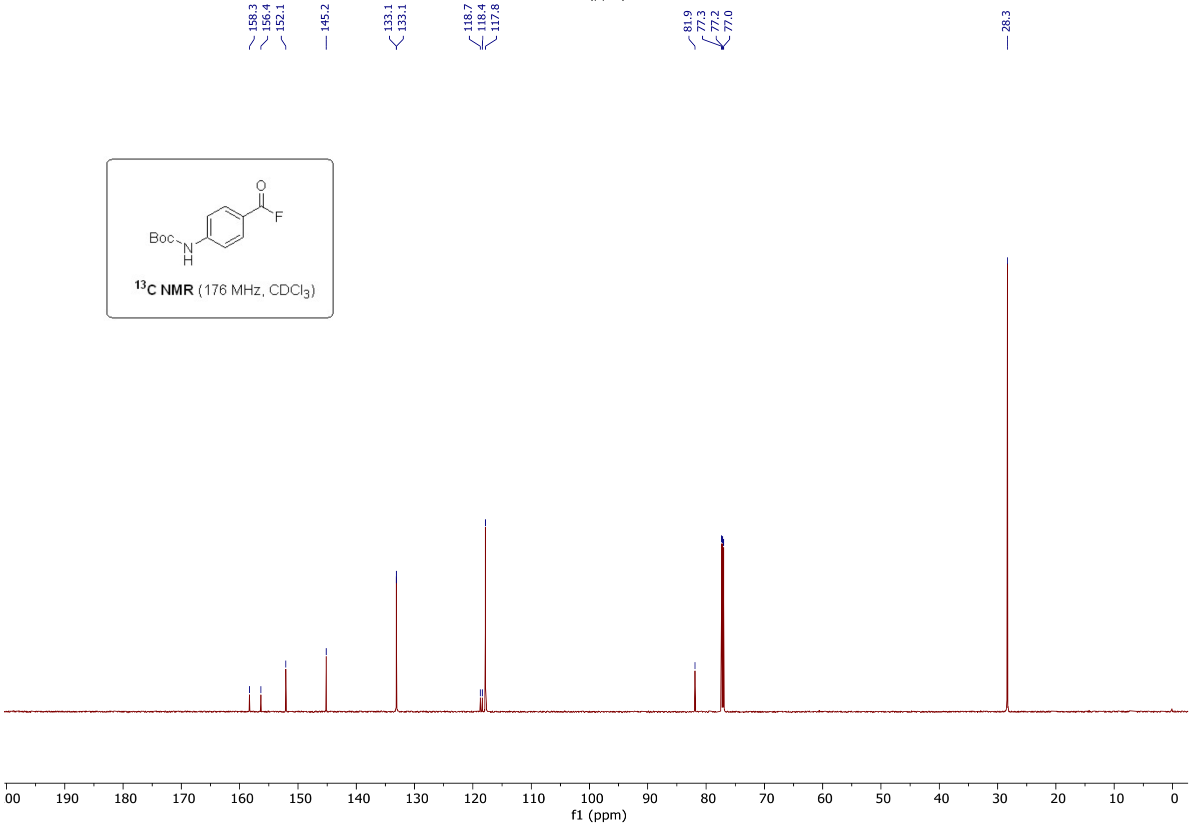
158.3
156.4
152.1
145.2
133.1
133.1
118.7
118.4
117.8
81.9
77.3
77.2
77.0
28.3
Boc
N
H
O
F
13C NMR (176 MHz, CDCl3)
00
190
180
170
160
150
140
130
120
110
100
90
80
70
60
50
40
30
20
10
0
f1 (ppm)

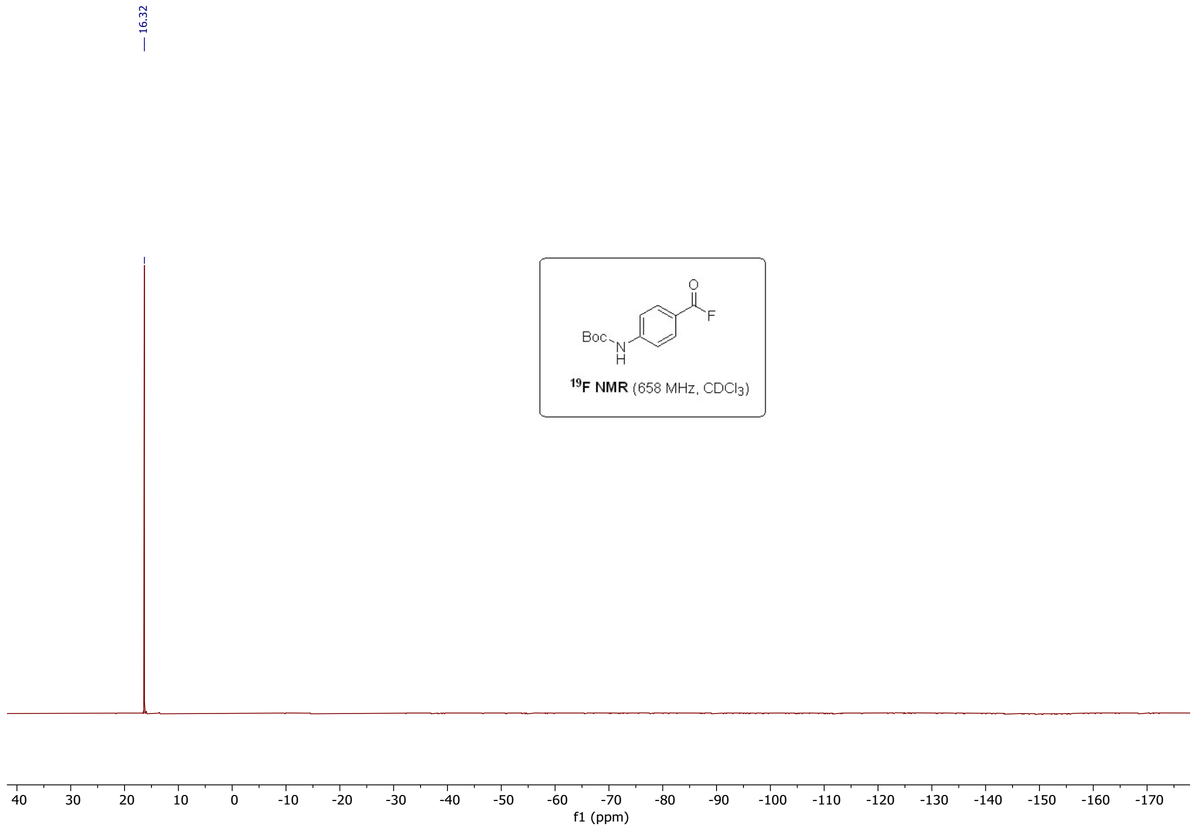

6-(3-(adamantan-1-yl)-4-methoxyphenyl)-2-naphthoyl fluoride (1zb)

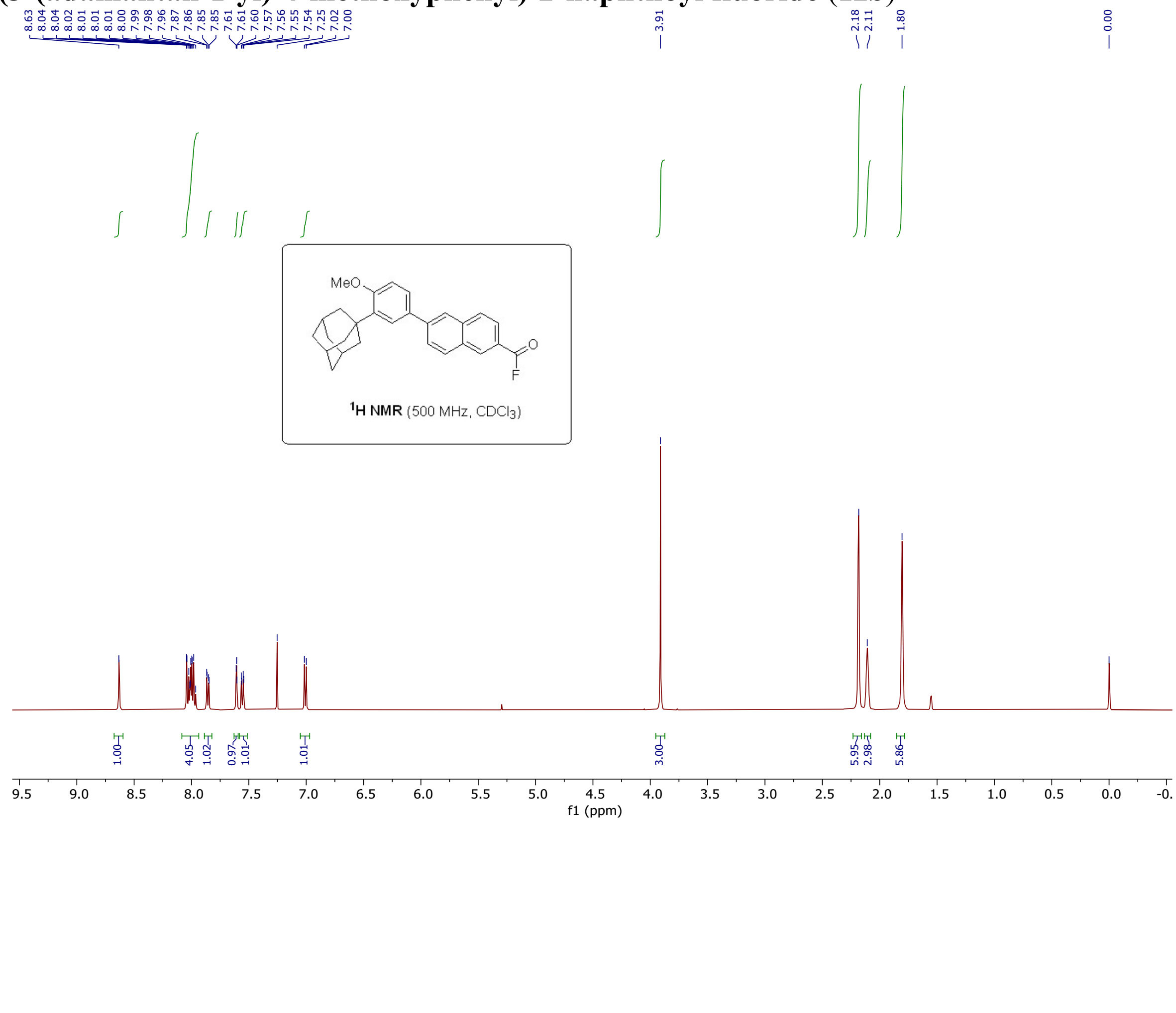

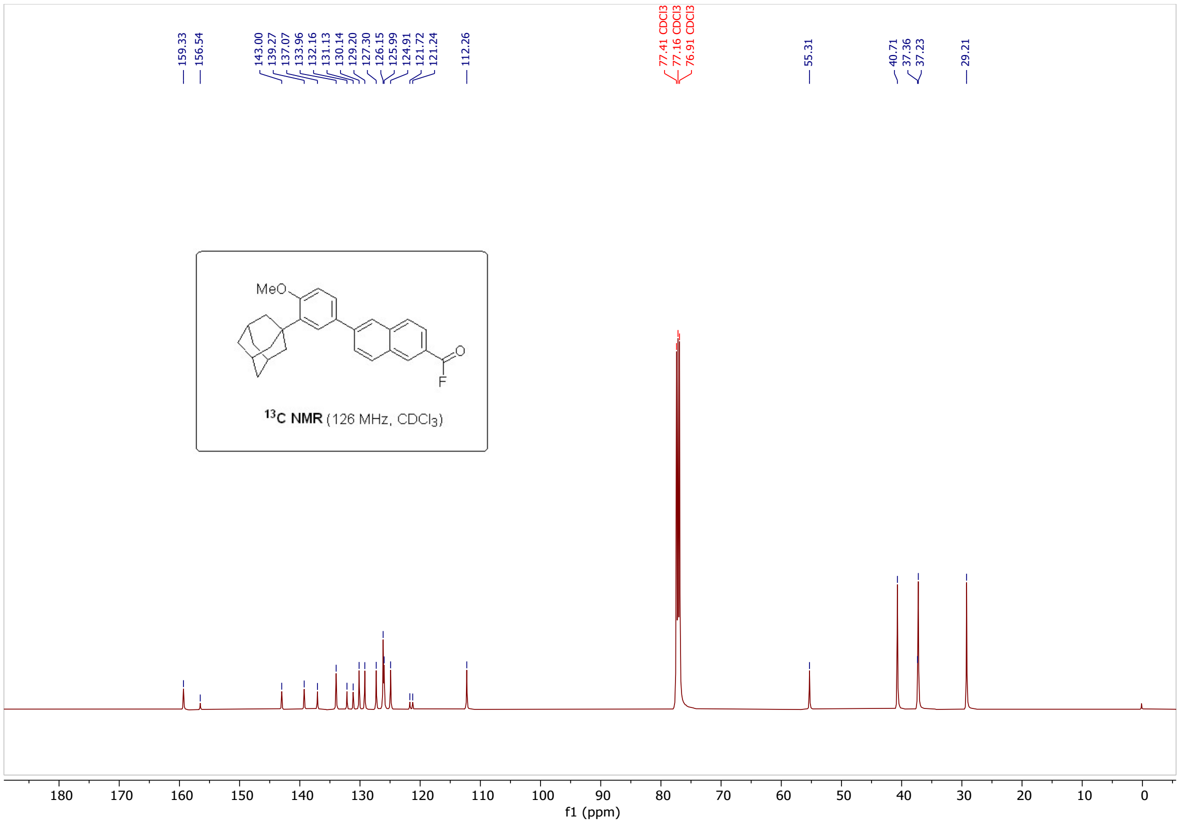

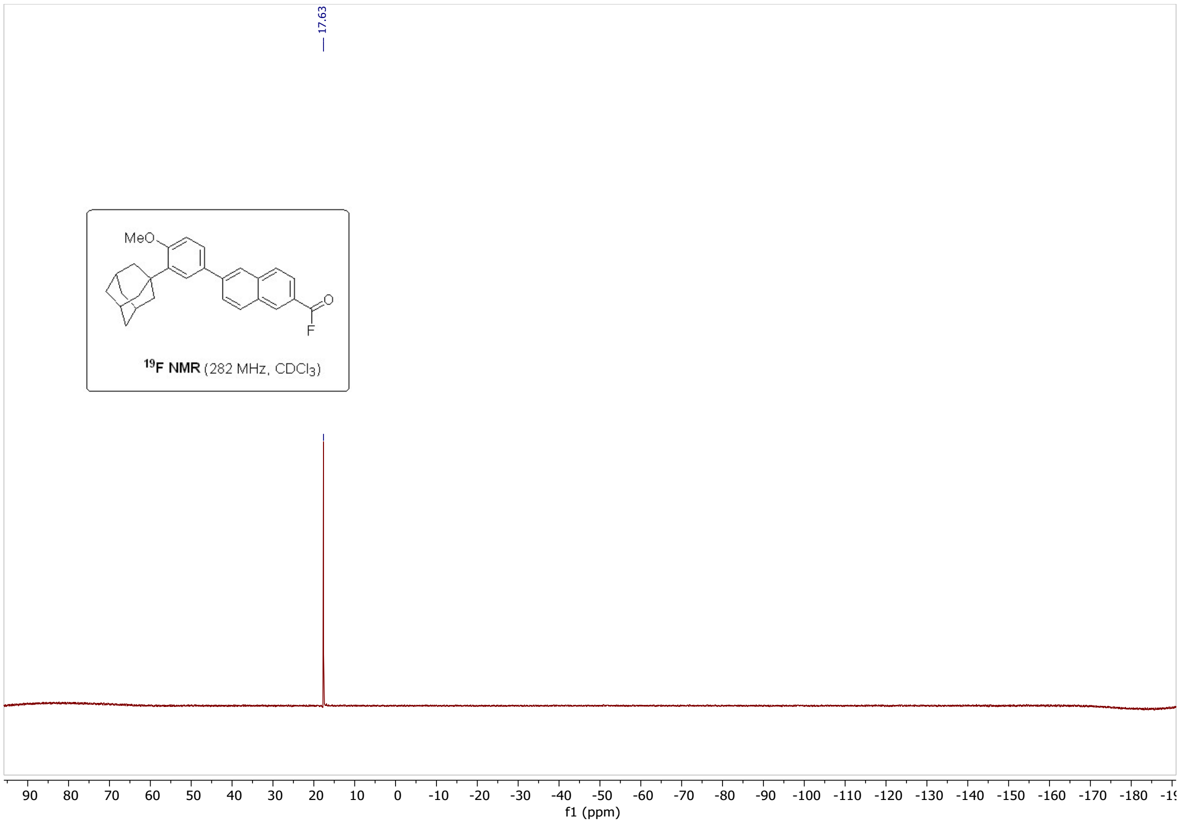

3-vinylbenzoyl fluoride (1zd)

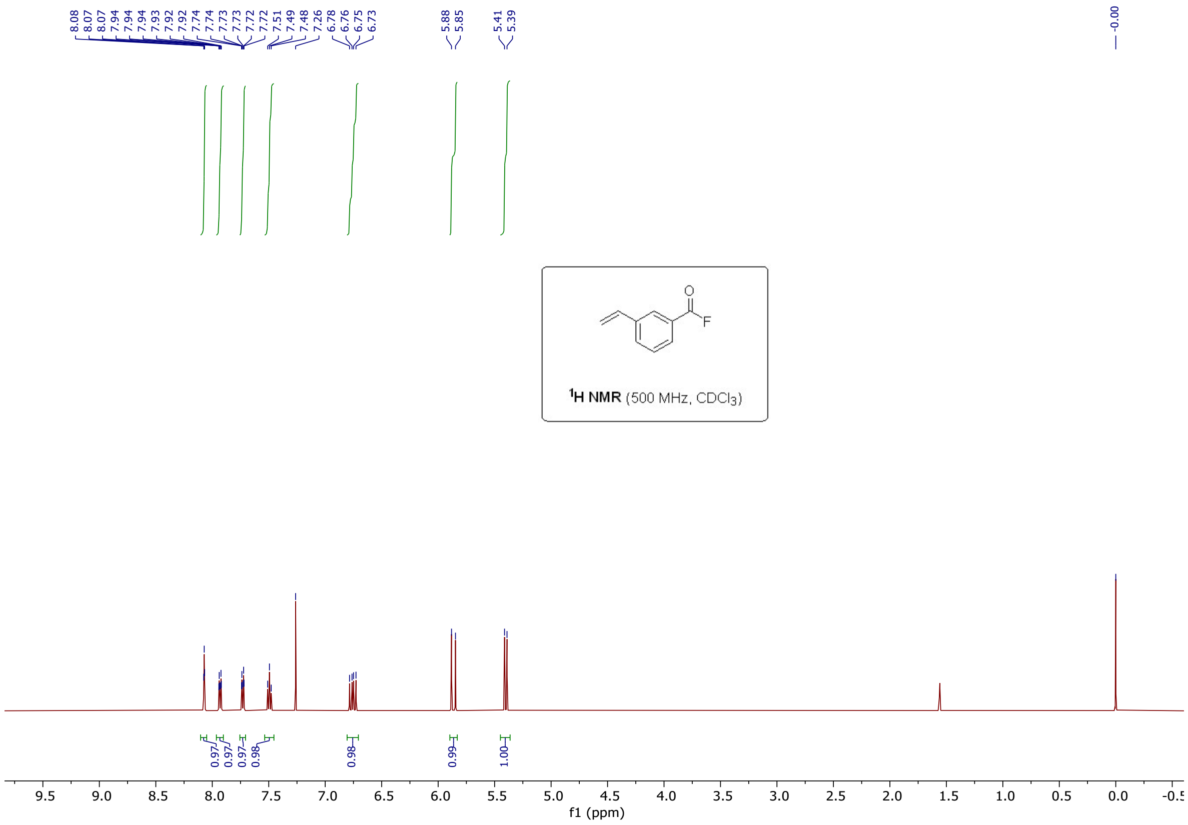
8.08 8.07 8.07 7.94 7.94 7.94 7.93 7.92 7.92 7.74 7.74 7.73 7.73 7.72 7.72 7.51 7.49 7.48 7.26 6.78 6.76 6.75 6.73
5.88 5.85
5.41 5.39
-0.00
O
F
¹H NMR (500 MHz, CDCl₃)
0.97 0.97 0.97 0.98 0.98 0.99 1.00
f1 (ppm)

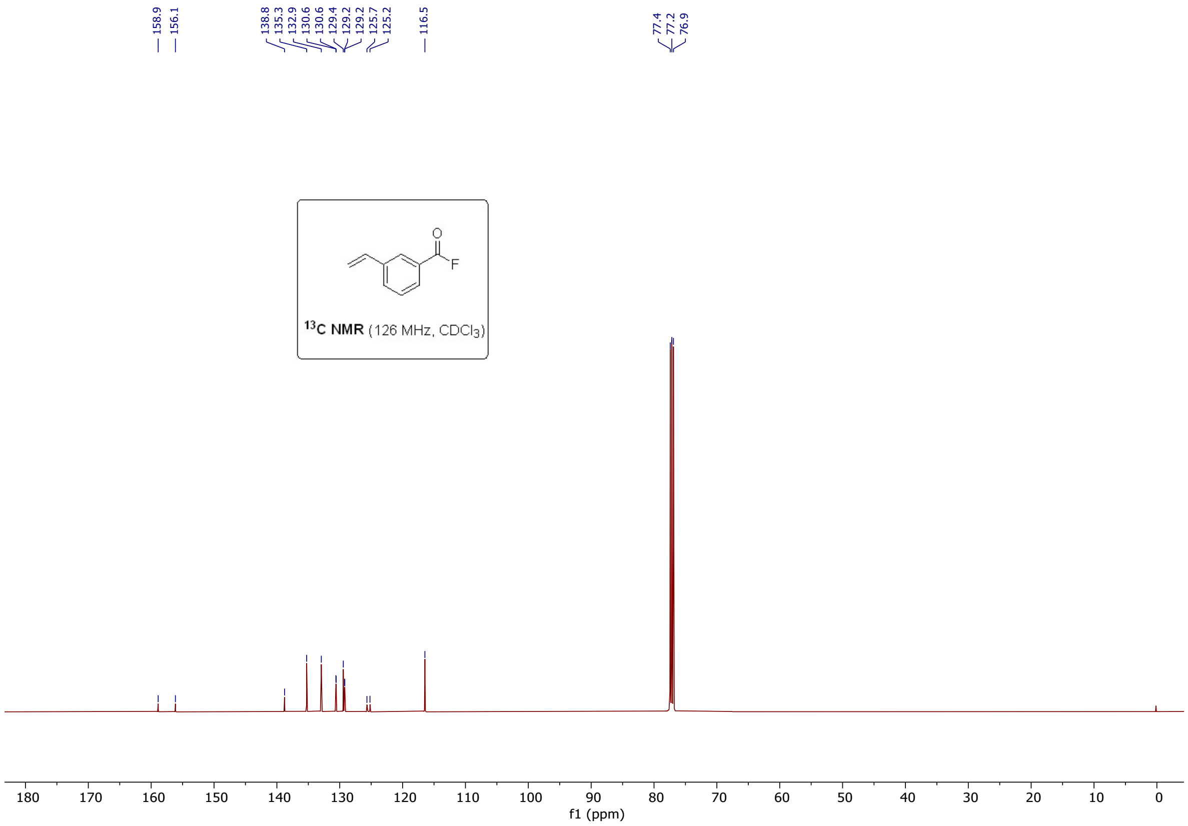
158.9 156.1
138.8 135.3 132.9 130.6 130.6 129.4 129.2 129.2 125.7 125.2
116.5
77.4 77.2 76.9
O
F
¹³C NMR (126 MHz, CDCl₃)
f1 (ppm)

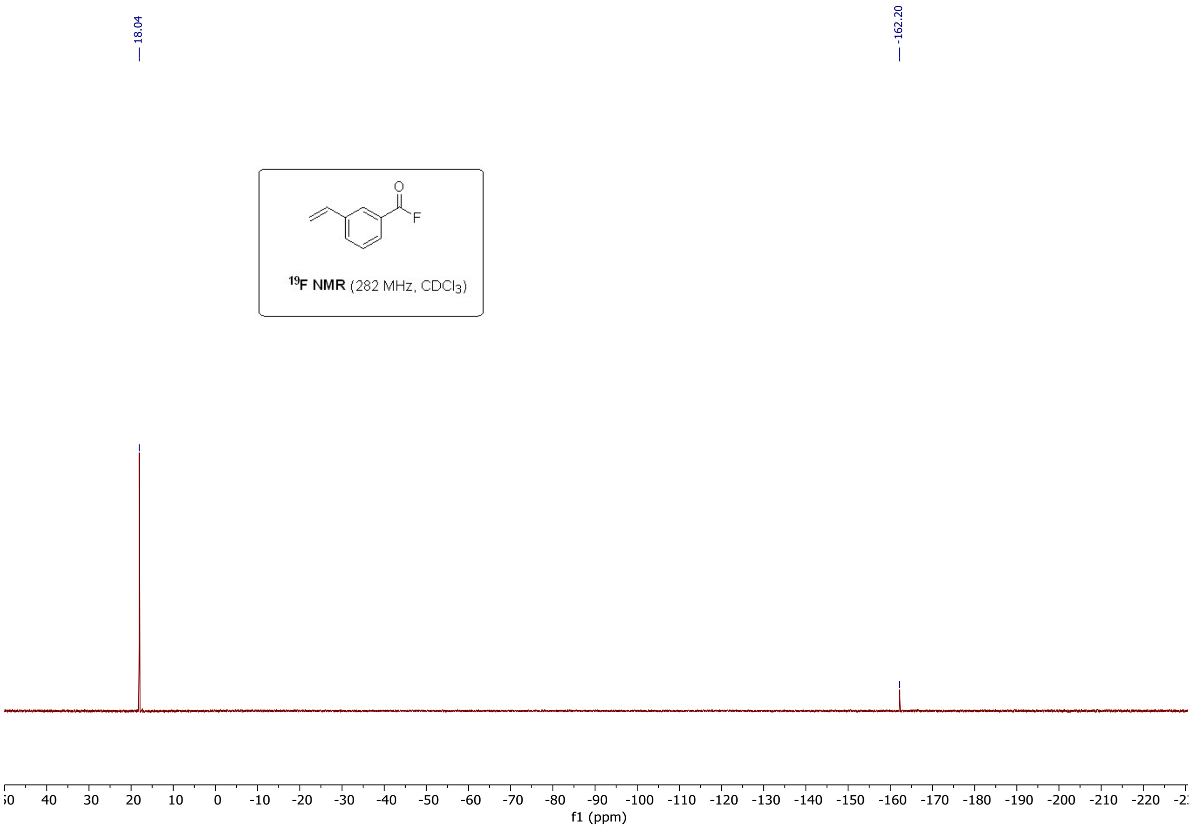

18.04
-162.20
O
F
19F NMR (282 MHz, CDCl3)
40 30 20 10 0 -10 -20 -30 -40 -50 -60 -70 -80 -90 -100 -110 -120 -130 -140 -150 -160 -170 -180 -190 -200 -210 -220
f1 (ppm)
